# The Rational Design of Reducing Organophotoredox Catalysts Unlocks Proton-Coupled Electron-Transfer and Atom Transfer Radical Polymerization Mechanisms

**DOI:** 10.1021/jacs.2c11364

**Published:** 2023-01-06

**Authors:** Tommaso Bortolato, Gianluca Simionato, Marie Vayer, Cristian Rosso, Lorenzo Paoloni, Edmondo M. Benetti, Andrea Sartorel, David Lebœuf, Luca Dell’Amico

**Affiliations:** § Department of Chemical Sciences, University of Padova, Via Marzolo 1, 35131, Padova, Italy; ¶ Institut de Science et d’Ingénierie Supramoléculaires (ISIS), CNRS UMR 7006, Université de Strasbourg, 8 allée Gaspard Monge, 67000Strasbourg, France; ∥ Dipartimento di Fisica e Astronomia G. Galilei, University of Padova, Via Marzolo 8, 35131, Padova, Italy

## Abstract

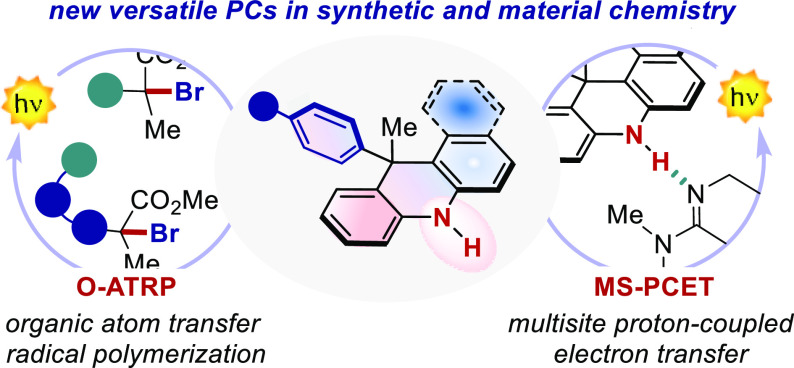

Photocatalysis has become a prominent tool in the arsenal of organic chemists to develop and (re)imagine transformations. However, only a handful of versatile organic photocatalysts (PCs) are available, hampering the discovery of new reactivities. Here, we report the design and complete physicochemical characterization of 9-aryl dihydroacridines (9ADA) and 12-aryl dihydrobenzoacridines (12ADBA) as strong reducing organic PCs. Punctual structural variations modulate their molecular orbital distributions and unlock locally or charge-transfer (CT) excited states. The PCs presenting a locally excited state showed better performances in photoredox defunctionalization processes (yields up to 92%), whereas the PCs featuring a CT excited state produced promising results in atom transfer radical polymerization under visible light (up to 1.21 *Đ*, and 98% I*). Unlike all the PC classes reported so far, 9ADA and 12ADBA feature a free NH group that enables a catalytic multisite proton-coupled electron transfer (MS-PCET) mechanism. This manifold allows the reduction of redox-inert substrates including aryl, alkyl halides, azides, phosphate and ammonium salts (*E*_red_ up to −2.83 vs SCE) under single-photon excitation. We anticipate that these new PCs will open new mechanistic manifolds in the field of photocatalysis by allowing access to previously inaccessible radical intermediates under one-photon excitation.

## Introduction

Over the past decades, the design and development of new catalysts has been a driving force for synthetic chemists to enable new reaction manifolds toward compounds of high interest in fine chemical synthesis.^1^ In this context, the current development of photocatalysis has set previously inaccessible mechanistic vistas,^2^ while allowing operation under mild and sustainable reaction conditions.^3^ In particular, the identification and use of simple and purely organic molecules as photocatalysts (PCs) has become a major goal in the chemical community.^4^ Organic PCs present clear advantages with respect to their metal counterparts (e.g., Ru- or Ir-complexes), including (i) their higher accessibility and sustainability,^3c^ (ii) their overall lower price,^4^ and (iii) a higher tunability of their scaffolds,^5^ which enables (iv) a more direct assessment of the structure–property relationships.^6^ Likewise the metal-to-ligand charge transfer (MLCT) in metal complexes, organic PCs can access a charge transfer (CT) excited state. The CT excited state occurs when the lowest unoccupied molecular orbital (LUMO) and the highest occupied molecular orbital (HOMO) are spatially separated in the molecule,^5c^ and involves a directional electron movement from the HOMO to the LUMO. The new electron distribution grants access to more stable excited states that have an extended lifetime (from ns to μs) as well as more balanced redox potentials.^3c^ In this context, highly reducing organic PCs play a crucial role.^6,7^ These molecules are highly efficient for the activation of alkyl and aryl halides, ketones, and aldehydes, as well as for the generation of polymers under mild metal-free conditions, thus overcoming metal-contamination issues.^4,7^

**Figure 1:**
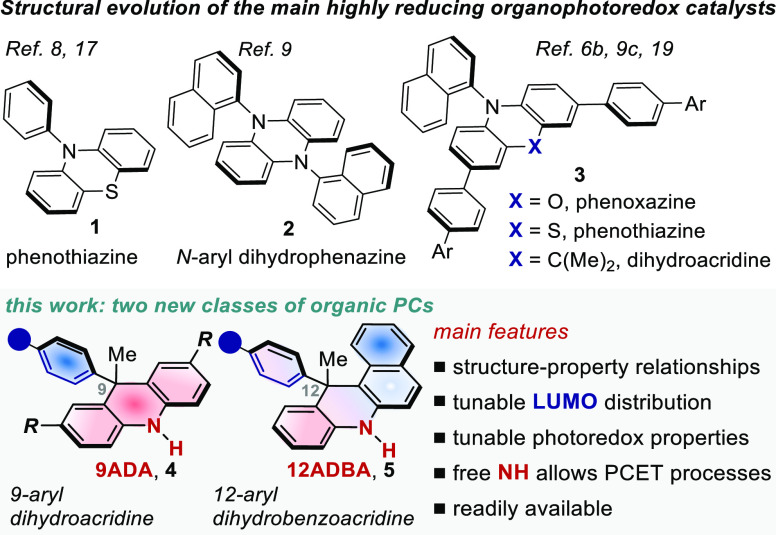
Highly reducing PCs previously reported and our new classes of organic PCs disclosed.

The preliminary findings in this area have involved the use of phenothiazine (PTH) **1** (Figure 1),^8^ which is still one of the PCs of choice for thermodynamically challenging reductive processes. Nevertheless, the low visible-light absorption of PTH, its poor tunability, and inefficiency under atom transfer radical polymerization (ATRP) processes have encouraged the community to identify different scaffolds.^6b^ In that respect, structural motifs such as **2** and **3** have been recently reported to serve as convenient alternatives to PTH (Figure 1). These scaffolds merge high reducing power with a CT character, rendering their excited-state lifetime longer and their redox properties rationally tunable. Recently, dihydrophenazines **2** have shown their potential in light-driven metal-free polymerization processes.^9^ However, the widespread use of these known PC variants is often hampered by tedious synthetic routes, poor solubility, or low visible-light absorption. More importantly, the classes of PCs **1**–**3** share a moderate versatility when moving from classical oxidative quenching processes to ATRP and vice versa. Hence, the identification of novel structural classes capable of switching from the functionalization of small molecules to the synthesis of polymers, while unlocking previously inaccessible reactivity, is a formidable challenge. With this aim in mind, we turned our attention to 9,10-dihydroacridine scaffolds that are typically used in material chemistry as electron-donor moieties.^10^ Until recently, their use as photocatalysts has been largely underdeveloped, which might be explained by the challenges associated with their synthesis and their tunability. However, we recently solved this issue by devising a simple protocol to access those compounds in a single step from common alkyne and diarylamine precursors.^11^ We herein document the rational design and structural refinements of two new classes of highly reducing purely organic PCs incorporating a 9,10-dihydroacridine scaffold (Figure 1, **4** and **5**). We performed complete physicochemical characterization of various scaffolds, while delineating structure–property relationships. Their synthetic potential is illustrated in thermodynamically challenging reductive photoredox processes. Furthermore, we disclose a new mechanistic pathway based on a catalytic proton-coupled electron transfer (PCET) mechanism that relies on the crucial activity of the free NH group, a key structural signature of these molecules. This reaction manifold is currently not possible to access by the previous PC classes (**1**–**3**) as they lack the essential H-bond donor moiety. Lastly, the use of our catalysts in metal-free controlled radical polymerization processes under visible-light was investigated.

## Results and Discussion

### Photophysical Characterization

**Figure 2:**
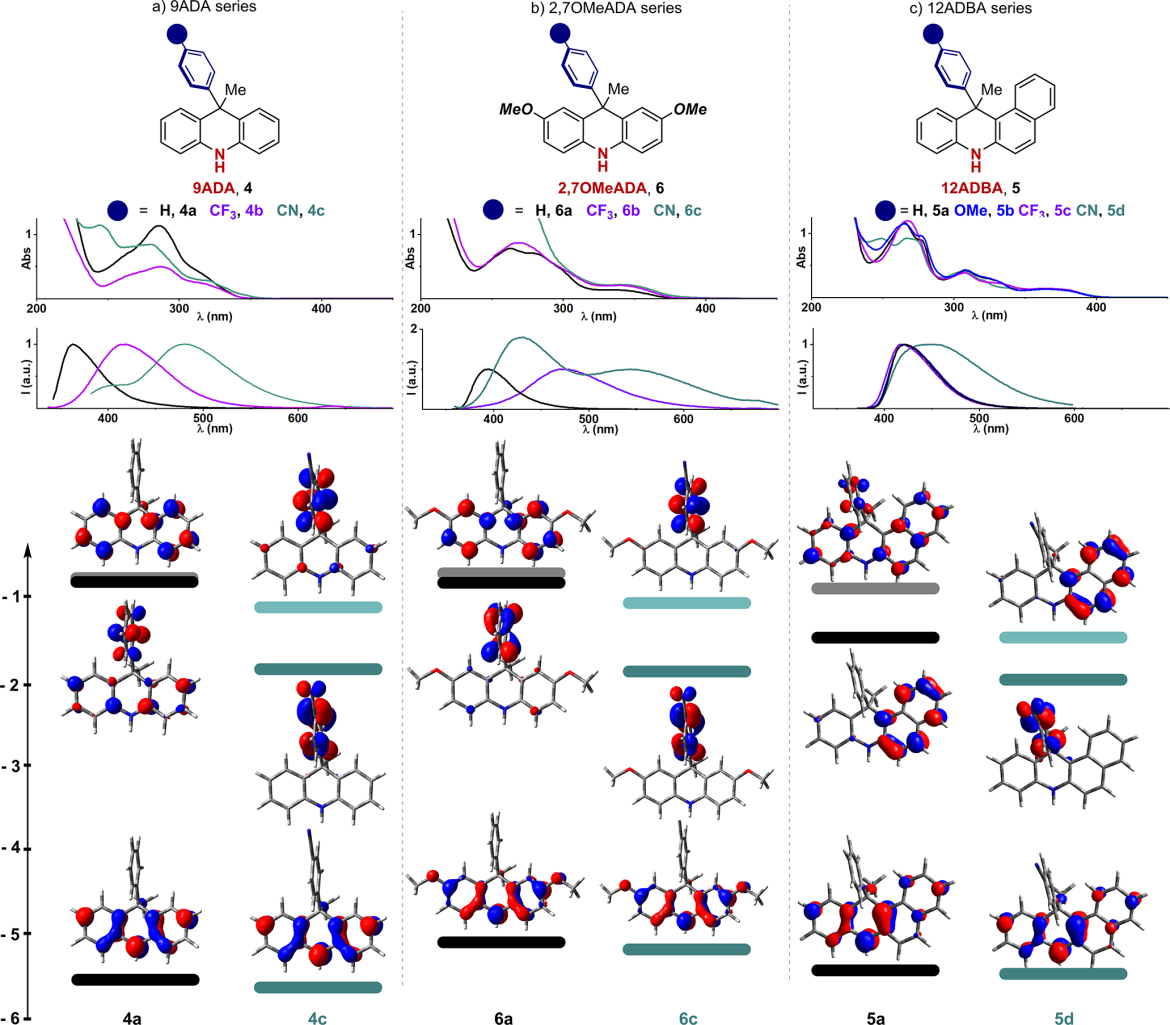
Absorption and emission profiles of (a) 9ADA PCs **4a**–**c**, (b) 2,7-OMeADA PCs **6a**–**c**, and (c) 12ADBA PCs **5a**–**d**. All the experimental data were collected in MeCN (see SI, Section F). Selected frontier MOs and relative calculated energies of PCs bearing an unsubstituted Ph ring at position 9 or 12 (**4a**, **6a**, and **5a**) and PCs substituted with a *p*-CN group (**4c**, **6c**, and **5d**). The LUMO+1 levels are depicted by lighter color bars.

By assessing the previously reported classes of reducing PCs, we questioned whether the presence of an aryl ring on the N atom was essential to reach a CT excited state. Our investigation was guided by the idea that having a free NH group would allow new types of reactivity. We first considered the 9-aryl dihydroacridine (9ADA) scaffold **4a**–**c** bearing electronically varied functional groups (H, CF_3_, CN; Figure 2a), in which the exocyclic aromatic ring could potentially accommodate the LUMO. Indeed, DFT calculations at the B3LYP/6-311++G** level of theory confirmed our hypothesis and revealed the possibility of accessing a CT excited state (Figure 2a, bottom). Time dependent DFT calculations were performed to verify which MOs are actually involved in the formation of the lowest excited state of each PC (see Table S7 in the SI). In the case of compound **4a**, the lowest excited state primarily involves a transition between HOMO and LUMO+1 MOs, which are both located on the tricyclic core, while the LUMO is located on the external aryl ring (Figure 2a, bottom). This MO distribution suggests access to a CT excited state while allowing the exploitation of the free amino group. Based on previous precedents,^4−6,10^ the CF_3_ and CN groups were selected to evaluate the impact of an increased electron-withdrawing (EW) character of the exocyclic aryl ring on the MOs, and later on the overall photoredox properties of the molecules.

**Figure 3:**
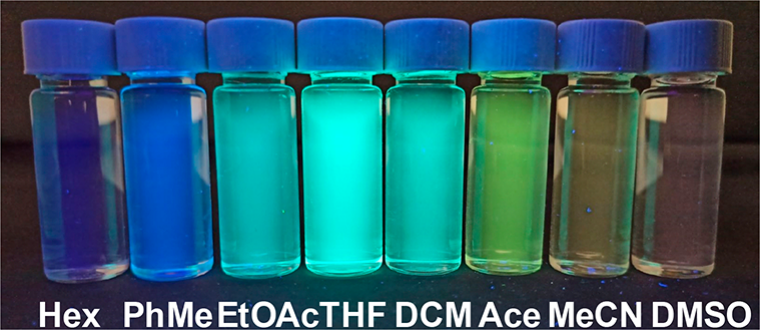
Photograph of the PC **6c** dissolved in solvents with increasing polarity, as indicated by their increasing Reichardt parameter (*E*_T_(30) in kcal/mol). From left to right: *n*-hexane (*E*_T_(30) = 31.0 kcal/mol), toluene (*E*_T_(30) = 33.9 kcal/mol), ethyl acetate (*E*_T_(30) = 38.1 kcal/mol), tetrahydrofuran (*E*_T_(30) = 37.4 kcal/mol), dichloromethane (*E*_T_(30) = 40.7 kcal/mol), acetone (*E*_T_(30) = 42.2 kcal/mol), acetonitrile (*E*_T_(30) = 45.6 kcal/mol), and dimethyl sulfoxide (*E*_T_(30) = 45.1 kcal/mol).

By adding the CN group, we computed a LUMO-lowering and a lowest excited state primarily involving a transition between HOMO and LUMO. These data suggest the CT character of the lowest excited state of **4c**. With respect to the experimental data, it strongly increases the CT character within **4c**. The absorption slightly increases along the series, with **4c** tailing to the visible region (Figure 2a, abs.). On the other hand, the emission profile is significantly red-shifted, with the λ_max_ passing from 362 to 418 and 484 nm (Figure 2a, em.). We next evaluated a HOMO-raising strategy by introducing an electron-donating group (EDG, OMe) at position 2 and 7 of the original scaffold **4** (Figure 2b, bottom). As expected, this class of molecules behaves similarly to **4** while showing a red-shifted absorption, tailing up to 390 nm for **6c**. Additionally, the emission is red-shifted, passing from 362 nm for **6a**, up to 543 nm for **6c**. Over the two series of PCs **4** and **6** (Figure 2a and 2b), the addition of EWGs at the exocyclic aryl group (H to CF_3_ and CN) resulted in a higher Stokes shift, passing from 77 to 204 nm for PCs **4**, and from 24 to 201 nm for PCs **6** (*vide infra*Table 1). This behavior indicates an increased CT excited state character. It is noteworthy that the emission profile of **6c** and in part also the one of **4c** presented two maxima, which can be attributed to two alternative excited configurations: a local S_1_ excited configuration and a S_1_ CT excited configuration.^12^ Indeed, the solvatochromic properties of PC **6c** were experimentally observed by dissolving this PC in solvent having an increased polarity (Figure 3).^13^ These analyses indicate that the photochemical properties of these molecules (**4a**–**c** and **6a**–**c**) can be simply tuned by modifying the substitution pattern of the exocyclic aryl ring. In fact, a mere H-to-CN replacement confers increased visible-light absorption and red-shifted emission, while highly impacting the PC’s lifetime and redox properties (*vide infra*).

**Table 1:**
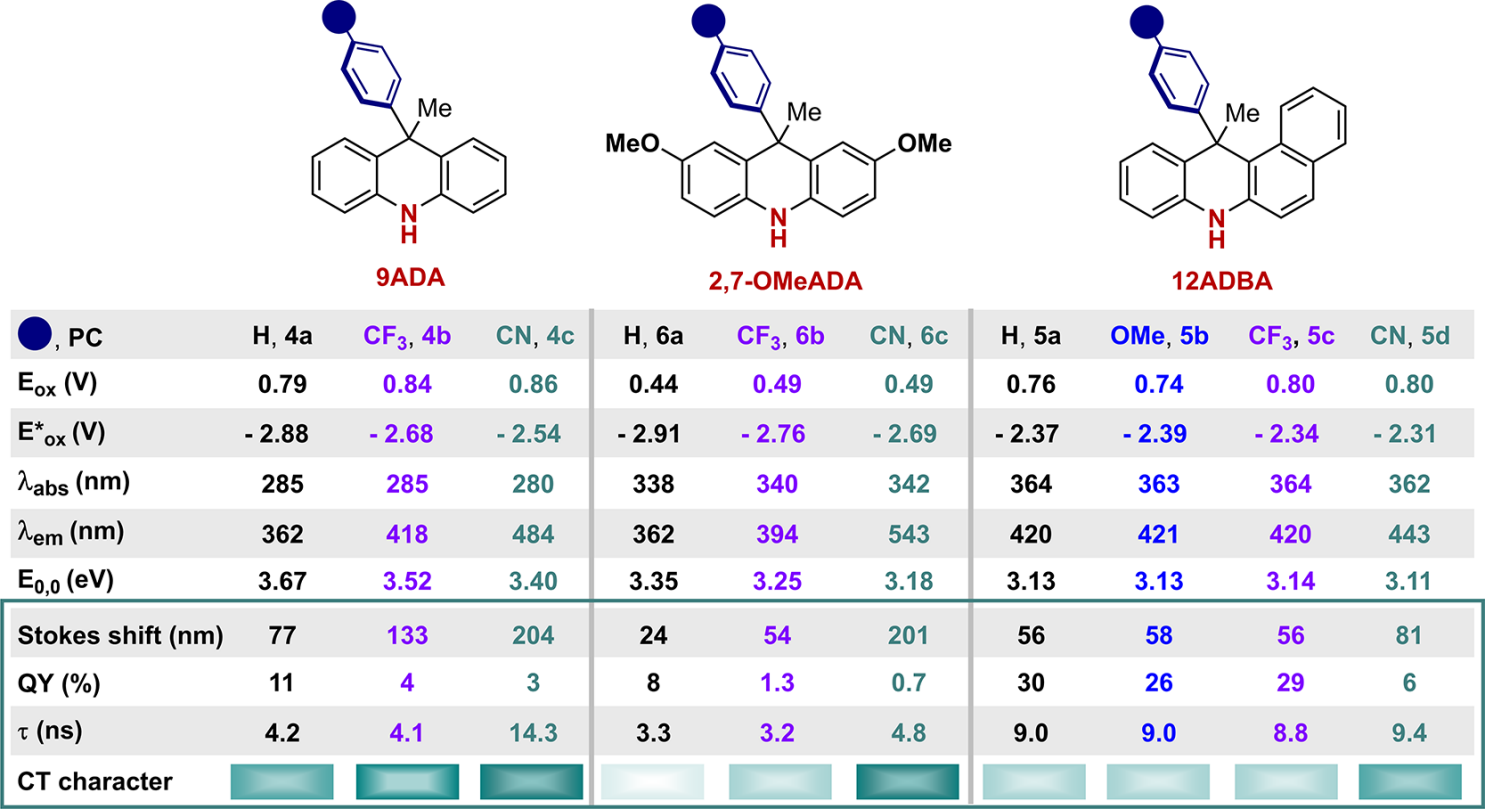
Summary of the Excited- and Ground-State Photoredox Properties^a^

a All potentials were measured in MeCN. Values are reported in V versus SCE (see SI, Sections A and F). The colored bars qualitatively indicate the presence of a CT excited state–the color intensity is based on Stokes shift and QY.

While computationally investigating alternative structural variants and maintaining the free NH group, we considered the 12-aryl dihydrobenzoacridine scaffold (12ADBA) **5**. In the case of this family of PCs, the calculated LUMO is placed at a different position depending on the presence or absence of an EWG on the exocyclic aryl ring (Figure 2c, bottom). In the presence of a H, OMe, or CF_3_ group (**5a**–**5c**), the LUMO lies onto the naphthalene within the PC’s core, whereas, in the case of CN-substituted PC **5d**, it is placed on the exocyclic ring similarly to the other PC’s classes. As a result, a different substitution pattern was used to deliberately alter the LUMO distribution within the scaffolds and thus their photochemical properties. Thanks to the extended conjugation, all PCs **5** have an enhanced visible-light absorption with respect to the other classes of PCs **4** and **6** (Figure 2c, top). The emission profiles are almost identical for PCs **5a**–**c** while an alternative spectroscopic signature is observed for **5d**, as suggested by the alternative LUMO distribution. The simple addition of a CN group also results in a significant variation of the MO’s distribution which has a major impact on the photochemical properties of this molecule.

### Characterization of the PCs Redox Properties

**Figure 4:**
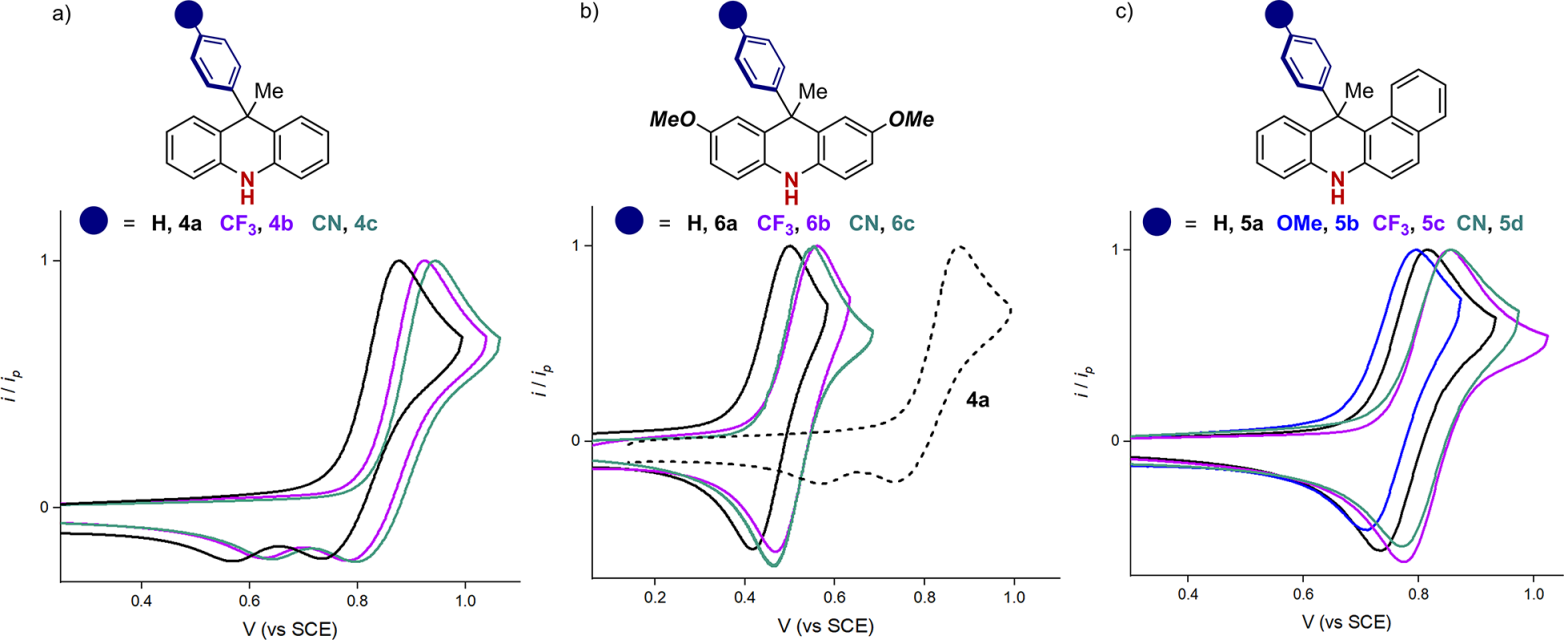
Normalized cyclic voltammograms of PCs **4a**–**c**, **6a**–**c**, and **5a**–**d**. Potentials are reported in V vs SCE. Normalized cyclic voltammograms of PCs **6a**–**c** (solid traces) compared with the normalized cyclic voltammogram of PC **4a** (dashed trace).

We continued the structure–property relationship study by analyzing the cyclic voltammetry (CV) of our PCs to define their ground and excited state redox potentials (Figure 4). As predicted by DFT calculations, the PCs **4a**–**c** showed *E*_ox_ within a narrow range of potentials spanning from 0.79 to 0.86 V vs SCE. However, the cathodic sweeps revealed the presence of two different peaks. Such behavior has been already observed for structurally related systems.

**Figure 5:**
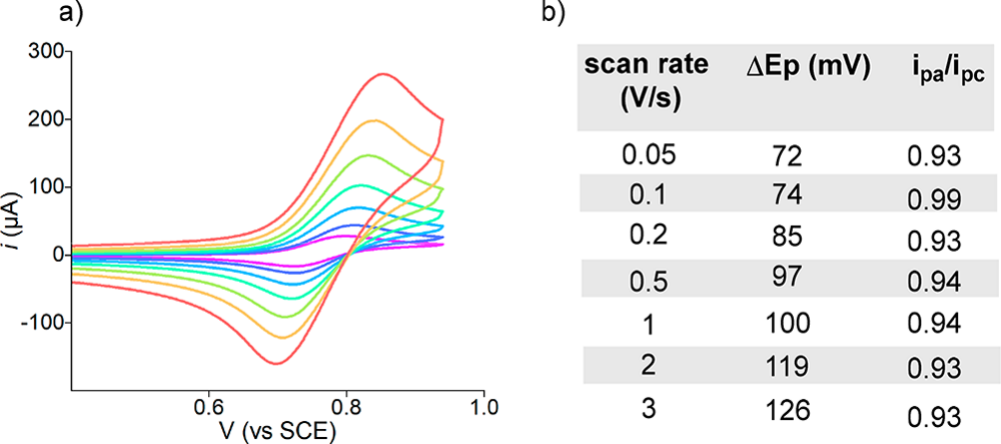
(a) Cyclic voltammograms of **5a** recorded at increasing scan rates. (b) Selected experimental values of Δ*E*_p_ and *i*_pa_/*i*_pc_ at different scan rate (see Section E in the SI).

The second peak was attributed to the presence of electroactive dimerization products in solution deriving from the corresponding radical cations reacting at position 2 and/or 7.^14^ Introducing substituents at such positions inhibited the dimerization pathway. Indeed, the CVs of PCs **5a**–**d** and **6a**–**c** showed a completely reversible behavior with a single cathodic peak. In turn, the more electron-rich PCs **6a**–**c** have less positive *E*_ox_ than both **4a**–**c** and **5a**–**d** (Figure 4), spanning from 0.44 to 0.49 V vs SCE. The presence of OMe groups on the dihydroacridine scaffold results in a HOMO-raising, that facilitates the single electron oxidation by 0.3 V. Although being characterized by a red-shifted absorption, PCs **5a**–**d** showed *E*_ox_ very close to those of **4a**–**c**, spanning from 0.74 to 0.80 V vs SCE. Indeed, insights from the calculated HOMO revealed negligible alteration through the addition of the fused phenyl ring. In the case of PC **5a**, the CV analysis under oxidative scan shows a reversible one-electron wave (Δ*E*_p_ = 74 mV, *i*_pa_/*i*_pc_ = 0.99 at 0.1 V/s scan rate, Figure 5), while the plot of the anodic peak current vs *v*^1/2^ provided a linear relationship, indicating a diffusion-controlled process.^15^

It is important to stress how punctual structural modifications on the scaffolds **4**–**6** can be used to impart a substantial variation of the redox properties while still maintaining a highly reducing character, with the *E*_ox_ spanning from 0.86 to 0.44 for **4c** and **6a**, respectively. This feature is not always possible or easy to access by the currently available highly reducing organic PCs (e.g., **1**–**3**).

We next calculated excited state redox potentials across all the developed PCs. All the E*_ox_ values are highly negative, spanning from −2.31 to −2.91 eV. These data are summarized together with the photochemical and redox properties in Table 1.

### Evaluation of the Excited State Charge Transfer Character

We then completed the assessment of the PCs properties by looking at the ability of the diverse molecules to access a CT excited state. We thus measured the luminescence quantum yield (QY) and the excited state lifetimes (τ). As summarized in Table 1 (green box), the QY decreases over the series of PCs **4** and **6**, passing from 11% to 3% for PCs **4**, and from 8% to 0.7% for PCs **6**, according to the increased CT character. On the other hand, τ increases from 4.2 to 14.3 ns for PCs **4a** → **4c**, and from 3.3 to 4.2 ns for PCs **6a** → **6c**. These trends, together with the highly increased Stokes shift—which exceeds 200 nm for PCs **4c** and **6c**—clearly indicate that PCs **4b**, **4c**, and **6c** can access a CT excited state. A different behavior was observed for the 12ADBA series. Specifically, PCs **5a**–**c** have moderate CT character, with QY and τ remaining to similar levels for these scaffolds. On the other hand, PC **5d** showed an increased Stokes shift of 81 nm, a reduced QY of 6%, and a slightly extended τ of 9.4 nm, indicating a more significant CT character. Remarkably, selecting the suitable substitution pattern allows not only control of the photochemical properties but also access to CT character with longer τ and more balanced redox potentials.

### Exploitation of the NH Group To Accessing PCET Manifolds

Having established structure–property relationships, we next sought to use the free NH group to explore new types of reactivity that were challenging with the previously reported photocatalytic systems. We reasoned that, in the presence of a suitable base associated in a hydrogen-bonding network with the PC, the reduction of the substrate by the PC* could occur through a PCET mechanism, with definite kinetic and thermodynamic benefits.^16^ Apart from the possibility of opening new mechanistic perspectives, a PCET would allow the reduction of redox-inert substrates by extending the redox window of the PC.^16a,16b^ The oxidation potential of the PC* is expressed in eq 1 (where *E*_0,0_ is the energy gap between the lowest vibrational levels of the excited and the ground states) and refers to the semi reaction shown in eq 2.
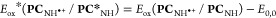
1
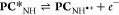
2

In the presence of a base **B** capable of promoting a PCET with the **PC**, the involved semi reaction turns into eq 3, that can be considered as the sum of eqs 2, 4, and 5:
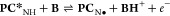
3
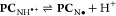
4
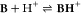
5

The reduction potential from eq 3 can be expressed in eq 6, and it is thus dependent on the potential of the **PC**_NH•+_/**PC**_NH_* couple and on the difference in acidity of **PC**_NH•+_ and **BH**^+^:
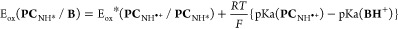
6with  = 0.0257 V.

**Figure 6:**
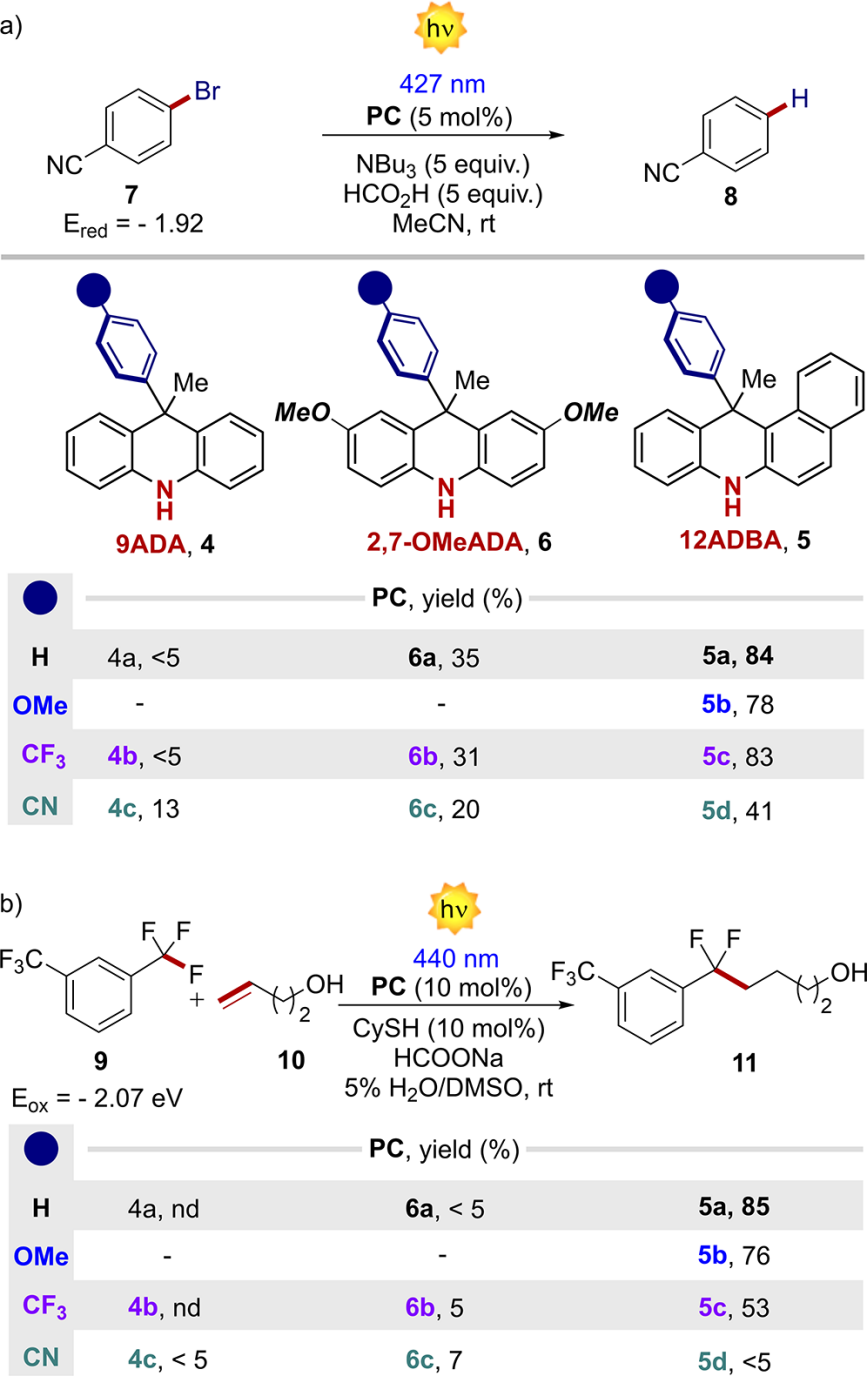
(a) Photocatalytic dehalogenation reaction. (b) Defluorinative arene alkylation. Reaction time 6 h. Yields were determined by GC-FID and ^19^F-NMR (see SI, Section G).

The oxidation potential of the **PC**_NH_*/**B** couple expressed in eq 6 can be in principle adjusted depending on the strength of the base. In particular, the stronger the base (p*K*_a_(**BH**^+^) higher), the more negative the oxidation potential, and the higher the reducing power of the PC*/**B** couple is. With the aim of evaluating the benefits of a PCET vs a classical ET manifold, we started testing the ability of the PCs to engage in reductive dehalogenation processes (Figure 6a). These types of photoreactions require an extremely reducing PC excited state. In fact, they classically proceed either under strong UV-light irradiation,^17^ or by relying on the photoexcitation of specific radical anions.^18^ While in previous reports, PTH **1** was used under UV-light irradiation (<380 nm),^17^ we compared the reaction performance of the designed PCs under visible light (427 nm) using both kinetics and final yields (see Section G of the SI, and Figure 6). The best results were obtained with PCs **5a**-**c** that outperformed all the other 9ADA variants **4** and **6**, as well as PTH **1** – the previous PC of choice for this reaction, that furnished the product in 52% yield (see SI, Section G). Besides, the CT character does not infer any advantage in terms of reactivity. We finally wondered which of the excited forms of the PCs (S_1_ vs T_1_) was engaged under the reductive process. We thus performed the control experiments in the presence of two well-known triplet quenchers. The reaction of **5a** in the presence of O_2_ (air) or diazabicyclo[2.2.2]octane (DABCO) furnished the product **8** in nearly identical yield, 81% and 82%, respectively, ruling out the hypothesis of a T_1_-based reactivity of the PCs.

Of note, apart from previous mechanistic studies,^18^ the activity of a XAT-based manifold under the reported reaction conditions is unlikely due to the highly reducing nature of the PCs and to the presence of a stoichiometric amount of acid. To confirm this finding, we selected a second photoreaction to evaluate the PCs under the ET reaction manifold, namely the photoreduction of trifluoromethylarenes (Figure 6b),^13^ employing 1,3-bistrifluoromethylbenzene **9** (*E*_red_ = −2.07 V vs SCE)^14^ as a model substrate with olefin **10** (for the reported mechanism see Section G of SI). It has been recently reported that only extremely reducing PCs such as PTH **1** could promote this challenging process under energetic light irradiation (380–400 nm).^13,15^ We decided to screen all the PCs in our possession under a less energetic 440 nm irradiation, while comparing their kinetic profiles at the initial stage of the reaction (see Section G of the SI). The analysis of kinetic profiles showed the best performances with PCs **5a**, **5b**, and **5c**. In the case of PCs **5a** and **5b**, the target product **11** was obtained in 85% and 76% yield in 6 h, respectively. As a comparison, PTH **1** reached 87% yield after 24 h under a more energetic irradiation.

**Figure 7:**
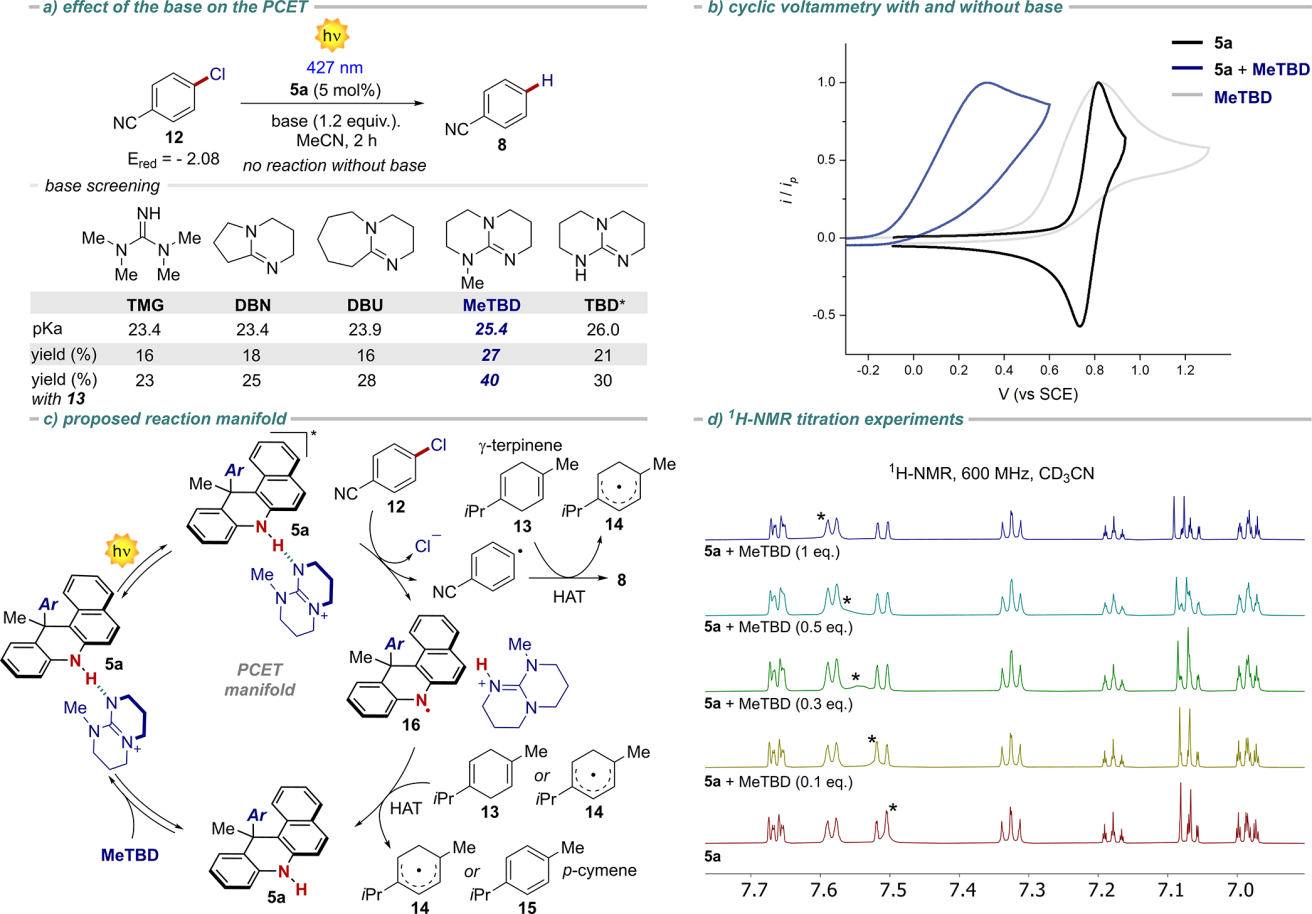
(a) Base screening. (b) Cyclic voltammetry of **5a** in the presence and absence of MeTBD in MeCN, V vs SCE. (c) Proposed reaction manifold. (d) ^1^H NMR titration experiment revealing the presence of a H-bonding complex. Reactions performed at 0.2 mmol scale, [**10**]_0_ = 0.1 M. * TBD resulted partially insoluble under the reaction conditions (see SI, Section H).

Having identified **5a** as the best PC under the classical ET manifold, we subsequently evaluated the feasibility of a PCET mechanism. In this case, we targeted the photoreductive dehalogenation of the thermodynamically challenging aryl chloride **12** (*E*_red_ = −2.08 vs SCE), comparing the reaction outcome in the presence of different bases (p*K*_a_ in the range 23.4–26.0 in MeCN) at a short reaction time (2 h). The reaction in the presence of NBu_3_ proved to be highly inefficient (9% yield), while no reaction was observed without a base. The addition of a base engaging in H-bonding showed improved performances (Figure 7a). MeTBD was identified as the best base, delivering the product in 27% yield. In agreement with the involvement of a PCET mechanism (Figure 7c), a competent HAT donor (e.g., γ-terpinene **13**) was added to the reaction mixture. Under these conditions, the process ended up being faster and more selective. The diverse bases showed a trend with respect to their p*K*_a_. TBD, the only solid base along the series, was the only exception, likely due to its inferior solubility. The best result was obtained when MeTBD was used, providing product **8** in 40% yield after only 2 h with a virtually perfect mass balance. In this case, the control experiments also point to the operation of an S_1_ excited state, as the reactions in the presence of O_2_ or DABCO resulted in 31% and 36% yields, respectively.

**Figure 8:**
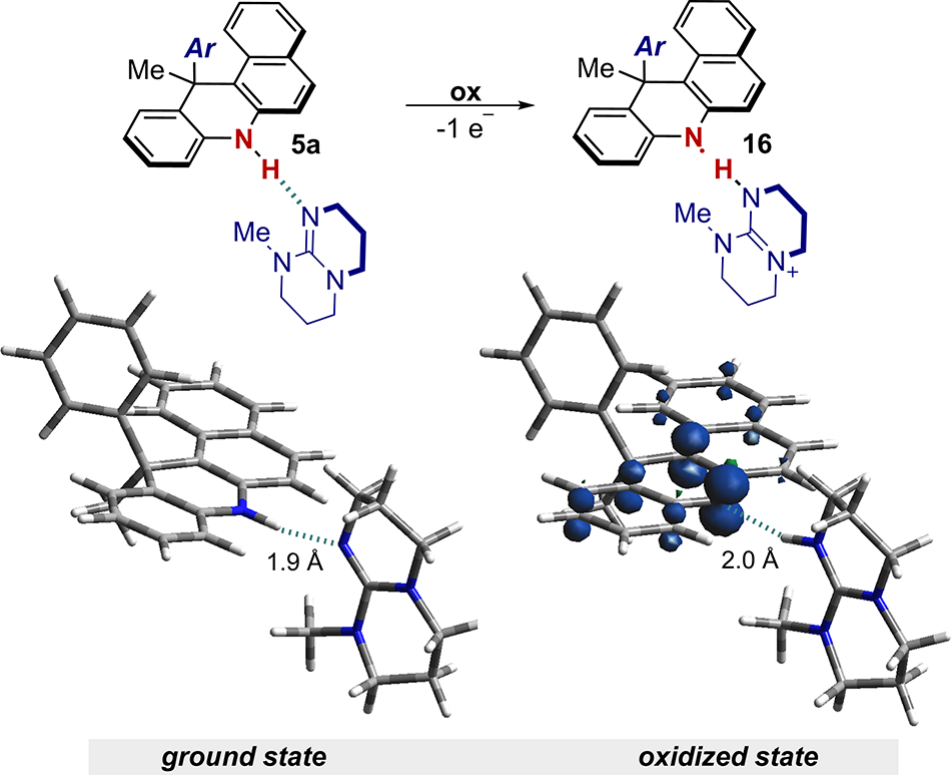
Calculated H-bonding complexes of the PC **5a** with MeTBD at the ground (left), and oxidized state (right) with spin-density map. Structures, energies and electronic features of the complexes were calculated at B3LYP/6-311++G** level of theory (see SI, Section H).

Finally, an extended reaction time with MeTBD (12 h) led to **8** in 65% yield. We next attempted to rationalize the obtained results. Consistent with eq 3, the cyclic voltammetry of **5a** in the presence of MeTBD showed an anodic wave peaking at 0.32 V vs SCE, thus being anticipated by 0.44 V (Figure 7b). This experiment suggests an interaction between the PC and the base, and demonstrates that the oxidation of PC **5a** is anticipated of 0.44 V in the presence of MeTBD. Based on this experimental evidence, we can assume that when the base is added to the reaction mixture, an alternative mechanistic pathway operates, in which the PC is preorganized into a H-bonded complex with MeTBD (Figure 7c). To get insight into the nature of such PC-base interaction, we performed ^1^H NMR titrations. Those experiments, in the presence of increasing amounts of MeTBD, clearly indicate the existence of an H-bonding complex between the base and the PC (Figure 7d). An alternative deprotonation process was ruled out due to the p*K*_a_ of the involved species (MeTBD = 25.4, p*K*_a_**5a** = 32, vide infra), and the different ^1^H NMR trace of the K-salt of **5a** (see SI, Sections D and J). Following this mechanistic proposal (Figure 7c), upon light irradiation, the PC-MeTBD complex reaches an electronically excited state that takes part in a multisite PCET with chlorobenzonitrile **12**. While the electron is transferred to **12**, the proton moves to the MeTBD, resulting in the generation of (*i*) the aryl radical, (*ii*) the protonated MeTBD, and (*iii*) the PC N-centered radical **16**. At this juncture, a HAT donor is necessary to close the catalytic cycle, delivering the final product **8** while restoring the PC. This last step was confirmed by the detection of *p*-cymene (**15**), deriving from the aromatization of γ-terpinene, in the reaction crude by both ^1^H NMR and GC-FID. The quantum yield of the reaction was found to be 0.15, corroborating our mechanistic hypothesis, ruling out at the same time an alternative XAT process (see Section H of the SI). DFT calculations further supported the experimental data, computing a ground-state H-bonding complex of PC **5a** and MeTBD with a NH···N distance of 1.9 Å (Figure 8, left). In agreement with the experimental data, when one electron is removed from the complex, the proton moves to the base with the formation of the N-centered radical **16** (Figure 8, right). The spin-density map reveals that the radical is mainly localized on the N atom. DFT calculations were also performed to estimate the p*K*_a_ values for **5a** and its radical cation **5a**^•**+**^. We used a relative determination method, based on the experimental p*K*_a_ of the base and the difference in energy of the optimized adducts, where the proton is deliberately located on the PC or on the base (see SI, Section H).

**Figure 9:**
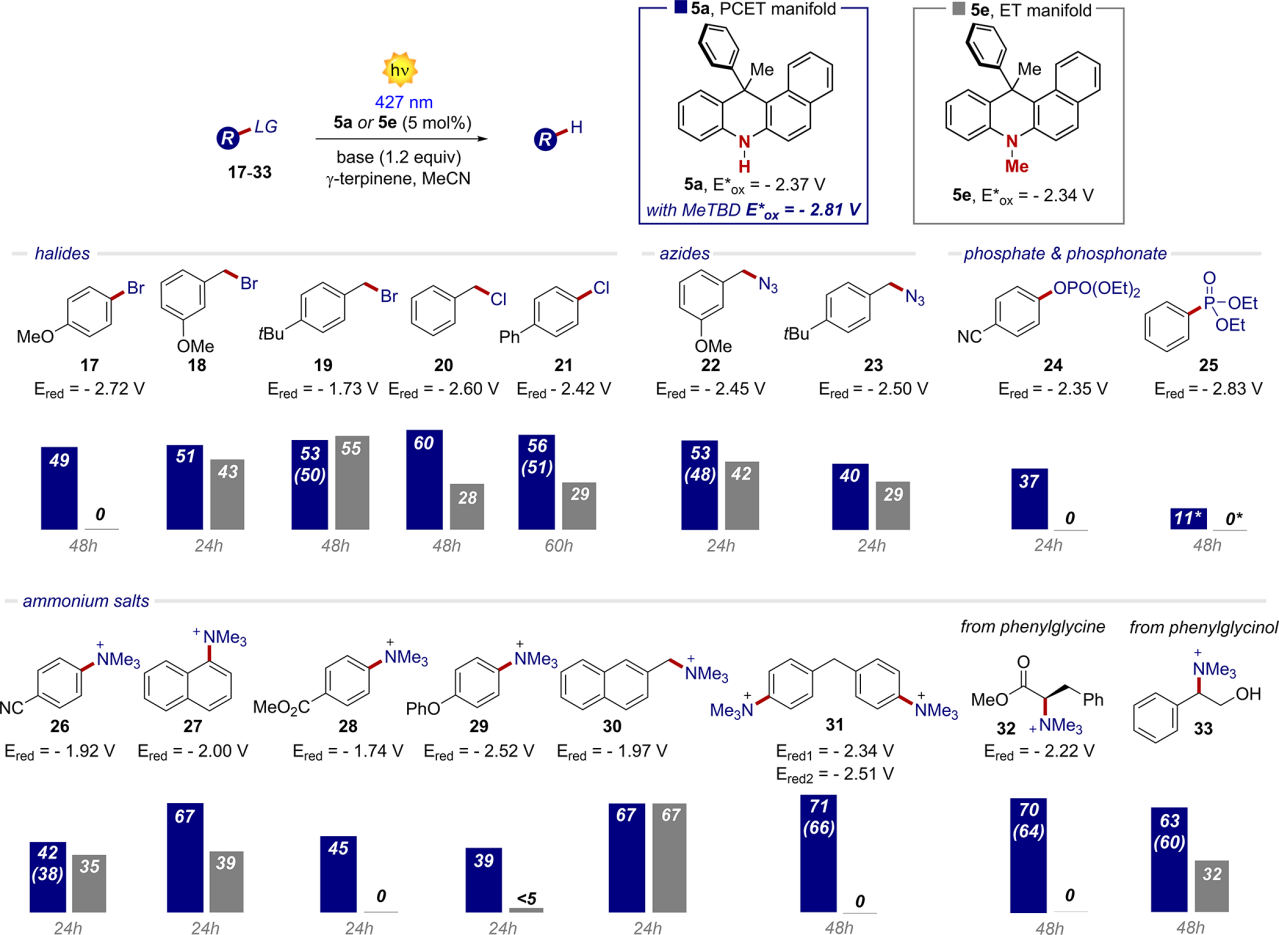
Generality and limits of the PCET manifold (base = MeTBD) and comparison with the classical ET process (base = NBu_3_). Yields determined by GC-FID or ^1^H NMR analysis of the crude reaction mixture; isolated yields are reported in parentheses (see SI, Section G). *The reported value refers to the conversion of the substrate.

This estimation led to a p*K*_a_ of 32 and 17 for **5a** and **5a**^•**+**^, respectively, thus in agreement with a proton shift during the PCET. Having designed an alternative manifold to the classical ET, we then investigated the generality of the PCET manifold with respect to the ET approach for the defunctionalization of structurally diverse substrates. In this regard, we synthesized the methylated PC **5e**, for which a PCET is not possible. From there, we selected a series of compounds **17**-**33** of diverse chemical nature that were all characterized by a very negative E_red_, approaching −2.8 eV vs SCE (in MeCN). Remarkably, without the need of any optimization process, aryl and benzyl halides, as well as azides were found to be suitable substrates, with yields ranging from 40 to 60%. In contrast, under the ET manifold, the corresponding defunctionalized products were obtained, almost in all the cases, in inferior yields (Figure 9). Phosphonates and phosphates were more reluctant substrates, although the PCET manifold delivered the corresponding products in up to 37% yield. Lastly, the ability of defunctionalize alkyl ammonium salts was explored. Here the PCET manifold demonstrated his superior generality and synthetic potential with yields up to 71%. In several cases, under ET, we observed a significant decrease of the efficiency, ammonium salts **28**, **29**, **31** and **32** proving completely unreactive. Remarkably, the PCET manifold also demonstrated its versatility in the presence of difunctionalized substrates (**31**), amino acids derivatives (**32**) and hydroxyl-group-free compounds (**33**). It has to be stressed that the reduction of these molecules (**26**-**33**) usually require photoelectrochemical synthesis or a multiphoton excitation process.^19^ An alternative XAT-based strategy is also not possible.^18^ Here, we just exploited the structural feature of the PCs under a single-photon excitation. While **5a** can still afford the defunctionalized products even beyond its redox potentials (E*_ox_ = −2.37) through a PCET, the *N*-methylated PC **5e**—working by ET—loses the ability to promote photoreductions when approaching its redox potential limit. These results indicate that, by accessing a catalytic PCET manifold, **5a** has key kinetic and thermodynamic benefits.

### PCs for Photoinduced Organocatalyzed Atom Transfer Radical Polymerization (O-ATRP)

**Figure 10:**
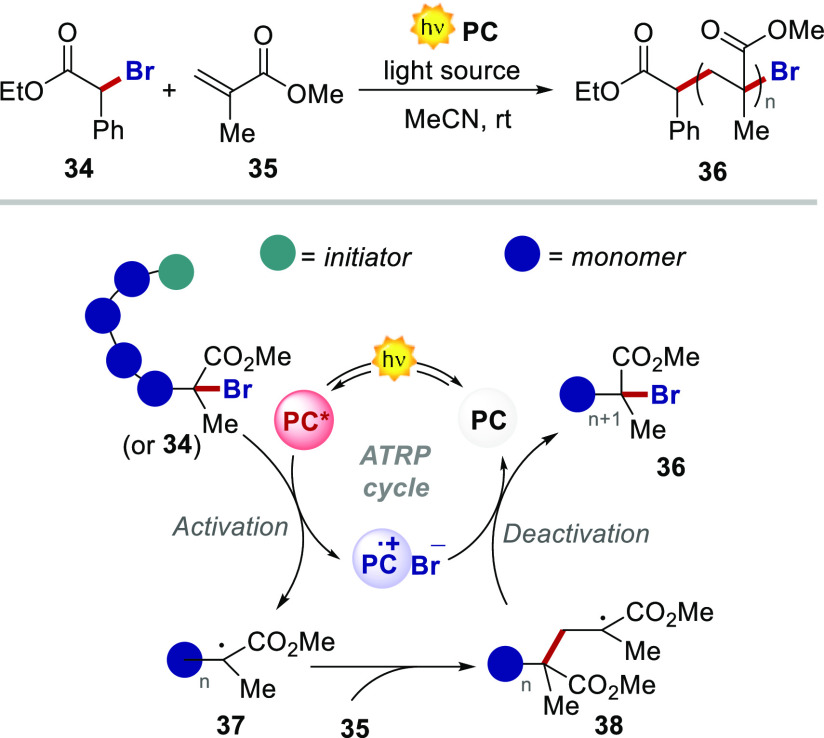
O-ATRP and reported reaction manifold.

As mentioned above, highly reducing PCs have found important applications in organocatalyzed atom transfer radical polymerization (O-ATRP).^9^ We thus decided to assess the ability of our PCs in this type of industrially relevant transformation. As model substrates, we selected ethyl α-bromophenylacetate (BPA) **34** as the initiator and methyl methacrylate (MMA) **35** as the monomer. The efficient use of the developed PCs under ET or PCET manifolds did not guarantee their applicability under ATRP. This is because the reaction manifold in ATRP is significantly different from ET or PCET processes (Figure 10). Upon light excitation, the PC reaches an electronically excited state that is responsible for the activation step by single-electron reduction of the alkyl radical (chain or **28**).

This ET, which requires a high reducing power, leads to the formation of the corresponding radical **37** and the PC^•+^. A delicate interlay should be balanced between the stability of the PC^•+^ and its ability to oxidize the propagating radical **38** or the Br anion, to efficiently deactivate the propagation process and deliver a controlled polymerization. PCs **4**–**6** were subsequently tested as catalysts in photoinduced organocatalyzed atom transfer radical polymerization (O-ATRP) of MMA **35** in acetonitrile (MeCN) as a solvent (Table 2). All the structurally different PCs were able to trigger the polymerization of MMA. An important parameter in the evaluation of the PCs performances in O-ATRP is the molecular weight dispersity of the polymer chains *Đ* (*Đ* = *M*_w_/*M*_n_, where *M*_w_ is the mass-average molecular weight and *M*_n_ is the number-average molecular weight).^9^ Ideally, the *Đ* value should be as close as possible to 1 to obtain a virtually monodispersed polymer. Another relevant parameter is the initiation efficiency (I*) that corresponds to *M*_n_ (theoretical)/*M*_n_ (experimental), where the *M*_n,exp_ is the experimentally measured number-average molecular weight.^9^ In this case the optimal value is 100%, where the PC exerts the highest control over the polymerization process, with a perfect balance on the activation and deactivation steps. PCs **4a**–**c** provided poly(methyl methacrylate) (PMMA) **36** with *Đ* between 1.33 and 1.59 (entries 1–4). In the case of **4c**, the polymerization proceeded more rapidly than with the structurally related **4a**–**b**. This was presumably due to the superior CT character of this particular PC and a corresponding longer excited state lifetime. Of note, while PCs **4a**–**b** displayed moderate to low initiator efficiency (I* = *M*_n,theo_/*M*_n,exp_ × 100%, where *M*_n,theo_ is the theoretical and *M*_n,exp_ the experimentally measured number-average molecular weight),^9^ the compound **4c** exhibited an I* of 113%. Given the red-shifted light absorption of **4c**, we also evaluated its performance as PC for O-ATRP under visible light irradiation at 400 nm (entry 4). Under these conditions, O-ATRP of MMA catalyzed by **4c** showed improved control, reaching *Đ* = 1.33, albeit with a lower I* (70%). This behavior could be attributed to an inferior catalytic activity of the PC, thus exerting lower control on the *M*_W_.^6c^ Since all PCs of the series **6a**–**c** (entries 5–8) featured a red-shifted absorption with respect to **4a**–**c**, they provided relatively high MMA conversion after only 2 h of irradiation (76–86%) with high I*, which approached 100% for **6a** (98%) and **6c** (96%). However, *Đ* remained moderately low, reaching 1.38 for PC **6c** (entry 8).

**Table 2:**
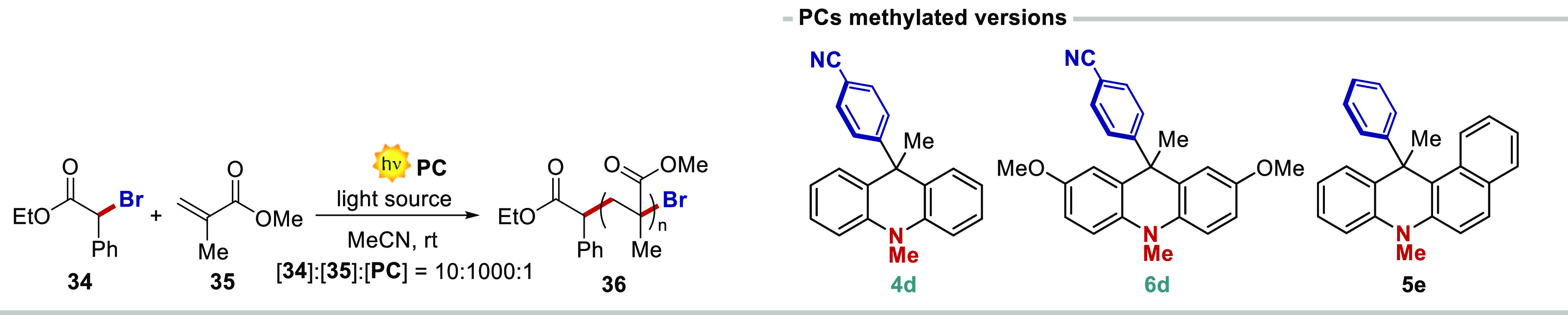
Results of the Polymerization of MMA in Batch^a^

Entry	PC	Wavelength (nm)	Polymerization time (h)	Conversion (%)	*M*_w_ (kDa)	*M*_n_ (kDa)	*Đ*	I* (%)
1	**4a**	390	9	>98	19.8	12.6	1.59	78
2	**4b**	390	7	85	18.7	13.4	1.40	63
3	**4c**	390	2	95	13.1	8.4	1.56	113
4	**4c**	400	4	98	18.5	13.9	1.33	70
5	**6a**	390	2	84	12.1	8.6	1.41	98
6	**6b**	390	2	76	13.8	9.6	1.43	79
7	**6c**	390	2	86	13.2	9.0	1.46	96
8	**6c**	400	4	81	17.4	12.6	1.38	64
9	**5a**	427	6	86	10.8	6.9	1.52	124
10	**5b**	427	6	81	25.8	13.2	1.96	61
11	**5c**	427	6	>98	13.1	8.2	1.57	119
12	**5d**	427	6	>98	12.4	8.0	1.55	122
13	**4d**	400	4	76	19.8	13.1	1.51	58
14	**6d**	400	4	91	16.3	12.2	1.34	74
15	**5e**	427	6	>98	16.2	12.2	1.44	80

a Reaction were performed in MeCN with a ratio [MMA]:[EBP]:[PC] = 1000:10:1, [MMA]:[MeCN] = 1:1 (v/v) (see SI, Section G).

**Figure 11:**
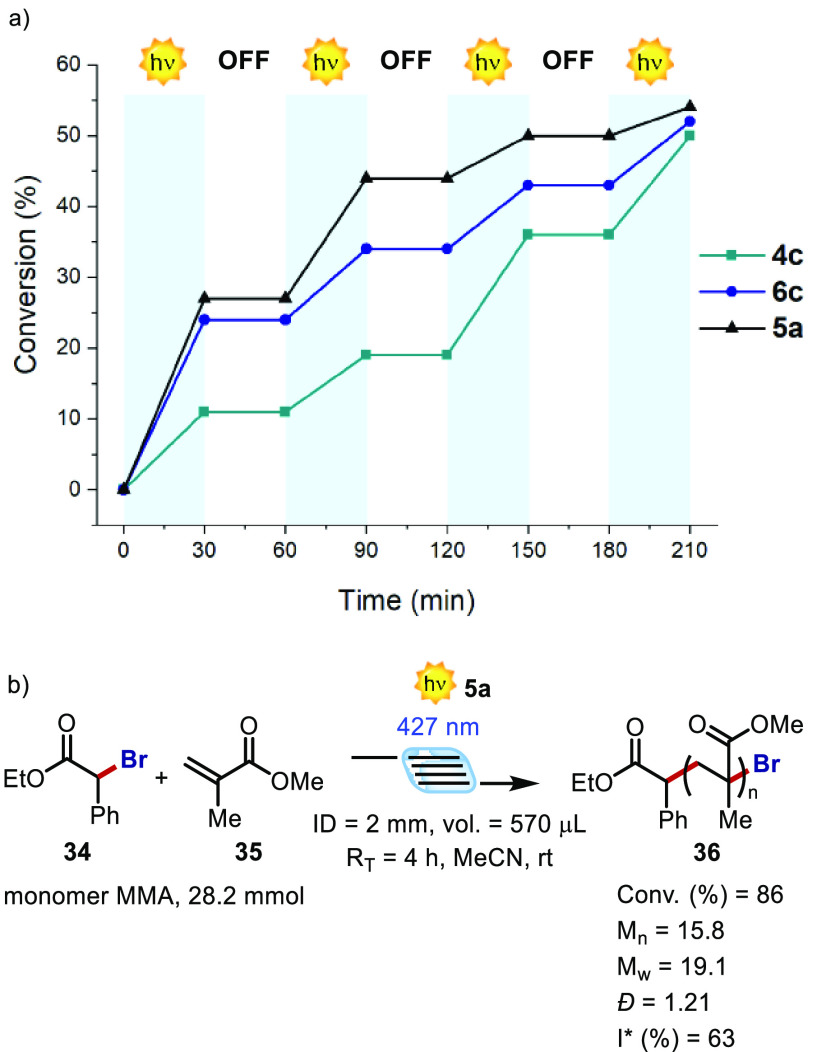
(a) On/off studies performed at 400 nm with PCs **4c**, **6c**, and **5a**. (b) In-flow O-ATRP process (see SI, Section G).

The activity of PCs **5a**–**5d** for O-ATRP of MMA was subsequently evaluated by using 427 nm irradiation. **5c** and **5d** provided full MMA conversion in 6 h, while for **5a** and **5b** the reaction was relatively slower. PCs **5a**, **5c**, and **5d** all provided similar control on *Đ* and exhibited similarly high I*. In turn, **5b** stood out in the series by showing a poor I* (61%) and providing rather polydisperse PMMA (*Đ* = 1.96). Control experiments performed with the *N*-methylated scaffolds of **4c**, **6c**, and **5a** (entries 13–15) resulted in poor control over O-ATRP, highlighting the benefit of the free NH group within the PC structure for the controlled synthesis of PMMA.^20^ In order to test the temporal control over O-ATRP, we performed a series of experiments in which the reaction mixture was exposed to a pulsed irradiation (400 nm, 30 min per cycle). As it can be observed in Figure 11a, using PCs **4c**, **6c**, and **5a**, polymerization occurred only during irradiation, while it reversibly stopped when the reaction solution was kept in the dark. Hence, O-ATRP could be efficiently controlled by switching on and off the light source, enabling excellent control over chain growth by using irradiation as a trigger.^7,21^ Among the PCs, **5a** performed the best, in terms of both initial polymerization rate and conversion over 2 h. The result could be ascribed to a more efficient visible photon harvesting of **5a** with respect to **4c** and **6c** (Figure 2).

Finally, we evaluated the performance of **5a** as PC for O-ATRP under flow conditions (Figure 11b). During a flow process the irradiation is more efficient and homogeneous, permitting a more effective excitation of the PC and activation of the alkyl bromide.^22^ In addition, under these conditions, it is possible to obtain an increased amount of polymer just by parallelization or numbering-up strategy.^23^ In the present study, a simple syringe pump connected to a 3D-printed flow reactor was employed as a flow reactor (see SI, Section G). We scaled-up the polymerization using 28.2 mmol of the monomer. Remarkably, in a relatively short reaction time (*R*_T_) of 4 h, and under visible-light irradiation (427 nm), 86% of MMA conversion with *Đ* = 1.21 was obtained, although a relatively low I* was recorded (see Table 2, entry 9 for comparison). These results further highlight the potential and versatility of the newly developed PCs, and their applicability in O-ATRP, especially when it is performed in continuous flow.

## Conclusions

In conclusion, we have reported free-NH 9-aryl (9ADA) and 12-aryl dihydroacridines (12ADBA), two novel classes of strongly reducing organic PCs engaging under both catalytic PCET and ATRP processes. Based on UV–vis absorption, emission, excited-state lifetime, quantum yield, cyclic voltammetry, and DFT calculations, we have assessed their complete structure–property relationships. The key structural features of these PCs allow access to a CT excited state even in the absence of the common N–Ar moiety, which is present in all the other reported PCs classes (Figure 1). Here, the free NH group is engaging in an H-bonding interaction with a competent base (e.g., MeTBD), expanding the PC’s redox limits to enable the activation of redox-inert alkyl and aryl halides as well as azides, and aryl and alkyl ammonium salts (yields up 71%). The disclosed PCET-based reactivity has been investigated by CV and ^1^H NMR titration along with computational data. Finally, we also evaluated the potential use of these new PCs in catalyzing O-ATRP processes. Across the diverse PC structures, we determined that the 9ADA family was more performant, delivering the polymer under controlled conditions in up to 1.21 *Đ*, and 98% I* (Table 2). Importantly, the PCs accessing a CT excited state (longer τ, and more balanced redox potentials) gave better results under ATRP processes, while the PCs accessing a locally excited state (shorter τ, and higher E*_ox_) performed better for the activation of thermodynamically challenging substrates. Here, we have shown that previously inaccessible mechanistic vistas can be opened by using the free NH group, without losing the ability of catalyzing O-ATRP processes. We thus foresee a broad utilization of these versatile purely organic PCs for the activation of redox-inert substrates under synthetic and material chemistry settings.

## Supporting Information Available

Experimental procedures, characterization data, and NMR spectra (PDF)
