# Supplementary material for: The Rational Design of Reducing Organophotoredox Catalysts Unlocks Proton-Coupled Electron-Transfer and Atom Transfer Radical Polymerization Mechanisms
Source: J Am Chem Soc. 2023 Jan 6;145(3):1835–46. doi: 10.1021/jacs.2c11364 (PMC9881005; doi:10.1021/jacs.2c11364)
Supplement: Supplementary file 1 — ja2c11364_si_001.pdf [file ja2c11364_si_001.pdf]

Supporting Information

The Rational Design of Reducing Organophotoredox Catalysts Unlocks Proton-Coupled Electron-Transfer and Atom Transfer Radical Polymerization Mechanisms

Tommaso Bortolato,[§] Gianluca Simionato,[§] Marie Vayer,[⁋] Cristian Rosso,[§] Lorenzo Paoloni,[‖] Edmondo M. Benetti,[§] Andrea Sartorel,[§] David Leboeuf,[⁋,*] and Luca Dell'Amico[§,*]

[§]Department of Chemical Sciences, University of Padova, Via Marzolo 1, 35131, Padova, Italy.

[⁋]Institut de Science et d'Ingénierie Supramoléculaires (ISIS), CNRS UMR 7006, Université de Strasbourg, 8 allée Gaspard Monge, 67000 Strasbourg, France.

[‖]Dipartimento di Fisica e Astronomia G. Galilei, Via Marzolo 8, 35131, Padova, Italy.

Table of Contents

A. GENERAL INFORMATION 1

B. LIGHT SOURCES EMISSION SPECTRA 3

KESSIL LIGHTS 3

C. EXPERIMENTAL SETUP FOR LIGHT IRRADIATION 4

REACTION SETUP WITH KESSIL LED PR160L 4

D. SYNTHESIS OF THE PHOTOCATALYSTS 5

GENERAL PROCEDURE A 5
9-METHYL-9-PHENYL-9,10-DIHYDROACRIDINE (4A) 5
9-METHYL-9-(4-(TRIFLUOROMETHYL)PHENYL)-9,10-DIHYDROACRIDINE (4B) 5
4-(9-METHYL-9,10-DIHYDROACRIDIN-9-YL)BENZONITRILE (4C) 6
4-(9,10-DIMETHYL-9,10-DIHYDROACRIDIN-9-YL)BENZONITRILE (4D) 6
12-METHYL-12-PHENYL-7,12-DIHYDROBENZO[A]ACRIDINE (5A) 6
12-(4-METHOXYPHENYL)- 12-METHYL-7,12-DIHYDROBENZO[A]ACRIDINE (5B) 6
12-(4-(TRIFLUOROMETHYL)PHENYL)- 12-METHYL-7,12-DIHYDROBENZO[A]ACRIDINE (5C) 7
4-(12-METHYL-7,12-DIHYDROBENZO[A]ACRIDIN-12-YL)BENZONITRILE (5D) 7
7,12-DIMETHYL-12-PHENYL-7,12-DIHYDROBENZO[A]ACRIDINE (5E) 7
12-METHYL-12-PHENYL-7,12-DIHYDROBENZO[A]ACRIDINE POTASSIUM SALT (K-5A) 8
2,7-DIMETHOXY-9-METHYL-9-PHENYL-9,10-DIHYDROACRIDINE (6A) 8
2,7-DIMETHOXY-9-(4-TRIFLUOROMETHYL)-9-METHYL-9,10-DIHYDROACRIDINE (6B) 8
4-(2,7-DIMETHOXY-9-METHYL-9,10-DIHYDROACRIDIN-9-YL)BENZONITRILE (6C) 8
4-(2,7-DIMETHOXY-9,10-DIMETHYL-9,10-DIHYDROACRIDIN-9-YL)BENZONITRILE (6D) 9

E. SYNTHESIS OF THE STARTING MATERIALS 10

4-CHLORO-1,1'-BIPHENYL (21) 10
1-(AZIDOMETHYL)-3-METHOXYBENZENE (22) 10
1-(AZIDOMETHYL)-4-(TERT-BUTYL)BENZENE (23) 10
4-CYANOPHENYL DIETHYL PHOSPHATE (24) 10
DIETHYL PHENYLPHOSPHONATE (25) 10
4-CYANO-*N,N,N*-TRIMETHYLBENZENAMINIUM TRIFLUOROMETHANESULFONATE (26) 10
N,N,N-TRIMETHYLNAPHTHALEN-1-AMINIUM TRIFLUOROMETHANESULFONATE (27) 11
4-(METHOXYCARBONYL)-N,N,N-TRIMETHYLBENZENAMINIUM TRIFLUOROMETHANESULFONATE (28) 11
N,N,N-TRIMETHYL-4-PHENOXYBENZENAMINIUM TRIFLUOROMETHANESULFONATE (29) 11
N,N,N-TRIMETHYL-1-(NAPHTHALEN-2-YL)METHANAMINIUM TRIFLUOROMETHANESULFONATE (30) 11
4,4'-METHYLENEBIS(*N,N,N*-TRIMETHYLBENZENAMINIUM) TRIFLUOROMETHANESULFONATE (31) 11

F. PHOTOREDOX PROPERTIES 13

LUMINESCENCE QUANTUM YIELD MEASUREMENTS 13
REDOX POTENTIAL OF THE EXCITED STATE 13

Photoredox properties summary 13
9-methyl-9-phenyl-9,10-dihydroacridine (4a) 14
9-methyl-9-(4-(trifluoromethyl)phenyl)-9,10-dihydroacridine (4b) 17
4-(9-methyl-9,10-dihydroacridin-9-yl)benzonitrile (4c) 20
4-(9,10-dimethyl-9,10-dihydroacridin-9-yl)benzonitrile (4d) 23
12-methyl-12-phenyl-7,12-dihydrobenzo[a]acridine (5a) 25
12-(4-methoxyphenyl)- 12-methyl-7,12-dihydrobenzo[a]acridine (5b) 28
12-(4-(trifluoromethyl)phenyl)- 12-methyl-7,12-dihydrobenzo[a]acridine (5c) 31
4-(12-methyl-7,12-dihydrobenzo[a]acridin-12-yl)benzonitrile (5d) 34
7,12-dimethyl-12-phenyl-7,12-dihydrobenzo[a]acridine (5e) 37
2,7-dimethoxy-9-methyl-9-phenyl-9,10-dihydroacridine (6a) 39
2,7-dimethoxy-9-(4-trifluoromethyl)-9-methyl-9,10-dihydroacridine (6b) 42
4-(2,7-dimethoxy-9-methyl-9,10-dihydroacridin-9-yl)benzonitrile (6c) 45
4-(2,7-dimethoxy-9,10-dimethyl-9,10-dihydroacridin-9-yl)benzonitrile (6d) 48
1-(azidomethyl)-3-methoxybenzene (22) 50
1-(azidomethyl)-4-(tert-butyl)benzene (23) 50
4-cyanophenyl diethyl phosphate (24) 51
N,N,N-trimethylnaphthalen-1-aminium trifluoromethanesulfonate (27) 51
N,N,N-trimethyl-4-phenoxybenzenaminium trifluoromethanesulfonate (29) 52
N,N,N-trimethyl-1-(naphthalen-2-yl)methanaminium trifluoromethanesulfonate (30) 52
4,4'-methylenebis(N,N,N-trimethylbenzenaminium) trifluoromethanesulfonate (31) 53
(S)-1-methoxy-N,N,N-trimethyl-1-oxo-3-phenylpropan-2-aminium trifluoromethanesulfonate (32) 53
Oxidation reversibility analysis 55
Photoredox properties comparison 57
PCs 4a-d 57
PCs 5a-e 59
PCs 6a-d 61

G. EVALUATION OF THE PHOTOREDOX PERFORMANCES 63

Reductive dehalogenation of *p*-bromobenzonitrile 63
General Procedure B 63
Kinetics 64
Control Experiments 65
Mechanism 65
Catalytic defluoroalkylation of 1,3-bis(trifluoromethyl)benzene 66
General procedure C 66
Kinetics 66
Mechanism 67
Reductive dechlorination of 4-chlorobenzonitrile via PCET - effect of base 68
General procedure D 68
Reductive dechlorination of 4-chlorobenzonitrile via PCET - effect of base and γ-terpinene 69
General procedure E 69
Reduction of thermodynamically demanding substrates via PCET 70
General procedure F 70
Reduction of 17 70
Reduction of 18 70
Reduction of 19 70
Reduction of 20 70
Reduction of 21 71
Reduction of 22 71

REDUCTION OF 23 71
REDUCTION OF 24 71
REDUCTION OF 25 72
REDUCTION OF 26 72
REDUCTION OF 27 72
REDUCTION OF 28 73
REDUCTION OF 29 73
REDUCTION OF 30 73
REDUCTION OF 31 73
REDUCTION OF 32 73
REDUCTION OF 33 74
REDUCTION OF THERMODYNAMICALLY DEMANDING SUBSTRATES VIA ET 74
GENERAL PROCEDURE G 74
REDUCTION OF 17 74
REDUCTION OF 18 74
REDUCTION OF 19 75
REDUCTION OF 20 75
REDUCTION OF 21 75
REDUCTION OF 22 75
REDUCTION OF 23 75
REDUCTION OF 24 76
REDUCTION OF 25 76
REDUCTION OF 26 76
REDUCTION OF 27 77
REDUCTION OF 28 77
REDUCTION OF 29 77
REDUCTION OF 30 77
REDUCTION OF 31 77
REDUCTION OF 32 78
REDUCTION OF 33 78
POLYMERIZATION OF METHYL METHACRYLATE 35 78
GENERAL POLYMERIZATION PROCEDURE IN BATCH 78
ISOLATION OF THE POLYMER 78
ANALYSIS OF MN AND MW 78
POLYMERIZATION KINETIC 78
POLYMERIZATION OF MMA IN FLOW 79
ON-OFF EXPERIMENTS 79

H. MECHANISTIC STUDIES 82

DFT CALCULATIONS 82
OPTIMIZED GEOMETRIES AND SCF ENERGIES 82
ADDITIONAL DATA: HOMO AND LUMO ENERGIES, IONIZATION POTENTIALS AND ELECTRON AFFINITIES **ERROR! BOOKMARK NOT DEFINED.**
ADDUCT BETWEEN THE ELECTRONIC GROUND STATE OF THE NEUTRAL SINGLET 5A AND TMG 102
ADDUCT BETWEEN THE ELECTRONIC GROUND STATE OF THE CATIONIC DOUBLET $5A^{\cdot+}$ AND TMG 105
ADDUCT BETWEEN THE FIRST EXCITED ELECTRONIC STATE OF THE NEUTRAL SINGLET 5A* AND TMG 108
COMMENT 111
ADDUCT BETWEEN THE ELECTRONIC GROUND STATE OF THE NEUTRAL SINGLET 5A AND METBD 112
ADDUCT BETWEEN THE ELECTRONIC GROUND STATE OF THE CATIONIC DOUBLET $5A^{\cdot+}$ AND METBD 115
COMMENT 119

Estimation of the pK_A values of the N-H group in PC 5a 119
UV-Vis absorption of 5a in the presence of MeTBD 121
Fluorescence emission of 5a in the presence of MeTBD 122
NMR Titrations 123
Titration with MeTBD 123
Titration with TMG 124
Cyclic Voltammetry with the presence of a base 128
CV with MeTBD 128
CV with TBD 130
CV with TMG 132
CV with DBU 134
CV with DBN 136
Stern-Volmer quenching of 5a 138
Reaction quantum yield measurements 139
Actinometry 139
Quantum yield of the reduction of 27 140

I. REFERENCES 143

J. NMR SPECTRA 145

A. General Information

All reactions were carried out in anhydrous solvents purchased from commercial suppliers over molecular sieves in a sealed bottle which were used without further purification. Chemicals were purchased from commercial sources (Sigma–Aldrich, Fluorochem or TCI) and used without further purification. Organic solvents were purchased from Sigma–Aldrich. Reactions were monitored by thin-layer chromatography on silica gel 60F254, and/or by ^{19}F NMR spectroscopy.

NMR spectra were collected on a Bruker AC-300 spectrometer fitted with a Bruker PABBO BB/19F-1H/D probe head, Bruker 200 equipped with a QNP probehead, Bruker 400 Avance III HD spectrometer equipped with a BBI-z grad probehead, Bruker 500 Avance III equipped with a BBI-ATM-z grad probehead, Bruker Neo 600 equipped with a Prodigy probehead, Bruker UltraShield 400 or Bruker UltraShield 500 operating at the denoted spectrometer frequency given in MHz for the specified nucleus. Reported coupling constants and chemicals shifts were based on a first order analysis. The internal reference for ^{1}H NMR was the residual peak of $CDCl_3$ (7.26 ppm), CD_2Cl_2 (5.32 ppm) or acetone-*d*$_6$ (2.05 ppm), The internal reference for ^{13}C NMR was the residual peak of $CDCl_3$ (77.16 ppm), CD_2Cl_2 (53.84 ppm) or acetone-*d*$_6$ (29.84 ppm). The reference for ^{19}F NMR was $CFCl_3$ (0.0 ppm). All coupling constants (J) are reported in Hz with the following abbreviations: s = singlet, d = doublet, dd = double doublet, t = triplet, dt = double triplet, q = quadruplet, m = multiplet, br = broad.

Thin-layer chromatography (TLC) analysis was performed on pre-coated Merck TLC plates (silica gel 60G F254, 0.25 mm). Visualization of the developed purification was performed by checking UV absorbance (254 nm) as well as with aqueous ceric ammonium molybdate and potassium permanganate solutions. Chromatographic purification of the products was accomplished using flash chromatography on silica gel (SiO_2, 0.04-0.0063 mm) purchased from Sigma-Aldrich, with the indicated solvent system according to the standard techniques, or with pre-coated Merck preparative TLC plates (silica gel 60G F254, 20x20 cm). Organic solutions were concentrated under reduced pressure on a Büchi rotary evaporator.

Steady-state absorption spectroscopy studies were performed at room temperature on a Varian Cary 50 UV-vis; 10 mm path length Hellma Analytics 100 QS quartz cuvettes were used.

Steady-state emission spectroscopy studies were performed at room temperature on a Varian Cary Eclipse Fluorescence spectrophotometer; 10 mm path length Hellma Analytics 117.100F QS quartz cuvettes were used.

Excited-state lifetimes measurements were performed at room temperature on a FLS1000 UV/Vis/NIR photoluminescence spectrometer (Edinburgh Instruments) at the PanLab facility of the Department of Chemical Sciences, University of Padova, founded by the MIUR-"Dipartimenti di Eccellenza" grant NExuS. The instrument was coupled with a high-speed detector with amplifier, operating in the spectral range: 230-850 nm and with a response time width <180 ps. Excitations were performed with EPLED 280 or EPLED 340 (Edinburgh Instruments), emitting at 286.1 nm and at 341.6 nm, respectively.

The electrochemical characterizations were carried out in acetonitrile (MeCN)/0.1 M tetrabutylammonium hexafluorophosphate ($TBAPF_6$) at room temperature, on an BASi EC Epsilon potentiostat-galvanostat in a glass cell. All the cyclic voltammograms were recorded with a scan rate of 0.1 V/s. A typical three-electrode cell was employed, which was composed of a glassy carbon (GC) working electrode (3 mm diameter), a platinum wire as counter electrode and an Ag/AgCl electrode as reference electrode. The glass electrochemical cell was kept closed with a stopper annexed to the potentiostat. Oxygen was removed by purging the solvent with high-purity Nitrogen (N_2), introduced from a line into the cell by means of a glass pipe. The potential of ferrocenium/ferrocene (Fc^+/Fc)

couple was used as internal reference system to calibrate the potentiostat. All the results were subsequently converted in V vs SCE, in agreement with the value reported in literature [$E_{1/2}(Fc^+/Fc)$ = +0.395 V vs SCE].[1] The IR compensation implemented within the potentiostat was used, and every effort was made throughout the experiments to minimize the resistance of the solution. The full electrochemical reversibility of the voltammetric wave of ferrocene was taken as an indicator of the absence of uncompensated resistance effects. The GC electrode was polished before any measurement with diamond paste, carefully rinsed with de-ionized water, ethanol, acetone and ultrasonically rinsed with a methanol/ethanol/acetone 1:1:1 (v/v) mixture for 5 minutes. After each series of CV experiments, the electrochemical cell was carefully rinsed with ethanol, acetone and de-ionized water; afterwards, the cell and the magnetic stirrer were sonicated for 5-10 min with acetone.

Gas chromatographic (GC) analyses were performed on a Shimadzu GC-2010 Pro gas chromatograph equipped with a flame ionization detector (FID), powered by a current of hydrogen mixed with air. The analyses were performed using an Equity-5 column (15 m, Ø 0.1mm, 0.1 µm film thickness) column and helium as a carrier gas. Every measurement was performed by automatic injection of 1 µL of the sample solution.

High-resolution mass spectrometry (HRMS) analyses were performed on MicroTOF-Q Bruker (ESI) and a GC Thermo Scientific Trace 1300 GC unit coupled to an APPI MasCom source mounted on a Thermo Scientific Exactive Plus EMR mass unit (Orbitrap FT-HRMS analyzer) or on a Xevo G2-XS QToF.

The Kessil lamps PR160L (50W) were purchased from Kessil webpage: www.kessil.com/science/PR160L.php.

B. Light sources emission spectra

Kessil lights

In Figure S1 the emission spectra of Kessil LED PR160L lights are reported. The image can be found in the Kessil website. (More info at: www.kessil.com/science/PR160L.php)

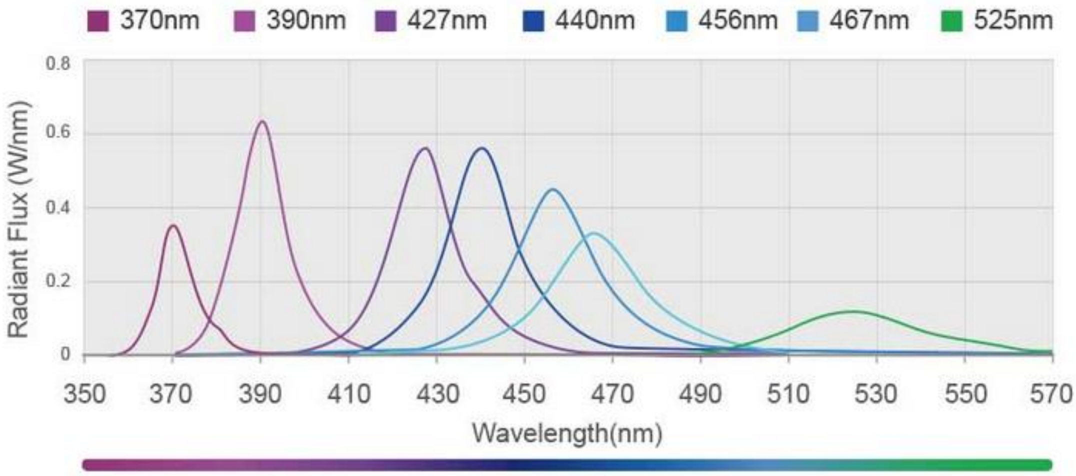

Figure S1: Emission spectra of the Kessil lights used in this work

C. Experimental setup for light irradiation

Reaction setup with Kessil LED PR160L

Figure S2 shows the general setup of a batch reaction performed under Kessil LED PR160L light irradiation (390 nm, 400 nm, 427 nm). The reaction mixture is placed at a fixed distance (about 5 cm) from the light source and stirred vigorously. A maximum of three reaction vessels were irradiated at the same time placing them following the lines of homogeneous irradiance provided by the producer. To maintain a stable reaction temperature one fan was placed above the irradiated vials.

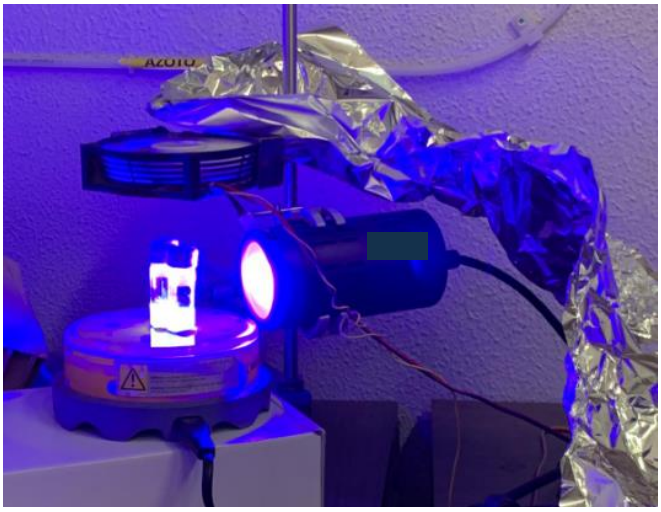

Figure S2: Reaction setup using Kessil lights.

D. Synthesis of the photocatalysts

General Procedure A

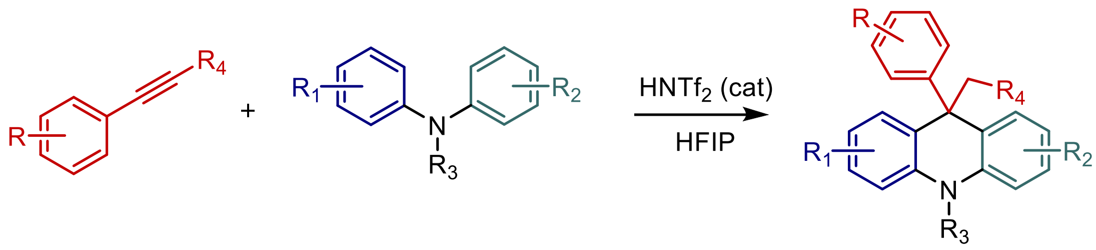

The synthesis follows the reported procedure in literature.[2] Under air, a 10 ml tube equipped with a Teflon-coated magnetic stir bar was charged with arylamine and HFIP. Then, $HNTf_2$ (10 mol%) was added followed by aryl alkyne, and the tube was sealed. Upon completion, the reaction mixture was quenched with a solution of sat. NH_4Cl (30 mL) and then extracted with EtOAc (30 mL x 3). The combined organic layers were washed with brine (30 mL), dried over $MgSO_4$ and concentrated under reduced pressure. The crude mixture was purified by flash column chromatography (FC) over silica gel to furnish the target products.

9-methyl-9-phenyl-9,10-dihydroacridine (4a)

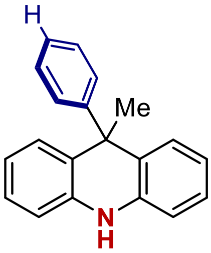

Synthesized following the general procedure starting from diphenylamine (33.8 mg, 0.2 mmol, 1 equiv), phenylacetylene (20.4 mg, 0.2 mmol, 1 equiv) and $HNTf_2$ (2.8 mg, 5 mol%), 53.1 mg of **4a** were obtained (98%) after 16 h at 80°C. Flash column chromatography using Pentane/DCM 7:1 as eluent. **^{1}H-NMR (300 MHz, CD_2Cl_2):** δ 7.20-7.09 (m, 5H), 6.99-6.94 (m, 2H), 6.72-6.62 (m, 6H), 6.22 (br, 1H), 1.75 (s, 3H) ppm. **^{13}C-NMR (60 MHz, CD_2Cl_2):** δ 150.4, 138.4, 129.4, 129.0 (2C), 128.1, 127.2, 126.2, 120.7, 113.7, 45.8, 30.8 ppm. These data match with the previously reported in literature.[2]

9-methyl-9-(4-(trifluoromethyl)phenyl)-9,10-dihydroacridine (4b)

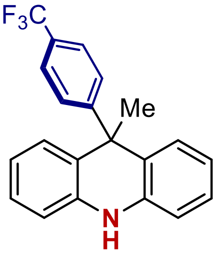

Synthesized following the general procedure starting from diphenylamine (33.8 mg, 0.2 mmol, 1 equiv), 1-ethynyl-4-(trifluoromethyl)benzene (51.0 mg, 0.3 mmol, 1.5 equiv) and $HNTf_2$ (2.8 mg, 5 mol%), 58.3 mg of **4b** were obtained (86%) after 16 h at 80°C. Flash column chromatography using Pentane/DCM 5:1 as eluent. **^{1}H-NMR (360 MHz, CD_2Cl_2):** δ 7.59 (d, J = 7.9 Hz, 2H), 7.49 (d, J = 7.9 Hz, 2H), 7.17-7.12 (m, 2H), 6.84-6.80 (m, 6H), 6.41 (brs, 1H), 1.93 (s, 3H) ppm. **^{13}C-NMR (90 MHz, CD_2Cl_2):** δ 154.1, 138.1, 128.9, 128.4, 128.1, 127.8 (q, J^2 = 32.3 Hz), 127.2, 124.7 (q, J^3 = 4.1 Hz), 124.4 (q, J^1 = 270.4 Hz), 120.5, 113.5, 45.5, 30.2 ppm. **^{19}F-NMR (235 MHz, CD_2Cl_2):** δ -62.4. These data match with the previously reported in literature.[2]

4-(9-methyl-9,10-dihydroacridin-9-yl)benzonitrile (4c)

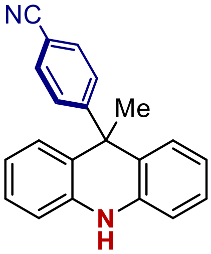

Synthesized following the general procedure starting from diphenylamine (33.8 mg, 0.2 mmol, 1 equiv), 1-ethynylbenzonitrile (38.1 mg, 0.3 mmol, 1.5 equiv) and $HNTf_2$ (2.8 mg, 5 mol%), 45.0 mg of **4c** were obtained (76%) after 72 h at 120°C. Flash column chromatography using Pentane/DCM 4:1 as eluent. **^{1}H-NMR (300 MHz, CD_2Cl_2):** δ 7.61 (d, *J* = 8.4 Hz, 2H), 7.45 (d, *J* = 8.4 Hz, 2H), 7.17-7.19 (m, 2H), 6.83-6.79 (m, 6H), 6.43 (brs, 1H), 1.92 (s, 3H)ppm. **^{13}C-NMR (90 MHz, Acetone-d6):** δ 155.8, 138.7, 131.5, 129.3, 128.2, 127.3, 127.2, 127.0, 118.5, 113.7, 109.5, 45.5, 29.8 ppm. These data match with the previously reported in literature.[2]

4-(9,10-dimethyl-9,10-dihydroacridin-9-yl)benzonitrile (4d)

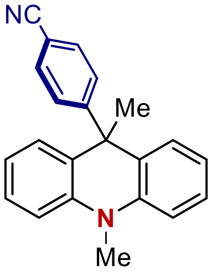

Synthesized following the general procedure starting from *N*-methyldiphenylamine (36.6 mg, 0.2 mmol, 1 equiv), 4-ethynylbenzonitrile (38.1 mg, 0.3 mmol, 1.5 equiv) and $HNTf_2$ (2.8 mg, 5 mol%), 42.2 mg of **4d** were obtained (68%) after 72 h at 120 °C. Flash column chromatography using Pentane/DCM 3:2 as eluent. **^{1}H NMR (300 MHz, CD_2Cl_2):** δ 7.51 (d, *J* = 8.4 Hz, 2H), 7.34-7.28 (m, 2H), 7.19-7.12 (m, 4H), 7.03-6.97 (m, 4H), 3.38 (s, 3H), 1.94 (s, 3H) ppm. **^{13}C NMR (75 MHz, CD_2Cl_2):** δ 154.8, 142.2, 131.4, 130.5, 128.6, 127.3, 126.4, 120.4, 118.8, 112.3, 109.7, 45.9, 33.2, 26.8 ppm. These data match with the previously reported in literature.[2]

12-methyl-12-phenyl-7,12-dihydrobenzo[a]acridine (5a)

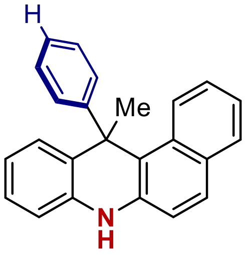

Synthesized following the general procedure starting from *N*-Phenyl-2-naphtylamine (87.6 mg, 0.4 mmol, 2 equiv), phenylacetylene (20.4 mg, 0.2 mmol, 1 equiv) and $HNTf_2$ (2.8 mg, 5 mol%), 39.1 mg of **5a** were obtained (61%) after 16 h at 80°C. Flash column chromatography using Pentane/DCM 3:1 as eluent. **^{1}H-NMR (300 MHz, Acetone-d6):** δ 8.32 (brs, 1H), 7.66-7.60 (m, 4H), 7.54 (d, *J* = 7.8 Hz, 1H), 7.33-7.27 (m, 2H), 7.17-7.11 (m, 2H), 7.06-6.90 (m, 3H), 6.82-6.76 (m, 2H), 6.65-6.60 (m, 1H), 2.24 (s, 3H) ppm. **^{13}C-NMR (75 MHz, Acetone-d6):** δ 152.2, 136.3, 135.9, 132.2, 130.7, 129.8, 129.4, 129.1, 128.9, 128.0 (2C), 126.4, 125.2, 125.2, 125.1, 121.3, 120.0, 117.1, 116.5, 113.1, 45.5, 29.4 ppm. These data match with the previously reported in literature.[2] **5a** was further characterized by ^{1}H and COSY NMR in CD_3CN: **^{1}H NMR (600 MHz, CD_3CN):** δ 7.65 – 7.62 (m, 2H), 7.55 (d, *J* = 7.2 Hz, 2H), 7.51 – 7.46 (m, 2H), 7.31 – 7.27 (m, 2H), 7.15 (tt, *J* = 7.5, 1.2 Hz, 1H), 7.07 – 7.02 (m, 2H), 6.96 (dddt, *J* = 8.5, 4.9, 3.5, 1.5 Hz, 2H), 6.77 (dd, *J* = 7.9, 1.2 Hz, 1H), 6.73 (dd, *J* = 8.0, 1.0 Hz, 1H), 6.64 (ddd, *J* = 8.2, 7.2, 1.3 Hz, 1H), 2.20 (s, 3H) ppm.

12-(4-methoxyphenyl)- 12-methyl-7,12-dihydrobenzo[a]acridine (5b)

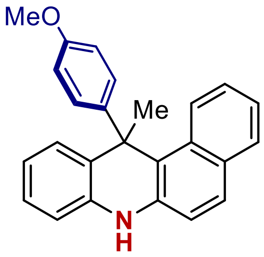

Synthesized following the general procedure with *N*-phenyl-2-naphthylamine (1.31 g, 6.0 mmol, 2.0 equiv.) and 1-ethynyl-4-methoxybenzene (396 mg, 3.0 mmol, 1.0 equiv.) in the presence of $HNTf_2$ (42 mg, 5 mol%) in HFIP (15 mL). The reaction mixture was stirred at 80 °C for 16 h. Purification by FC over silica gel (*n*-pentane/DCM, 100/0 to 50/50) afforded **5b** (135 mg, 0.38 mmol, 13% yield) as a yellow powder. **^{1}H NMR (400 MHz, CD_2Cl_2):** *δ* 7.61 (d, *J* = 8.4 Hz, 2H), 7.54 (d, *J* = 8.3 Hz, 1H), 7.46 (d, *J* = 8.5 Hz, 2H), 7.13 – 6.89 (m, 4H), 6.84 (d, *J* = 9.0 Hz, 2H), 6.79 (d, *J* = 7.8 Hz, 1H), 6.72 – 6.61 (m, 2H), 6.37 (brs, 1H), 3.76 (s, 3H), 2.19 (s, 3H). **^{13}C NMR (100 MHz, CD_2Cl_2):** *δ* 157.7, 144.8, 135.8, 135.6, 132.5, 131.1, 130.4, 130.3, 129.6, 129.3, 129.2 (2C), 126.9, 125.8, 125.8, 122.1, 120.9, 117.6, 117.3, 113.8, 113.3, 55.5, 45.3, 30.3. **HRMS (ESI+):** *m/z* calcd. for $C_{25}H_{21}ON$ $[M]^+$: 351.1623. Found: 351.1619.

12-(4-(trifluoromethyl)phenyl)- 12-methyl-7,12-dihydrobenzo[a]acridine (5c)

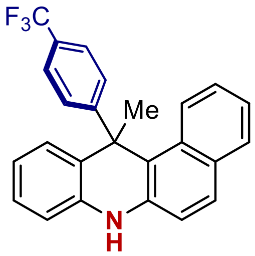

Synthesized following the general procedure with *N*-phenyl-2-naphthylamine (658 mg, 3.0 mmol, 1.0 equiv.) and 4-(trifluoromethyl)phenyl acetylene (765 mg, 4.5 mmol, 1.5 equiv.) in the presence of $HNTf_2$ (42 mg, 5 mol%) in HFIP (15 mL). The reaction mixture was stirred at 80 °C for 16 h. Purification by FC over silica gel (*n*-pentane/DCM, 100/0 to 70/30) afforded **5c** (150 mg, 0.39 mmol, 13% yield) as a yellow powder. **^{1}H NMR (400 MHz, acetone-*d$_6$*):** *δ* 8.41 (brs, 1H), 7.83 (d, *J* = 8.0 Hz, 2H), 7.70 – 7.61 (m, 4H), 7.42 (d, *J* = 8.7 Hz, 1H), 7.16 (d, *J* = 8.8 Hz, 1H), 7.07 – 7.02 (m, 1H), 7.01 – 6.93 (m, 2H), 6.82 – 6.74 (m, 2H), 6.68 – 6.61 (m, 1H), 2.30 (s, 3H). **^{13}C NMR (100 MHz, acetone-*d$_6$*):** *δ* 157.5 (q, *J* = 1.2 Hz), 137.3, 136.8, 132.8, 131.7, 130.7, 130.4, 130.0, 129.6, 129.3, 127.7 (q, *J* = 32.0 Hz), 127.7, 126.4, 125.9 (q, *J* = 3.8 Hz), 125.8, 125.5 (q, *J* = 270.4 Hz), 122.4, 121.1, 118.2, 116.5, 114.4, 46.7, 30.3. **^{19}F NMR (471 MHz, CD_2Cl_2):** *δ* -62.4. **HRMS (ESI+):** *m/z* calcd. for $C_{25}H_{19}F_3N$ $[M+H]^+$: 390.4064. Found: 390.1441.

4-(12-methyl-7,12-dihydrobenzo[a]acridin-12-yl)benzonitrile (5d)

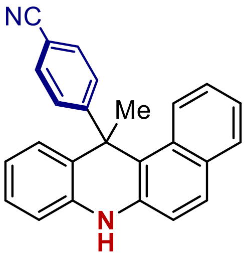

Synthesized following the general procedure starting from *N*-Phenyl-2-naphtylamine (43.8 mg, 0.2 mmol, 1 equiv), 4-ethynylbenzonitrile (38.1 mg, 0.3 mmol, 1.5 equiv) and $HNTf_2$ (2.8 mg, 5 mol%), 47.0 mg of **5d** were obtained (68%) after 72 h at 120°C. Flash column chromatography using Pentane/DCM 2:3 as eluent. **^{1}H-NMR (300 MHz, CD_2Cl_2):** δ 7.76-7.62 (m, 6H), 7.35 (d, *J* = 8.4 Hz, 1H), 7.13-6.98 (m, 4H), 6.76-6.71 (m, 3H), 6.47 (brs, 1H), 2.28 (s, 3H) ppm. **^{13}C-NMR (90 MHz, CD_2Cl_2):** δ 157.3, 135.9, 135.6, 132.5, 132.0, 131.1, 130.2, 130.1, 129.5, 129.1, 128.6, 127.4, 126.2, 125.2, 122.3, 121.2, 119.2, 117.3, 116.2, 113.7, 109.7, 46.4, 29.8 ppm. These data match with the previously reported in literature.[2]

7,12-dimethyl-12-phenyl-7,12-dihydrobenzo[a]acridine (5e)

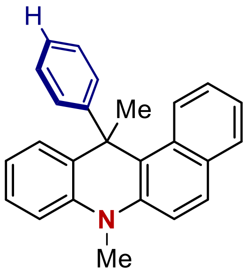

A 25 mL round-bottom flask was charged with a stirring bar and **5a** (100 mg, 0.31 mmol, 1 equiv). The flask was closed with a rubber septum and degassed with a flow of Ar. 5 mL of dry THF were then added via syringe. The flask was cooled to 0°C and NaH (20 mg, 0.47 mmol, 1.5 equiv) was added under stirring. The flask was allowed to warm to room temperature and stirred for 10 minutes. MeI (23 µL, 0.37 mmol, 1.2 equiv) was then added via syringe and the reaction stirred for 16 hours at 40°C. After complete consumption of the starting material, the reaction was quenched by the addition of saturated aqueous $NaHCO_3$ and extracted with EtOAc (3x10 mL). The combined organic layers were then washed with brine, dried over Na_2SO_4 and evaporated under reduced pressure. The residue was purified by flash column chromatography (95:5 hexane:EtOAc) and the product **5e** isolated as a yellow solid in 83% yield (86 mg, 0.26 mmol). **^{1}H NMR (400 MHz, $CDCl_3$):** δ 7.74 (d, *J* = 9.0 Hz, 1H), 7.66 (d, *J* = 8.1 Hz, 1H), 7.49 (t, *J* = 8.7 Hz, 3H), 7.34 – 7.27 (m, 3H), 7.19 (t, *J* = 7.3 Hz, 1H), 7.16 – 7.05 (m, 2H), 6.97 (ddd, *J* = 8.6, 6.7, 1.5 Hz, 1H), 6.90 (d, *J* = 8.2 Hz, 1H), 6.73 (t, *J* = 7.5 Hz, 1H), 6.65 (dd, *J* = 7.9, 1.6 Hz, 1H), 3.58 (s, 3H), 2.13 (s, 3H) ppm. **^{13}C NMR (100 MHz, $CDCl_3$):** δ 151.6, 139.7, 139.3, 133.2, 131.6, 130.1, 129.6, 129.0, 128.8, 128.7, 128.3, 126.8, 126.0, 125.6, 125.2, 122.3, 122.1, 120.4, 115.0, 111.8, 46.2, 35.3, 28.8 ppm. **HRMS (ESI+)** *m/z* calcd. for $C_{25}H_{21}N^+$ $[M]^+$: 335.1674. Found: 335.1673.

12-methyl-12-phenyl-7,12-dihydrobenzo[a]acridine potassium salt (K-5a)

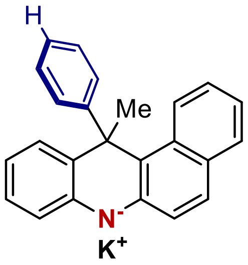

A 4 mL vial was charged with a stirring bar and **5a** (10 mg, 0.031 mmol, 1 equiv). The vial was closed with a cap equipped with a septum and degassed with a flow of Ar. 700 µl of dry CD_3CN were then added and the vial cooled to -23°C with a salt-ice bath. KHMDS (6.8 mg, 0.034 mmol, 1.1 equiv) was then added under stirring. After 10 min, the reaction was allowed to warm to room temperature and stirred for 30 min. The reaction mixture was then transferred via syringe to an Ar-degassed, screw-cap NMR tube equipped with a septum and analyzed immediately by ^{1}H and COSY NMR. **^{1}H NMR (600 MHz, CD_3CN)** δ 7.56 (d, *J* = 7.6 Hz, 4H), 7.45 (d, *J* = 8.0 Hz, 1H), 7.27 (t, *J* = 7.7 Hz, 2H), 7.18 – 7.10 (m, 2H), 7.01 – 6.96 (m, 1H), 6.93 – 6.88 (m, 2H), 6.80 (d, *J* = 7.1 Hz, 1H), 6.73 (d, *J* = 7.1 Hz, 1H), 6.63 – 6.53 (m, 1H), 2.20 (s, 3H) ppm.

2,7-dimethoxy-9-methyl-9-phenyl-9,10-dihydroacridine (6a)

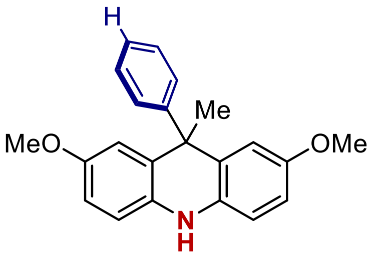

Synthesized following the general procedure with 4,4'-dimethoxydiphenylamine (687 mg, 3.0 mmol, 1.0 equiv.) and phenylacetylene (460 mg, 4.5 mmol, 1.5 equiv.) in the presence of $HNTf_2$ (42 mg, 5 mol%) in HFIP (15 mL). The reaction mixture was stirred at 110 °C for 16 h. Purification by FC over silica gel (*n*-pentane/DCM, 100/0 to 70/30) afforded **6a** (150 mg, 0.45 mmol, 15% yield) as a brown powder. **^{1}H NMR (400 MHz, acetone-*d6*):** *δ* 7.36 – 7.27 (m, 4H), 7.19 (t, *J* = 6.7 Hz, 1H), 6.97 (d, *J* = 7.7 Hz, 1H), 6.82 (d, *J* = 8.6 Hz, 1H), 6.69 (brs, 2H), 6.37 (brs, 2H), 3.67 (brs, 6H), 1.85 (s, 3H). **^{13}C NMR (100 MHz, acetone-*d6*):** *δ* 154.4, 139.0, 129.1, 128.3, 126.4, 119.2, 115.0 (2C), 55.4, 55.3, 30.3, 3C missing. **HRMS (ESI+):** *m/z* calcd. for $C_{22}H_{21}O_2N$ $[M]^+$: 331.1572. Found: 331.1568.

2,7-dimethoxy-9-(4-trifluoromethyl)-9-methyl-9,10-dihydroacridine (6b)

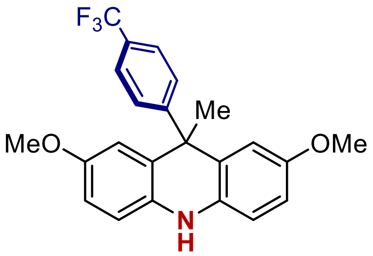

The General Procedure was followed with 4,4'-dimethoxydiphenylamine (687 mg, 3.0 mmol, 1.0 equiv.) and 4-(trifluoromethyl)phenyl acetylene (765 mg, 4.5 mmol, 1.5 equiv.) in the presence of $HNTf_2$ (42 mg, 5 mol%) in HFIP (15 mL). The reaction mixture was stirred at 110 °C for 16 h. Purification by FC over silica gel (*n*-pentane/DCM, 100/0 to 50/50) afforded **6b** (90 mg, 0.23 mmol, 8% yield) as a brown powder. **^{1}H NMR (400 MHz, acetone-*d6*):** *δ* 7.63 (d, *J* = 8.2 Hz, 2H), 7.52 (d, *J* = 8.2 Hz, 2H), 7.00-6.60 (m, 4H), 6.42 (s, 2H), 5.62 (s, 1H), 3.62 (brs, 6H), 1.95 (s, 3H). **^{13}C NMR (100 MHz, CD_2Cl_2):** *δ* 129.2 (2C), 128.4 (q, *J* = 32.2 Hz), 125.3 (d, *J* = 3.0 Hz), 125.2, 124.8 (q, *J* = 271.8 Hz), 55.6, 30.1, 6C missing. **NMR (471 MHz, CD_2Cl_2):** *δ* -62.7. **HRMS (ESI+):** *m/z* calcd. for $C_{23}H_{20}O_2NF_3$ $[M]^+$: 399.1446. Found 399.1443.

4-(2,7-dimethoxy-9-methyl-9,10-dihydroacridin-9-yl)benzonitrile (6c)

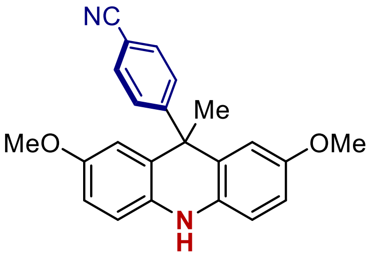

Synthesized following the general procedure starting from bis(4-methoxyphenyl)amine (45.8 mg, 0.2 mmol, 1 equiv), 4-ehtynylbenzonitrile (38.1 mg, 0.3 mmol, 1.5 equiv) and $HNTf_2$ (2.8 mg, 5 mol%), 59.1 mg of **6c** were obtained (95%) after 72 h at 120°C. Flash column chromatography using Pentane/DCM 1:4 as eluent. **^{1}H NMR (300 MHz, CD_2Cl_2):** δ 7.62-7.58 (m, 2H), 7.45- 7.42 (m, 2H), 6.79-6.74 (m, 4H), 6.44-6.41 (m, 2H), 6.27 (brs, 1H), 3.69 (s, 6H), 1.93 (s, 3H) ppm. **^{13}C NMR (75 MHz, CD_2Cl_2):** δ 154.7, 153.9, 133.1, 131.7,

129.1, 127.9, 118.9, 114.2, 113.9, 112.9, 109.8, 55.5, 46.3, 29.1 ppm. These data match with the previously reported in literature.[2]

4-(2,7-dimethoxy-9,10-dimethyl-9,10-dihydroacridin-9-yl)benzonitrile (6d)

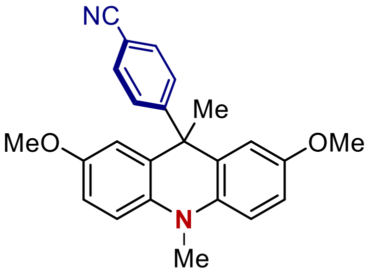

A 25 mL round-bottom flask was charged with a stirring bar and **6c** (40 mg, 0.12 mmol, 1 equiv). The flask was closed with a rubber septum and degassed with a flow of Ar. 1 mL of glacial acetic acid was added, followed by paraformaldehyde (36 mg, 1.2 mmol, 10 equiv). $NaBH_3CN$ (38 mg, 0.6 mmol, 5 equiv) was then added portionwise trying to minimize the vigorous bubbling. The reaction was stirred at room temperature for 16 h. The crude reaction mixture was then cooled to 0°C and quenched by the addition of 5M NaOH until basic pH was reached. The crude was then extracted with EtOAc (2x20 mL) and the organic phase washed with water and brine, dried over Na_2SO_4 and evaporated under reduced pressure. The residue was purified by flash column chromatography (75:25 hexane:EtOAc) and the product **6d** isolated as a yellow solid in 67% yield (30 mg, 0.81 mmol). **^{1}H NMR (300 MHz, $CDCl_3$):** δ 7.47 (d, *J* = 8.1 Hz, 2H), 7.15 (d, *J* = 8.1 Hz, 2H), 6.84 (s, 4H), 6.70 (s, 2H), 3.75 (s, 6H), 3.27 (s, 3H), 1.89 (s, 3H) ppm. **^{13}C NMR (75 MHz, $CDCl_3$):** δ 154.3, 154.0, 137.3, 131.7, 131.6, 128.7, 119.1, 113.6, 112.7, 111.8, 110.0, 55.9, 46.6, 33.6, 26.8 ppm. **HRMS (ESI+)** *m/z* calcd. for $C_{24}H_{22}N_2O_2^+$ $[M]^+$: 370.1681. Found: 370.1717.

E. Synthesis of the starting materials

4-chloro-1,1'-biphenyl (21)

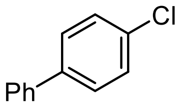

4-chloro-1,1'-biphenyl **21** was synthesized following a reported procedure.[3] The spectral data matched with those reported in the literature.[4] **^{1}H NMR (CDCl$_3$, 400 MHz):** δ 7.62-7.53 (m, 4H), 7.52-7.37 (m, 5H) ppm. **^{13}C NMR (CDCl$_3$, 100 MHz):** δ 140.0, 139.7, 133.4, 128.9, 128.9, 128.4, 127.6, 127.0 ppm.

1-(azidomethyl)-3-methoxybenzene (22)

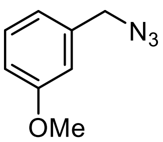

1-(azidomethyl)-3-methoxybenzene **22** was synthesized following a reported procedure.[5] The spectral data matched with those reported in the literature.[5] **^{1}H NMR (CDCl$_3$, 400 MHz):** δ 7.33-7.27 (m, 1H), 6.93-6.84 (m, 3H), 4.32 (s, 2H), 3.83 (s, 3H) ppm. **^{13}C NMR (CDCl$_3$, 100 MHz):** δ 160.1, 137.0, 130.0, 120.5, 114.0, 113.8, 55.4, 54.9 ppm.

1-(azidomethyl)-4-(tert-butyl)benzene (23)

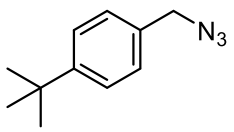

1-(azidomethyl)-4-(tert-butyl)benzene **23** was synthesized following a reported procedure.[6] The spectral data matched with those reported in the literature.[6] **^{1}H NMR (CDCl$_3$, 400 MHz):** δ 7.48 (d, J = 8.4 Hz, 2H), 7.32 (d, J = 8.4 Hz, 2H), 4.36 (s, 2H), 1.40 (s, 9H) ppm. **^{13}C NMR (CDCl$_3$, 100 MHz):** δ 51.2, 132.3, 127.9, 125.7, 54.4, 34.5, 31.2 ppm.

4-cyanophenyl diethyl phosphate (24)

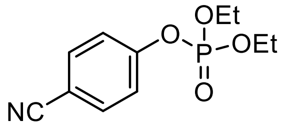

4-cyanophenyl diethyl phosphate **24** was synthesized following a reported procedure.[7] The spectral data matched with those reported in the literature.[7] **^{1}H NMR (400 MHz, CDCl$_3$):** δ 7.60 (dd, J = 8.9, 2.9 Hz, 2H), 7.30–7.26 (m, 2H), 4.23–4.12 (m, 4H), 1.33–1.27 (m, 6H) ppm. **^{13}C NMR (100 MHz, CDCl$_3$):** δ 154.05, 134.04, 120.93, 118.13, 108.73, 65.06, 65.00, 16.03, 15.96 ppm.

Diethyl phenylphosphonate (25)

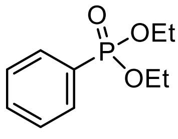

Diethyl phenylphosphonate **25** was synthesized following a reported procedure.[8] The spectral data matched with those reported in the literature.[8] **^{1}H NMR (400 MHz, CDCl$_3$) :** δ 7.74-7.82 (m, 2 H), 7.48-7.55 (m, 1 H), 7.39-7.46 (m, 2 H), 3.99-4.17 (m, 4 H), 1.28 (td, *J* = 7.0, 2.3 Hz, 6H) ppm. **^{13}C NMR (100 MHz, CDCl$_3$):** δ 132.3 (d, *J* = 3.2 Hz), 131.7 (d, *J* = 9.9 Hz), 128.4 (d, *J* = 14.9 Hz), 128.3 (d, *J* = 187.9 Hz), 62.0 (d, *J* = 5.2 Hz), 16.3 (d, *J* = 6.5 Hz) ppm.

4-cyano-*N,N,N*-trimethylbenzenaminium trifluoromethanesulfonate (26)

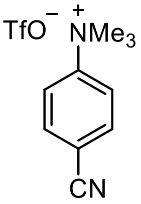

4-cyano-*N,N,N*-trimethylbenzenaminium trifluoromethanesulfonate **26** was synthesized following a reported procedure.[9] The spectral data matched with those reported in the literature.[9] **^{1}H NMR (400 MHz, CDCl$_3$):** δ 8.24-8.14 (m, 4 H), 3.64 (s, 9 H) ppm. **^{13}C NMR (100 MHz, CDCl$_3$):** δ 150.19, 134.15, 133.08, 120.58 (q, JC-F = 322.2 Hz), 122.07, 56.28 ppm. **^{19}F NMR (376 MHz, CDCl$_3$):** δ -77.80 ppm.

N,N,N-trimethylnaphthalen-1-aminium trifluoromethanesulfonate (27)

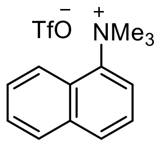

N,N,N-trimethylnaphthalen-1-aminium trifluoromethanesulfonate **27** was synthesized following a reported procedure.[3] The spectral data matched with those reported in the literature.[3] **^{1}H NMR (400 MHz, DMSO-*d*$_6$):** δ 8.63 (d, *J* = 8.8 Hz, 1H), 8.27 – 8.19 (m, 2H), 8.12 (d, *J* = 8.0 Hz, 1H), 7.81 (ddd, *J* = 8.7, 6.9, 1.6 Hz, 1H), 7.75 (ddd, *J* = 7.9, 6.8, 1.0 Hz, 1H), 7.70 (t, *J* = 8.1 Hz, 1H), 3.92 (s, 9H) ppm. **^{13}C NMR (100 MHz, DMSO-*d*$_6$):** δ 144.7, 135.9, 132.8, 131.0, 128.6, 127.2, 125.3, 124.1, 123.7, 121.1 (q, $J_{C\text{-}F}$ = 322.5 Hz), 120.71, 57.52 ppm. **^{19}F NMR (376 MHz, DMSO-*d*$_6$):** δ -77.76 ppm.

4-(methoxycarbonyl)-N,N,N-trimethylbenzenaminium trifluoromethanesulfonate (28)

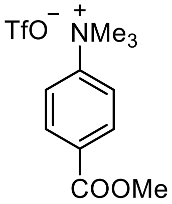

4-(methoxycarbonyl)-*N,N,N*-trimethylbenzenaminium trifluoromethanesulfonate **28** was synthesized following a reported procedure.[3] The spectral data matched with those reported in the literature.[3] **^{1}H NMR (400 MHz, DMSO-*d*$_6$):** δ 8.16 (q, J = 8.6 Hz, 4H), 3.91 (s, 3H), 3.64 (s, 9H) ppm. **^{13}C NMR (100 MHz, DMSO-*d*$_6$):** δ 170.12, 155.72, 136.29, 135.99, 126.58, 61.61, 57.92 ppm.

N,N,N-trimethyl-4-phenoxybenzenaminium trifluoromethanesulfonate (29)

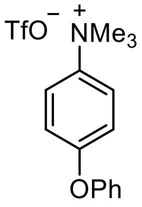

N,N,N-trimethyl-4-phenoxybenzenaminium trifluoromethanesulfonate **29** was synthesized following a modified procedure.[3] To a flask was added 4-phenoxyaniline (2.6 mmol, 560 mg, 1 equiv.) and DCM (4 mL). The solution was cooled to 0°C and methyl trifluoromethanesulfonate (2.9 mmol, 317 µL, 1.1 equiv) was added dropwise. After stirring for 2 hours at room temperature, diethyl ether was added and the obtained precipitate was collected by filtration to yield pure product **29** as a white solid. **^{1}H NMR (500 MHz, CDCl$_3$):** δ 7.79 – 7.71 (m, 2H), 7.40 – 7.32 (m, 2H), 7.21 – 7.15 (m, 1H), 7.10 – 7.04 (m, 2H), 7.03 – 6.98 (m, 2H), 3.67 (s, 9H) ppm. **^{13}C NMR (126 MHz, CDCl$_3$):** δ 159.26, 155.34, 141.18, 130.34, 125.05, 121.50, 120.10, 119.18, 57.63 ppm.

N,N,N-trimethyl-1-(naphthalen-2-yl)methanaminium trifluoromethanesulfonate (30)

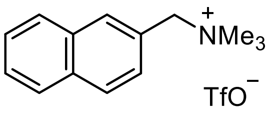

N,N,N-trimethyl-1-(naphthalen-2-yl)methanaminium trifluoromethane sulfonate **30** was synthesized following a reported procedure.[9] The spectral data matched with those reported in the literature.[9] **^{1}H NMR (400 MHz, CDCl$_3$):** δ 7.98 (s, 1H), 7.85 – 7.73 (m, 3H), 7.55 – 7.43 (m, 3H), 4.71 (s, 3H), 3.15 (s, 9H) ppm. **^{13}C NMR (100 MHz, CDCl$_3$):** δ 133.80, 133.51, 132.79, 129.13, 128.60, 128.45, 127.86, 127.66, 127.07, 124.38, 120.70 (q, JC-F = 319.7 Hz), 69.50, 52.46 ppm. **^{19}F NMR (376 MHz, CDCl$_3$):** δ -78.35 ppm.

4,4'-methylenebis(*N,N,N*-trimethylbenzenaminium) trifluoromethanesulfonate (31)

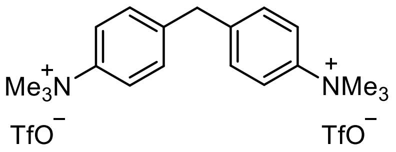

4,4'-methylenebis(*N,N,N*-trimethylbenzenaminium) trifluoromethanesulfonate **31** was synthesized following a reported procedure.[10] The spectral data matched with those reported in the literature.[10] **^{1}H NMR (400 MHz, DMSO-*d*$_6$) :** δ 7.88 (d, J = 8.3 Hz, 4H), 7.55 (d, J = 8.4 Hz, 4H), 4.10 (s, 2H), 3.56 (s, 18H) ppm. **^{13}C NMR (100 MHz, DMSO-*d*$_6$):** δ 150.7, 148.0, 135.3, 125.8, 61.6 ppm.

(S)-2-hydroxy-*N,N,N*-trimethyl-1-phenylethan-1-aminium trifluoromethanesulfonate (33)

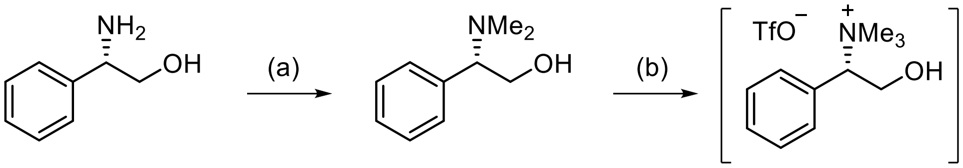

(S)-2-hydroxy-*N,N,N*-trimethyl-1-phenylethan-1-aminium trifluoromethanesulfonate **33** was synthesized from (S)-2-phenylglycinol following a two-steps modified procedure.[3,11] *Step (a)*. To a flask was added (S)-2-phenylglycinol (3 mmol, 410 mg, 1 equiv.), formic acid (12.6 mmol, 475 µL, 4.2 equiv) and formaldehyde solution in water (37% v/v, 6.6 mmol, 500 µL, 2.2 equiv.). The solution was heated up to 100°C and stirred for 2 hours. After cooling down the reaction mixture, 2M NaOH (10 mL) was added. The resulting mixture was extracted with DCM and the organic phase was dried over sodium sulfate. (S)-2-(dimethylamino)-2-phenylethan-1-ol was then obtained by removing the solvent under reduced pressure as a colorless oil (458 mg, 92% yield). The spectral data matched with those reported in the literature.[12] **^{1}H NMR (400 MHz, CDCl$_3$):** δ 7.39 (m, 5 H), 4.72 (dd, J = 3.6,10.5 Hz, 1), 2.31-2.57 (m, 2 H), 2.37 (s, 6 H) ppm. *Step (b)*. (S)-2-hydroxy-*N,N,N*-trimethyl-1-phenylethan-1-aminium trifluoromethanesulfonate (**33**) was then synthesized following a modified procedure.[3] To a flask was added (S)-2-(dimethylamino)-2-phenylethan-1-ol (2.8 mmol, 460 mg, 1 equiv.) and DCM (4 mL). The solution was cooled to 0°C and methyl trifluoromethanesulfonate (3.0 mmol, 340 µL, 1.1 equiv) was added dropwise. After stirring for 2 hours at room temperature, diethyl ether was added, and the obtained biphasic mixture was separated by decantation by removing the supernatant. This procedure was repeated three times. The desired product (**33**) was collected as a colorless oil and used without further purifications due to the difficulties in crystallizing the pure compound. Diagnostic signals at **^{1}H NMR (400 MHz, CDCl$_3$):** 4.81 (dd, J = 7.3, 3.5 Hz, 1H), 4.51 – 4.44 (m, 1H), 4.33 – 4.23 (m, 1H) ppm.

F. Photoredox properties

Luminescence quantum yield measurements

The luminescence quantum yield measurements were performed with quinine sulphate in 0.105 M $HClO_4$ as the standard following a literature procedure.[13] The fluorescence quantum yields were calculated according to equation:

$$\Phi_x = \Phi_{st} \times \frac{I_x}{I_{st}} \times \frac{f_{st}}{f_x} \times \frac{\eta_x^2}{\eta_{st}^2}$$

I is the measured integrated fluorescence emission intensity, f is the absorption factor, η is the refractive index of the solvent and Φ is the quantum yield. The index *x* denotes the sample and the index *st* denotes the standard.

Redox potential of the excited state

The oxidation potential of the excited state photocatalysts $E_{ox}^{*}$was estimated by means of the Rehm-Weller formula:[14]

$$E_{ox}^{*} = E_{ox} - E_{0,0}$$

the oxidation potential E_{ox} was determined by cyclic voltammetry measurements and the excitation energy $E_{0,0}$ was determined from the intersection point between the absorption and the emission profiles.[15]

Photoredox properties summary

PC	λ_{max}abs (nm)	λ_{max}em (nm)	$E_{0,0}$ (S_1)	QY %	τ (ns)	E_{ox} ($PC^{\bullet+}$/PC)	E_{ox}^{*} ($PC^{\bullet+}$/PC*)
**4a**	285	362	3.67	11	4.2	0.79	-2.88
**4b**	285	418	3.52	4	4.1	0.84	-2.68
**4c**	280	484	3.40	3	14.3	0.86	-2.54
**4d**	285	475	3.54	n.d.	n.d.	0.92	-2.62
**5a**	364	420	3.13	30	9	0.76	-2.37
**5b**	363	421	3.13	26	9	0.74	-2.39
**5c**	364	420	3.14	29	8.8	0.80	-2.34
**5d**	362	443	3.11	6	9.4	0.80	-2.31
**5e**	365	425	3.12	n.d.	n.d.	0.79	-2.33
**6a**	338	362	3.35	8	3.3	0.44	-2.91
**6b**	340	394	3.25	1.3	3.2	0.49	-2.76
**6c**	342	543	3.18	0.7	4.8	0.49	-2.69
**6d**	340	543	3.26	n.d.	n.d.	0.58	-2.68

Table S1: All potentials in V vs SCE. Measurements were performed in MeCN.

9-methyl-9-phenyl-9,10-dihydroacridine (4a)

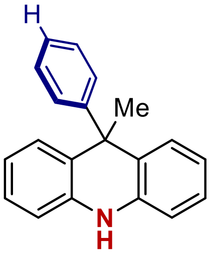

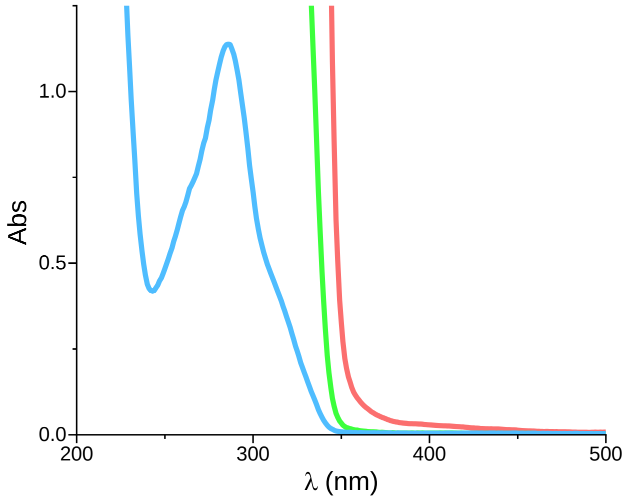

	Concentration (mmol/mL)
	$5 \cdot 10^{-3}$
	$5 \cdot 10^{-4}$
	$5 \cdot 10^{-5}$
λ_{peak}	285 nm
$\lambda_{shoulder}$	320 nm

*Figure S3: UV-Vis absorption of **4a** at different concentrations. 5·10^{-3} M (red trace); 5·10^{-4} M(green trace); 5·10^{-5} M (blue trace). Recorded in MeCN in quartz cuvettes (1 cm optical path).*

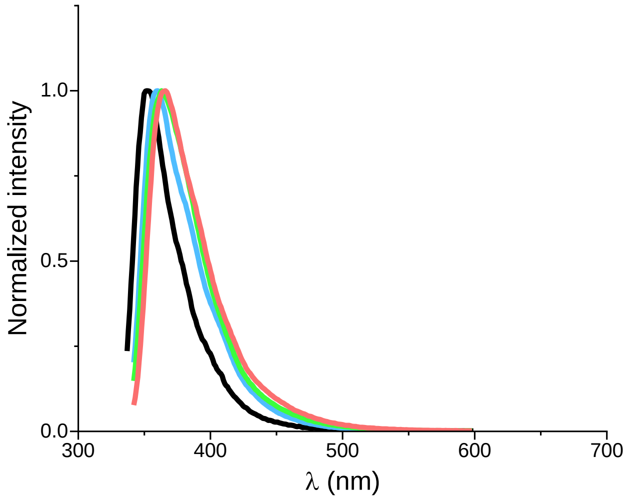

$\lambda_{excitation}$ = 285nm

	Solvent	Peak (nm)
	Toluene	352
	THF	360
	MeCN	362
	DMF	365

*Figure S4: Normalized fluorescence emission of **4a** 2·10^{-5} M in different solvents. Toluene (black trace); THF (blue trace); MeCN (green trace); DMF (red trace).*

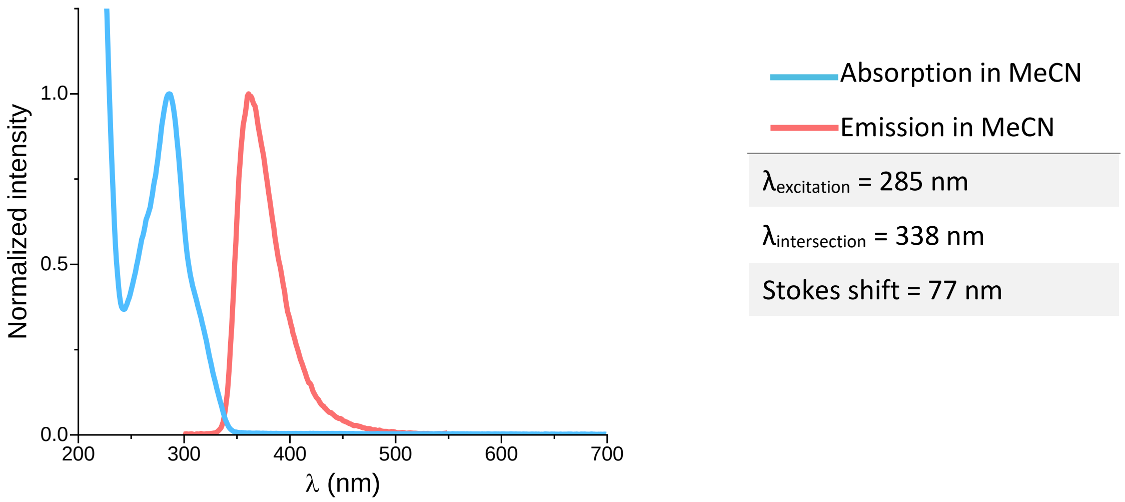

*Figure S5: Normalized optical absorption spectrum (blue trace) and emission spectra (red trace) of **4a***

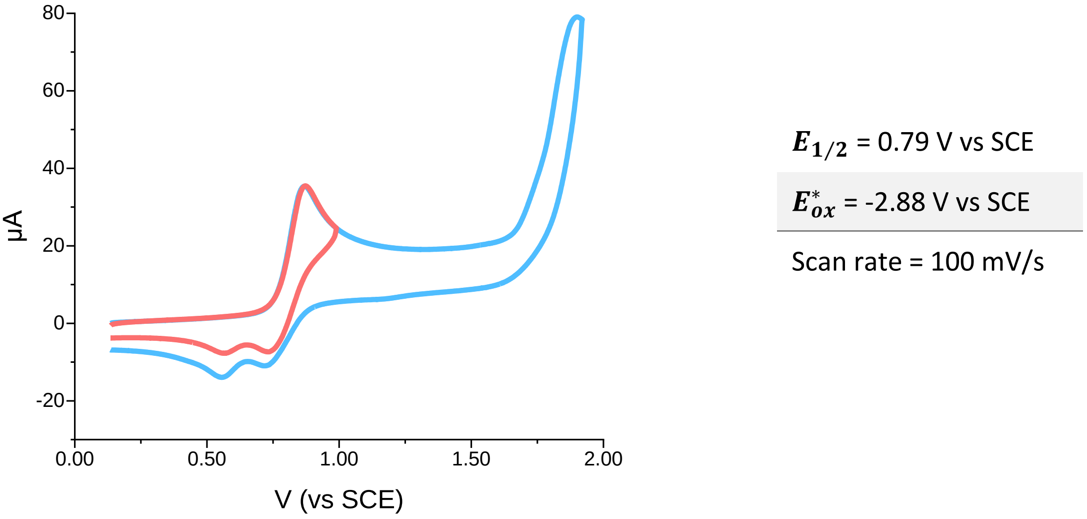

*Figure S6: Cyclic voltammograms of **4a** 10^{-3} M. Recorded with GC as the working electrode in MeCN with 0.1 M $[Bu_4N][PF_6]$ as the supporting electrolyte at 0.1 V/s scan rate*

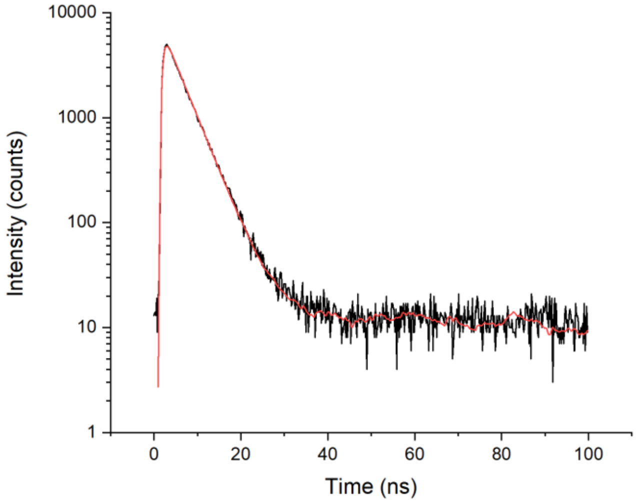

$\lambda_{excitation}$ = 286.1 nm

$\lambda_{analysis}$ = 354 nm

τ = 4.2 ns

Figure S7: Time-resolved emission decay (excitation at 286.1 nm, analysis at 354 nm) of ***4a*** *$2{\cdot}10^{-5}$ M in MeCN solution measured by TC-SPC (τ = 4.2 ns, from deconvolution and single-exponential fitting, X^2 = 1.398).*

9-methyl-9-(4-(trifluoromethyl)phenyl)-9,10-dihydroacridine (4b)

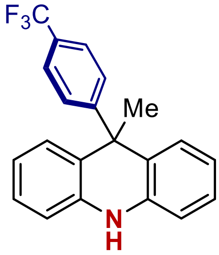

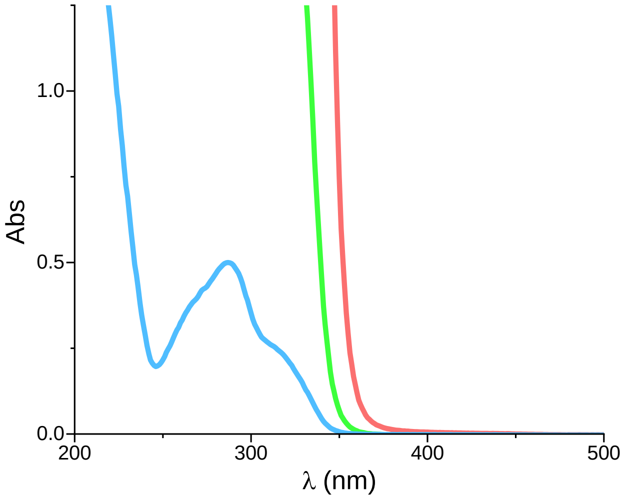

Concentration (mmol/mL)

red	5·10
green	$5\cdot10^{-4}$
blue	$5\cdot10^{-5}$
λ_{peak}	285 nm
$\lambda_{shoulder}$	320 nm

*Figure S8: UV-Vis absorption of **4b** at different concentrations. $5\cdot10^{-3}$ M (red trace); $5\cdot10^{-4}$ M (green trace); $5\cdot10^{-5}$ M (blue trace). Recorded in MeCN in quartz cuvettes (1 cm optical path).*

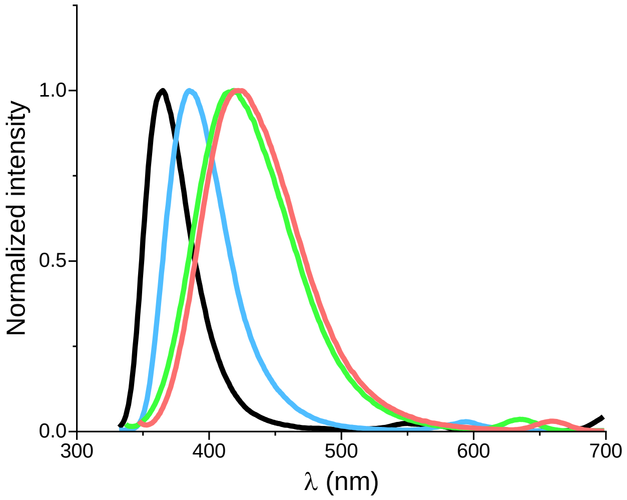

$\lambda_{excitation}$ = 285nm

	Solvent	Peak (nm)
black	Toluene	365
blue	THF	386
green	MeCN	418
red	DMF	423

*Figure S9: Normalized fluorescence emission of **4b** $2\cdot10^{-5}$ M in different solvents. Toluene (black trace); THF (blue trace); MeCN (green trace); DMF (red trace).*

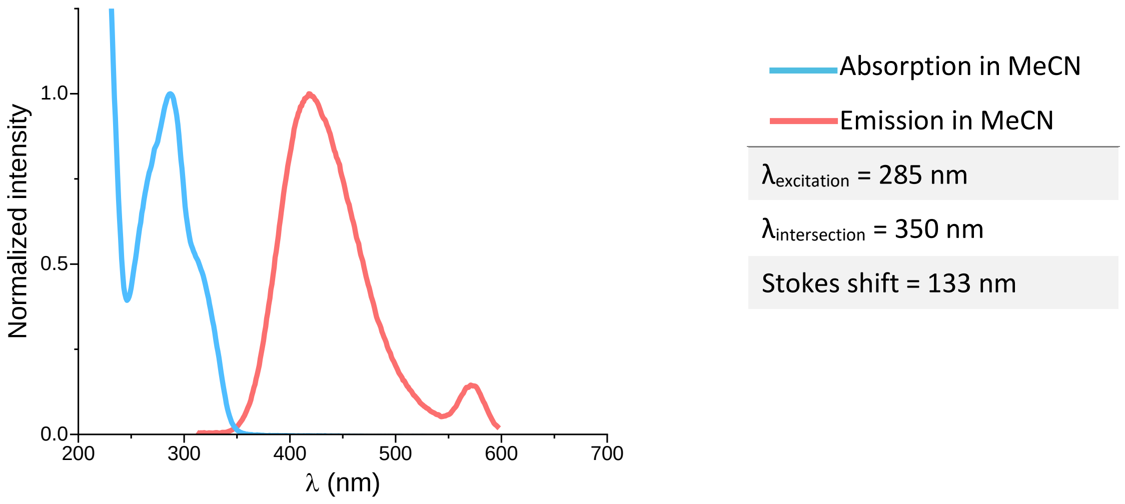

*Figure S10: Normalized optical absorption spectrum (blue trace) and emission spectra (red trace) of **4b**.*

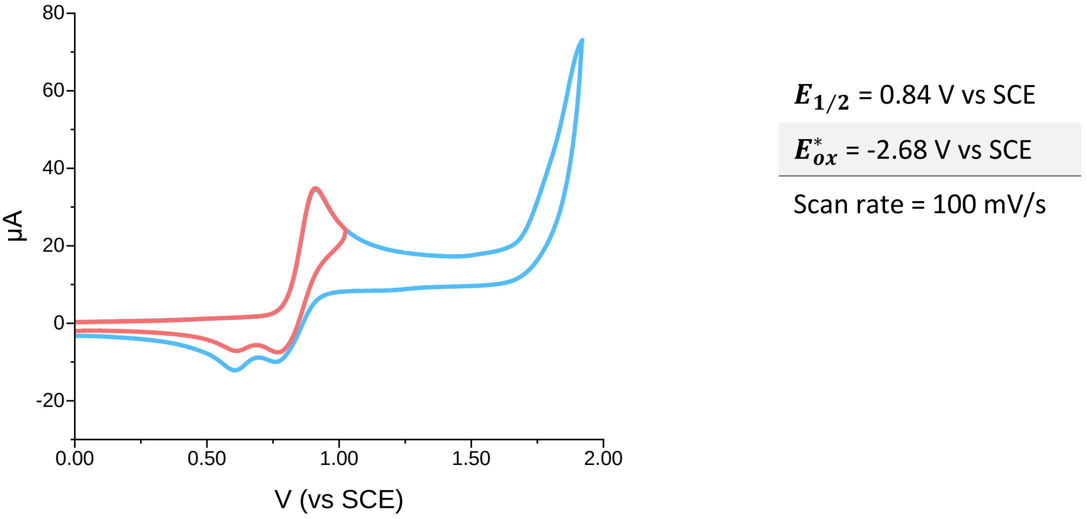

*Figure S11: Cyclic voltammograms of **4b** 10^{-3} M. Recorded with GC as the working electrode in MeCN with 0.1 M $[Bu_4N][PF_6]$ as the supporting electrolyte at 0.1 V/s scan rate.*

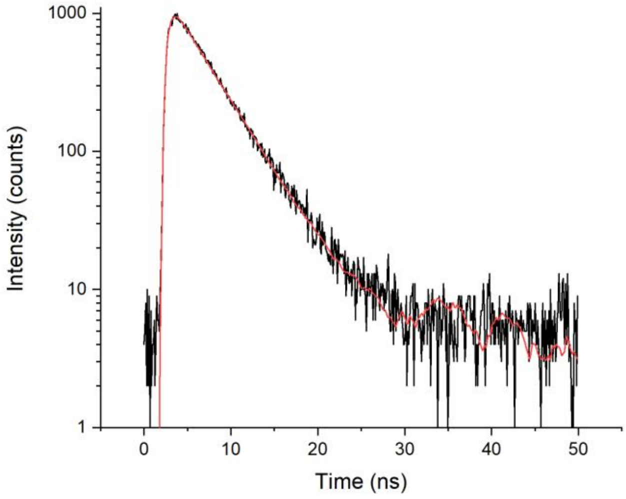

$\lambda_{excitation}$ = 286.1 nm

$\lambda_{analysis}$ = 420 nm

τ = 4.1 ns

*Figure S12: Time-resolved emission decay (excitation at 286.1 nm, analysis at 420 nm) of **4b** $2{\cdot}10^{-5}$ M in MeCN solution measured by TC-SPC (τ = 4.1 ns, from deconvolution and single-exponential fitting, X^2 = 1.848).*

4-(9-methyl-9,10-dihydroacridin-9-yl)benzonitrile (4c)

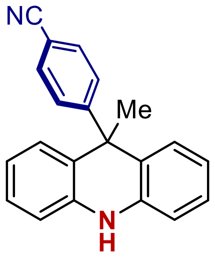

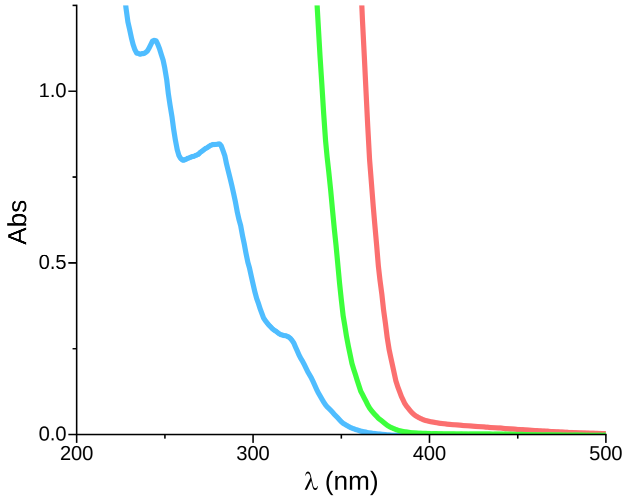

Concentration (mmol/mL)	
	5·10
	$5 \cdot 10^{-4}$
	$5 \cdot 10^{-5}$
λ_{peak}	280 nm
$\lambda_{shoulder}$	320 nm

Figure S13: UV-Vis absorption of ***4c*** *at different concentrations. 5·10 M (red trace); $5 \cdot 10^{-4}$ M(green trace); $5 \cdot 10^{-5}$ M (blue trace). Recorded in MeCN in quartz cuvettes (1 cm optical path).*

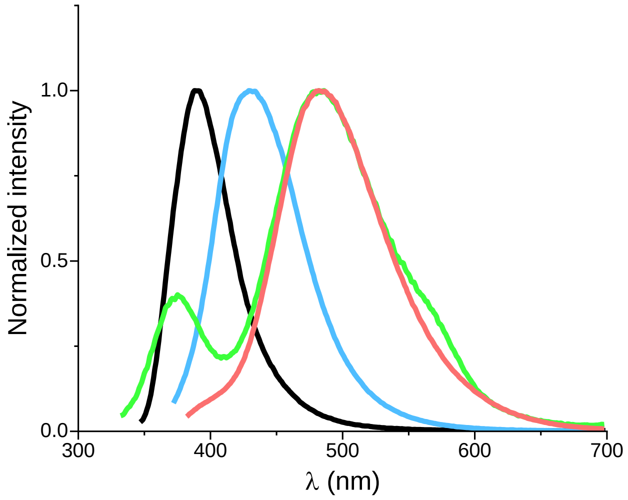

$\lambda_{excitation}$ = 280nm

	Solvent	Peak (nm)
	Toluene	389
	THF	431
	MeCN	484
	DMF	484

Figure S14: Normalized fluorescence emission of ***4c*** *$2 \cdot 10^{-5}$ M in different solvents. Toluene (black trace); THF (blue trace); MeCN (green trace); DMF (red trace).*

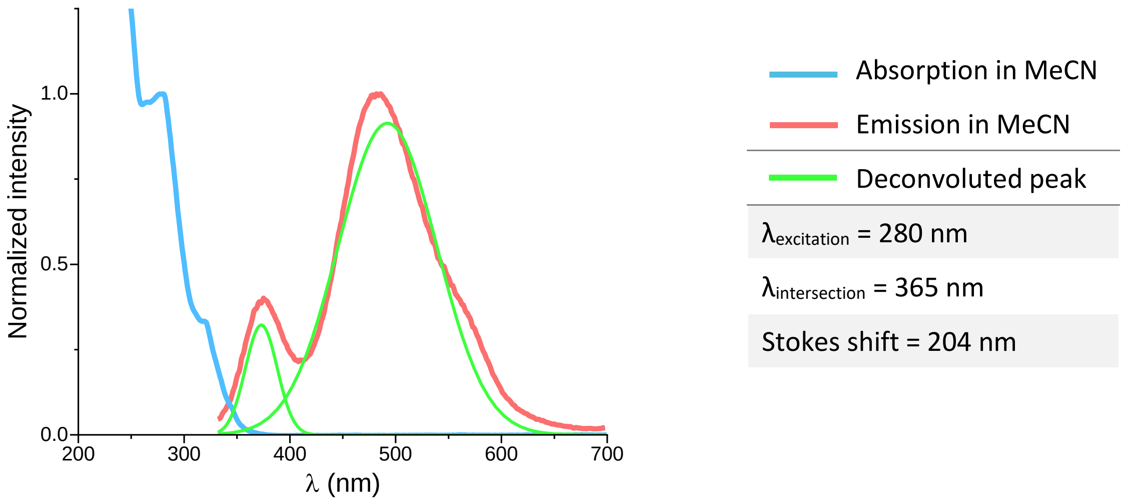

*Figure S15: Normalized optical absorption spectrum (blue trace) and emission spectra (red trace) of **4c**. The emission peaks were deconvoluted using the « peak analyser » function in OriginPro. The intersection wavelenght was calculated using the deconvoluted trace.*

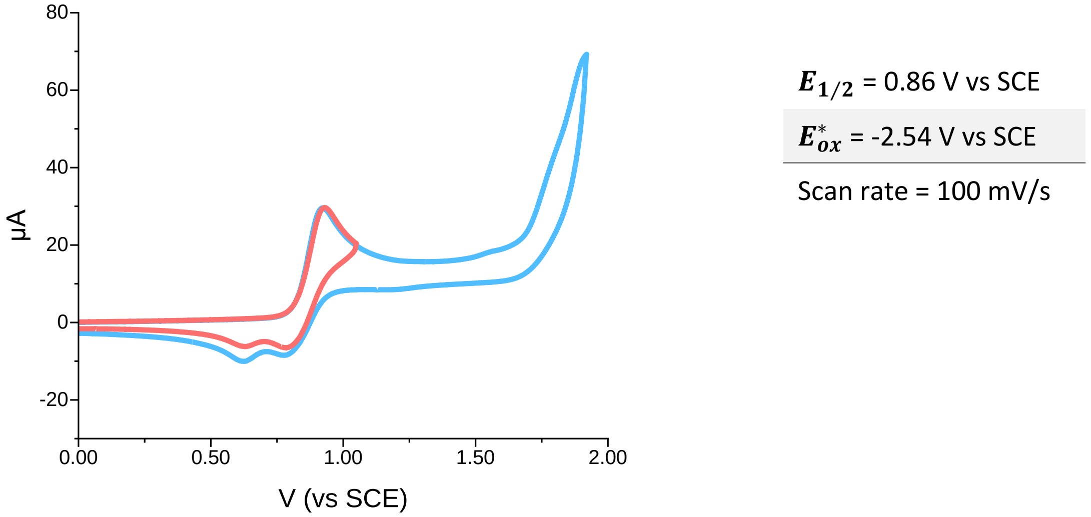

*Figure S16: Cyclic voltammograms of **4c** 10^{-3} M**.** Recorded with GC as the working electrode in MeCN with 0.1 M $[Bu_4N][PF_6]$ as the supporting electrolyte at 0.1 V/s scan rate.*

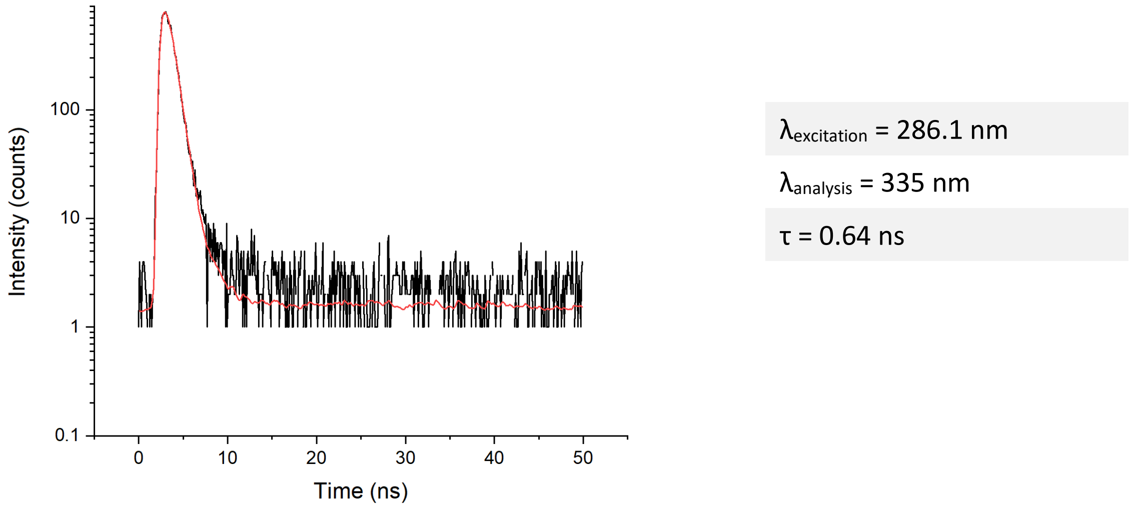

*Figure S17: Time-resolved emission decay (excitation at 286.1 nm, analysis at 335 nm) of **4c** $2{\cdot}10^{-5}$ M in MeCN solution measured by TC-SPC (τ = 0.64 ns, from deconvolution and single-exponential fitting, X^2 = 0.909).*

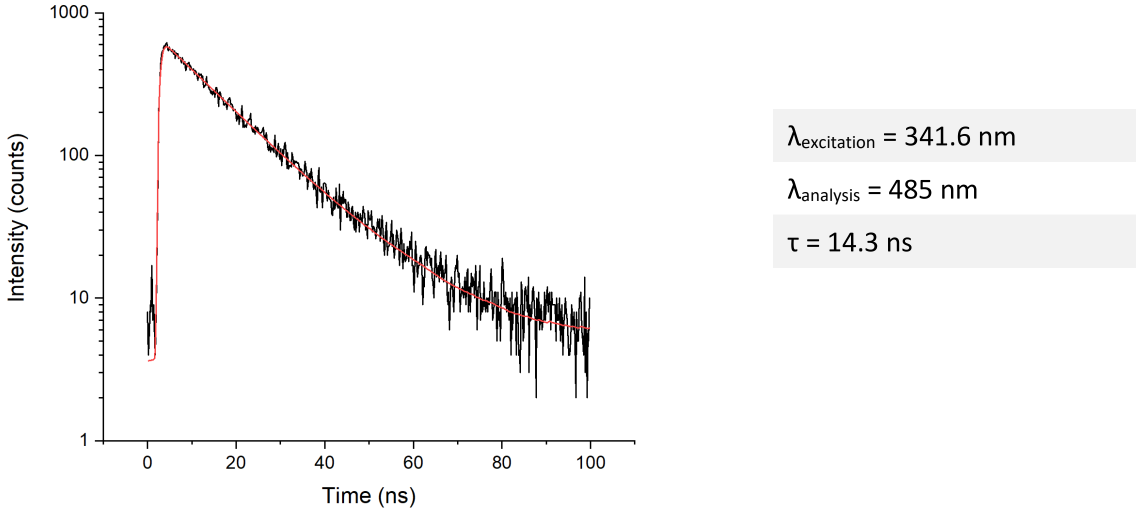

*Figure S18: Time-resolved emission decay (excitation at 341.6 nm, analysis at 485 nm) of **4c** $2{\cdot}10^{-5}$ M in MeCN solution measured by TC-SPC (τ = 14.3 ns, from deconvolution and single-exponential fitting, X^2 = 1.132).*

4-(9,10-dimethyl-9,10-dihydroacridin-9-yl)benzonitrile (4d)

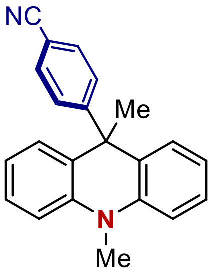

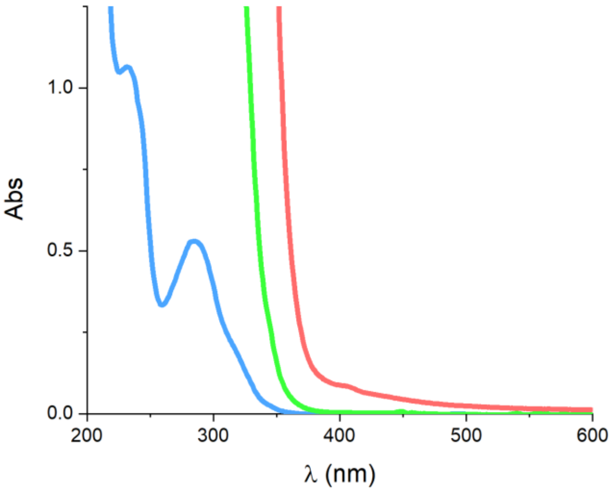

Concentration (mmol/mL)	
	$5\cdot10^{-3}$
	$5\cdot10^{-4}$
	$5\cdot10^{-5}$
λ_{peak}	285 nm
$\lambda_{shoulder}$	320 nm

Figure S19: UV-Vis absorption of ***4d*** *at different concentrations.* $5\cdot10^{-3}$ *M (red trace);* $5\cdot10^{-4}$ *M (green trace);* $5\cdot10^{-5}$ *M (blue trace). Recorded in MeCN in quartz cuvettes (1 cm optical path).*

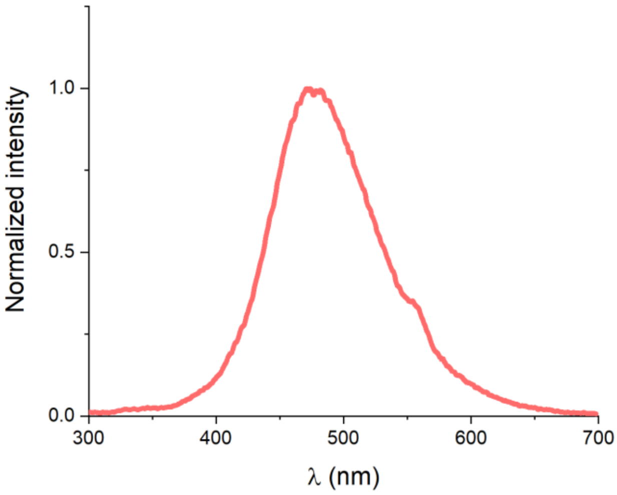

$\lambda_{excitation}$ = 280 nm

	Solvent	Peak (nm)
	MeCN	475

Figure S20: Normalized fluorescence emission of ***4d*** $2\cdot10^{-5}$ *M in MeCN.*

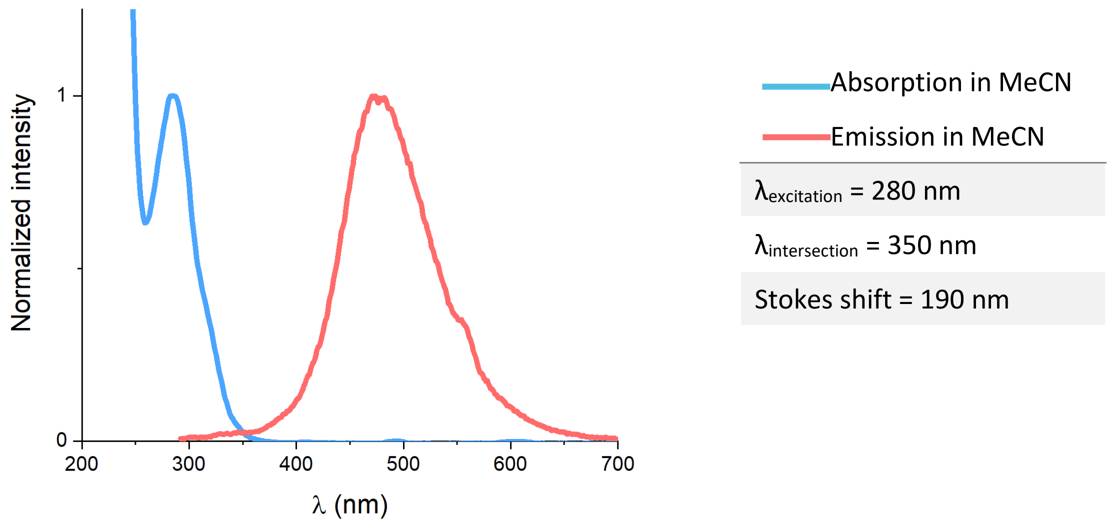

*Figure S21: Normalized optical absorption spectrum (blue trace) and emission spectra (red trace) of **4d**.*

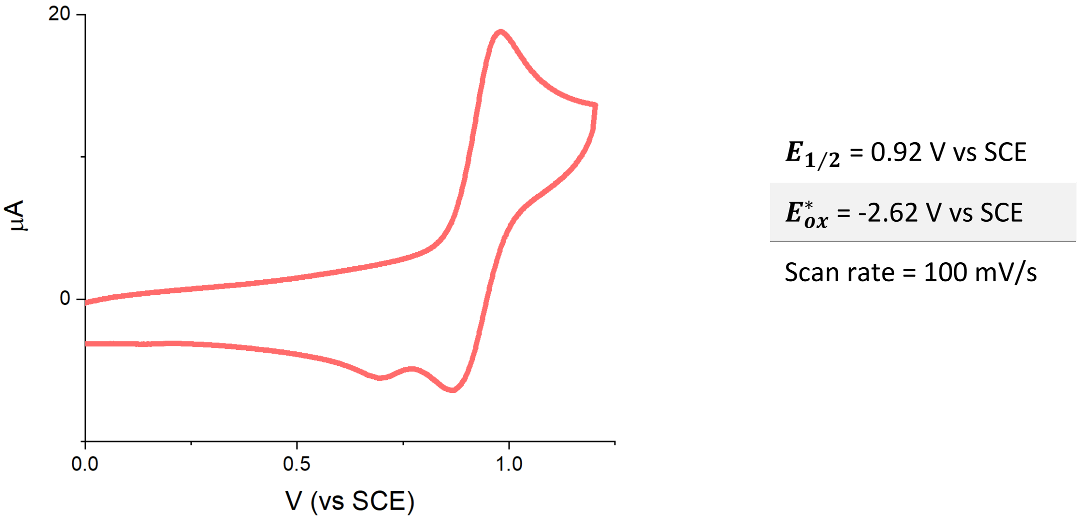

*Figure S22: Cyclic voltammograms of **4d** 10^{-3} M. Recorded with GC as the working electrode in MeCN with 0.1 M $[Bu_4N][PF_6]$ as the supporting electrolyte at 0.1 V/s scan rate.*

12-methyl-12-phenyl-7,12-dihydrobenzo[a]acridine (5a)

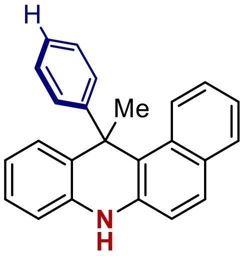

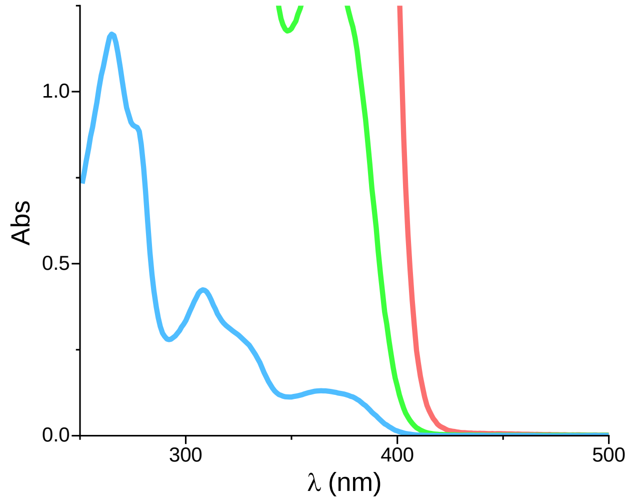

	Concentration (mmol/mL)
	5·10
	$5·10^{-4}$
	$5·10^{-5}$
λ_{peak} (1)	308nm
λ_{peak} (2)	364nm

*Figure S23: UV-Vis absorption of **5a** at different concentrations. 5·10 M (red trace); $5·10^{-4}$ M (green trace); $5·10^{-5}$ M (blue trace). Recorded in MeCN in quartz cuvettes (1 cm optical path).*

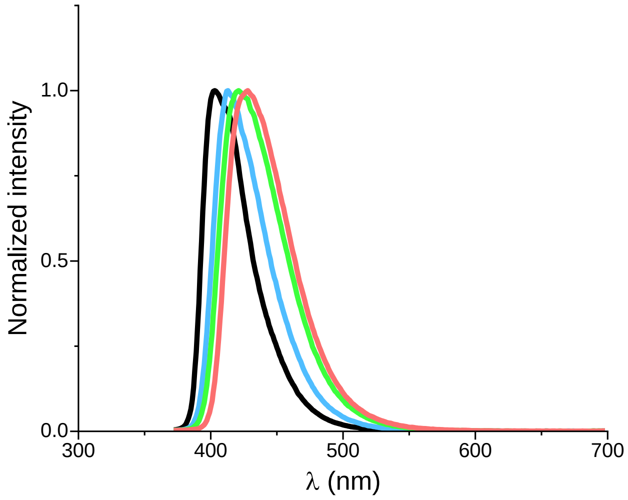

$\lambda_{excitation}$ = 364nm

	Solvent	Peak (nm)
	Toluene	403
	THF	413
	MeCN	420
	DMF	428

*Figure S24: Normalized fluorescence emission of **5a** $2·10^{-5}$ M in different solvents. Toluene (black trace); THF (blue trace); MeCN (green trace); DMF (red trace).*

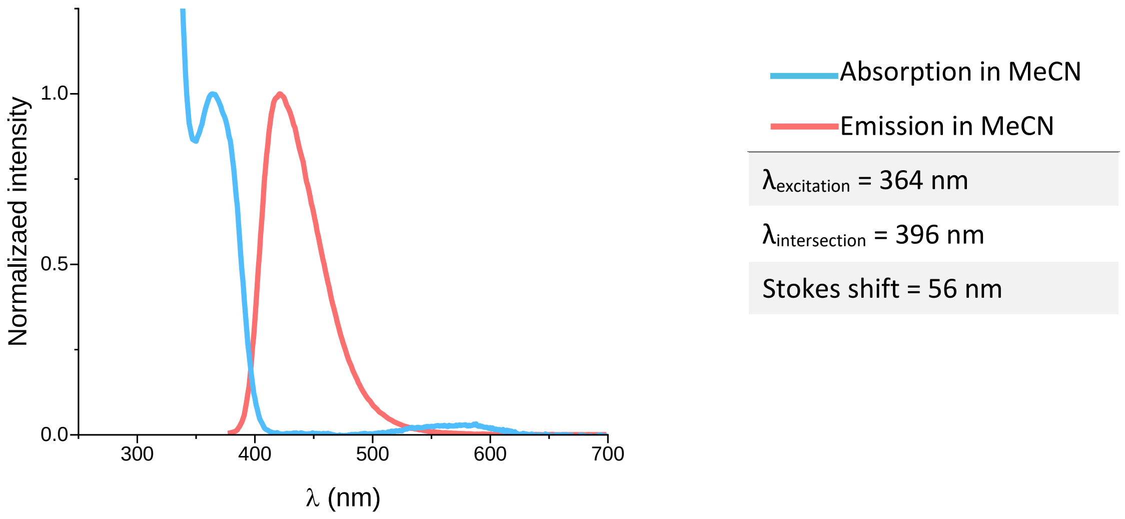

*Figure S25: Normalized optical absorption spectrum (blue trace) and emission spectra (red trace) of **5a**.*

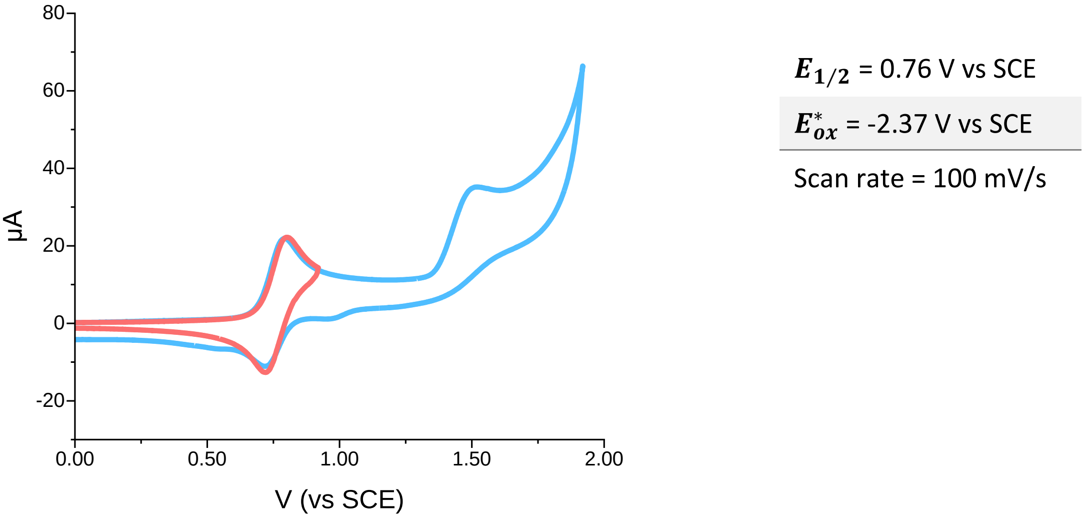

*Figure S26: Cyclic voltammograms of **5a** 10^{-3} M. Recorded with GC as the working electrode in MeCN with 0.1 M $[Bu_4N][PF_6]$ as the supporting electrolyte at 0.1 V/s scan rate.*

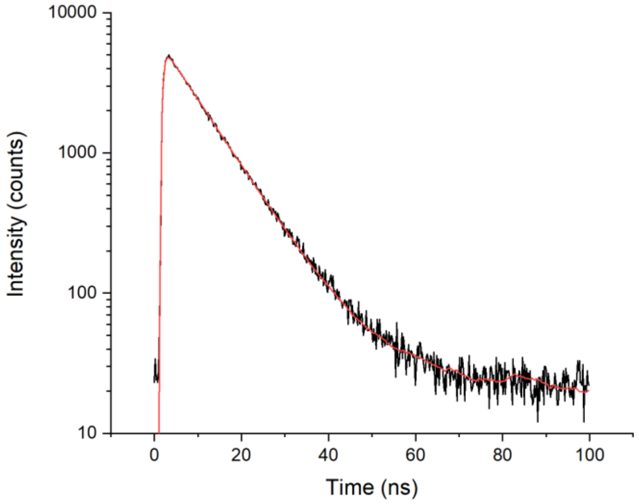

$\lambda_{excitation}$ = 286.1 nm

$\lambda_{analysis}$ = 420 nm

τ = 9.0 ns

*Figure S27: Time-resolved emission decay (excitation at 286.1 nm, analysis at 420 nm) of **5a** 2·10^{-5} M in MeCN solution measured by TC-SPC (τ = 9.0 ns, from deconvolution and single-exponential fitting, X^2 = 1.496).*

12-(4-methoxyphenyl)- 12-methyl-7,12-dihydrobenzo[a]acridine (5b)

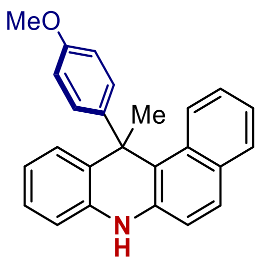

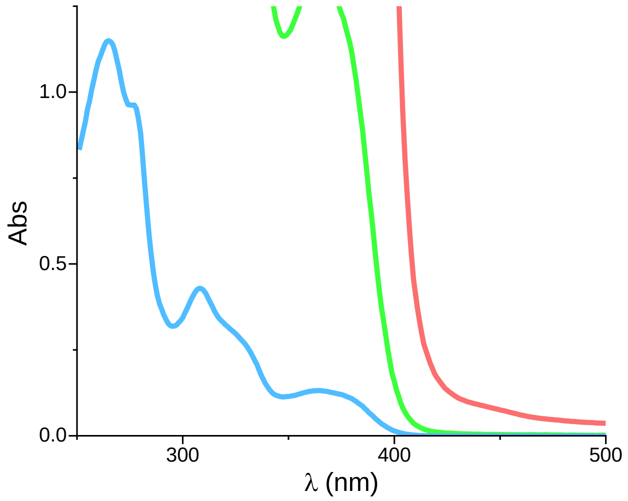

Concentration (mmol/mL)	
red	$5\cdot10^{-3}$
green	$5\cdot10^{-4}$
blue	$5\cdot10^{-5}$
λ_{peak} (1)	307nm
λ_{peak} (2)	363nm

*Figure S28: UV-Vis absorption of **5b** at different concentrations. 5·10 M (red trace); $5\cdot10^{-4}$ M(green trace); $5\cdot10^{-5}$ M (blue trace). Recorded in MeCN in quartz cuvettes (1 cm optical path).*

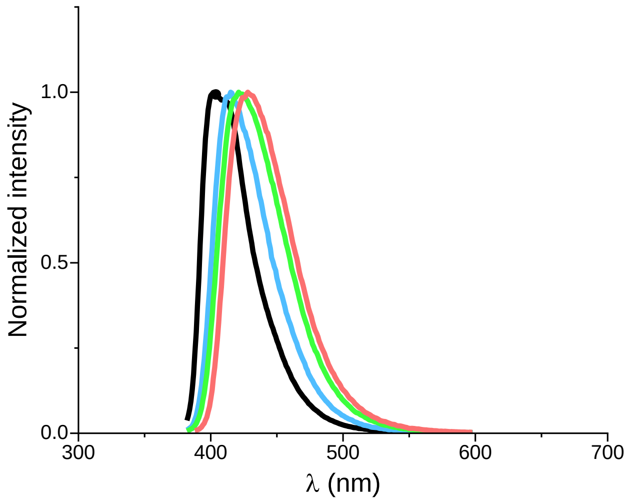

$\lambda_{excitation}$ = 364nm

	Solvent	Peak (nm)
black	Toluene	403
blue	THF	415
green	MeCN	421
red	DMF	428

*Figure S29: Normalized fluorescence emission of **5b** $2\cdot10^{-5}$ M in different solvents. Toluene (black trace); THF (blue trace); MeCN (green trace); DMF (red trace).*

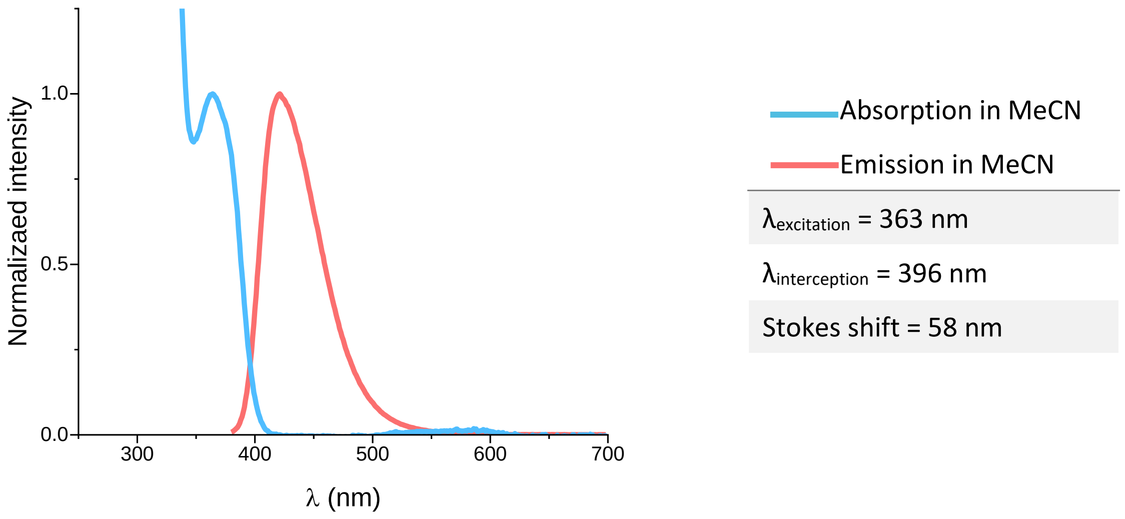

*Figure S30: Normalized optical absorption spectrum (blue trace) and emission spectra (red trace) of **5b**.*

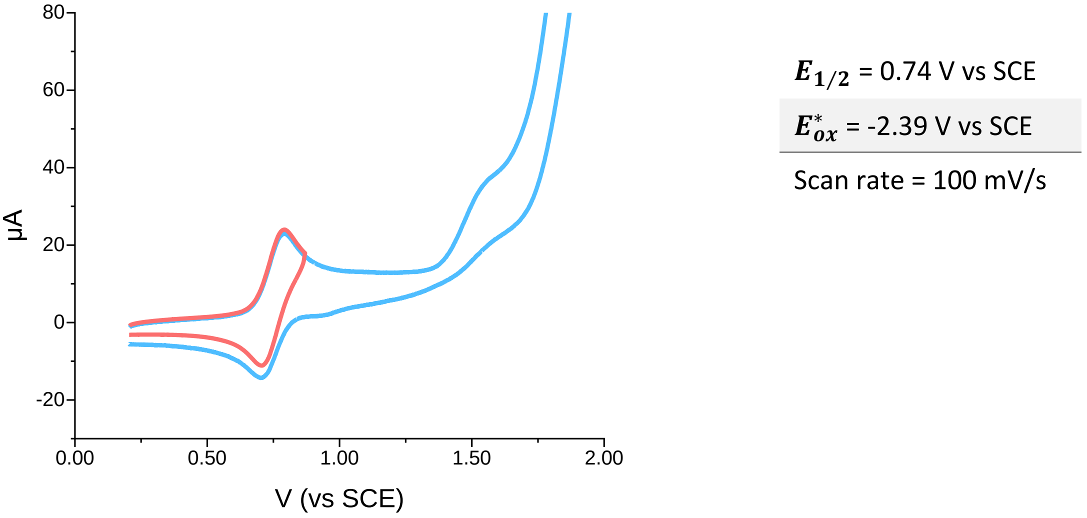

*Figure S31: Cyclic voltammograms of **5b** 10^{-3} M. Recorded with GC as the working electrode in MeCN with 0.1 M [Bu_4N][PF_6] as the supporting electrolyte at 0.1 V/s scan rate.*

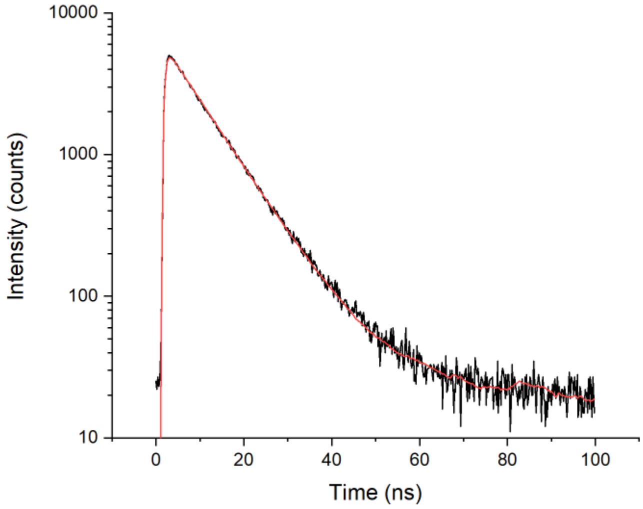

$\lambda_{excitation}$ = 286.1 nm

$\lambda_{analysis}$ = 420 nm

τ = 9.0 ns

Figure S32: Time-resolved emission decay (excitation at 286.1 nm, analysis at 420 nm) of ***5b*** *$2\cdot10^{-5}$ M in MeCN solution measured by TC-SPC (τ = 9.0 ns, from deconvolution and single-exponential fitting, X^2 = 1.517).*

12-(4-(trifluoromethyl)phenyl)- 12-methyl-7,12-dihydrobenzo[a]acridine (5c)

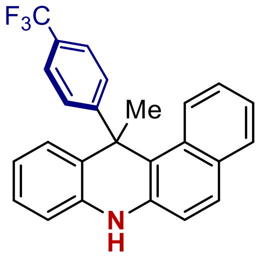

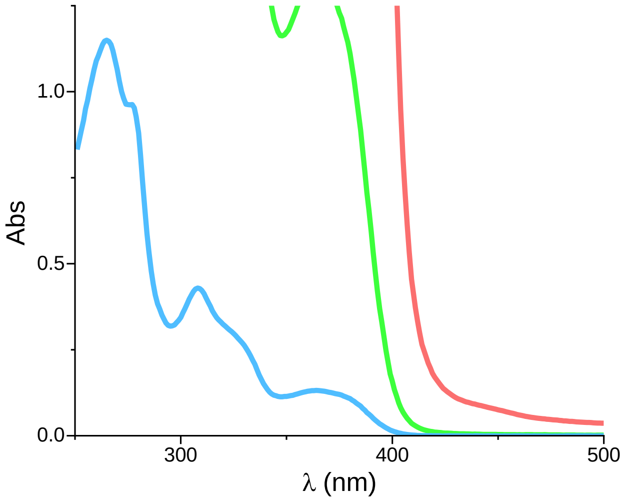

	Concentration (mmol/mL)
(red)	$5{\cdot}10^{-3}$
(green)	$5{\cdot}10^{-4}$
(blue)	$5{\cdot}10^{-5}$
λ_{peak} (1)	307nm
λ_{peak} (2)	364nm

*Figure S33: UV-Vis absorption of **5c** at different concentrations. 5·10 M (red trace); $5{\cdot}10^{-4}$ M(green trace); $5{\cdot}10^{-5}$ M (blue trace). Recorded in MeCN in quartz cuvettes (1 cm optical path).*

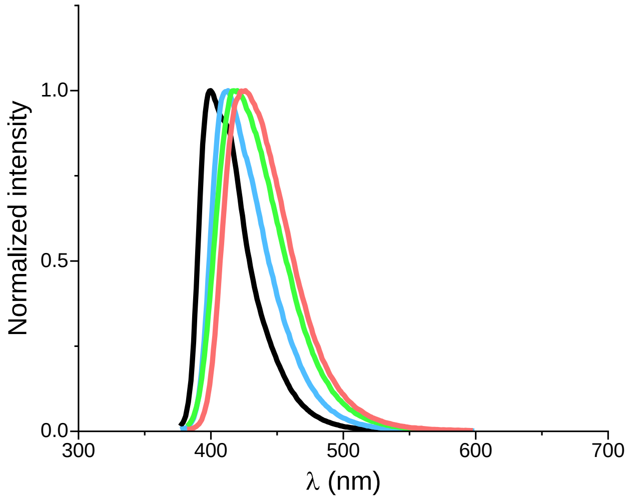

$\lambda_{excitation}$ = 364nm

	Solvent	Peak (nm)
(black)	Toluene	400
(blue)	THF	412
(green)	MeCN	420
(red)	DMF	424

*Figure S34: Normalized fluorescence emission of **5c** $2{\cdot}10^{-5}$ M in different solvents. Toluene (black trace); THF (blue trace); MeCN (green trace); DMF (red trace).*

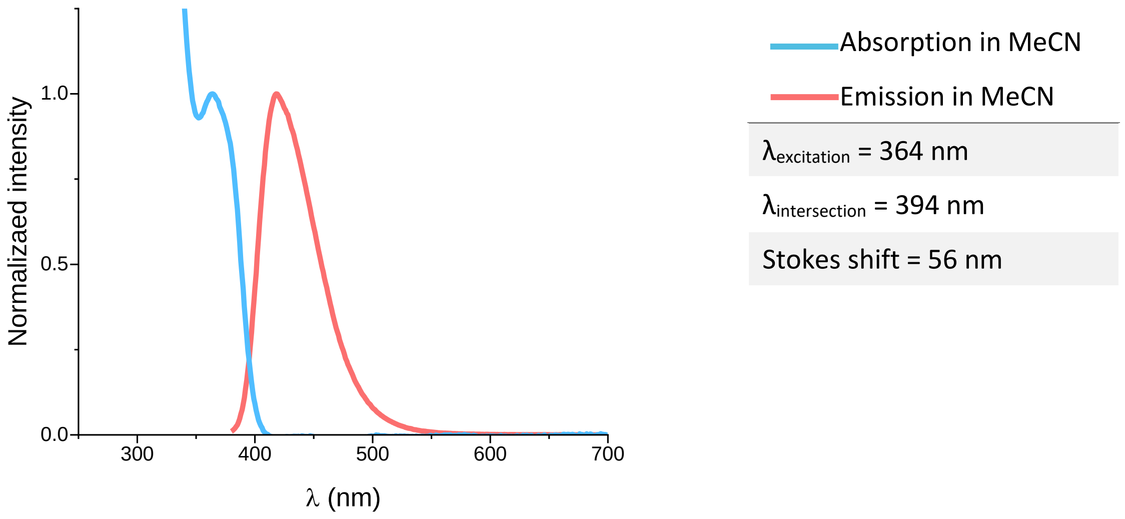

*Figure S35: Normalized optical absorption spectrum (blue trace) and emission spectra (red trace) of **5c.***

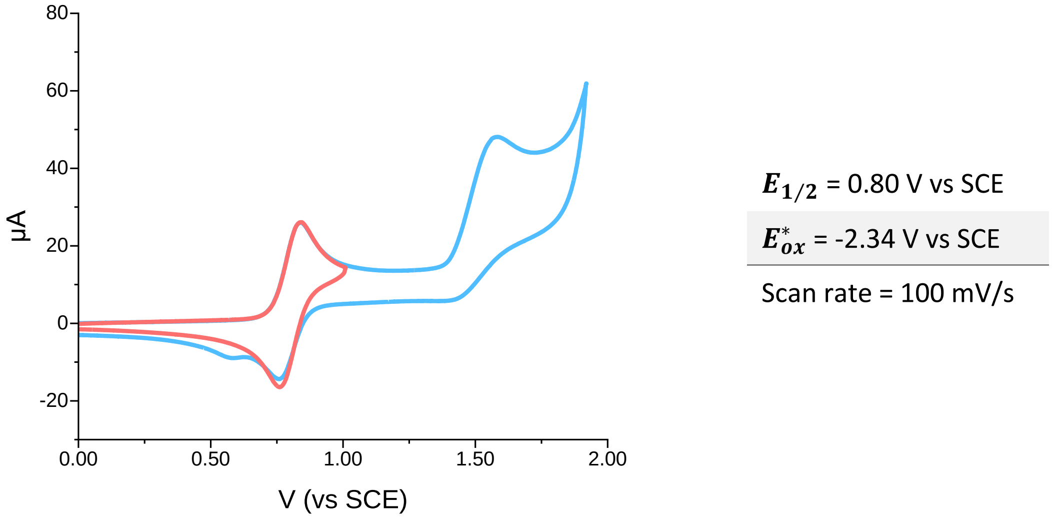

*Figure S36: Cyclic voltammograms of **5c** 10^{-3} M**.** Recorded with GC as the working electrode in MeCN with 0.1 M $[Bu_4N][PF_6]$ as the supporting electrolyte at 0.1 V/s scan rate.*

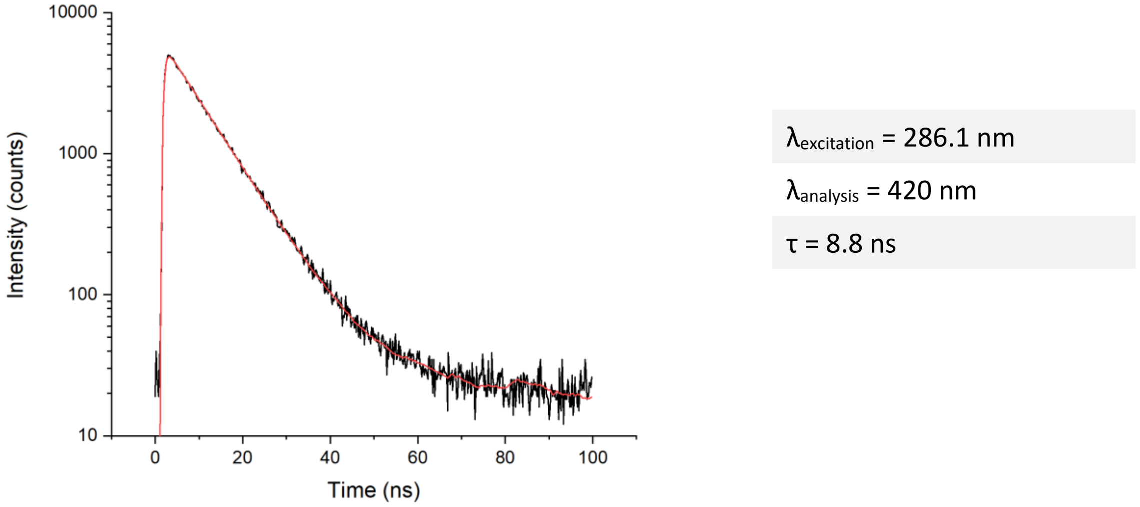

Figure S37: Time-resolved emission decay (excitation at 286.1 nm, analysis at 420 nm) of ***5c*** *$2{\cdot}10^{-5}$ M in MeCN solution measured by TC-SPC (τ = 8.8 ns, from deconvolution and single-exponential fitting, X^2 = 1.433).*

4-(12-methyl-7,12-dihydrobenzo[a]acridin-12-yl)benzonitrile (5d)

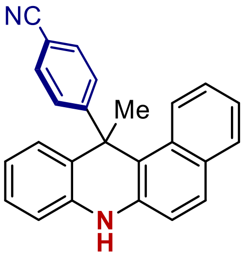

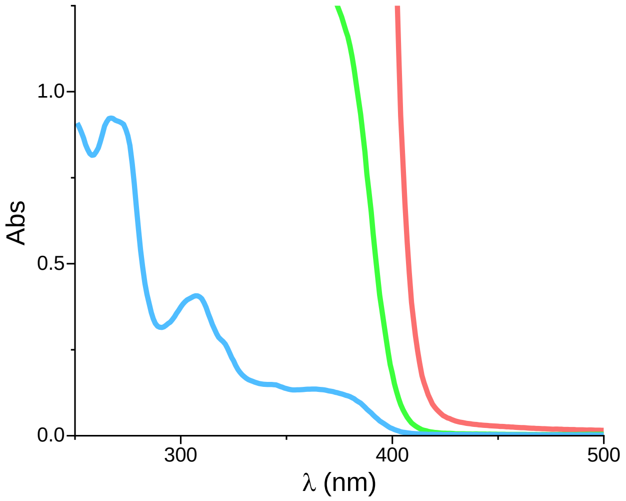

	Concentration (mmol/mL)
red	$5\cdot10^{-3}$
green	$5\cdot10^{-4}$
blue	$5\cdot10^{-5}$
λ_{peak} (1)	308nm
λ_{peak} (2)	362nm

*Figure S38: UV-Vis absorption of **5d** at different concentrations. 5·10 M (red trace); $5\cdot10^{-4}$ M(green trace); $5\cdot10^{-5}$ M (blue trace). Recorded in MeCN in quartz cuvettes (1 cm optical path).*

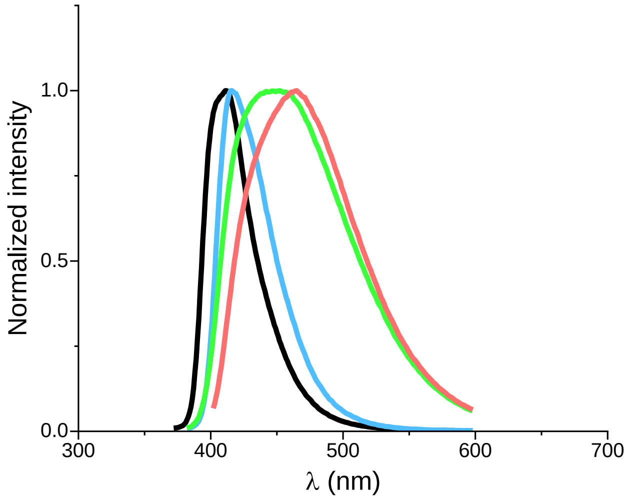

$\lambda_{excitation}$ = 364nm

	Solvent	Peak (nm)
black	Toluene	410
blue	THF	416
green	MeCN	443
red	DMF	464

*Figure S39: Normalized fluorescence emission of **5d** $2\cdot10^{-5}$ M in different solvents. Toluene (black trace); THF (blue trace); MeCN (green trace); DMF (red trace).*

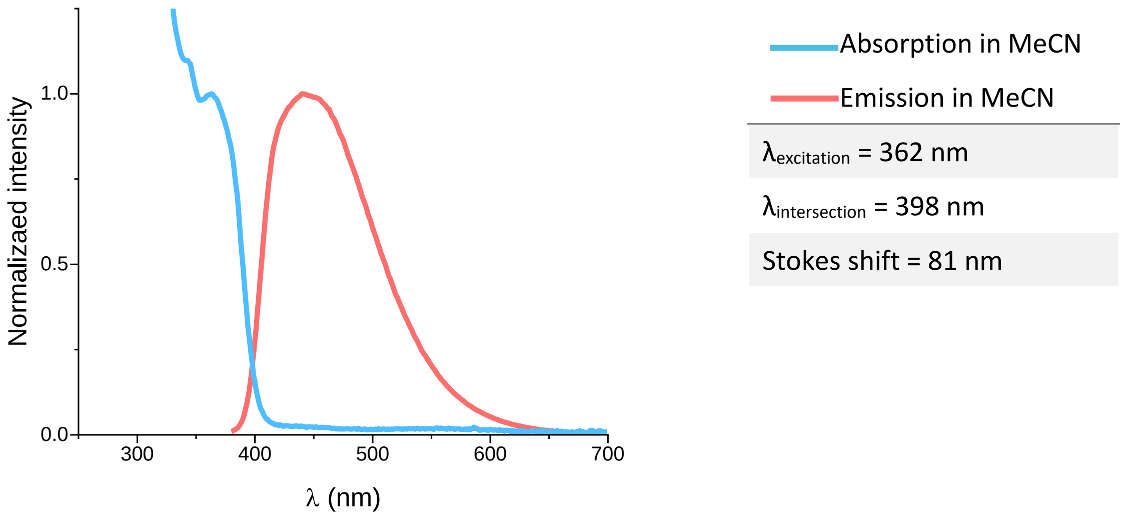

Figure S40: Normalized optical absorption spectrum (blue trace) and emission spectra (red trace) of ***5d.***

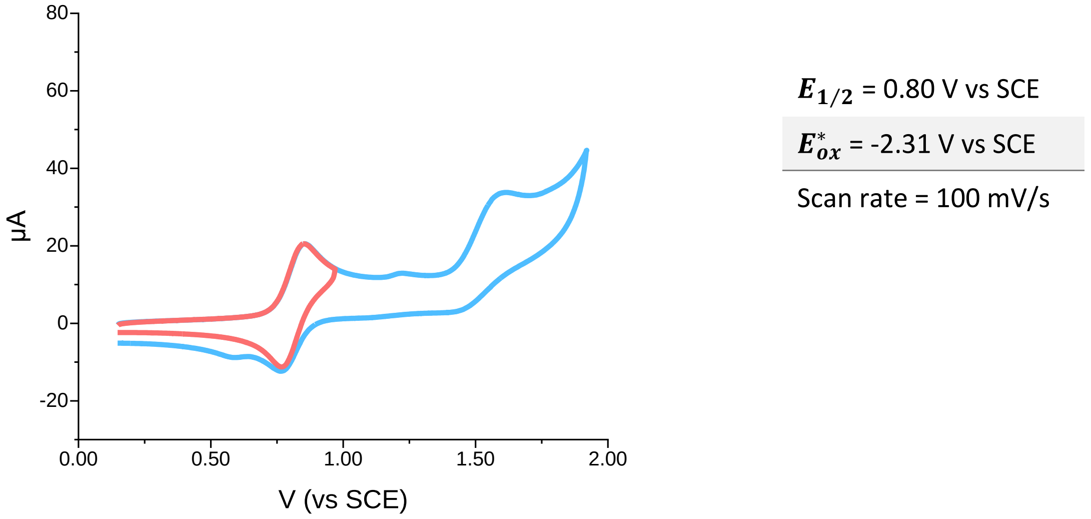

Figure S41: Cyclic voltammograms of ***5d*** *10^{-3} M. Recorded with GC as the working electrode in MeCN with 0.1 M $[Bu_4N][PF_6]$ as the supporting electrolyte at 0.1 V/s scan rate.*

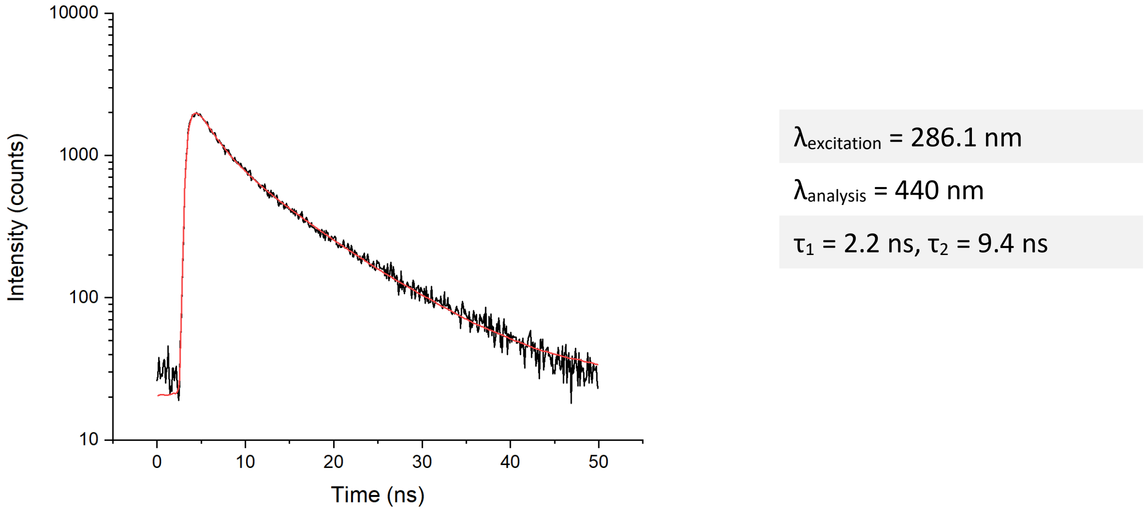

Figure S42: Time-resolved emission decay (excitation at 341.6 nm, analysis at 440 nm) of ***5d*** *$2{\cdot}10^{-5}$ M in MeCN solution measured by TC-SPC (τ_1 = 2.2 ns, τ_2 = 9.4 ns from deconvolution and double-exponential fitting, X^2 = 1.181).*

7,12-dimethyl-12-phenyl-7,12-dihydrobenzo[a]acridine (5e)

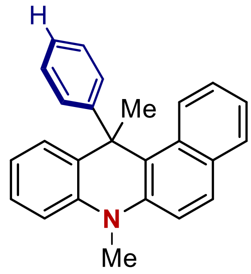

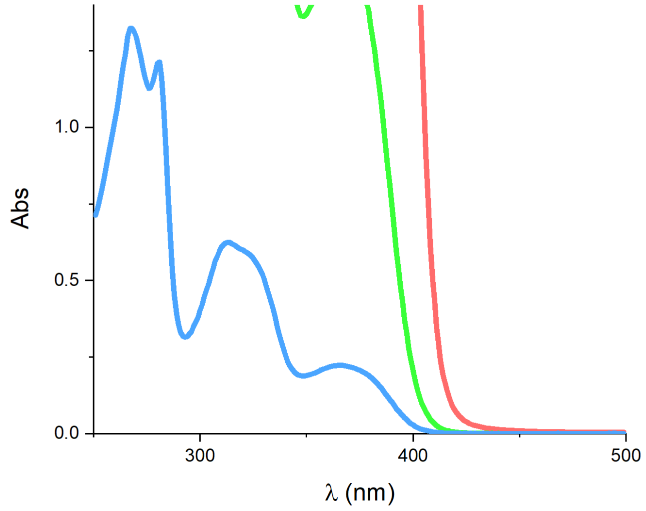

Concentration (mmol/mL)	
red trace	5·10
green trace	$5{\cdot}10^{-4}$
blue trace	$5{\cdot}10^{-5}$
λ_{peak} (1)	313 nm
λ_{peak} (2)	365 nm

*Figure S43: UV-Vis absorption of **5e** at different concentrations. 5·10 M (red trace); $5{\cdot}10^{-4}$ M (green trace); $5{\cdot}10^{-5}$ M (blue trace). Recorded in MeCN in quartz cuvettes (1 cm optical path).*

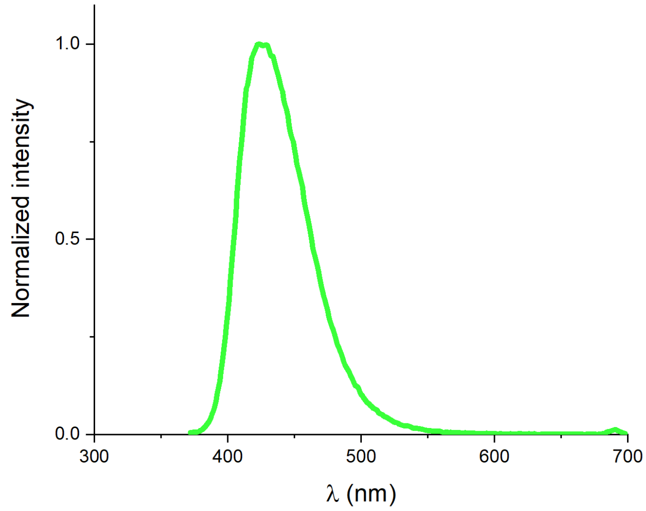

$\lambda_{excitation}$ = 345nm

	Solvent	Peak (nm)
green trace	MeCN	425

*Figure S44: Normalized fluorescence emission of **5e** $2{\cdot}10^{-5}$ M in MeCN.*

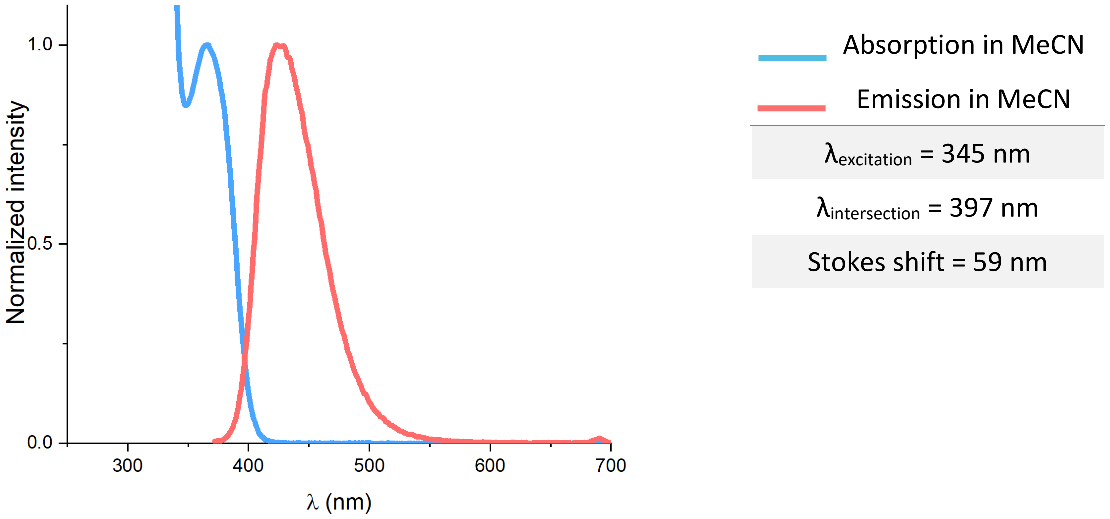

*Figure S45: Normalized optical absorption spectrum (blue trace) and emission spectrum (red trace) of **5e**.*

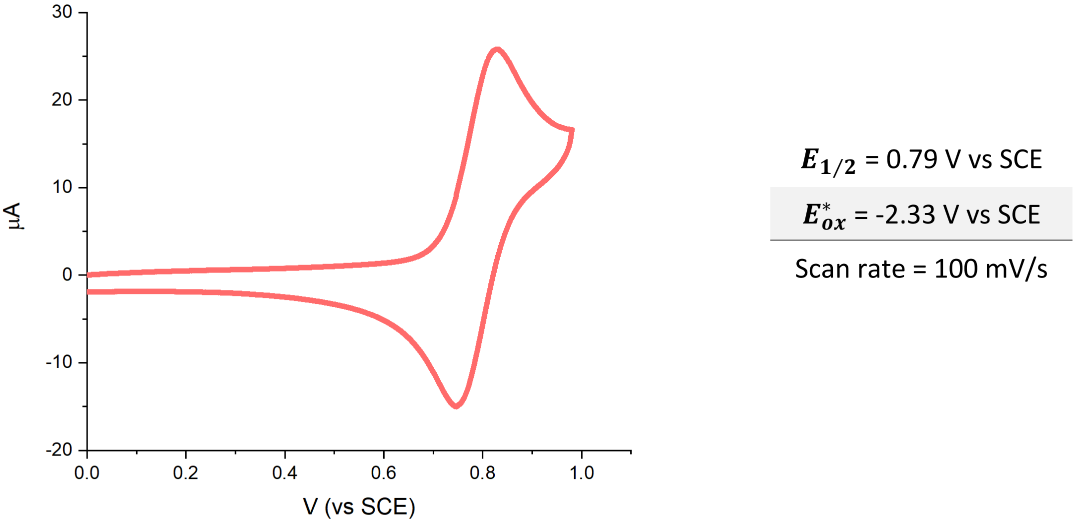

*Figure S46: Cyclic voltammogram of **5e** 10^{-3} M**.** Recorded with GC as the working electrode in MeCN with 0.1 M $[Bu_4N][PF_6]$ as the supporting electrolyte at 0.1 V/s scan rate.*

2,7-dimethoxy-9-methyl-9-phenyl-9,10-dihydroacridine (6a)

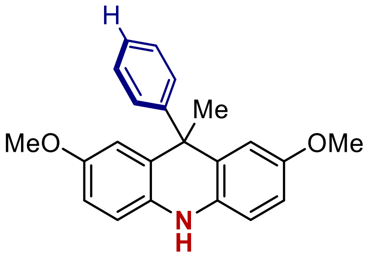

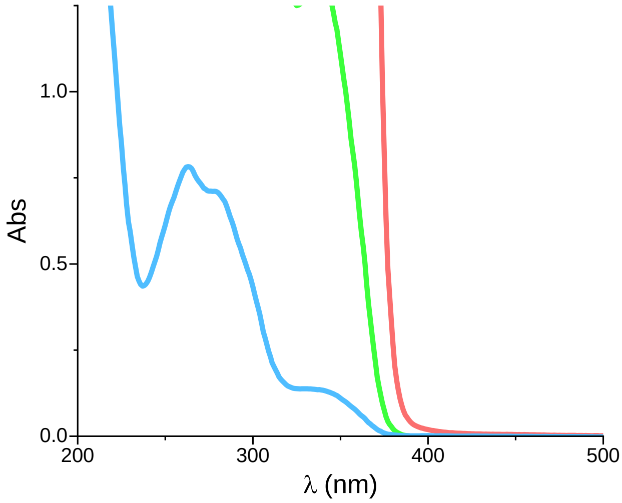

	Concentration (mmol/mL)
red trace	$5 \cdot 10^{-3}$
green trace	$5 \cdot 10^{-4}$
blue trace	$5 \cdot 10^{-5}$
λ_{peak} (1)	263nm
λ_{peak} (2)	338nm

*Figure S47: UV-Vis absorption of **6a** at different concentrations. 5·10 M (red trace); $5 \cdot 10^{-4}$ M(green trace); $5 \cdot 10^{-5}$ M (blue trace). Recorded in MeCN in quartz cuvettes (1 cm optical path).*

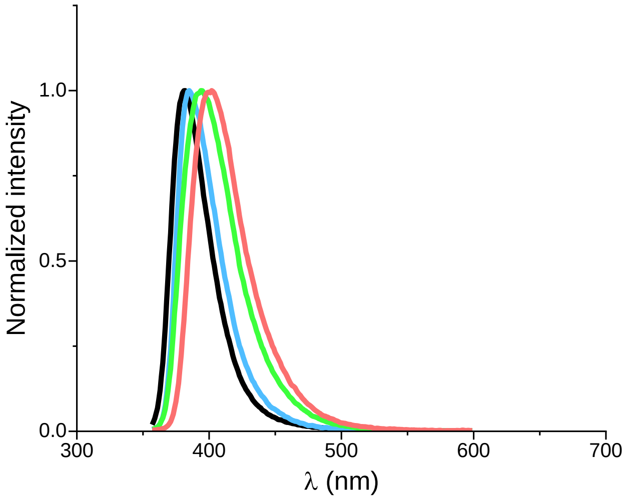

$\lambda_{excitation}$ = 364nm

	Solvent	Peak (nm)
black trace	Toluene	381
blue trace	THF	385
green trace	MeCN	394
red trace	DMSO	401

*Figure S48: Normalized fluorescence emission of **6a** $2 \cdot 10^{-5}$ M in different solvents. Toluene (black trace); THF (blue trace); MeCN (green trace); DMSO (red trace).*

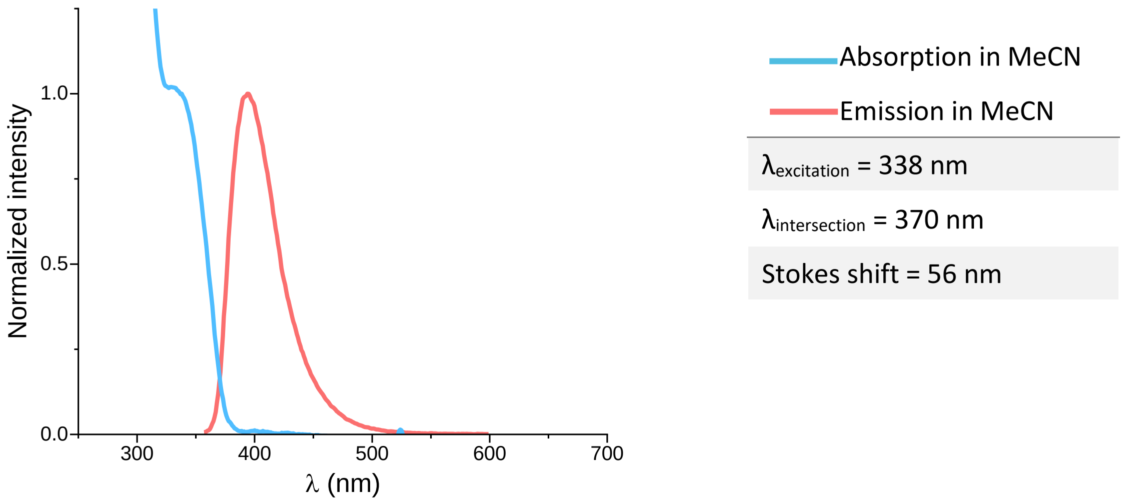

*Figure S49: Normalized optical absorption spectrum (blue trace) and emission spectra (red trace) of **6a.***

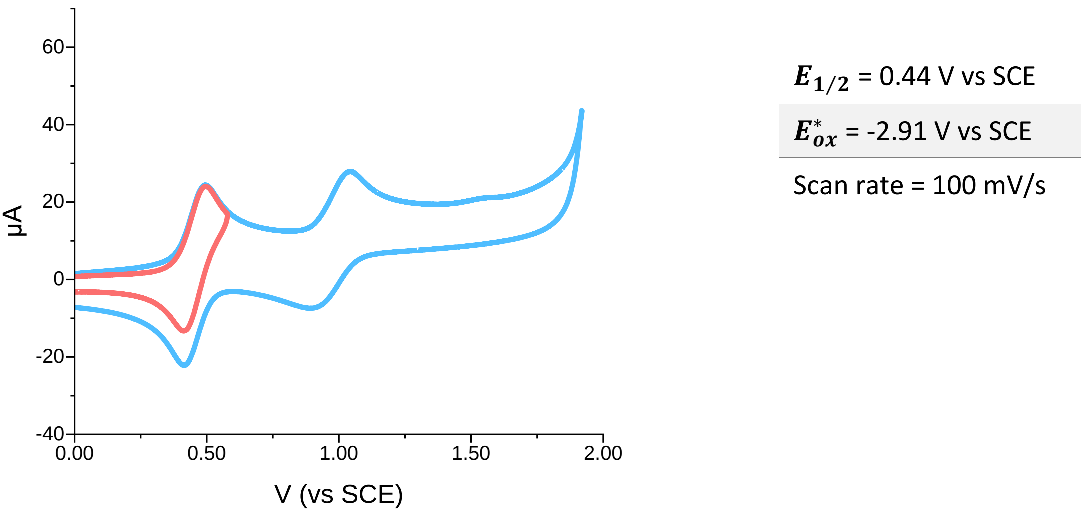

*Figure S50: Cyclic voltammograms of **6a** 10^{-3} M. Recorded with GC as the working electrode in MeCN with 0.1 M $[Bu_4N][PF_6]$ as the supporting electrolyte at 0.1 V/s scan rate.*

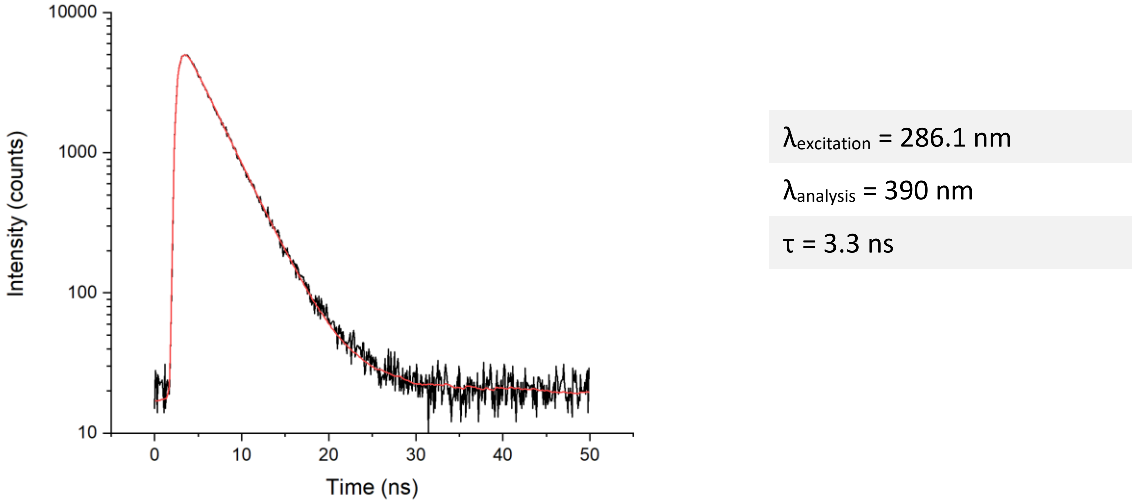

Figure S51: Time-resolved emission decay (excitation at 286.1 nm, analysis at 390 nm) of ***6a*** *2·10^{-5} M in MeCN solution measured by TC-SPC (τ = 3.3 ns, from deconvolution and single-exponential fitting, X^2 = 1.031).*

2,7-dimethoxy-9-(4-trifluoromethyl)-9-methyl-9,10-dihydroacridine (6b)

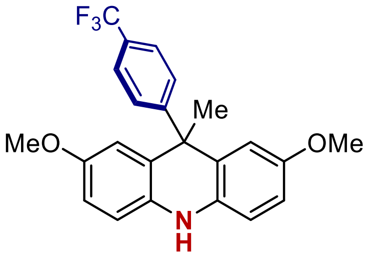

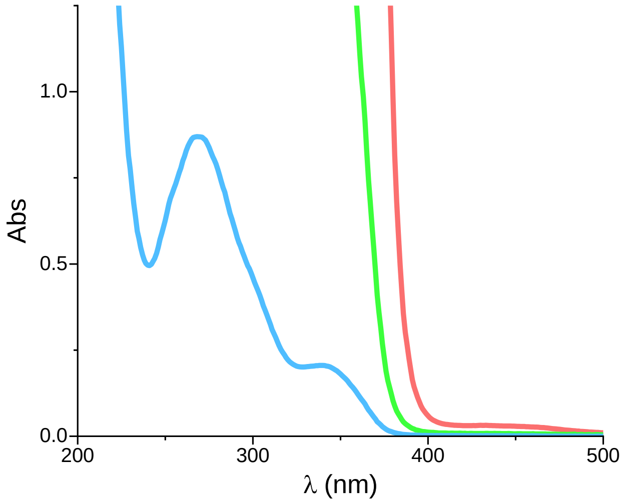

Concentration (mmol/mL)	
red trace	$5 \cdot 10^{-3}$
green trace	$5 \cdot 10^{-4}$
blue trace	$5 \cdot 10^{-5}$
λ_{peak} (1)	270 nm
λ_{peak} (2)	340 nm

*Figure S52: UV-Vis absorption of **6b** at different concentrations. 5·10 M (red trace); $5 \cdot 10^{-4}$ M(green trace); $5 \cdot 10^{-5}$ M (blue trace). Recorded in MeCN in quartz cuvettes (1 cm optical path).*

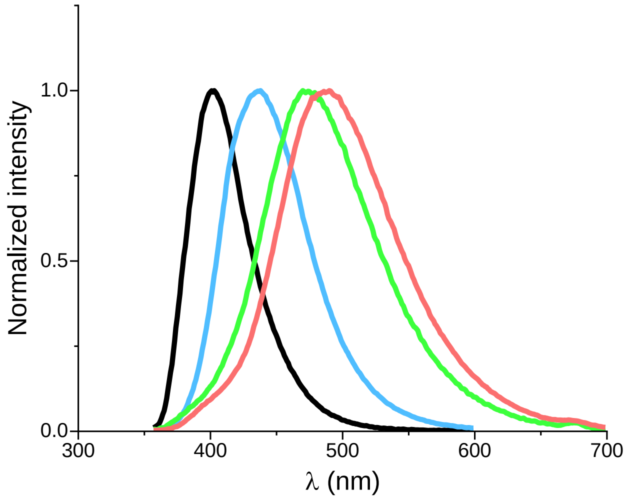

$\lambda_{excitation}$ = 364nm

	Solvent	Peak (nm)
black trace	Toluene	402
blue trace	THF	437
green trace	MeCN	473
red trace	DMSO	488

*Figure S53: Normalized fluorescence emission of **6b** $2 \cdot 10^{-5}$ M in different solvents. Toluene (black trace); THF (blue trace); MeCN (green trace); DMSO (red trace).*

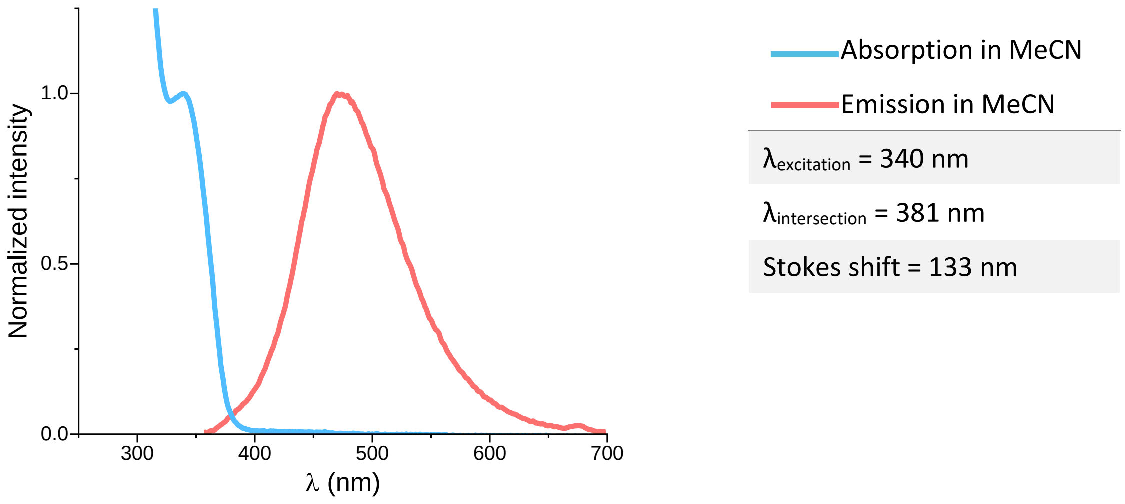

*Figure S54: Normalized optical absorption spectrum (blue trace) and emission spectra (red trace) of **6b.***

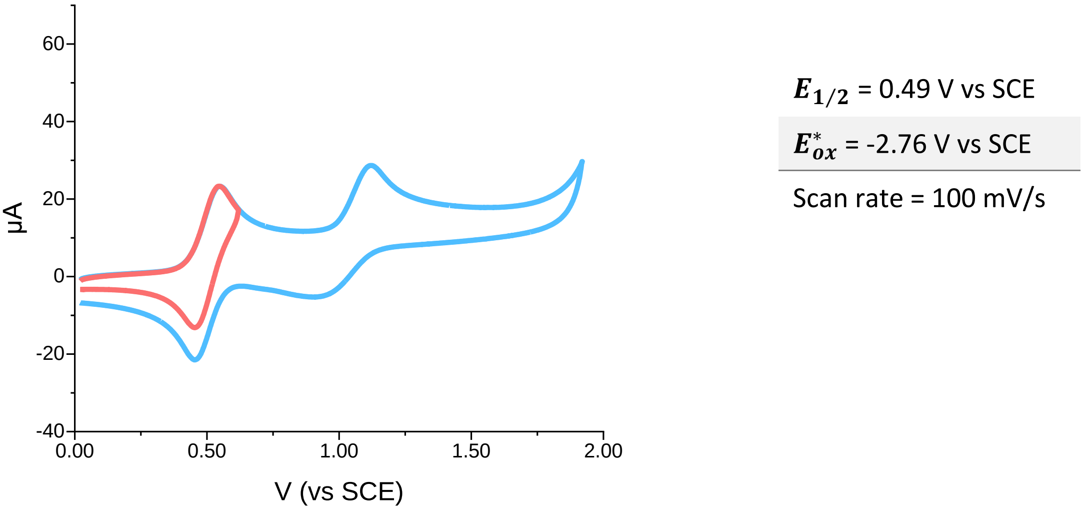

*Figure S55: Cyclic voltammograms of **6b** 10^{-3} M. Recorded with GC as the working electrode in MeCN with 0.1 M $[Bu_4N][PF_6]$ as the supporting electrolyte at 0.1 V/s scan rate.*

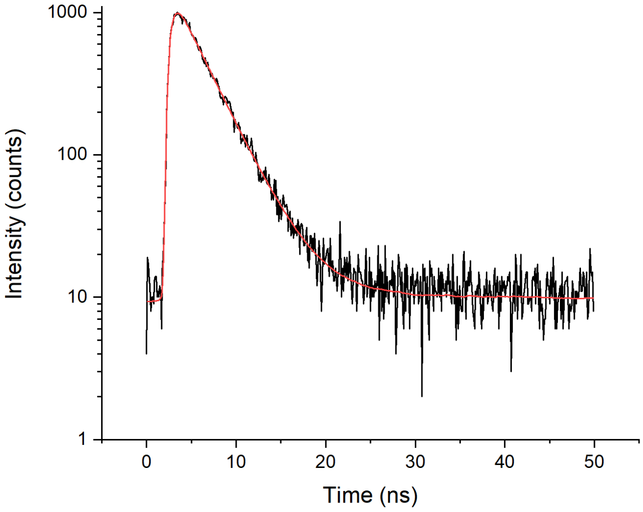

$\lambda_{excitation}$ = 286.1 nm

$\lambda_{analysis}$ = 480 nm

τ = 3.2 ns

*Figure S56: Time-resolved emission decay (excitation at 286.1 nm, analysis at 480 nm) of **6b** $2{\cdot}10^{-5}$ M in MeCN solution measured by TC-SPC (τ = 3.2 ns, from deconvolution and single-exponential fitting, X^2 = 1.224).*

4-(2,7-dimethoxy-9-methyl-9,10-dihydroacridin-9-yl)benzonitrile (6c)

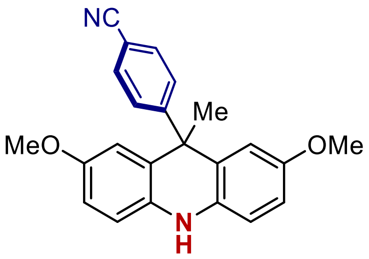

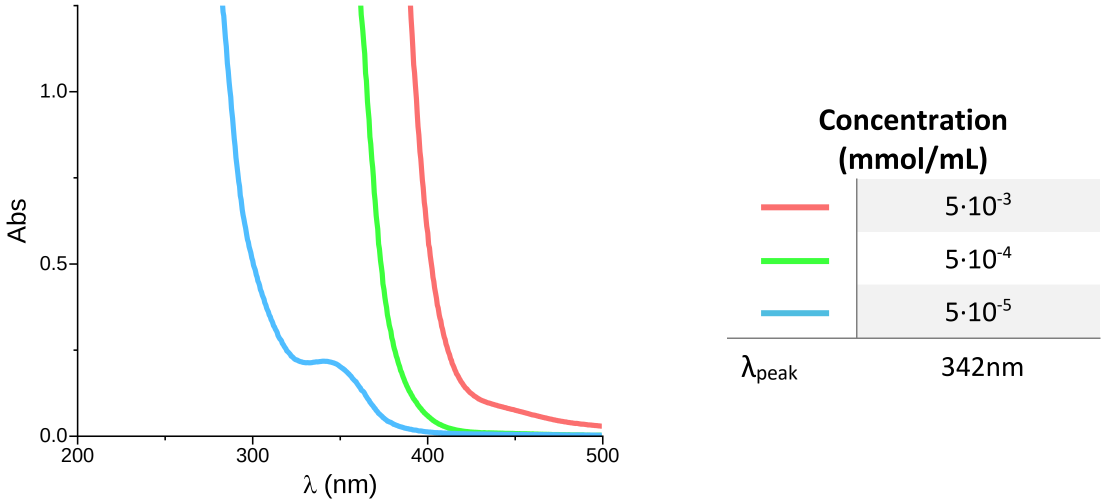

*Figure S57: UV-Vis absorption of **6c** at different concentrations. 5·10 M (red trace); 5·10^{-4} M (green trace); 5·10^{-5} M (blue trace). Recorded in MeCN in quartz cuvettes (1 cm optical path).*

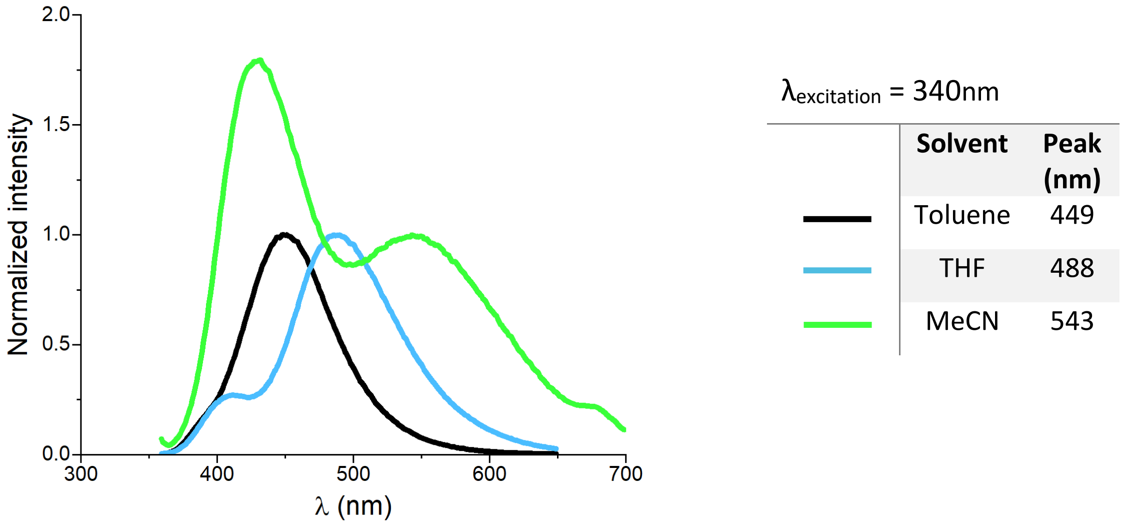

*Figure S58: Normalized fluorescence emission of **6c** 2·10^{-5} M in different solvents. Toluene (black trace); THF (blue trace); MeCN (green trace).*

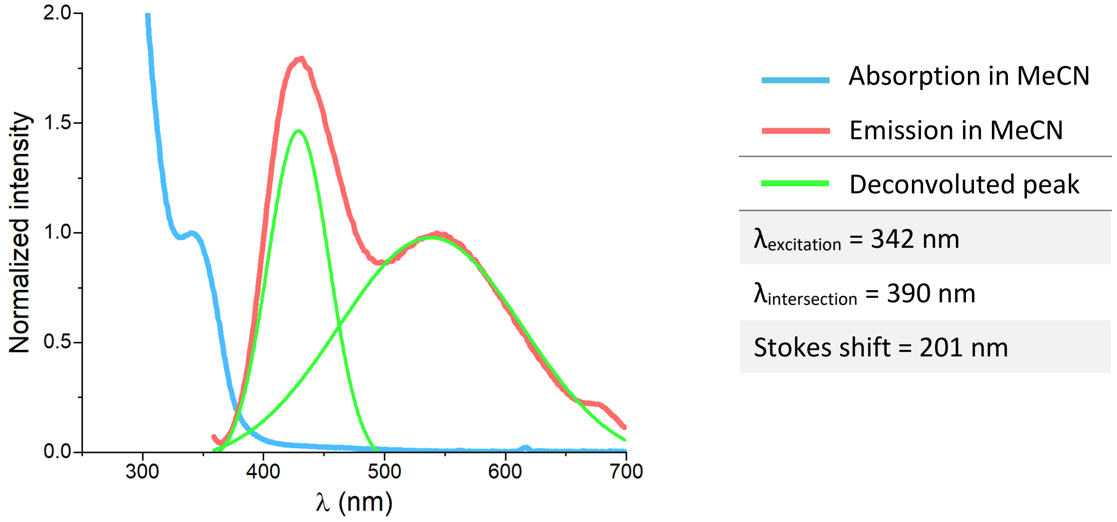

*Figure S59: Normalized optical absorption spectrum (blue trace) and emission spectra (red trace) of **6c**. The emission peaks were deconvoluted using the « peak analyser » function in OriginPro. The intersection wavelength was calculated using the deconvoluted trace.*

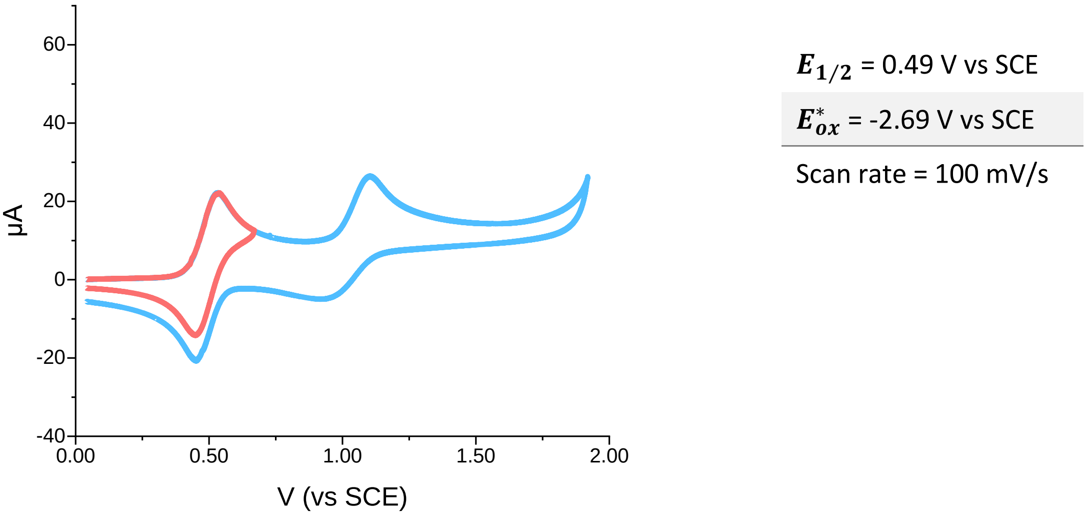

*Figure S60: Cyclic voltammograms of **6c** 10^{-3} M. Recorded with GC as the working electrode in MeCN with 0.1 M $[Bu_4N][PF_6]$ as the supporting electrolyte at 0.1 V/s scan rate.*

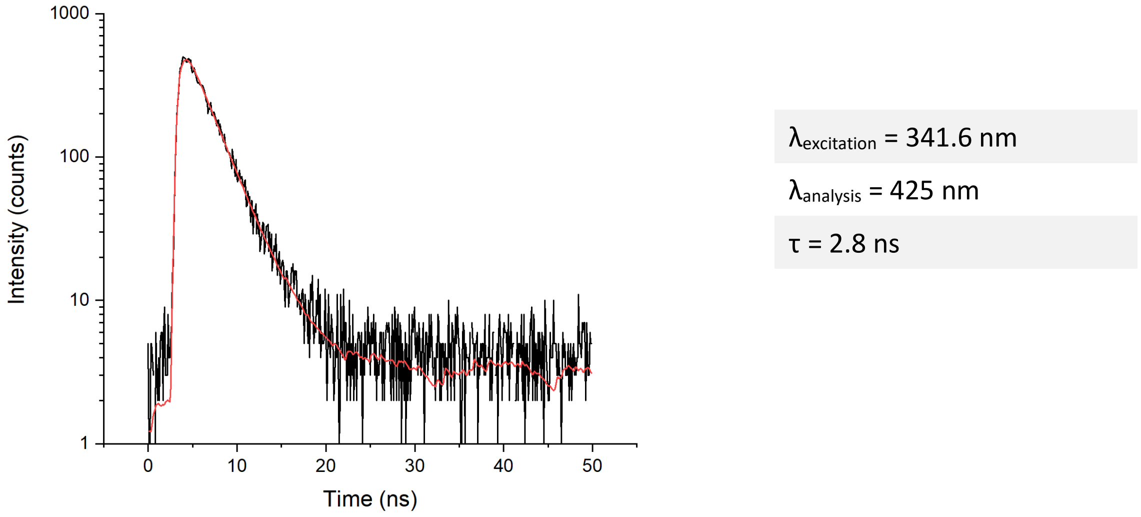

*Figure S61: Time-resolved emission decay (excitation at 341.6 nm, analysis at 425 nm) of **6c** 2·10^{-5} M in MeCN solution measured by TC-SPC (τ = 2.8 ns, from deconvolution and single-exponential fitting, X^2 = 1.281).*

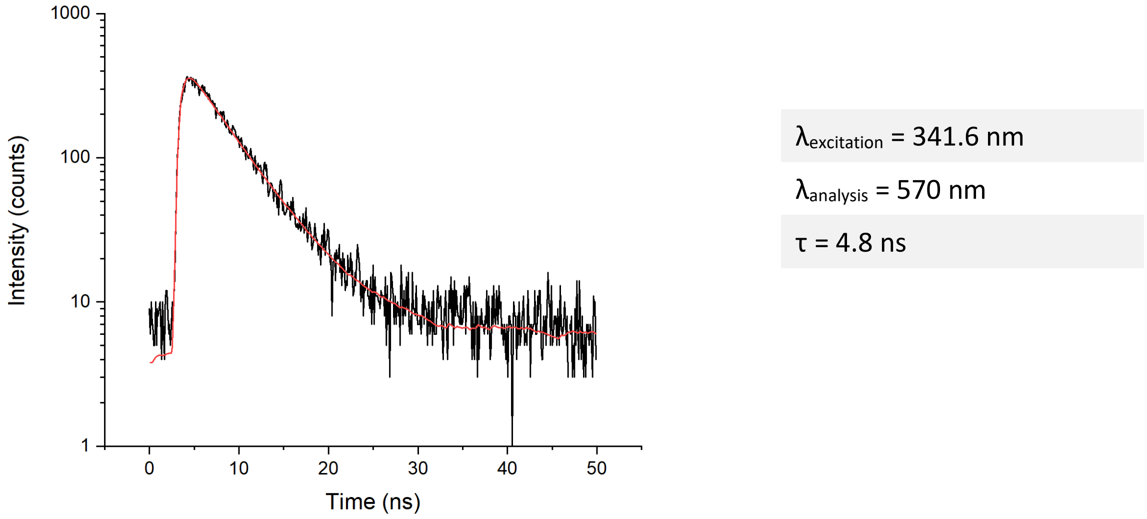

*Figure S62: Time-resolved emission decay (excitation at 341.6 nm, analysis at 570 nm) of **6c** 2·10^{-5} M in MeCN solution measured by TC-SPC (τ = 4.8 ns, from deconvolution and single-exponential fitting, X^2 = 1.207).*

4-(2,7-dimethoxy-9,10-dimethyl-9,10-dihydroacridin-9-yl)benzonitrile (6d)

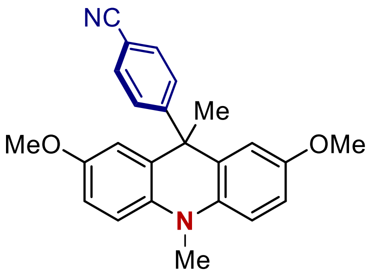

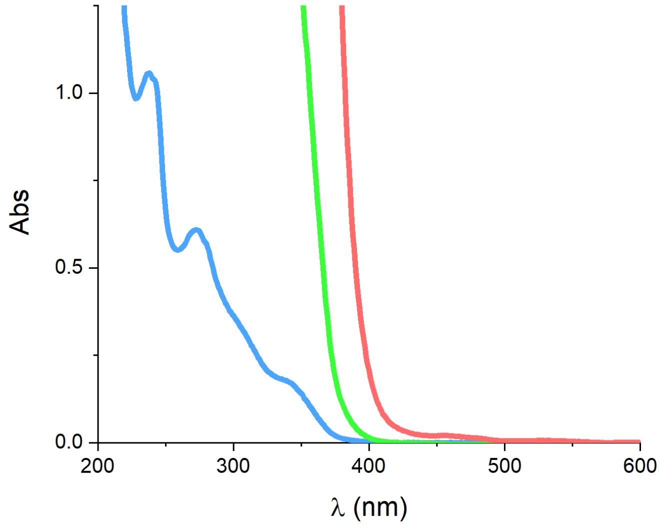

	Concentration (mmol/mL)
red	$5 \cdot 10^{-3}$
green	$5 \cdot 10^{-4}$
blue	$5 \cdot 10^{-5}$
λ_{peak}	275 nm
$\lambda_{shoulder}$	340 nm

*Figure S63: UV-Vis absorption of **6d** at different concentrations. 5·10 M (red trace); $5 \cdot 10^{-4}$ M (green trace); $5 \cdot 10^{-5}$ M (blue trace). Recorded in MeCN in quartz cuvettes (1 cm optical path).*

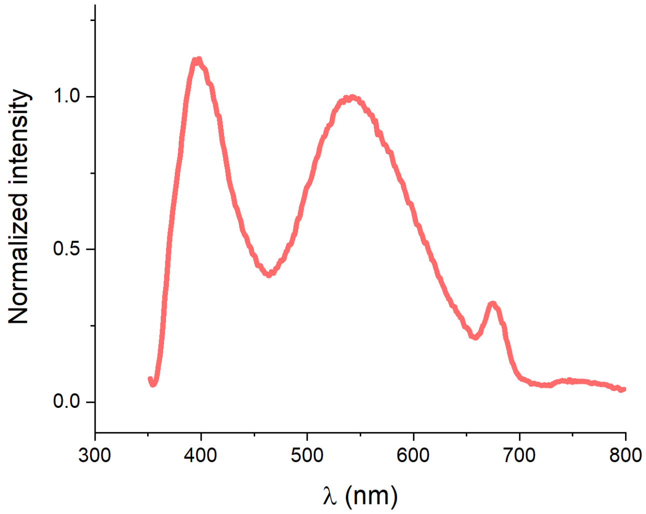

$\lambda_{excitation}$ = 340nm

	Solvent	Peak (nm)
red	MeCN	543

*Figure S64: Normalized fluorescence emission of **6d** $2 \cdot 10^{-5}$ M in MeCN.*

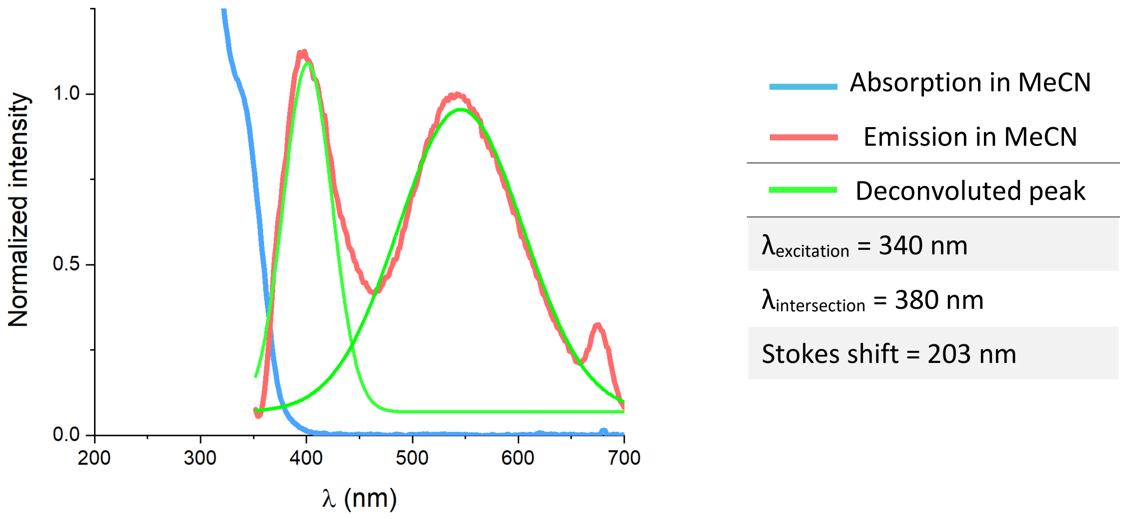

*Figure S65: Normalized optical absorption spectrum (blue trace) and emission spectra (red trace) of **6d**. The emission peaks were deconvoluted using the « peak analyser » function in OriginPro. The intersection wavelength was calculated using the deconvoluted trace.*

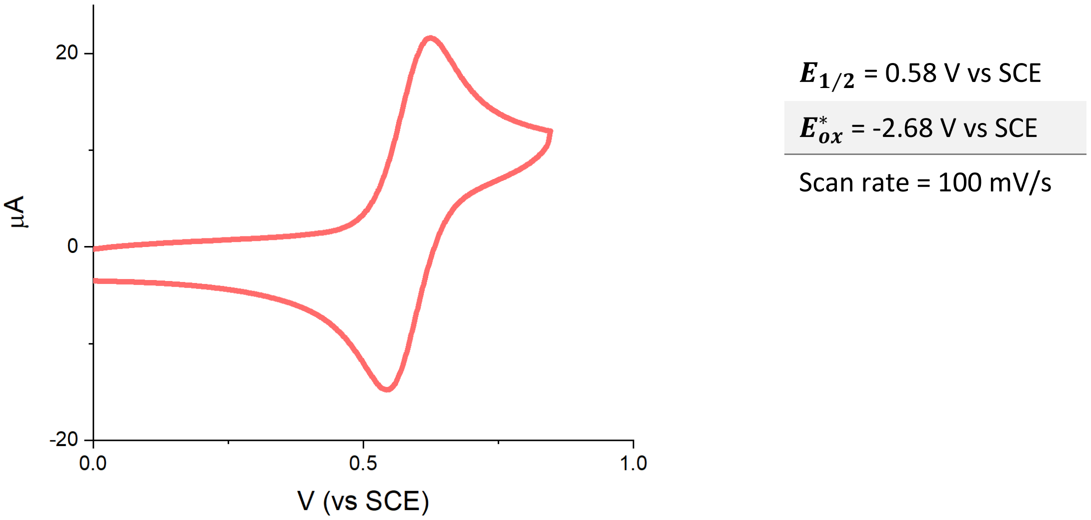

*Figure S66: Cyclic voltammograms of **6d** 10^{-3} M. Recorded with GC as the working electrode in MeCN with 0.1 M $[Bu_4N][PF_6]$ as the supporting electrolyte at 0.1 V/s scan rate.*

1-(azidomethyl)-3-methoxybenzene (22)

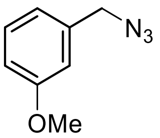

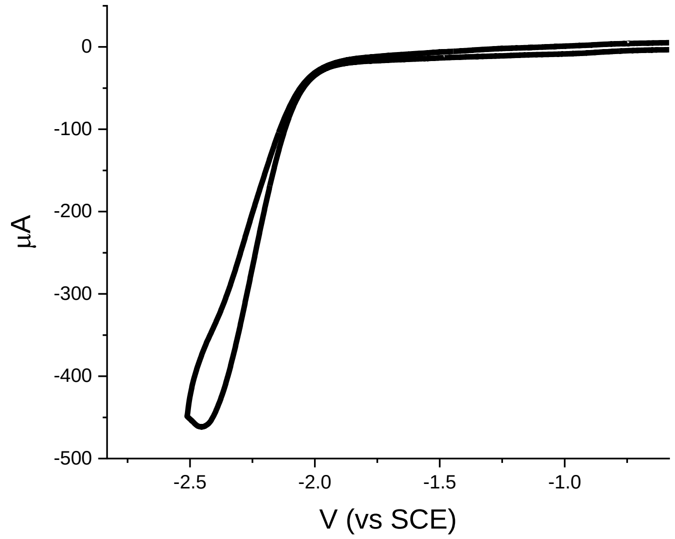

$\boldsymbol{E_p}$ = -2.45 V vs SCE

Scan rate = 100 mV/s

*Figure S67: Cyclic voltammogram of **22** 5·10^{-3} M. Recorded with GC as the working electrode in MeCN with 0.1 M [Bu_4N][PF_6] as the supporting electrolyte at 0.1 V/s scan rate.*

1-(azidomethyl)-4-(tert-butyl)benzene (23)

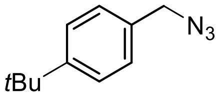

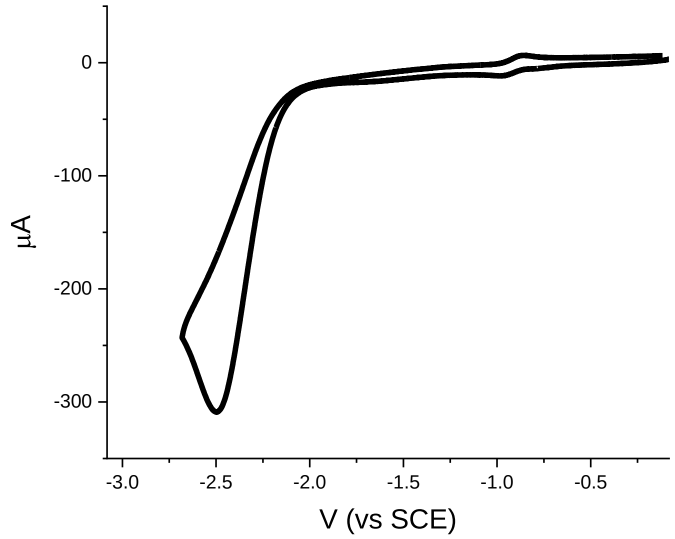

$\boldsymbol{E_p}$ = -2.50 V vs SCE

Scan rate = 100 mV/s

*Figure S68: Cyclic voltammogram of **23** 5·10^{-3} M. Recorded with GC as the working electrode in MeCN with 0.1 M [Bu_4N][PF_6] as the supporting electrolyte at 0.1 V/s scan rate.*

4-cyanophenyl diethyl phosphate (24)

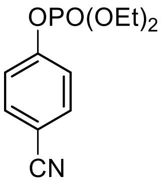

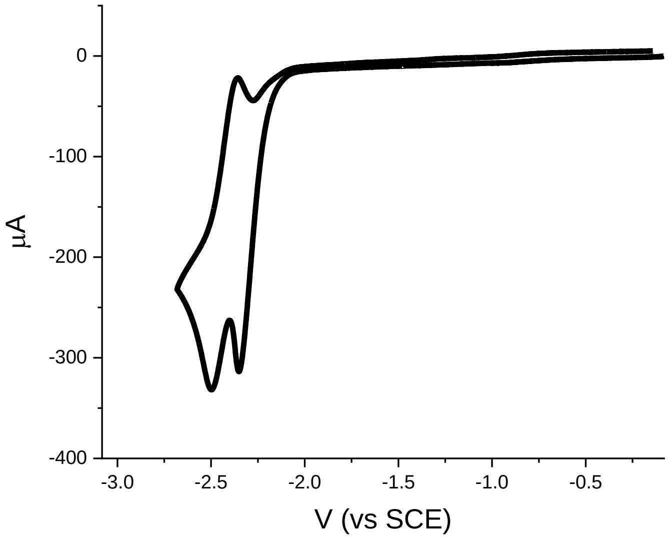

E_p = -2.35 V vs SCE

Scan rate = 100 mV/s

*Figure S69: Cyclic voltammogram of **24** 5·10^{-3} M. Recorded with GC as the working electrode in MeCN with 0.1 M [Bu$_4$N][PF$_6$] as the supporting electrolyte at 0.1 V/s scan rate.*

N,N,N-trimethylnaphthalen-1-aminium trifluoromethanesulfonate (27)

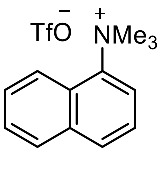

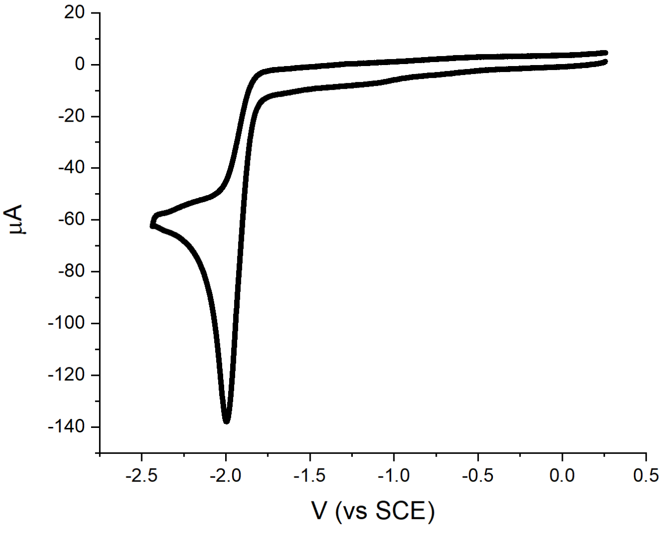

E_p = -2.00 V vs SCE

Scan rate = 100 mV/s

*Figure S70: Cyclic voltammogram of **27** 5·10^{-3} M. Recorded with GC as the working electrode in MeCN with 0.1 M [Bu$_4$N][PF$_6$] as the supporting electrolyte at 0.1 V/s scan rate.*

N,N,N-trimethyl-4-phenoxybenzenaminium trifluoromethanesulfonate (29)

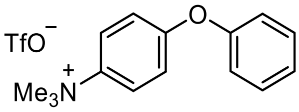

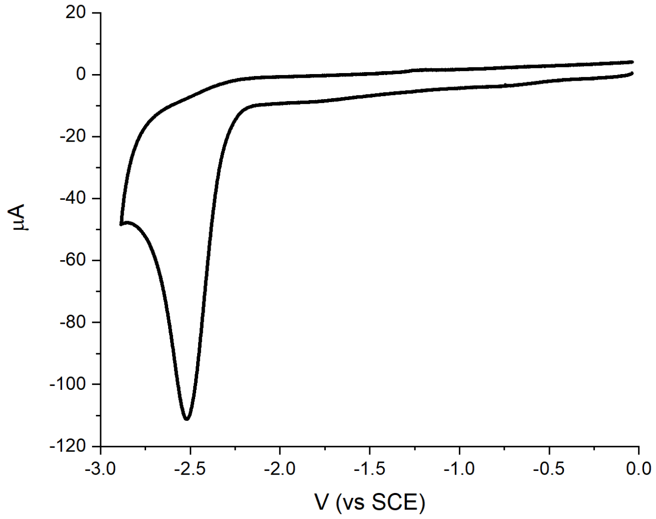

$\boldsymbol{E_p}$ = -2.52 V vs SCE

Scan rate = 100 mV/s

*Figure S71: Cyclic voltammogram of **29** 5·10^{-3} M. Recorded with GC as the working electrode in MeCN with 0.1 M [Bu$_4$N][PF$_6$] as the supporting electrolyte at 0.1 V/s scan rate.*

N,N,N-trimethyl-1-(naphthalen-2-yl)methanaminium trifluoromethanesulfonate (30)

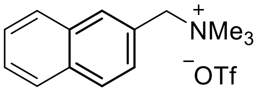

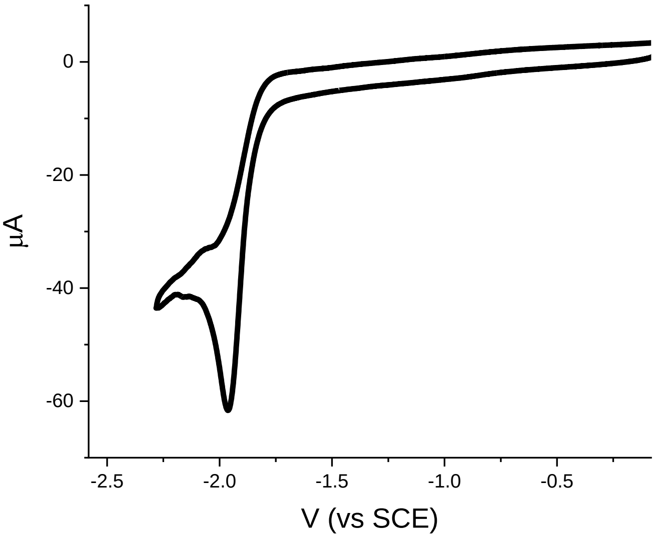

$\boldsymbol{E_p}$ = -1.97 V vs SCE

Scan rate = 100 mV/s

*Figure S72: Cyclic voltammogram of **30** 5·10^{-3} M. Recorded with GC as the working electrode in MeCN with 0.1 M [Bu$_4$N][PF$_6$] as the supporting electrolyte at 0.1 V/s scan rate.*

4,4'-methylenebis(N,N,N-trimethylbenzenaminium) trifluoromethanesulfonate (31)

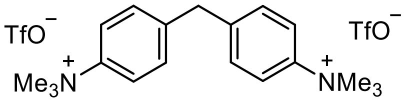

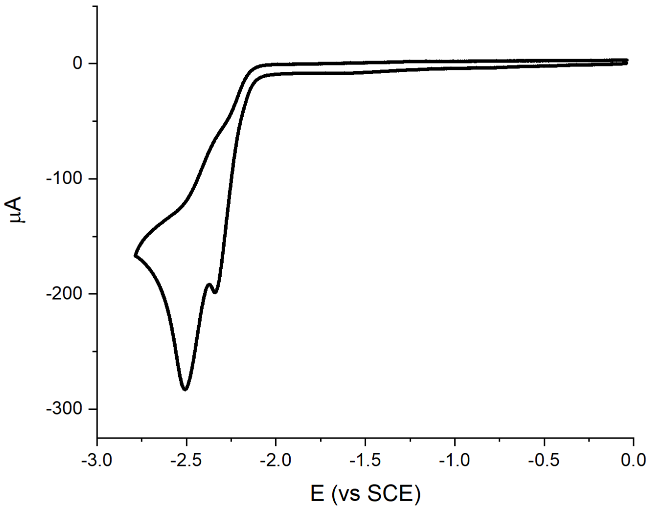

$E_{p,1}$ = -2.34 V vs SCE

$E_{p,2}$ = -2.51 V vs SCE

Scan rate = 100 mV/s

Figure S73: Cyclic voltammogram of ***31*** *5·10^{-3} M. Recorded with GC as the working electrode in MeCN with 0.1 M [Bu$_4$N][PF$_6$] as the supporting electrolyte at 0.1 V/s scan rate.*

(S)-1-methoxy-N,N,N-trimethyl-1-oxo-3-phenylpropan-2-aminium trifluoromethanesulfonate (32)

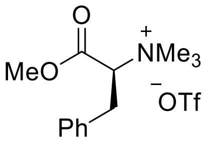

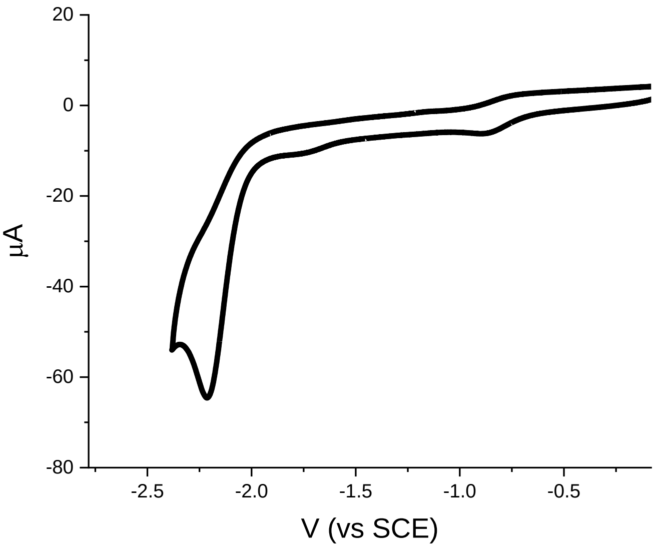

E_p = -2.22 V vs SCE

Scan rate = 100 mV/s

Figure S74: Cyclic voltammogram of ***32*** *5·10^{-3} M. Recorded with GC as the working electrode in MeCN with 0.1 M [Bu$_4$N][PF$_6$] as the supporting electrolyte at 0.1 V/s scan rate.*

For the reduction potential of the other substrates see:

Org. Lett., **2022**, 24, 4170-4175

Chem. Commun., **2020**,56, 5026-5029

J. Am. Chem. Soc. **2010**, 132, 17199–17210

J. Am. Chem. Soc. **2004**, 126, 16051-16057

J. Am. Chem. Soc. **2020**, 142, 2093-2099

Oxidation reversibility analysis

The oxidation reversibility of PC **5a** was studied performing several cyclic voltammetry scans, with different scan rates.

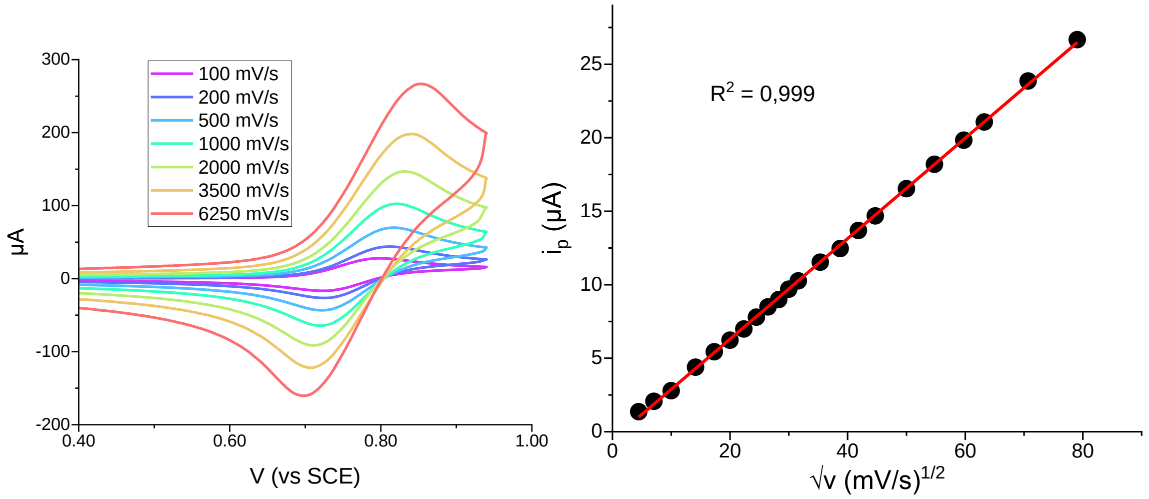

Figure S75: Left: cyclic voltammograms of ***5a*** *10^{-3} M at different scan rates. Recorded with GC as the working electrode in MeCN with 0.1 M $[Bu_4N][PF_6]$ as the supporting electrolyte; only selected scans are represented for clarity. Right: linear interpolation of the anodic peak current versus the square root of the scan rate.*

scan rate (mV/s)	Ep*a* (V vs SCE)	Ep*c* (V vs SCE)	ΔEp (mV)	$E_{1/2}$ (V)	ip*a* (μA)	ip*c* (μA)	ip*a*/ ip*c*
20	0.803	0.728	75	0.77	8.17	8.28	0.99
30	0.800	0.726	74	0.76	9.43	10.09	0.93
40	0.801	0.728	73	0.76	12.73	13.65	0.93
50	0.800	0.728	72	0.76	13.66	14.61	0.93
60	0.800	0.725	75	0.76	15.32	16.33	0.94
70	0.798	0.726	72	0.76	16.53	17.48	0.95
80	0.798	0.724	74	0.76	18.17	19.38	0.94
90	0.798	0.725	73	0.76	20.10	20.28	0.99
100	0.795	0.721	74	0.76	20.89	21.08	0.99
200	0.809	0.724	85	0.77	31.68	33.84	0.94
300	0.807	0.725	82	0.77	39.40	43.00	0.92
400	0.814	0.721	93	0.77	45.00	48.48	0.93
500	0.816	0.719	97	0.77	51.50	54.41	0.95
600	0.815	0.721	94	0.77	57.92	60.71	0.95
700	0.818	0.717	101	0.77	63.41	66.52	0.95
800	0.817	0.719	98	0.77	66.88	71.06	0.94
900	0.820	0.718	102	0.77	72.72	77.51	0.94
1000	0.818	0.718	100	0.77	74.63	79.33	0.94
1250	0.822	0.714	108	0.77	84.58	89.92	0.94
1500	0.828	0.709	119	0.77	91.37	96.35	0.95
1750	0.827	0.711	116	0.77	102.85	104.95	0.98
2000	0.828	0.709	119	0.77	106.82	114.43	0.93
2500	0.827	0.710	117	0.77	122.38	127.91	0.96

3000	0.833	0.707	126	0.77	134.92	140.59	0.96
3571	0.841	0.705	136	0.77	144.68	152.76	0.95
4000	0.842	0.705	137	0.77	152.09	158.90	0.96
5000	0.845	0.699	146	0.77	164.22	183.45	0.90
6250	0.850	0.695	155	0.77	182.63	206.10	0.89
7143	0.857	0.692	165	0.77	202.42	227.28	0.89
8333	0.859	0.691	168	0.78	222.20	238.20	0.93

Photoredox properties comparison

PCs 4a-d

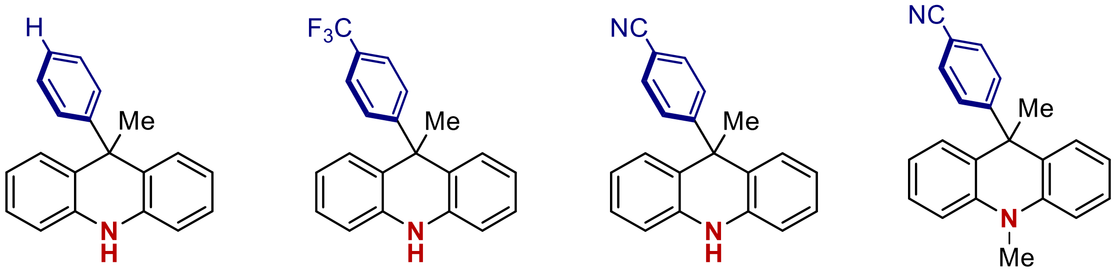

4a **4b** **4c** **4d**

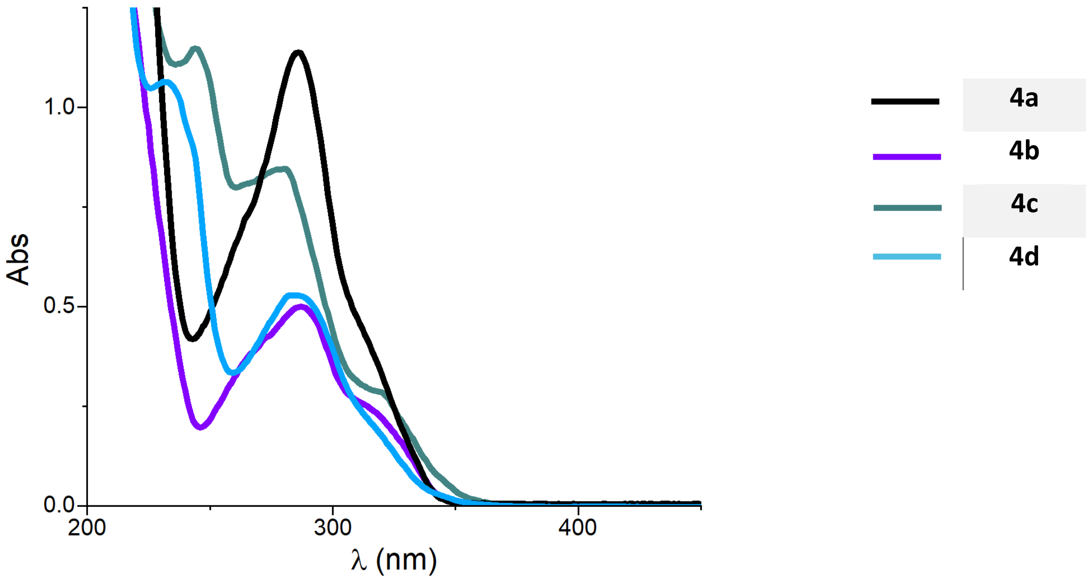

*Figure S76: Superimposed absorption spectra of PCs **4a** (black trace), **4b** (violet trace), **4c** (green trace) and **4d** (blue trace) 5·10^{-5} M in MeCN.*

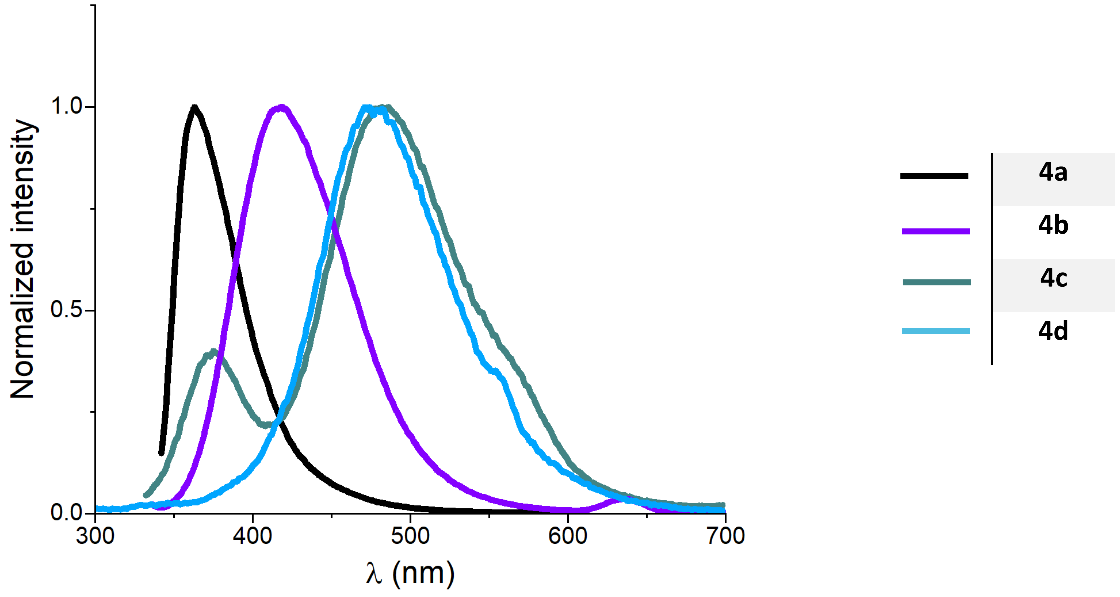

*Figure S77: Superimposed normalized emission spectra of PCs **4a** (black trace), **4b** (violet trace), **4c** (green trace) and **4d** (blue trace) 2·10^{-5} M in MeCN.*

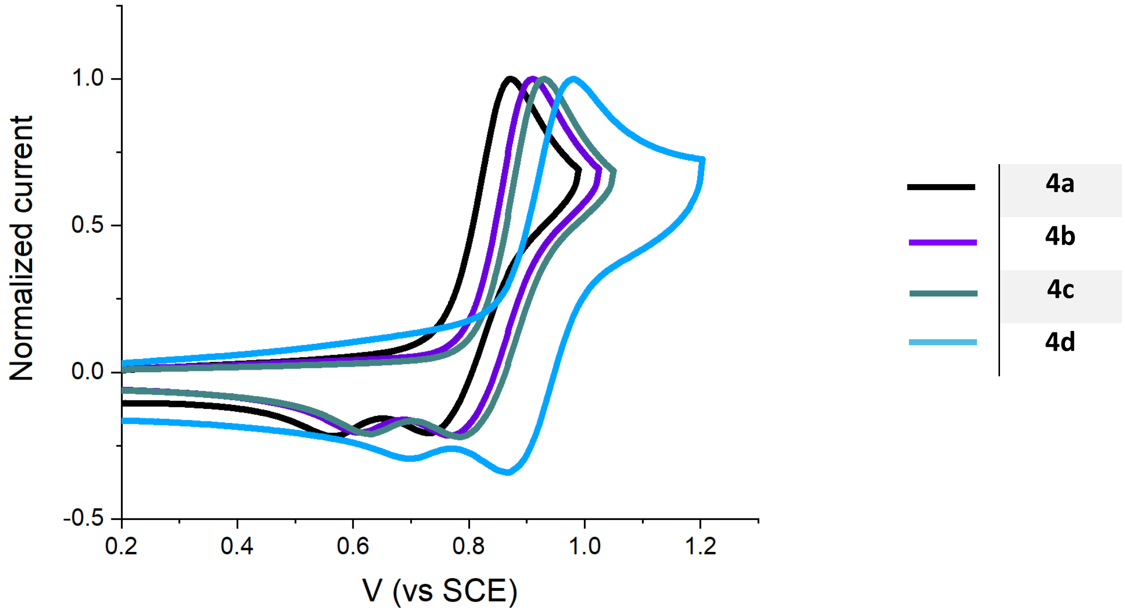

*Figure S78: Superimposed normalized cyclic voltammograms of PCs **4a** (black trace), **4b** (violet trace), **4c** (green trace) and **4d** (blue trace) 10^{-3} M. Recorded with GC as the working electrode in MeCN with 0.1 M [Bu_4N][PF_6] as the supporting electrolyte at 0.1 V/s scan rate.*

PCs 5a-e

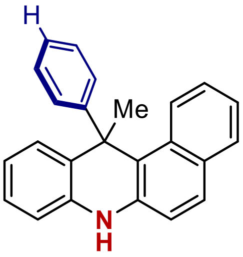

5a

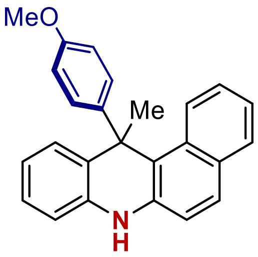

5b

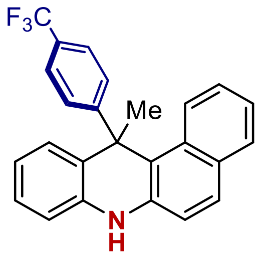

5c

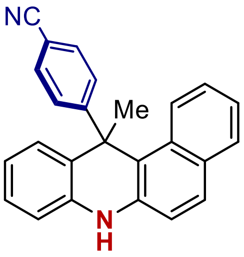

5d

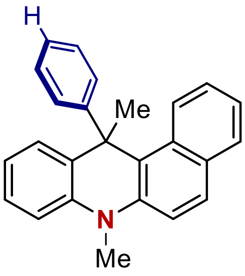

5e

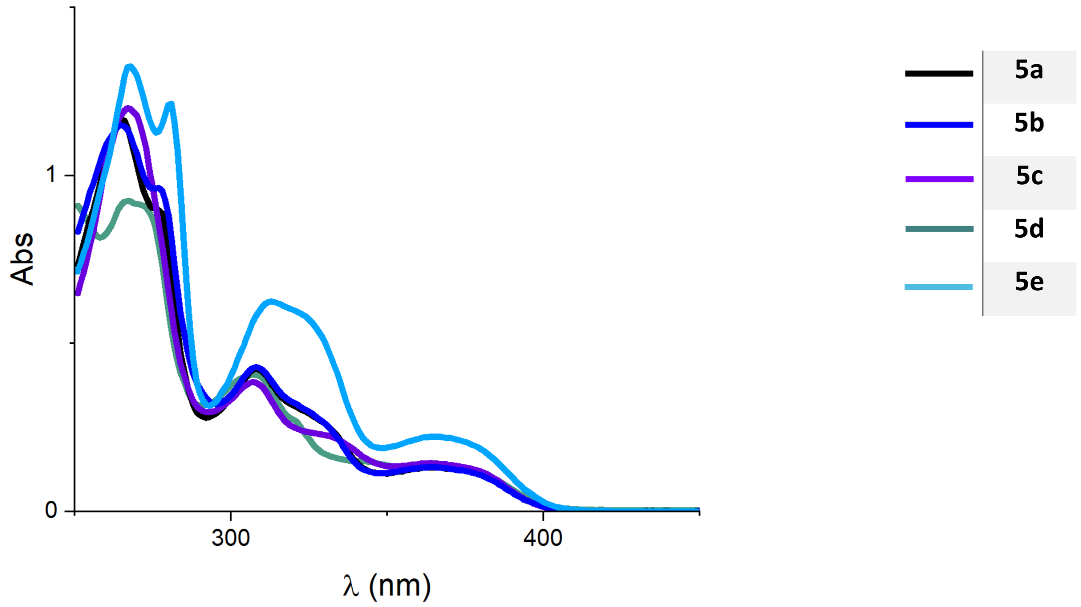

*Figure S79: Superimposed absorption spectra of PCs **5a** (black trace), **5b** (dark blue trace), **5c** (violet trace), **5d** (green trace) and **5e** (light blue trace) $5{\cdot}10^{-5}$ M in MeCN*

.

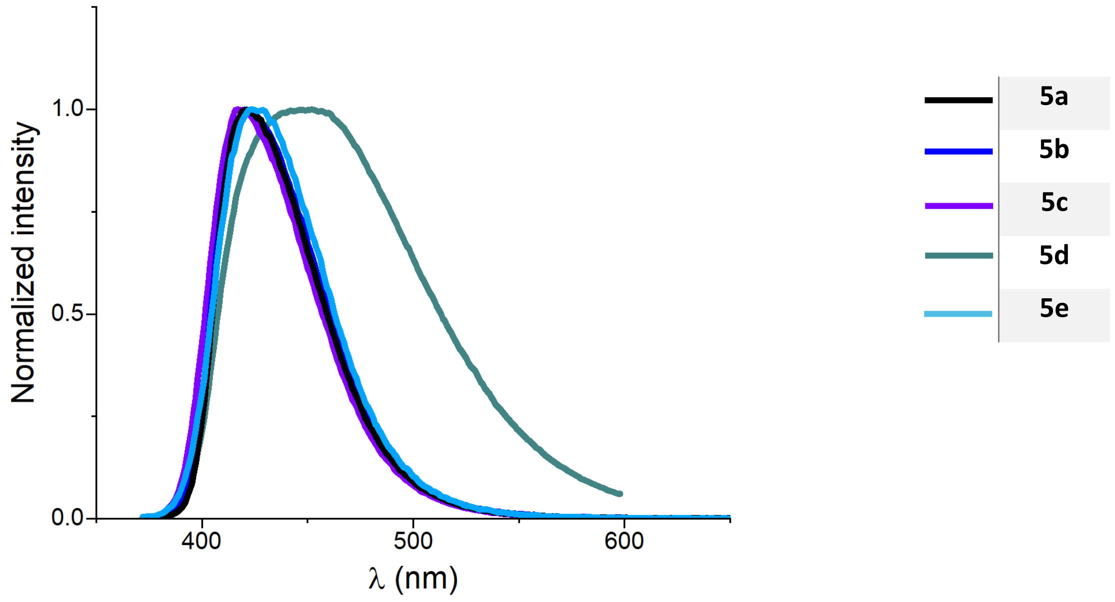

Figure S80: Superimposed normalized emission spectra of PCs ***5a*** *(black trace),* ***5b*** *(dark blue trace),* ***5c*** *(violet trace),* ***5d*** *(green trace) and* ***5e*** *(light blue trace) 2·10^{-5} M in MeCN.*

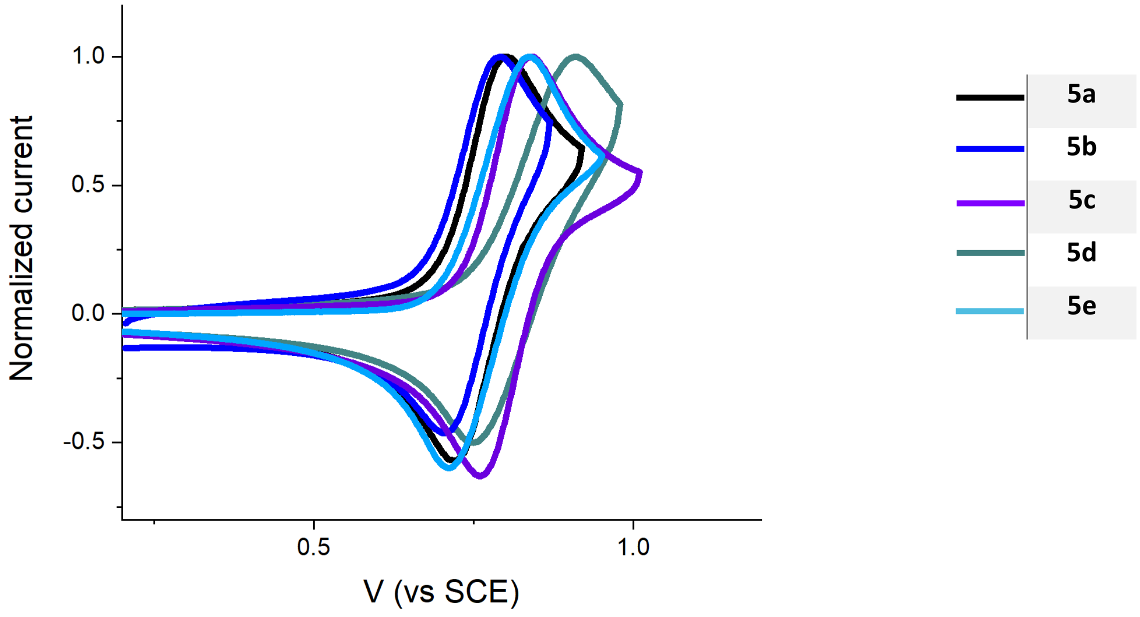

Figure S81: Superimposed normalized cyclic voltammograms of PCs ***5a*** *(black trace),* ***5b*** *(dark blue trace),* ***5c*** *(violet trace),* ***5d*** *(green trace) and* ***5e*** *(light blue trace) 10^{-3} M. Recorded with GC as the working electrode in MeCN with 0.1 M [Bu_4N][PF_6] as the supporting electrolyte at 0.1 V/s scan rate.*

PCs 6a-d

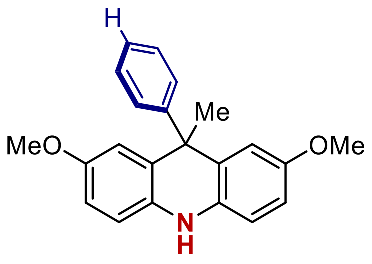

6a

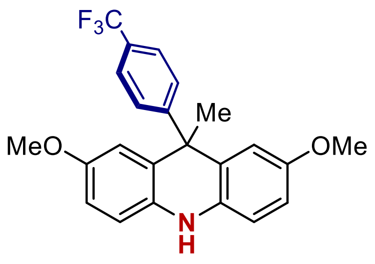

6b

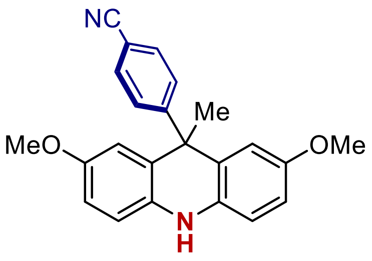

6c

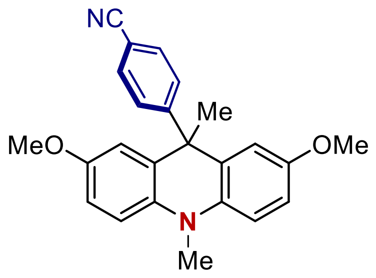

6d

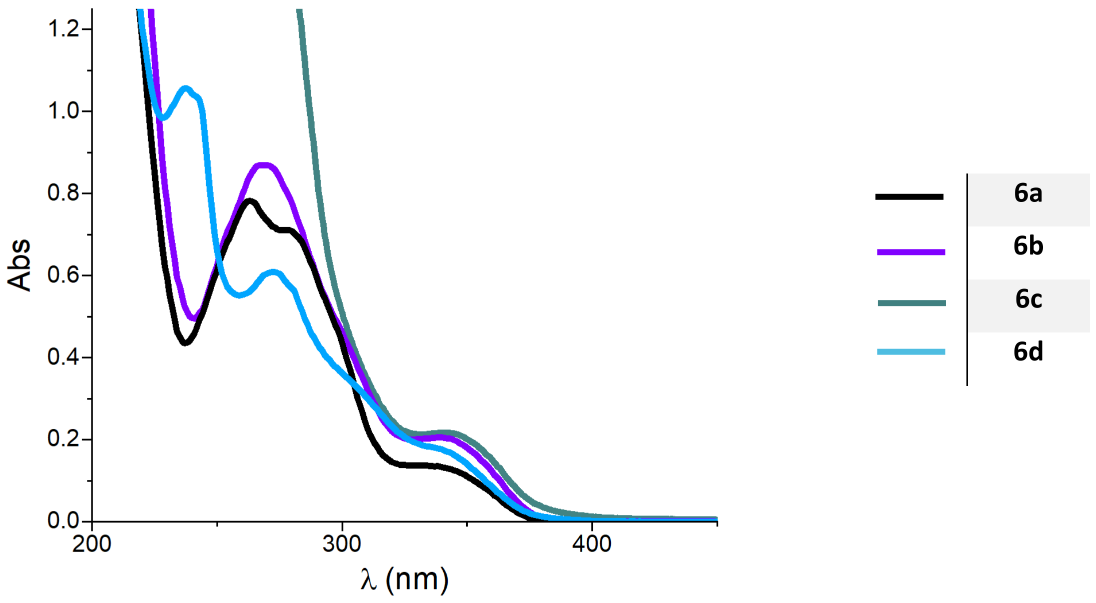

*Figure S82: Superimposed absorption spectra of PCs **6a** (black trace), **6b** (violet trace), **6c** (green trace) and **6d** (blue trace) $5{\cdot}10^{-5}$ M in MeCN.*

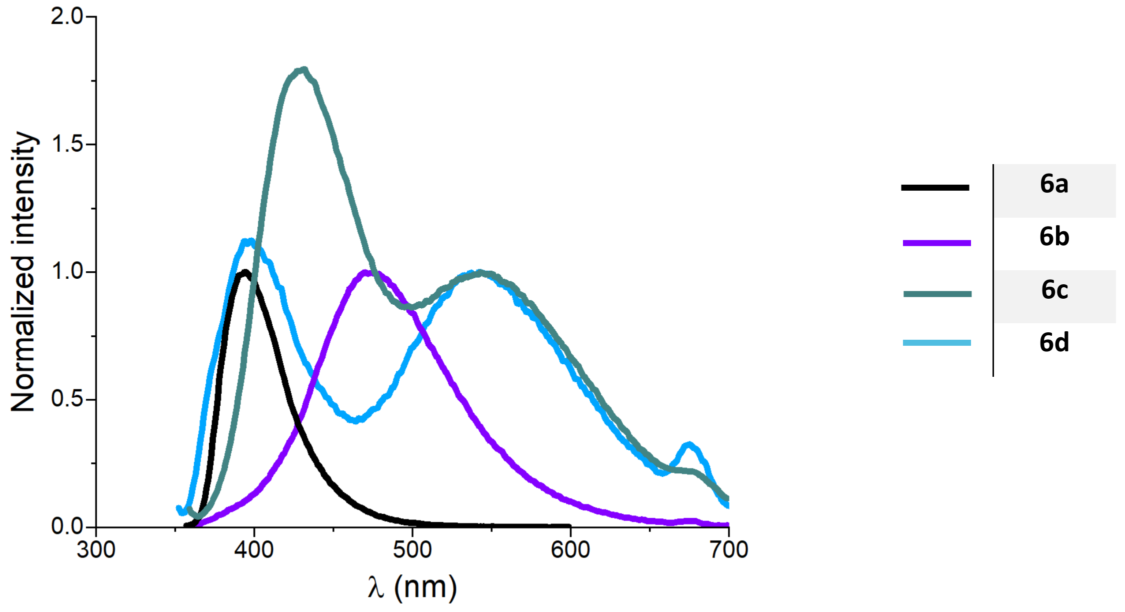

*Figure S83: Superimposed normalized emission spectra of PCs **6a** (black trace), **6b** (violet trace), **6c** (green trace) and **6d** (blue trace) 2·10^{-5} M in MeCN.*

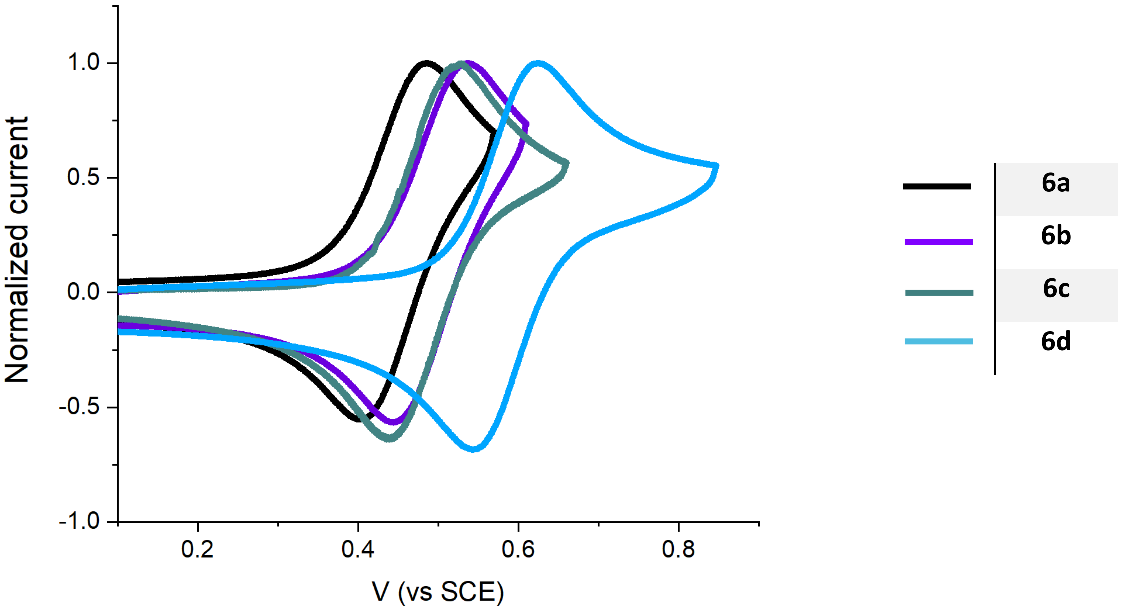

*Figure S84: Superimposed cyclic voltammograms of PCs **4a** (black trace), **4b** (violet trace), **4c** (green trace) and **4d** (blue trace) 10^{-3} M. Recorded with GC as the working electrode in MeCN with 0.1 M [Bu_4N][PF_6] as the supporting electrolyte at 0.1 V/s scan rate.*

G. Evaluation of the photoredox performances

Reductive dehalogenation of *p*-bromobenzonitrile

General Procedure B

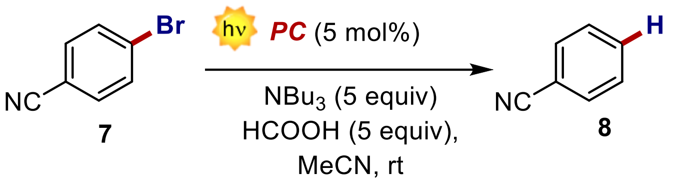

The procedure was adapted from the literature.[16] A 4 mL vial equipped with a septum was charged with a magnetic stirbar, 4-bromobenzonitrile **7** (18.2 mg, 0.1 mmol) and PC (5 mol%) and degassed with Ar. 1 mL of already degassed MeCN was added. Bu_3N (0.12 mL, 5 equiv) and HCOOH (19 µL, 5 equiv) were added via syringe and the reaction mixture was purged with Ar for 1 minute. The vial was irradiated for 6 hours with a 427 nm Kessil lamp set at 25% of its maximum output power.

GC-FID yield: First, a calibration curve of the instrument was obtained for both 4-bromobenzonitrile **7** and benzonitrile **8** with 1,3,5-trimethoxybenzene as the internal standard using the GC-FID method described below. Before irradiation, a sample of 100 µL was taken from the reaction mixture and diluted with 800 µL of MeCN. 100 µL of a 0.05 M solution of 1,3,5-trimethoxybenzene in MeCN were then added as the internal standard. The resulting solution was analysed by GC-FID and the initial concentration of 4-bromobenzonitrile **7** was calculated using the calibration curve. To calculate the GC-FID yield at a given time, a sample of 100 µL was taken from the reaction crude and diluted with 800 µL of MeCN. 100 µL of a 0.05 M solution of 1,3,5-trimethoxybenzene in MeCN were then added as the internal standard. The concentration of benzonitrile **8** was obtained by GC-FID analysis using the calibration curve. Yield was calculated from the initial concentration of 4-bromobenzonitrile **7**.

GC-FID method: Injector: 270°C, 1:100 split ratio, pressure 331.9 kPa, total flow 43.3 mL/min, column flow 0.40 mL/min, linear velocity 30.0 cm/s, purge flow 3.0 mL/min, He as carrier gas. Temperature program: 3 min at 40°C, 3°C/min until 100°C, 40°C/min until 280°C, 9 min at 280°C. Column: Equity 5, length 15.0 m, ID 0.10 mm, film thickness 0.10 µm. FID detector: 330 °C, H_2 flow 40.0 mL/min, Ar flow 400.0 mL/min, makeup flow 30.0 mL/min.

PC class	PC	Yield of 8 (%)
**9ADA**	**4a**	<5
	**4b**	<5
	**4c**	13
**12ADBA**	**5a**	84
	**5b**	78
	**5c**	83
	**5d**	41
**2,7OMeADA**	**6a**	35
	**6b**	31
	**6c**	20
**PTH**	**1**	52

*Table S2: Results of the screening of different PCs in the reductive debromination of 4-bromobenzonitrile **7** according to General procedure C.*

Kinetics

Two identical reaction vials for each PC were prepared according to General procedure B and irradiated with a 427 nm Kessil lamp set at 25% of its maximum output power. Before irradiation, a sample of 100 µL was taken from the reaction mixture and diluted with 800 µL of MeCN. 100 µL of a 0.05 M solution of 1,3,5-trimethoxybenzene in MeCN were then added as the internal standard. The resulting solution was analysed by GC-FID and the initial concentration of 4-bromobenzonitrile was calculated using the calibration curve. 100 µL aliquots were then sampled from the reaction vials (no more than 2 aliquots from each vial) at 15, 30, 45 and 60 minutes after the beginning of the irradiation. To each sample were added 800 µL of MeCN and 100 µL of a 0.05 M solution of 1,3,5-trimethoxybenzene in MeCN as the internal standard. The resulting solutions were analyzed by CG-FID to determine the product concentration via the calibration curve. Yields were calculated from the initial concentration of 4-bromobenzonitrile and plotted as a function of time to obtain the kinetic profiles.

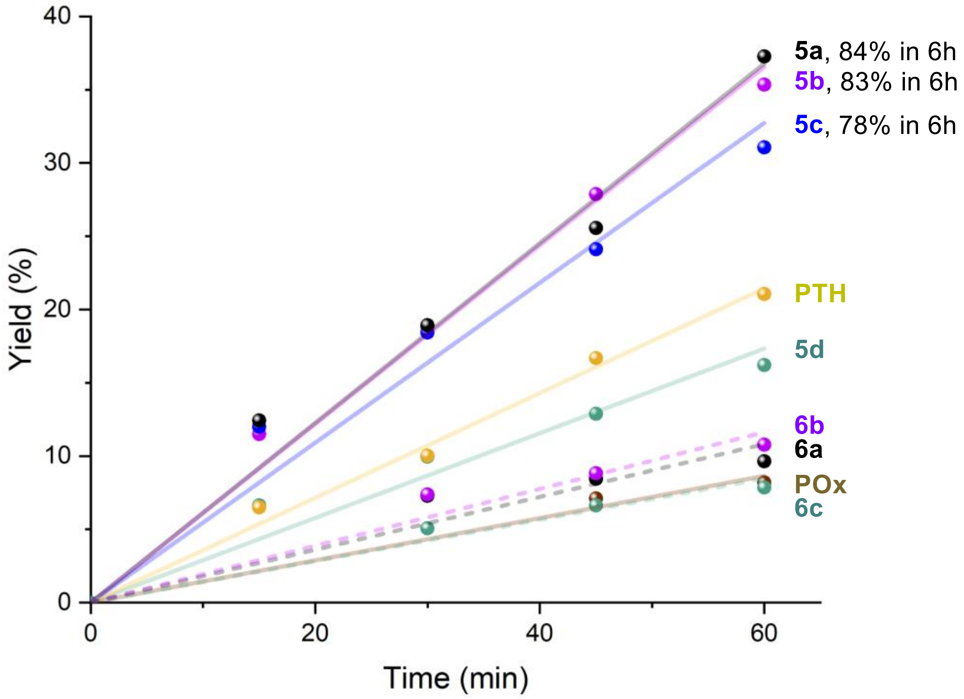

*Figure S85: Kinetic traces for the catalytic reductive debromination of 4-bromobenzonitrile with, **5a** (black dots), **5b** (blue dots), **5c** (violet dots), **5d** (green dots), **6a** (black dots), **6b** (violet dots), **6c** (green dots), **PTH** (yellow dots) and **POx** (brown dots) as the PCs. All the other investigated PCs provided less than 5% of product after 360 min.*

POx = 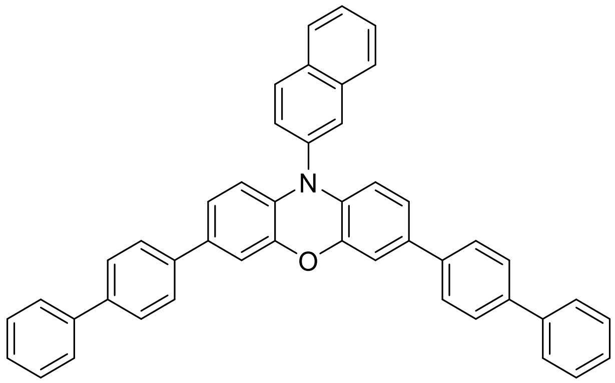

Control Experiments

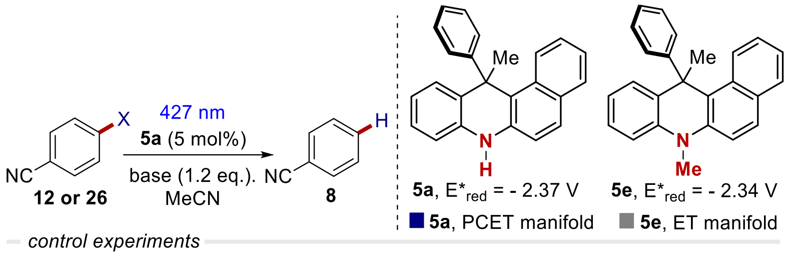

control experiments

		no variations	no light	no PC	no base	MeTBD/NBu_3 switch
PCET manifold	X = Cl	40 (36)	-	-	< 5	13
	X = -$\overset{+}{N}Me_3$	42 (38)	-	-	-	17
ET manifold	X = Cl	23	-	-	7	19
	X = -$\overset{+}{N}Me_3$	38	-	-	9	21

PCET manifold (base = MeTBD) and comparison with the classical ET process (base = NBu_3 1.2 eq. and HCO_2H 1.2 eq.). All the reported values refer to NMR yield (%). The isolated yields are reported in parentesis.

Scheme S1: Control experiments for the PCET and ET manifold.

Observations. i) The reaction without light or without the PC does not proceed at all with complete recovery of the starting material; ii) the reaction without the base furnished only traces of product under the PCET manifold, while showed low yields under ET manifold; iii) when switching the base under both manifolds (PCET or ET) the yields drop significantly. These observations are in line with our discussion within the main text. Indeed, it is expected that the stronger the base, the more negative the oxidation potential, and the higher the reducing power of the PC*/B couple is.

The control experiments not only prove the effectiveness of the PET and PCET manifold, but also support the operation of the two alternative pathways depending by the type of PC and base used. Further, the experiments under the PCET manifold in presence of NBu_3 (theoretically the best condition for a XAT process) indicates that a XAT manifold is highly unlike to happen. It is worth nothing that, lacking the halogen atom, a XAT process for ammonium salt **26** is not possible.

Mechanism

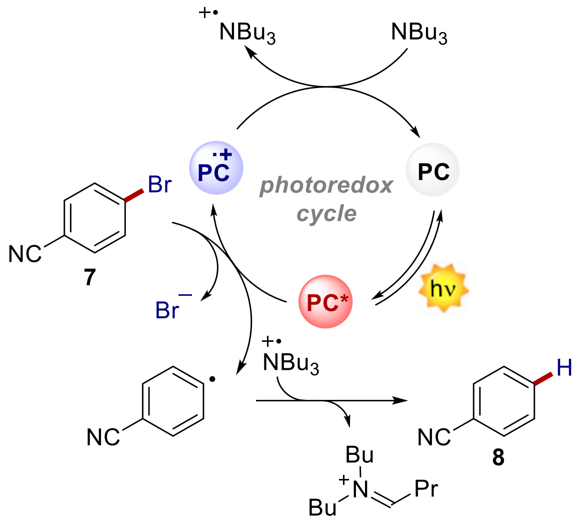

*Figure S86: Proposed mechanism for the photocatalytic dehalogenation of **7**.*

Catalytic defluoroalkylation of 1,3-bis(trifluoromethyl)benzene

General procedure C

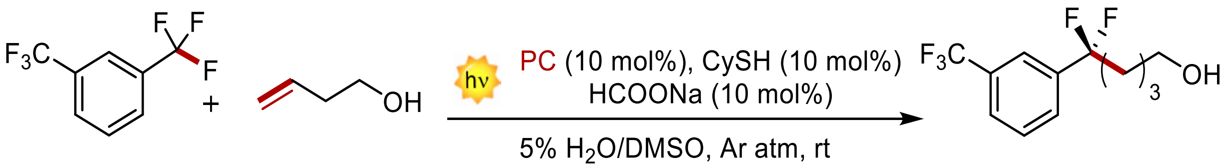

The procedure was adapted from the literature.[17] A 4 mL vial equipped with a septum was charged with a magnetic stirbar, HCOONa (30.6 mg, 3 equiv.) and PC (10 mol%) and degassed with Ar. 1.5 mL of the already degassed solvent mixture (5% H_2O in DMSO) were added via syringe through the vial septum. 3-buten-1-ol (39 µL, 3 equiv.), cyclohexanethiol (2 µL, 0.1 equiv.) were added via syringe and the reaction mixture was purged with Ar for 1 minute. Then 1,3-bis(trifluoromethyl)benzene (23.4 µL, 0.15 mmol) was added via syringe. The vial was irradiated with 440 nm LEDs.

i) ^{19}F-NMR yield: 4,4'-difluorobenzophenone (32.4 mg, 0.15 mmol) was added to the reaction mixture as internal standard.

ii) Product purification: The reaction mixture was diluted with H_2O and extracted with EtOAc. The organic layers were combined, washed with brine, and dried with $MgSO_4$. After concentration, the product was purified by column chromatography (eluent: 95:5 Hex:EtOAc).

^{1}H NMR (300 MHz, $CDCl_3$): δ 7.74 (s, 1H), 7.70 (d, J = 7.8 Hz, 1H), 7.66 (d, J = 7.8 Hz, 1H), 7.56 (t, J = 7.8 Hz, 1H), 3.52 (t, J = 6.6 Hz, 2H), 2.19 – 2.08 (m, 2H), 1.75 (m, J = 6.8 Hz, 2H), 1.50 – 1.40 (m, 4H), 1.36 (m, J = 7.6 Hz, 2H) ppm. **^{19}F-NMR (200 MHz, $CDCl_3$):** δ -63.2 (s, 3F), -96.6 (t, J = 16.5 Hz, 2F) ppm. These data match with the previously reported in literature.[17]

Kinetics

A vial for each PC was prepared according to General procedure C and irradiated with 440 nm LEDs. 100 µL aliquots were sampled from the reaction vial at 2, 5, 7, 10 and 20 minutes after the beginning of the irradiation. To each sample were added 400 µL of $CDCl_3$ and 100 µL of a 0.1 M solution of 4,4'-difluorobenzophenone in $CDCl_3$ as the internal standard. The resulting solutions were analyzed by ^{19}F-NMR to determine the product yield. Yields were plotted as a function of time to obtain the kinetic profiles.

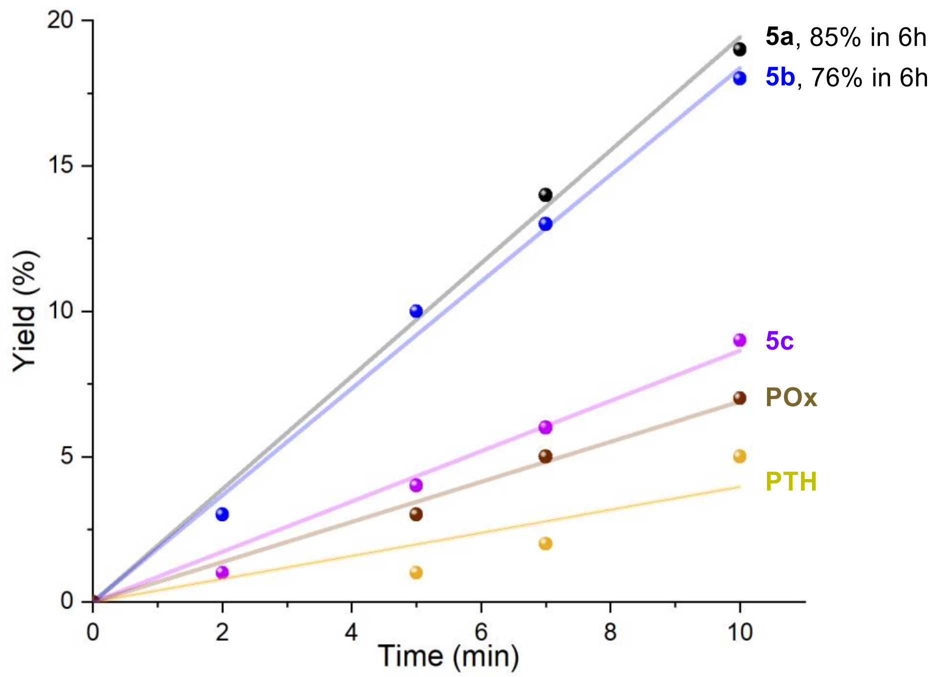

*Figure S87: Kinetic traces for the catalytic defluoroalkylation of 1,3-bis(trifluoromethyl)benzene with **5a** (black dots), **5b** (blue dots), **5c** (violet dots), **PTH** (yellow dots) and **POx** (brown dots) as the PCs. All the other investigated PCs provided less than 5% of product after 60 min.*

Mechanism

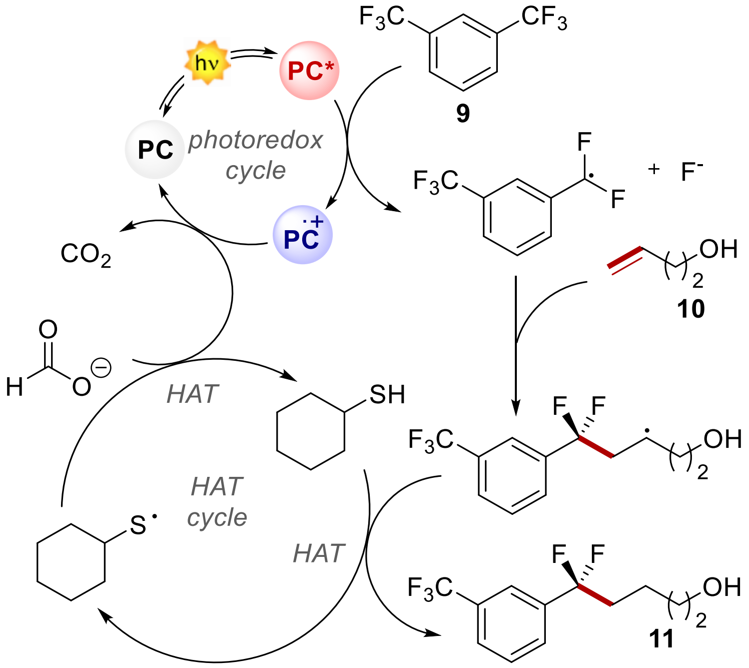

*Figure S88: Proposed mechanism for the photocatalytic defluoroalkylation of **9**.*

Reductive dechlorination of 4-chlorobenzonitrile via PCET - effect of base

General procedure D

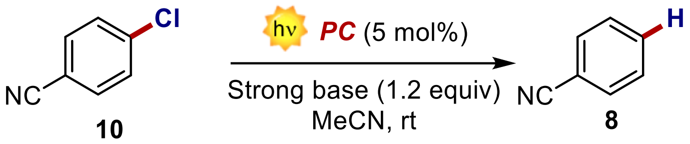

A 4 mL vial equipped with a septum was charged with a magnetic stirbar, 4-chlorobenzonitrile **10** (18.2 mg, 0.1 mmol) and PC (5 mol%) and degassed with Ar. If solid, the selected base (1.2 mmol, 1.2 equiv) was added at this stage. 1 mL of already degassed MeCN was added. If liquid, the selected base (1.2 mmol, 1.2 equiv) was added at this stage via syringe and the reaction mixture was purged with Ar for 1 minute. The vial was irradiated with a 427 nm Kessil lamp set at 25% of its maximum output power for 2h.

GC-FID yield: First, a calibration curve of the instrument was obtained for both 4-chlorobenzonitrile **10** and benzonitrile **8** with 1,3,5-trimethoxybenzene as the internal standard using the GC-FID method described below. Before irradiation, a sample of 100 µL was taken from the reaction mixture and diluted with 800 µL of MeCN. 100 µL of a 0.05 M solution of 1,3,5-trimethoxybenzene in MeCN were then added as the internal standard. The resulting solution was analysed by GC-FID and the initial concentration of 4-chlorobenzonitrile **10** was calculated using the calibration curve. To calculate the GC-FID yield at the end of the reaction, a sample of 100 µL was taken from the reaction crude and diluted with 800 µL of MeCN. 100 µL of a 0.05 M solution of 1,3,5-trimethoxybenzene in MeCN were then added as the internal standard. The concentration of benzonitrile **8** was obtained by GC-FID analysis using the calibration curve. Yield was calculated from the initial concentration of 4-chlorobenzonitrile **10**.

GC-FID method: Injector: 270°C, 1:100 split ratio, pressure 331.9 kPa, total flow 43.3 mL/min, column flow 0.40 mL/min, linear velocity 30.0 cm/s, purge flow 3.0 mL/min, He as carrier gas. Temperature program: 3 min at 40°C, 3°C/min until 100°C, 40°C/min until 280°C, 9 min at 280°C. Column: Equity 5, length 15.0 m, ID 0.10 mm, film thickness 0.10 µm. FID detector: 330 °C, H_2 flow 40.0 mL/min, Ar flow 400.0 mL/min, makeup flow 30.0 mL/min.

Entry	Base	Yield of 8 (%)
1	NBu_3	9
2	TMG	16
3	DBN	18
4	DBU	16
5	MeTBD	27
6	TBD	21

*Table S3: Results of the screening of different bases in the reductive dechlorination of 4-chlorobenzonitrile **10** via PCET according to General procedure E.*

Reductive dechlorination of 4-chlorobenzonitrile via PCET - effect of base and γ-terpinene

General procedure E

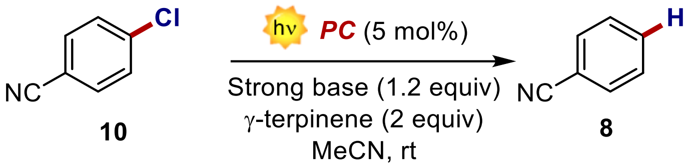

A 4 mL vial equipped with a septum was charged with a magnetic stirbar, 4-chlorobenzonitrile **10** (18.2 mg, 0.1 mmol) and PC (5 mol%) and degassed with Ar. If solid, the selected base (0.12 mmol, 1.2 equiv) was added at this stage. 1 mL of already degassed MeCN was added, followed by γ-terpinene (32 µL, 0.2 mmol, 2 equiv.). If liquid, the selected base (1.2 mmol, 1.2 equiv) was added at this stage via syringe and the reaction mixture was purged with Ar for 1 minute. The vial was irradiated with a 427 nm Kessil lamp set at 25% of its maximum output power for 2h.

GC-FID yield: First, a calibration curve of the instrument was obtained for both 4-chlorobenzonitrile **10** and benzonitrile **8** with 1,3,5-trimethoxybenzene as the internal standard using the GC-FID method described below. Before irradiation, a sample of 100 µL was taken from the reaction mixture and diluted with 800 µL of MeCN. 100 µL of a 0.05 M solution of 1,3,5-trimethoxybenzene in MeCN were then added as the internal standard. The resulting solution was analysed by GC-FID and the initial concentration of 4-chlorobenzonitrile **10** was calculated using the calibration curve. To calculate the GC-FID yield at the end of the reaction, a sample of 100 µL was taken from the reaction crude and diluted with 800 µL of MeCN. 100 µL of a 0.05 M solution of 1,3,5-trimethoxybenzene in MeCN were then added as the internal standard. The concentration of benzonitrile **8** was obtained by GC-FID analysis using the calibration curve. Yield was calculated from the initial concentration of 4-chlorobenzonitrile **10**.

GC-FID method: Injector: 270°C, 1:100 split ratio, pressure 331.9 kPa, total flow 43.3 mL/min, column flow 0.40 mL/min, linear velocity 30.0 cm/s, purge flow 3.0 mL/min, He as carrier gas. Temperature program: 3 min at 40°C, 3°C/min until 100°C, 40°C/min until 280°C, 9 min at 280°C. Column: Equity 5, length 15.0 m, ID 0.10 mm, film thickness 0.10 µm. FID detector: 330 °C, H_2 flow 40.0 mL/min, Ar flow 400.0 mL/min, makeup flow 30.0 mL/min.

Entry	*B*ase	Yield of 8 (%)
1	NEt_3	24
2	TMG	23
3	DBN	25
4	DBU	28
5	MeTBD	40
6	TBD	30
7[a)]	MeTBD	31
8[b)]	MeTBD	36
9[c)]	MeTBD	65

*Table S4: Results of the screening of different bases in the reductive dechlorination of 4-chlorobenzonitrile **10** via PCET in the presence of γ-terpinene according to General Procedure F. a) The reaction was carried out under air. b) The reaction was carried out in the presence of 20 mol% of DABCO. c) The reaction mixture was irradiated for 12 hours.*

Reduction of thermodynamically demanding substrates via PCET

General procedure F

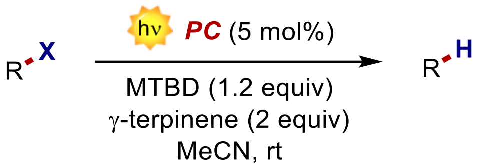

A 4 mL vial equipped with a septum was charged with a magnetic stirbar, the desired substrate **15**-**20** (0.1 mmol) and PC **5a** (5 mol%) and degassed with Ar. 1 mL of already degassed MeCN was added, followed by γ-terpinene (32 µL, 0.2 mmol, 2 equiv) and MeTBD (1.2 mmol, 1.2 equiv). The reaction mixture was purged with Ar for 1 minute. The vial was irradiated with a 427 nm Kessil lamp set at 25% of its maximum output power for the indicated amount of time.

Reduction of 17

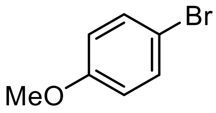

1-bromo-4-methoxy-benzene **17** (13 µL) was subjected to the reaction conditions described in general procedure F and irradiated for 48 hours. Yield of anisole was determined by ^{1}H NMR using CH_2Br_2 as the internal standard (49% yield). **^{1}H NMR (400 MHz, $CDCl_3$):** δ 7.31-7.27 (m, 2H), 6.96-6.89 (m, 3H), 3.80 (s, 3H) ppm. These data match with the previously reported in literature.[18]

Reduction of 18

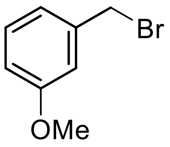

1-(bromomethyl)-3-methoxybenzene **18** (14 µL) was subjected to the reaction conditions described in general procedure F and irradiated for 24 hours. Yield of 1-methoxy-3-methyl-benzene was determined by ^{1}H NMR using CH_2Br_2 as the internal standard (51% yield). **^{1}H NMR (400 MHz, $CDCl_3$):** : δ 7.22-7.15 (m, 2H), 6.91-6.84 (m, 2H), 3.83 (s, 3H), 2.39 (s, 3H) ppm. These data match with the previously reported in literature.[19]

Reduction of 19

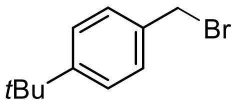

1-(bromomethyl)-4-(1,1-dimethylethyl)benzene **19** (18.4 µL) was subjected to the reaction conditions described in general procedure F and irradiated for 48 hours. Yield of 4-tert-butyltoluene was determined by ^{1}H NMR using CH_2Br_2 as the internal standard (53% yield). After solvent evaporation, the product was purified through column chromatography (eluent: 7:3 Hex:EtOAc). Product obtained: 7.4 mg (50% isolated yield). **^{1}H NMR (400 MHz, $CDCl_3$):** δ 7.32 (d, J = 8.2 Hz, 2H), 7.20 – 7.11 (m, 2H), 2.35 (s, 3H), 1.34 (s, 9H) ppm. These data match with the previously reported in literature.[20]

Reduction of 20

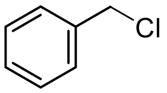

Benzyl chloride **20** (12 µL) was subjected to the reaction conditions described in general procedure F and irradiated for 48 hours. Conversion of **20** was determined by ^{1}H NMR using CH_2Br_2 as the internal standard (60% conversion).

Reduction of 21

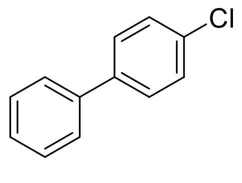

4'-biphenyl chloride **21** (18.8 mg) was subjected to the reaction conditions described in general procedure F and irradiated for 60 hours. Yield of 1,1’-biphenyl was determined by GC-FID using 1,3, 5-trimethoxybenzene as the internal standard (56% yield). After solvent evaporation, the product was purified through column chromatography (eluent: Hex). Product obtained: 7.9 mg (51% isolated yield).

GC-FID yield: First, a calibration curve of the instrument was obtained for both 4-chloro-1,1'-biphenyl **21** and commercially avaiable 1,1’-biphenyl with 1,3,5-trimethoxybenzene as the internal standard using the GC-FID method described below. Before irradiation, a sample of 100 µL was taken from the reaction mixture and diluted with 800 µL of MeCN. 100 µL of a 0.05 M solution of 1,3,5-trimethoxybenzene in MeCN were then added as the internal standard. The resulting solution was analysed by GC-FID and the initial concentration of 4-chloro-1,1'-biphenyl **21** was calculated using the calibration curve. To calculate the GC-FID yield at the end of the reaction, a sample of 100 µL was taken from the reaction crude and diluted with 800 µL of MeCN. 100 µL of a 0.05 M solution of 1,3,5-trimethoxybenzene in MeCN were then added as the internal standard. The concentration of 1,1’-biphenyl was obtained by GC-FID analysis using the calibration curve. Yield was calculated from the initial concentration of 4-chloro-1,1'-biphenyl **21**.

GC-FID method: Injector: 270°C, 1:100 split ratio, pressure 331.9 kPa, total flow 43.3 mL/min, column flow 0.40 mL/min, linear velocity 30.0 cm/s, purge flow 3.0 mL/min, He as carrier gas. Temperature program: 3 min at 40°C, 15°C/min until 280°C, 9 min at 280°C. Column: Equity 5, length 15.0 m, ID 0.10 mm, film thickness 0.10 µm. FID detector: 330 °C, H_2 flow 40.0 mL/min, Ar flow 400.0 mL/min, makeup flow 30.0 mL/min.

Reduction of 22

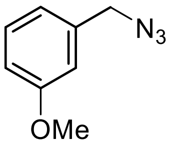

1-(azidomethyl)-3-methoxybenzene **22** (16.3 mg) was subjected to the reaction conditions described in general procedure F and irradiated for 24 hours. Yield of 1-methoxy-3-methylbenzene was determined by ^{1}H NMR using CH_2Br_2 as the internal standard (53% yield). After solvent evaporation, the product was purified through column chromatography (eluent: 9:1 Hex:EtOAc). Product obtained: 5.9 mg (48% isolated yield). **^{1}H NMR (400 MHz, $CDCl_3$):** : δ 7.22-7.15 (m, 2H), 6.91-6.84 (m, 2H), 3.83 (s, 3H), 2.39 (s, 3H) ppm. These data match with the previously reported in literature.[19]

Reduction of 23

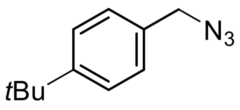

1-(azidomethyl)-4-(tert-butyl)benzene **23** (18.9 mg) was subjected to the reaction conditions described in general procedure F and irradiated for 24 hours. Yield of 4-tert-butyltoluene was determined by ^{1}H NMR using CH_2Br_2 as the internal standard (40% yield). **^{1}H NMR (400 MHz, $CDCl_3$):** δ 7.32 (d, J = 8.2 Hz, 2H), 7.20 – 7.11 (m, 2H), 2.35 (s, 3H), 1.34 (s, 9H) ppm. These data match with the previously reported in literature.[20]

Reduction of 24

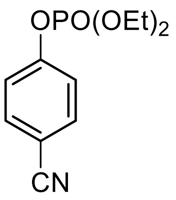

4-cyanophenyl diethyl phosphate **24** (25.5 mg) was subjected to the reaction conditions described in general procedure F and irradiated for 24 hours. Yield of benzonitrile was determined by GC-FID using 1,3,5-trimethoxybenzene as the internal standard (37% yield).

GC-FID yield: First, a calibration curve of the instrument was obtained for commercially available benzonitrile **8** with 1,3,5-trimethoxybenzene as the internal

standard using the GC-FID method described below. To calculate the GC-FID yield at the end of the reaction, a sample of 100 µL was taken from the reaction crude and diluted with 800 µL of MeCN. 100 µL of a 0.05 M solution of 1,3,5-trimethoxybenzene in MeCN were then added as the internal standard. The concentration of benzonitrile **8** was obtained by GC-FID analysis using the calibration curve.

GC-FID method: Injector: 270°C, 1:100 split ratio, pressure 331.9 kPa, total flow 43.3 mL/min, column flow 0.40 mL/min, linear velocity 30.0 cm/s, purge flow 3.0 mL/min, He as carrier gas. Temperature program: 3 min at 40°C, 3°C/min until 100°C, 40°C/min until 280°C, 9 min at 280°C. Column: Equity 5, length 15.0 m, ID 0.10 mm, film thickness 0.10 µm. FID detector: 330 °C, H_2 flow 40.0 mL/min, Ar flow 400.0 mL/min, makeup flow 30.0 mL/min.

Reduction of 25

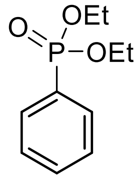

Diethyl phenylphosphonate **25** (21 µL) was subjected to the reaction conditions described in general procedure F and irradiated for 48 hours. The conversion was determined by ^{1}H NMR using CH_2Br_2 as the internal standard (11% conversion).

Reduction of 26

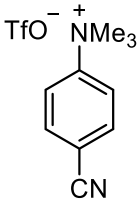

Cyano-*N,N,N*-trimethylbenzenaminium trifluoromethanesulfonate **26** (31.0 mg, 0.1 mmol) was subjected to the reaction conditions described in general procedure F and irradiated for 24 hours. Yield of benzonitrile was determined by GC-FID using 1,3,5-trimethoxybenzene as the internal standard (42% yield). After solvent evaporation, the product was purified through column chromatography (eluent: 7:3 Hex:EtOAc). Product obtained: 3.9 mg (38% isolated yield).

GC-FID yield: First, a calibration curve of the instrument was obtained for commercially available benzonitrile **8** with 1,3,5-trimethoxybenzene as the internal standard using the GC-FID method described below. To calculate the GC-FID yield at the end of the reaction, a sample of 100 µL was taken from the reaction crude and diluted with 800 µL of MeCN. 100 µL of a 0.05 M solution of 1,3,5-trimethoxybenzene in MeCN were then added as the internal standard. The concentration of benzonitrile **8** was obtained by GC-FID analysis using the calibration curve.

GC-FID method: Injector: 270°C, 1:100 split ratio, pressure 331.9 kPa, total flow 43.3 mL/min, column flow 0.40 mL/min, linear velocity 30.0 cm/s, purge flow 3.0 mL/min, He as carrier gas. Temperature program: 3 min at 40°C, 3°C/min until 100°C, 40°C/min until 280°C, 9 min at 280°C. Column: Equity 5, length 15.0 m, ID 0.10 mm, film thickness 0.10 µm. FID detector: 330 °C, H_2 flow 40.0 mL/min, Ar flow 400.0 mL/min, makeup flow 30.0 mL/min.

Reduction of 27

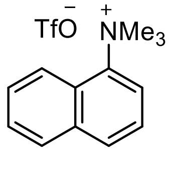

N,N,N-trimethylnaphthalen-1-aminium trifluoromethanesulfonate **27** (33,5 mg, 0.1 mmol) was subjected to the reaction conditions described in general procedure F and irradiated for 24 hours. Yield of naphthalene was determined by ^{1}H NMR using CH_2Br_2 as the internal standard (67% yield). **^{1}H NMR (400 MHz, $CDCl_3$):** δ 7.84 (dd, J = 6.1, 3.3 Hz, 4H), 7.47 (dd, J = 6.2, 3.2 Hz, 4H) ppm. These data match with the previously reported in literature.[21]

Reduction of 28

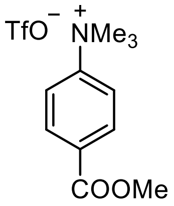

4-(methoxycarbonyl)-*N,N,N*-trimethylbenzenaminium trifluoromethanesulfonate **28** (34.3 mg, 0.1 mmol) was subjected to the reaction conditions described in general procedure F and irradiated for 24 hours. Yield of methyl benzoate was determined by ^{1}H NMR using CH_2Br_2 as the internal standard (45% yield). **^{1}H NMR (400 MHz, $CDCl_3$):** δ 3.90 (s, 3H), 7.42 (t, 2H, J=7.7 Hz), 7.54 (t, 1H, J=7.3 Hz), 8.04 (d, 2H, J=8.5 Hz) ppm. These data match with the previously reported in literature.[22]

Reduction of 29

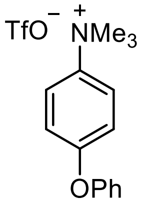

N,N,N-trimethyl-4-phenoxybenzenaminium trifluoromethanesulfonate **29** (37.7 mg, 0.1 mmol) was subjected to the reaction conditions described in general procedure F and irradiated for 24 hours. Yield of diphenylether was determined by ^{1}H NMR using CH_2Br_2 as the internal standard (39% yield). **^{1}H NMR (400 MHz, $CDCl_3$):** δ 7.58 – 7.43 (m, 4H), 7.34 – 7.15 (m, 6H) ppm. These data match with the previously reported in literature.[9]

Reduction of 30

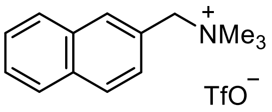

N,N,N-trimethyl-1-(naphthalen-2-yl)methanaminium trifluoromethanesulfonate **30** (34.9 mg) was subjected to the reaction conditions described in general procedure F and irradiated for 24 hours. Yield of 2-Methylnaphthalene was determined by was determined by ^{1}H NMR using CH_2Br_2 as the internal standard (67% yield). **^{1}H NMR (400 MHz, $CDCl_3$):** δ 7.76 (d, J = 7.8 Hz, 1H), 7.71 (dd, J = 7.8, 3.4 Hz, 2H), 7.57 (2, 1H), 7.39 (m, 2H), 7.27 (dd, J = 8.4, 1.6 Hz, 1H), 2.47 (s, 3H) ppm. These data match with the previously reported in literature.[23]

Reduction of 31

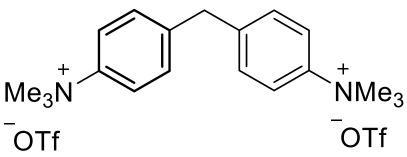

4,4'-methylenebis(*N,N,N*-trimethylbenzenaminium) trifluoromethanesulfonate **31** (28.4 mg) was subjected to the reaction conditions described in general procedure F and irradiated for 48 hours. Yield of diphenylmethane was determined by ^{1}H NMR using CH_2Br_2 as the internal standard (71% yield). After solvent evaporation, the product was purified through column chromatography (eluent: Hex). Product obtained: 11.1 mg (66% isolated yield). **^{1}H NMR (400 MHz, $CDCl_3$):** δ = 7.411 (t, J=8.0Hz , 4H); 7.331 (t, J = 3.2Hz, 4H); 7.311 (s, 2H), 4.111 (s, 2H) ppm. These data match with the previously reported in literature.[24]

Reduction of 32

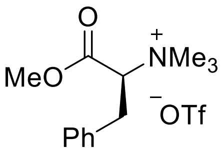

(S)-1-methoxy-N,N,N-trimethyl-1-oxo-3-phenylpropan-2-aminium trifluoromethanesulfonate **32** (22.2 mg) was subjected to the reaction conditions described in general procedure F and irradiated for 48 hours. Yield of methyl 3-phenylpropanoate was determined by ^{1}H NMR using CH_2Br_2 as the internal standard (70% yield). After solvent evaporation the product was purified through column chromatography (eluent: 7:3 Hex:EtOAc). Product obtained: 10.5 mg (64% isolated yield). **^{1}H NMR (400 MHz, $CDCl_3$):** δ 7.24–7.36 (m, 5H), 4.60 (s, 2H), 2.38 (s, 1H) ppm. These data match with the previously reported in literature.[25]

Reduction of 33

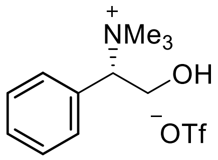

(S)-2-hydroxy-N,N,N-trimethyl-1-phenylethan-1-aminium trifluoromethanesulfonate **33** (18.0 mg) was subjected to the reaction conditions described in general procedure F and irradiated for 48 hours. Yield of 2-phenylethanol was determined by ^{1}H NMR using CH_2Br_2 as the internal standard (63% yield). After solvent evaporation, the product was purified through column chromatography (eluent: Hex). Product obtained: 7.3 mg (60% isolated yield).**^{1}H NMR (400 MHz, $CDCl_3$):** δ 7.24–7.36 (m, 5H), 4.60 (s, 2H), 2.38 (s, 1H) ppm. These data match with the previously reported in literature.[25]

Reduction of thermodynamically demanding substrates via ET

General procedure G

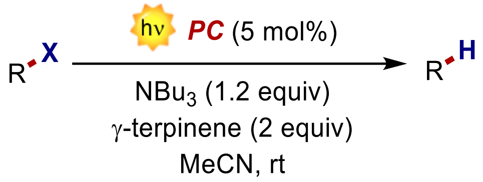

A 4 mL vial equipped with a septum was charged with a magnetic stirbar, the desired substrate **15**-**20** (0.1 mmol) and PC **5e** (5 mol%) and degassed with Ar. 1 mL of already degassed MeCN was added, followed by γ-terpinene (32 µL, 0.2 mmol, 2 equiv.) and NBu_3 (1.2 mmol, 1.2 equiv.). The reaction mixture was purged with Ar for 1 minute. The vial was irradiated with a 427 nm Kessil lamp set at 25% of its maximum output power for the indicated amount of time.

Reduction of 17

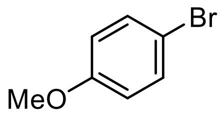

1-bromo-4-methoxy-benzene **17** (13 µL) was subjected to the reaction conditions described in general procedure G and irradiated for 48 hours. Yield of anisole was determined by ^{1}H NMR using CH_2Br_2 as the internal standard (<5% yield). **^{1}H NMR (400 MHz, $CDCl_3$):** δ 7.31-7.27 (m, 2H), 6.96-6.89 (m, 3H), 3.80 (s, 3H) ppm. These data match with the previously reported in literature.[18]

Reduction of 18

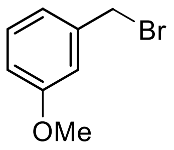

1-(bromomethyl)-3-methoxybenzene **18** (14 µL) was subjected to the reaction conditions described in general procedure G and irradiated for 24 hours. Yield of 1-methoxy-3-methyl-benzene was determined by ^{1}H NMR using CH_2Br_2 as the internal standard (43% yield). **^{1}H NMR (400 MHz, $CDCl_3$):** : δ 7.22-7.15 (m, 2H), 6.91-6.84 (m, 2H), 3.83 (s, 3H), 2.39 (s, 3H) ppm. These data match with the previously reported in literature.[19]

Reduction of 19

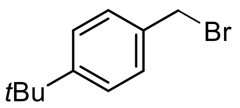

1-(bromomethyl)-4-(1,1-dimethylethyl)benzene **19** (18.4 µL) was subjected to the reaction conditions described in general procedure G and irradiated for 48 hours. Yield of 4-tert-butyltoluene was determined by ^{1}H NMR using CH_2Br_2 as the internal standard (55% yield). **^{1}H NMR (400 MHz, $CDCl_3$):** δ 7.32 (d, J = 8.2 Hz, 2H), 7.20 – 7.11 (m, 2H), 2.35 (s, 3H), 1.34 (s, 9H) ppm. These data match with the previously reported in literature.[20]

Reduction of 20

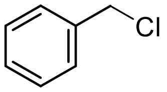

Benzyl chloride **20** (12 µL) was subjected to the reaction conditions described in general procedure G and irradiated for 48 hours. Conversion of **20** was determined by ^{1}H NMR using CH_2Br_2 as the internal standard (28% conversion).

Reduction of 21

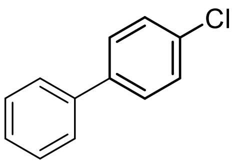

4-chloro-1,1'-biphenyl **21** (18.8 mg) was subjected to the reaction conditions described in general procedure G and irradiated for 60 hours. Yield of biphenyl was determined by GC-FID using 1,3,5-trimethoxybenzene as the internal standard (29% yield).

GC-FID yield: First, a calibration curve of the instrument was obtained for both 4-chloro-1,1'-biphenyl **21** and commercially available 1,1'-biphenyl with 1,3,5-trimethoxybenzene as the internal standard using the GC-FID method described below. Before irradiation, a sample of 100 µL was taken from the reaction mixture and diluted with 800 µL of MeCN. 100 µL of a 0.05 M solution of 1,3,5-trimethoxybenzene in MeCN were then added as the internal standard. The resulting solution was analysed by GC-FID and the initial concentration of 4-chloro-1,1'-biphenyl **21** was calculated using the calibration curve. To calculate the GC-FID yield at the end of the reaction, a sample of 100 µL was taken from the reaction crude and diluted with 800 µL of MeCN. 100 µL of a 0.05 M solution of 1,3,5-trimethoxybenzene in MeCN were then added as the internal standard. The concentration of 1,1'-biphenyl was obtained by GC-FID analysis using the calibration curve. Yield was calculated from the initial concentration of 4-chloro-1,1'-biphenyl **21**.

GC-FID method: Injector: 270°C, 1:100 split ratio, pressure 331.9 kPa, total flow 43.3 mL/min, column flow 0.40 mL/min, linear velocity 30.0 cm/s, purge flow 3.0 mL/min, He as carrier gas. Temperature program: 3 min at 40°C, 15°C/min until 280°C, 9 min at 280°C. Column: Equity 5, length 15.0 m, ID 0.10 mm, film thickness 0.10 µm. FID detector: 330 °C, H_2 flow 40.0 mL/min, Ar flow 400.0 mL/min, makeup flow 30.0 mL/min.

Reduction of 22

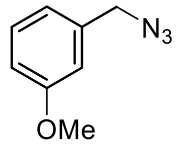

1-(azidomethyl)-3-methoxybenzene **22** (16.3 mg) was subjected to the reaction conditions described in general procedure G and irradiated for 24 hours. Yield of 1-methoxy-3-methyl-benzene was determined by ^{1}H NMR using CH_2Br_2 as the internal standard (42% yield). **^{1}H NMR (400 MHz, $CDCl_3$):** : δ 7.22-7.15 (m, 2H), 6.91-6.84 (m, 2H), 3.83 (s, 3H), 2.39 (s, 3H) ppm. These data match with the previously reported in literature.[19]

Reduction of 23

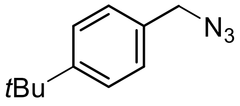

1-(azidomethyl)-4-(tert-butyl)benzene **23** (18.9 mg) was subjected to the reaction conditions described in general procedure G and irradiated for 24 hours. Yield of 4-tert-butyltoluene was determined by ^{1}H NMR using CH_2Br_2 as

the internal standard (29% yield). **^{1}H NMR (400 MHz, $CDCl_3$):** δ 7.32 (d, J = 8.2 Hz, 2H), 7.20 – 7.11 (m, 2H), 2.35 (s, 3H), 1.34 (s, 9H) ppm. These data match with the previously reported in literature.[20]

Reduction of 24

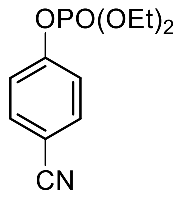

4-cyanophenyl diethyl phosphate **24** (25.5 mg) was subjected to the reaction conditions described in general procedure G and irradiated for 24 hours. Yield of benzonitrile was determined by GC-FID using 1,3,5-trimethoxybenzene as the internal standard (<5% yield).

GC-FID yield: First, a calibration curve of the instrument was obtained for commercially available benzonitrile **8** with 1,3,5-trimethoxybenzene as the internal standard using the GC-FID method described below. To calculate the GC-FID yield at the end of the reaction, a sample of 100 µL was taken from the reaction crude and diluted with 800 µL of MeCN. 100 µL of a 0.05 M solution of 1,3,5-trimethoxybenzene in MeCN were then added as the internal standard. The concentration of benzonitrile **8** was obtained by GC-FID analysis using the calibration curve.

GC-FID method: Injector: 270°C, 1:100 split ratio, pressure 331.9 kPa, total flow 43.3 mL/min, column flow 0.40 mL/min, linear velocity 30.0 cm/s, purge flow 3.0 mL/min, He as carrier gas. Temperature program: 3 min at 40°C, 3°C/min until 100°C, 40°C/min until 280°C, 9 min at 280°C. Column: Equity 5, length 15.0 m, ID 0.10 mm, film thickness 0.10 µm. FID detector: 330 °C, H_2 flow 40.0 mL/min, Ar flow 400.0 mL/min, makeup flow 30.0 mL/min.

Reduction of 25

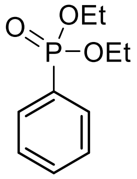

Diethyl phenylphosphonate **25** (21 µL) was subjected to the reaction conditions described in general procedure G and irradiated for 24 hours. The conversion was determined by ^{1}H NMR using CH_2Br_2 as the internal standard (<5% conversion).

Reduction of 26

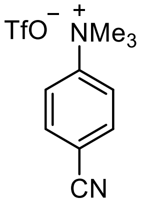

Cyano-*N,N,N*-trimethylbenzenaminium trifluoromethanesulfonate **26** (31.0 mg, 0.1 mmol) was subjected to the reaction conditions described in general procedure G and irradiated for 24 hours. Yield of benzonitrile was determined by GC-FID using 1,3,5-trimethoxybenzene as the internal standard (35% yield).

GC-FID yield: First, a calibration curve of the instrument was obtained for commercially available benzonitrile **8** with 1,3,5-trimethoxybenzene as the internal standard using the GC-FID method described below. To calculate the GC-FID yield at the end of the reaction, a sample of 100 µL was taken from the reaction crude and diluted with 800 µL of MeCN. 100 µL of a 0.05 M solution of 1,3,5-trimethoxybenzene in MeCN were then added as the internal standard. The concentration of benzonitrile **8** was obtained by GC-FID analysis using the calibration curve.

GC-FID method: Injector: 270°C, 1:100 split ratio, pressure 331.9 kPa, total flow 43.3 mL/min, column flow 0.40 mL/min, linear velocity 30.0 cm/s, purge flow 3.0 mL/min, He as carrier gas. Temperature program: 3 min at 40°C, 3°C/min until 100°C, 40°C/min until 280°C, 9 min at 280°C. Column: Equity 5, length 15.0 m, ID 0.10 mm, film thickness 0.10 µm. FID detector: 330 °C, H_2 flow 40.0 mL/min, Ar flow 400.0 mL/min, makeup flow 30.0 mL/min.

Reduction of 27

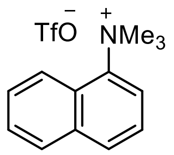

N,N,N-trimethylnaphthalen-1-aminium trifluoromethanesulfonate **27** (33,5 mg, 0.1 mmol) was subjected to the reaction conditions described in general procedure G and irradiated for 24 hours. Yield of naphthalene **27** was determined by ^{1}H NMR using CH_2Br_2 as the internal standard (39% yield). **^{1}H NMR (400 MHz, $CDCl_3$):** δ 7.84 (dd, J = 6.1, 3.3 Hz, 4H), 7.47 (dd, J = 6.2, 3.2 Hz, 4H) ppm. These data match with the previously reported in literature.[21]

Reduction of 28

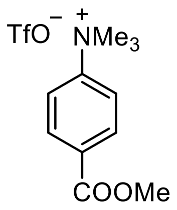

4-(Methoxycarbonyl)-*N,N,N*-trimethylbenzenaminium trifluoromethanesulfonate **28** (34.3 mg, 0.1 mmol) was subjected to the reaction conditions described in general procedure G and irradiated for 24 hours. Yield of methyl benzoate was determined by ^{1}H NMR using CH_2Br_2 as the internal standard (<5% yield). **^{1}H NMR (400 MHz, $CDCl_3$):** δ 3.90 (s, 3H), 7.42 (t, 2H, J=7.7 Hz), 7.54 (t, 1H, J=7.3 Hz), 8.04 (d, 2H, J=8.5 Hz) ppm. These data match with the previously reported in literature.[22]

Reduction of 29

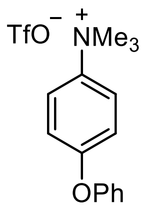

N,N,N-trimethyl-4-phenoxybenzenaminium trifluoromethanesulfonate **29** (37.7 mg, 0.1 mmol) was subjected to the reaction conditions described in general procedure G and irradiated for 24 hours. Yield of diphenylether was determined by ^{1}H NMR using CH_2Br_2 as the internal standard (<5% yield). **^{1}H NMR (400 MHz, $CDCl_3$):** δ 7.58 – 7.43 (m, 4H), 7.34 – 7.15 (m, 6H) ppm. These data match with the previously reported in literature.[9]

Reduction of 30

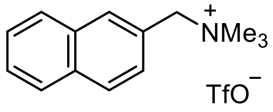

N,N,N-trimethyl-1-(naphthalen-2-yl)methanaminium trifluoromethanesulfonate **30** (34.9 mg) was subjected to the reaction conditions described in general procedure G and irradiated for 24 hours. Yield of 2-Methylnaphthalene was determined by was determined by ^{1}H NMR using CH_2Br_2 as the internal standard (67% yield). **^{1}H NMR (400 MHz, $CDCl_3$):** δ 7.76 (d, J = 7.8 Hz, 1H), 7.71 (dd, J = 7.8, 3.4 Hz, 2H), 7.57 (2, 1H), 7.39 (m, 2H), 7.27 (dd, J = 8.4, 1.6 Hz, 1H), 2.47 (s, 3H) ppm. These data match with the previously reported in literature.[23]

Reduction of 31

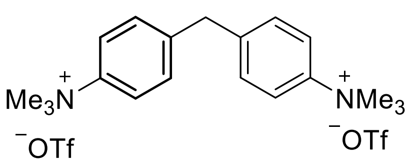

4,4'-methylenebis(N,N,N-trimethylbenzenaminium) trifluoromethanesulfonate **31** (28.4 mg) was subjected to the reaction conditions described in general procedure G and irradiated for 48 hours. Yield of diphenylmethane was determined by ^{1}H NMR using CH_2Br_2 as the internal standard (<5% yield). **^{1}H NMR (400 MHz, $CDCl_3$):** δ = 7.411 (t, J=8.0Hz, 4H); 7.331 (t, J = 3.2Hz, 4H); 7.311 (s, 2H), 4.111 (s, 2H) ppm. These data match with the previously reported in literature.[24]

Reduction of 32

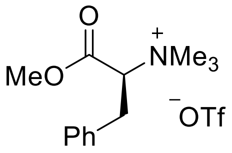

(S)-1-methoxy-N,N,N-trimethyl-1-oxo-3-phenylpropan-2-aminium trifluoromethanesulfonate **32** (22.2 mg) was subjected to the reaction conditions described in general procedure G and irradiated for 48 hours. Yield of methyl 3-phenylpropanoate was determined by ^{1}H NMR using CH_2Br_2 as the internal standard (<5%% yield). **^{1}H NMR (400 MHz, $CDCl_3$):** δ 7.24–7.36 (m, 5H), 4.60 (s, 2H), 2.38 (s, 1H) ppm. These data match with the previously reported in literature.[25]

Reduction of 33

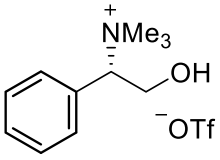

(S)-2-hydroxy-N,N,N-trimethyl-1-phenylethan-1-aminium trifluoromethanesulfonate **33** (18.0 mg) was subjected to the reaction conditions described in general procedure G and irradiated for 48 hours. Yield of 2-phenylethanol was determined by ^{1}H NMR using CH_2Br_2 as the internal standard (15% yield). **^{1}H NMR (400 MHz, $CDCl_3$):** δ 7.24–7.36 (m, 5H), 4.60 (s, 2H), 2.38 (s, 1H) ppm. These data match with the previously reported in literature.[25]

Polymerization of methyl methacrylate 35

General Polymerization Procedure in Batch

A 10 mL Schlenk tube was charged with a magnetic stirbar and the required amount of PC. Monomer **35**, MeCN and ethyl α-bromophenylacetate (BPA) **34** were then added sequentially via syringe. The Schlenk tube was then closed with a rubber septum and the reaction mixture degassed by Ar purging for 20 min. A 100 µL aliquot of the reaction mixture was then removed via syringe, injected into a vial containing 500 µL $CDCl_3$ with 250 ppm of butylated hydroxytoluene (BHT) and 25 µL of anisole as the internal standard and analyzed by ^{1}H NMR. The Schlenk tube was then irradiated by a Kessil LED PR160L light of the indicated wavelength set at its maximum output power. To analyze the progress of a polymerization at a given time point, a 100 µL aliquot of the reaction mixture was removed via syringe, injected into a vial containing 500 µL $CDCl_3$ with 250 ppm of butylated hydroxytoluene (BHT) and 25 µL of anisole as the internal standard and analyzed by ^{1}H NMR. Conversion of the monomer was calculated by comparison with the sample taken before irradiation.

Isolation of the polymer

At the end of the polymerization, the Schlenk tube was opened and the reaction mixture diluted with 1 mL of THF. The resulting solution was poured dropwise into 50 mL of MeOH under stirring, which caused the precipitation of the polymer. The mixture was stirred for 1 h and subsequently vacuum filtered. The obtained solid was dried under vacuum.

Analysis of Mn and Mw

A 5 mg/mL solution of the desired isolated polymer in DMF was prepared. Complete dissolution of the polymer was ensured by shaking the sample for 12 h. The solution was then filtered through a 0.45 µm PTFE filter and analyzed by GPC.

Polymerization kinetic

Kinetic studies were performed by following the General polymerization procedure with PC **5a** and irradiating with a Kessil LED PR160L 427 nm set at its maximum output power. The monomer

conversion over the reaction course were determined by ^{1}H NMR analysis, while Mn and Mw of the obtained polymer were determined by GPC analysis upon the standard isolation procedure.

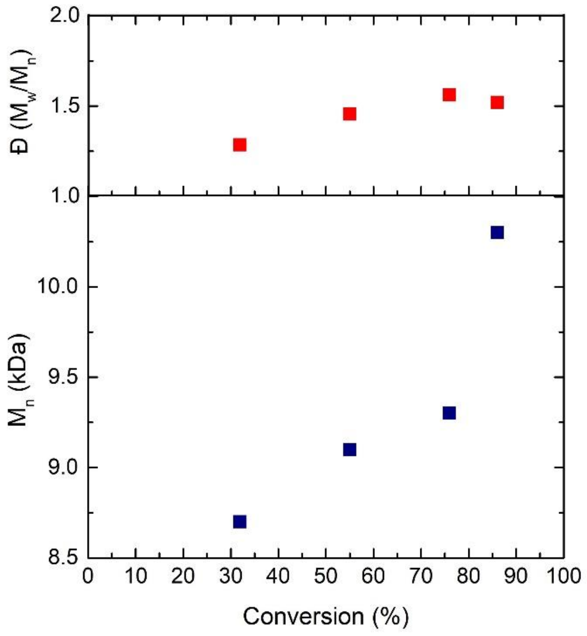

Figure S89: Plot of conversion of MMA as a function of M_n and Đ.

Polymerization of MMA in flow

A 10 mL flask was charged with **5a** (9.06 mg, 0.0282 mmol). MMA **35** (3 mL), DMAc (3 mL) and BPA **34** (49 µL, 0.282 mmol) were then added sequentially via syringe. The flask was then closed with a rubber septum and the reaction mixture degassed by Ar purging for 20 min. A 100 µL aliquot of the reaction mixture was then removed via syringe and analyzed by ^{1}H NMR. The solution was then pumped through the flow system depicted in Figure S80 at a linear velocity of 2.4 µL/min in order to have a residence time of 240 min of the reaction mixture in the photoreactor under the irradiation of a Kessil LED PR160L 427 nm light set at its maximum output power. The crude reaction mixture was collected and analyzed by ^{1}H NMR to determine the monomer conversion (86%, determined using the solvent peak as the internal standard). Conversion of the monomer was calculated by comparison with the sample taken before irradiation. The collected reaction mixture was poured dropwise into 300 mL of MeOH under stirring, which caused the precipitation of the polymer. The mixture was stirred for 1 h and subsequently vacuum filtered. The obtained solid was dried under vacuum. A 5 mg/mL solution of the isolated polymer in DMF was prepared. Complete dissolution of the polymer was ensured by shaking the sample for 12 h. The solution was then filtered through a 0.45 µm PTFE filter and analyzed by GPC.

On-Off experiments

On-off light experiments were carried out according to the General polymerization procedure with PCs **4c**, **5a** and **6c** irradiating with a Kessil LED PR160L 400 nm light set at its maximum output power. In all cases, no conversion of the monomer was detected when light irradiation was switched off (Figure S79).

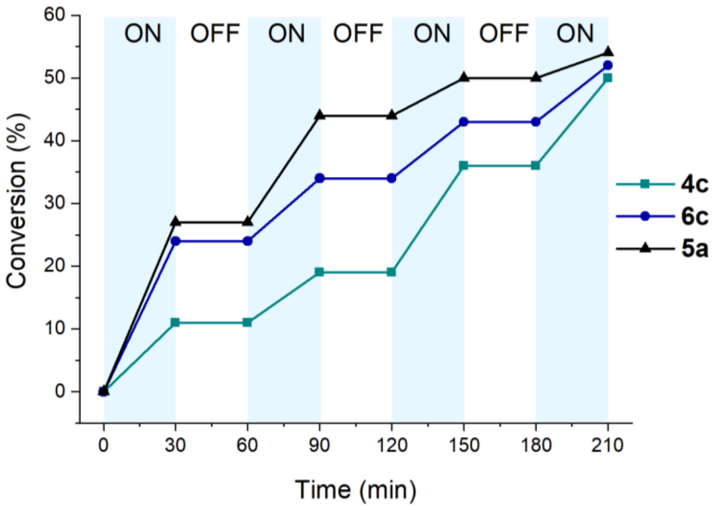

Figure S90: Plot of conversion of MMA as a function of time

Entry	PC	Ratio	Light source	Reaction time (h)	Conv (%)	M_w (kDa)	M_n (kDa)	PDI	I* (%)
**1**	**4a**	1000:10:1	390 nm	9	>98	19.8	12.6	1.59	78
**2**	**4b**	1000:10:1	390 nm	7	85	18.7	13.4	1.40	63
**3**	**4c**	1000:10:1	390 nm	2	95	13.1	8.4	1.56	113
**4**	**4c**	1000:10:1	390 nm	3	98	14.2	8.9	1.58	110
**5(a)**	**4c**	1000:10:1	400 nm	4	<10	-	-	-	-
**6**	**4c**	1000:10:1	400 nm	4	98	18.5	13.9	1.33	70
**7**	**4d**	1000:10:1	400 nm	4	76	19.8	13.1	1.51	58
**8**	**5a**	1000:10:0	427 nm	16	25	50.7	32.6	1.56	8
**9**	**5a**	1000:0:1	427 nm	16	5	-	-	-	-
**10**	**5a**	1000:10:1	-	16	6	-	-	-	-
**11**	**5a**	1000:10:1	427 nm	2	47	8.7	5.4	1.6	87
**12**	**5a**	1000:10:1	427 nm	6	86	10.8	6.9	1.52	124
**13**	**5b**	1000:10:1	427 nm	6	81	25.8	13.2	1.96	61
**14**	**5c**	1000:10:1	427 nm	6	>98	13.1	8.2	1.57	119
**15**	**5d**	1000:10:1	427 nm	6	>98	12.4	8.0	1.55	122
**16**	**5e**	1000:10:1	427 nm	6	>98	16.3	11.3	1.44	87
**17**	**6a**	1000:10:1	390 nm	2	84	12.1	8.6	1.41	98
**18**	**6a**	1000:10:1	400 nm	4	69	17.7	13.4	1.32	51
**19**	**6b**	1000:10:1	390 nm	2	76	13.8	9.6	1.43	79
**20**	**6c**	1000:10:1	390 nm	2	86	13.2	9.1	1.46	94
**21(b)**	**6c**	1000:10:1	390 nm	2	53	-	-	-	-
**22**	**6c**	1000:10:1	390 nm	3.5	95	15.0	10.0	1.49	95
**23**	**6c**	1000:10:1	390 nm	2	86	13.2	9.0	1.46	96
**24**	**6c**	1000:10:1	400 nm	4	81	17.4	12.6	1.38	64
**25**	**6d**	1000:10:1	400 nm	4	91	16.3	12.2	1.34	74
**26**	**6d**	1000:10:1	390 nm	2	84	15.0	11.0	1.36	76
**27**	**1, PTH**	1000:10:1	427 nm	6	5	24.9	15.2	1.64	3
**28(c)**	**5a**	1000:10:1	427 nm	4	86	19.1	15.8	1.21	54

Table S5: results of the light promoted polymerization of MMA in batch. Reaction were performed in MeCN with a ratio [MMA]:[EBP]:[PC] = [1000]:[10]:[1], [MMA]:[MeCN] = 1:1 (v/v) unless otherwise stated. (a) the reaction was performed without degassing. (b) The reaction was performed in the presence of MeTBD (32 µL, 0.2256 mmol). No polymer could be isolated during workup. (c) The reaction was performed in flow.

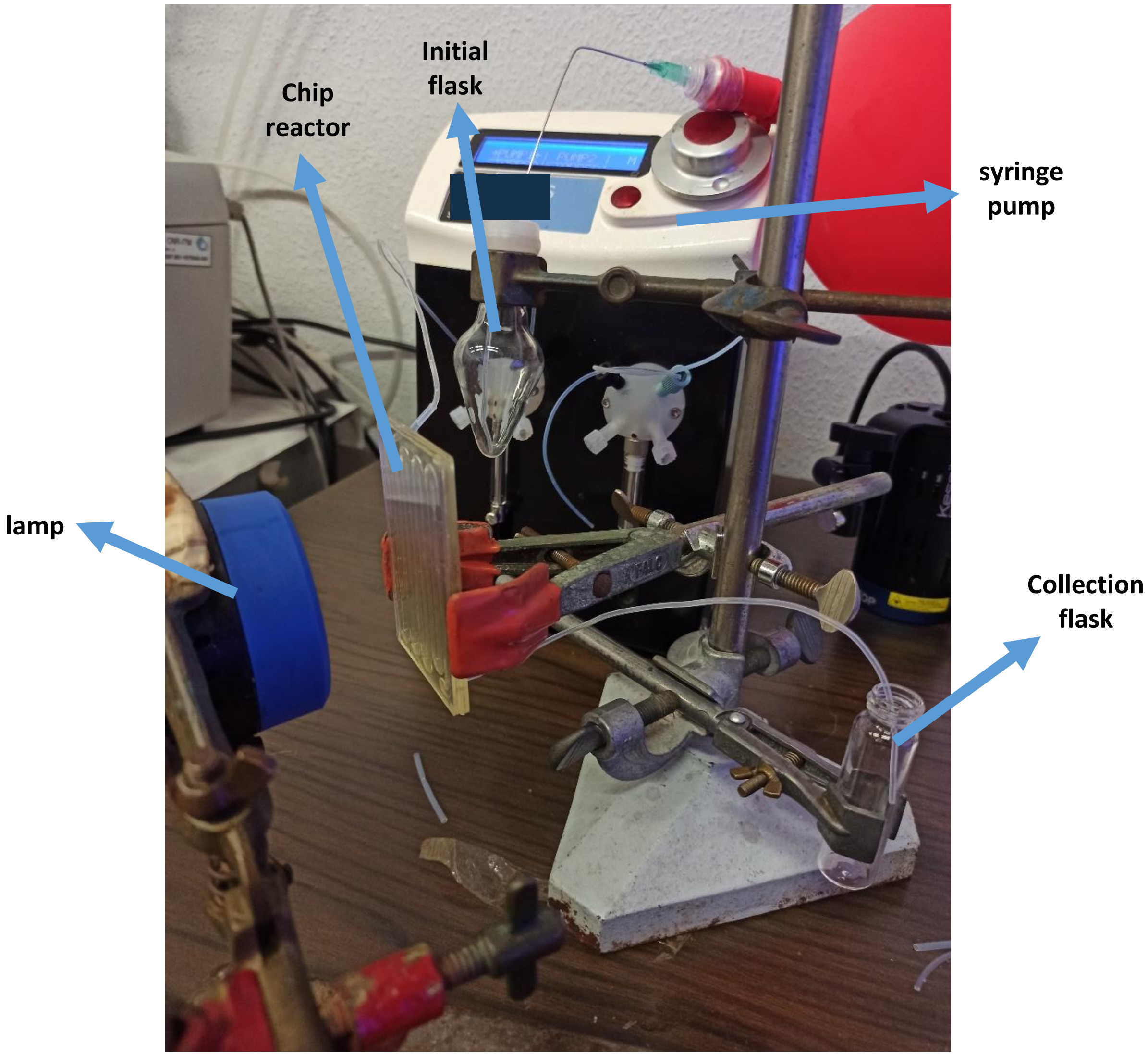

Figure S91: The flow system used for the polymerization of MMA. The lamp was placed at a fixed distance of 5 cm from the chip photoreactor. All the tubing, initial flask and collection flask were covered with aluminum foil to avoid undesired irradiation (not shown). Internal volume of the photoreactor: 570 µL.

H. Mechanistic studies

DFT calculations

All the calculations were performed with the GAUSSIAN16 suite of programs (Revision C.01). Each geometry was optimized with default convergence thresholds (RMS values of $3x10^{-4}$ hartree/bohr and $1.2x10^{-3}$ bohr and maximum values of $4.5x10^{-4}$ hartree/bohr and $1.8x10^{-3}$ bohr on forces and displacements, respectively) at B3LYP/6-311++G** level of theory. Polarization effects were taken into account with the polarizable continuum model (PCM).

Optimized geometries and SCF energies

9-methyl-9-phenyl-9,10-dihydroacridine (4a)

Neutral singlet, total number of atoms: 38

Vacuum calculation

SCF energy: -827.340541 Hartree

C -1.816870 -0.324840 0.636763
C -2.505108 -0.150598 -0.561164
C -3.886463 0.058008 -0.593809
C -4.576819 0.099838 0.628981
C -3.888577 -0.069024 1.839059
C -2.517638 -0.285651 1.842112
C -4.668608 0.118171 -1.919067
C -5.999993 0.865585 -1.717916
C -6.611990 0.877474 -0.453449
N -5.956398 0.303628 0.635453
C -7.878232 1.455419 -0.282889
C -8.544325 2.017145 -1.363257
C -7.948059 2.017890 -2.624187
C -6.687987 1.447684 -2.785881
C -3.825549 0.823985 -2.997116
C -3.428997 2.153452 -2.787584
C -2.667624 2.836372 -3.728506
C -2.280127 2.203751 -4.911594
C -2.664742 0.886766 -5.134614
C -3.430814 0.204123 -4.184659
H -1.686130 2.734201 -5.647354
C -5.022068 -1.344677 -2.305428
H -6.383369 0.443346 1.538614
H -8.458097 2.457548 -3.473048
H -0.745936 -0.489277 0.628763
H -3.723112 2.656446 -1.873206
H -2.375137 3.863648 -3.540408
H -2.372040 0.381022 -6.048319
H -3.714519 -0.820434 -4.387324
H -8.332350 1.461683 0.703481
H -9.523423 2.459850 -1.218882

H -6.225849 1.451983 -3.766151
H -4.120085 -1.948689 -2.432166
H -5.617831 -1.796352 -1.509879
H -5.611202 -1.378912 -3.225329
H -1.997002 -0.415994 2.784139
H -4.438017 -0.026336 2.774730
H -1.957765 -0.178859 -1.496117

PCM calculation

SCF energy: -827.348703 Hartree

C -1.820242 -0.393317 0.623297
C -2.516959 -0.220846 -0.570841
C -3.890155 0.041778 -0.594311
C -4.566021 0.136821 0.634855
C -3.868657 -0.029076 1.842529
C -2.506269 -0.297042 1.835758
C -4.672090 0.108324 -1.919578
C -6.006800 0.850566 -1.720076
C -6.600875 0.914360 -0.447409
N -5.934817 0.389290 0.656437
C -7.867375 1.498885 -0.284246
C -8.550179 2.012422 -1.378784
C -7.973252 1.957847 -2.649273
C -6.713573 1.382747 -2.802872
C -3.827824 0.830760 -2.987959
C -3.439791 2.160763 -2.762307
C -2.678032 2.859445 -3.692680
C -2.281734 2.241283 -4.881701
C -2.658171 0.923575 -5.120350
C -3.424602 0.225552 -4.180725
H -1.687812 2.783081 -5.609201
C -5.015378 -1.351917 -2.323213
H -6.351324 0.557946 1.560715
H -8.497283 2.357357 -3.509588
H -0.756666 -0.600466 0.607375
H -3.739923 2.653689 -1.844034
H -2.392400 3.886344 -3.492182
H -2.358882 0.429212 -6.038091
H -3.700763 -0.798275 -4.396845
H -8.306165 1.546765 0.707145
H -9.527838 2.459798 -1.238505
H -6.267677 1.341667 -3.790116
H -4.110097 -1.949632 -2.455079
H -5.611963 -1.815877 -1.534905
H -5.598681 -1.380822 -3.246852
H -1.980668 -0.424088 2.775568
H -4.405707 0.056349 2.781659
H -1.982201 -0.295893 -1.510822

9-methyl-9-(4-(trifluoromethyl)phenyl)-9,10-dihydroacridine (4b)

Neutral singlet, total number of atoms: 41

Vacuum calculation

SCF energy: -1164.490915 Hartree

C -1.800509 0.010428 0.681174
C -2.493951 0.062902 -0.531150
C -3.896610 -0.005453 -0.493850
C -4.568192 -0.119313 0.731963
C -3.855282 -0.159920 1.921881
C -2.462039 -0.097555 1.901348
C -1.772593 0.313428 -1.868470
C -2.634928 -0.183094 -3.043795
C -4.032259 -0.242155 -2.911519
N -4.622508 0.047521 -1.682536
C -4.834257 -0.583583 -4.010078
C -4.257189 -0.861207 -5.241220
C -2.871010 -0.811145 -5.387646
C -2.079563 -0.476469 -4.292333
C -0.417692 -0.416468 -1.873009
C 0.804075 0.242942 -2.006220
C 2.011535 -0.460509 -2.005055
C 2.008184 -1.842746 -1.869499
C 0.792387 -2.522648 -1.734599
C -0.398139 -1.814323 -1.737147
C 3.288374 -2.628834 -1.863157
C -1.618927 1.850672 -2.027595
H -5.614309 -0.121724 -1.610319
H -1.896273 -0.132269 2.824623
H -2.410691 -1.029831 -6.343687
H -1.333865 -2.350561 -1.632135
H 0.781556 -3.601130 -1.628504
H 2.947504 0.072920 -2.109968
H 0.837415 1.318815 -2.113570
H -5.652379 -0.177883 0.741379
H -4.386579 -0.247271 2.862826
H -0.717698 0.058173 0.669852
H -1.140078 2.105126 -2.976583
H -2.606729 2.315018 -2.017636
H -1.040668 2.278600 -1.204725
H -4.888497 -1.123070 -6.082744
H -5.912111 -0.631112 -3.887797
H -1.002749 -0.439164 -4.410512
F 4.381611 -1.849463 -2.009641
F 3.451433 -3.322142 -0.707319
F 3.323467 -3.547981 -2.861584

PCM calculation

SCF energy: -1164.500331 Hartree

C -1.796372 0.081248 0.681104
C -2.493428 0.079906 -0.531169
C -3.892990 -0.046013 -0.490442
C -4.557582 -0.166420 0.740915

C -3.840897 -0.155565 1.929882
C -2.450062 -0.032680 1.905848
C -1.773383 0.327302 -1.869924
C -2.634742 -0.166438 -3.047304
C -4.028737 -0.282657 -2.907423
N -4.625396 -0.044331 -1.673359
C -4.824462 -0.631646 -4.010802
C -4.244249 -0.858605 -5.251462
C -2.860757 -0.748424 -5.406083
C -2.076525 -0.406969 -4.306684
C -0.423802 -0.415147 -1.872937
C 0.803576 0.234514 -2.005277
C 2.004623 -0.479613 -2.002557
C 1.987220 -1.862568 -1.866230
C 0.765541 -2.533875 -1.732051
C -0.418038 -1.813424 -1.736301
C 3.258099 -2.657760 -1.858325
C -1.604652 1.862509 -2.029717
H -5.612044 -0.245210 -1.598276
H -1.881734 -0.024536 2.828388
H -2.398397 -0.924904 -6.370044
H -1.357491 -2.343323 -1.631830
H 0.741145 -3.612076 -1.625240
H 2.943283 0.048899 -2.106910
H 0.848731 1.309607 -2.113124
H -5.637616 -0.270835 0.752731
H -4.367233 -0.248677 2.873192
H -0.716697 0.178640 0.666565
H -1.120637 2.112077 -2.977126
H -2.588225 2.336446 -2.020740
H -1.021052 2.285258 -1.208235
H -4.870930 -1.126616 -6.094655
H -5.897941 -0.724675 -3.882330
H -1.003029 -0.320274 -4.431113
F 4.363541 -1.894535 -2.003185
F 3.419975 -3.358068 -0.702007
F 3.292632 -3.583020 -2.856321

4-(9-methyl-9,10-dihydroacridin-9-yl)benzonitrile (4c)

Neutral singlet, total number of atoms: 39

Vacuum calculation

SCF energy: -919.607895 Hartree

C 7.134898 8.820163 7.690979
C 6.512672 9.006154 6.453956
C 5.111796 9.095703 6.429415
C 4.359618 9.003960 7.588856
C 4.999208 8.816964 8.825375
C 6.393510 8.725745 8.867268
C 7.289278 9.116017 5.129720

C 6.861427 7.979092 4.183099
C 6.828390 8.195360 2.795491
N 7.082801 9.467815 2.287783
C 6.977801 10.607093 3.083180
C 7.016763 10.486493 4.482200
C 6.548117 7.136006 1.920161
C 6.306597 5.862005 2.414112
C 6.332380 5.631816 3.789594
C 6.606120 6.688037 4.653945
C 6.841284 11.868083 2.484635
C 6.749477 13.010508 3.266831
C 6.783121 12.907061 4.657432
C 6.913694 11.652462 5.246134
C 8.824586 8.997008 5.330053
H 6.933966 9.595069 1.298294
H 6.708918 13.792753 5.276863
H 6.141937 4.641437 4.185236
H 4.606138 9.239944 5.481980
H 3.279543 9.075462 7.549327
H 6.894170 8.581542 9.816938
H 8.212088 8.745778 7.756146
H 6.806003 11.942034 1.401959
H 6.645102 13.979142 2.791374
H 6.939314 11.576905 6.327202
H 9.095119 8.029277 5.760133
H 9.321688 9.081731 4.362024
H 9.204595 9.796631 5.971036
H 6.092018 5.051873 1.726473
H 6.519820 7.322686 0.850932
H 6.625037 6.504461 5.722128
C 4.231693 8.720916 10.028884
N 3.611263 8.643353 11.001086

PCM calculation

SCF energy: -919.621196 Hartree

C 7.122837 8.820863 7.689794
C 6.509416 9.007041 6.447935
C 5.108335 9.098570 6.409058
C 4.344359 9.008554 7.560666
C 4.977405 8.821258 8.801531
C 6.371978 8.727865 8.859569
C 7.298538 9.115404 5.129753
C 6.873988 7.976659 4.183598
C 6.793671 8.197703 2.797626
N 7.003171 9.473209 2.283798
C 6.942976 10.608512 3.085228
C 7.029468 10.487121 4.483087
C 6.509827 7.133163 1.926603
C 6.311886 5.852256 2.423794
C 6.386500 5.617194 3.798310
C 6.663770 6.677813 4.657475

C 6.803419 11.873935 2.492165
C 6.755579 13.016936 3.278526
C 6.838266 12.912443 4.668619
C 6.971949 11.654380 5.251169
C 8.831482 8.994821 5.344679
H 6.830407 9.601590 1.297338
H 6.800425 13.798619 5.290980
H 6.232115 4.621214 4.196131
H 4.609368 9.242966 5.458127
H 3.265087 9.081613 7.509280
H 6.866461 8.583568 9.812131
H 8.198784 8.745075 7.765936
H 6.730547 11.948229 1.412060
H 6.648287 13.987210 2.806780
H 7.038392 11.578096 6.330652
H 9.097185 8.028273 5.779766
H 9.337468 9.078534 4.380858
H 9.206447 9.792687 5.990377
H 6.094213 5.040031 1.739398
H 6.444142 7.323508 0.860342
H 6.723338 6.490312 5.723688
C 4.200345 8.726804 9.997164
N 3.570117 8.650513 10.964243

12-methyl-12-phenyl-7,12-dihydrobenzo[a]acridine (5a)

Neutral singlet, total number of atoms: 44

Vacuum calculation

SCF energy: -981.009061 Hartree

C 7.266232 11.174078 11.115560
C 7.356196 10.172296 10.140699
C 8.628018 9.698437 9.804705
C 9.773552 10.213077 10.416030
C 9.667571 11.212220 11.378197
C 8.403960 11.691152 11.725596
C 6.055871 9.591637 9.536168
C 5.086736 10.722531 9.099808
C 3.749584 10.669465 9.475797
N 3.250101 9.643379 10.266347
C 4.062148 8.734637 10.927180
C 5.430710 8.693479 10.632358
C 5.494797 11.793713 8.223782
C 4.538478 12.779212 7.802518
C 3.195601 12.679662 8.247793
C 2.811885 11.653539 9.056172
C 6.817225 11.944992 7.716353
C 7.163242 12.977705 6.874339
C 6.214654 13.941533 6.477242
C 4.926024 13.834815 6.940372
C 3.505562 7.866050 11.878983

C 4.304244 6.944948 12.538938
C 5.670096 6.888684 12.259881
C 6.212987 7.760023 11.320752
C 6.308974 8.678015 8.303786
H 10.555943 11.614405 11.852025
H 10.748257 9.829525 10.134194
H 8.304970 12.470379 12.473459
H 6.290856 11.556570 11.394780
H 8.742968 8.927790 9.052411
H 7.277610 7.720153 11.123889
H 6.307437 6.177831 12.772184
H 3.864267 6.277818 13.271545
H 2.442252 7.922924 12.091915
H 6.787966 9.215152 7.483574
H 6.923218 7.814477 8.567606
H 5.350902 8.299253 7.942289
H 2.296160 9.727425 10.579258
H 2.476829 13.427663 7.930718
H 1.782137 11.569274 9.390136
H 7.585570 11.245495 8.001983
H 8.184629 13.049576 6.516503
H 6.500369 14.751875 5.816512
H 4.174375 14.561415 6.648450

PCM calculation

SCF energy: -981.019010 Hartree

C 7.242883 11.176036 11.132642
C 7.350571 10.179858 10.152269
C 8.629742 9.719057 9.824717
C 9.765560 10.240943 10.449582
C 9.642740 11.234427 11.417175
C 8.370971 11.700563 11.756862
C 6.059439 9.592005 9.532120
C 5.088942 10.722591 9.098469
C 3.758354 10.691516 9.504583
N 3.264310 9.690991 10.325487
C 4.070866 8.754297 10.948568
C 5.431562 8.683980 10.618753
C 5.493985 11.782834 8.208228
C 4.540823 12.775152 7.792561
C 3.202869 12.693639 8.260538
C 2.822653 11.681483 9.088639
C 6.812876 11.920957 7.684356
C 7.158460 12.946451 6.831473
C 6.211888 13.915459 6.438024
C 4.926575 13.821844 6.917357
C 3.516954 7.882172 11.901398
C 4.310153 6.930212 12.524794
C 5.667735 6.842681 12.207856
C 6.207564 7.716797 11.268004

C 6.330021 8.684145 8.298899
H 10.523437 11.641408 11.901111
H 10.745729 9.867261 10.174000
H 8.258242 12.473680 12.509183
H 6.262084 11.548203 11.407889
H 8.759831 8.953193 9.070331
H 7.264050 7.647898 11.036899
H 6.299326 6.105018 12.688697
H 3.871979 6.261908 13.257475
H 2.461834 7.962247 12.142353
H 6.815559 9.222732 7.484175
H 6.948734 7.824840 8.564601
H 5.377169 8.300838 7.927922
H 2.311244 9.778188 10.643474
H 2.486035 13.445015 7.947590
H 1.799604 11.612562 9.443755
H 7.578370 11.214816 7.962090
H 8.175804 13.007088 6.460165
H 6.496168 14.718731 5.768128
H 4.177464 14.552558 6.629596

12-(4-methoxyphenyl)- 12-methyl-7,12-dihydrobenzo[a]acridine (5b)

Neutral singlet, total number of atoms: 48

Vacuum calculation

SCF energy: -1095.565102 Hartree

C 6.181630 7.762967 11.328675
C 5.400252 8.684045 10.622779
C 4.026597 8.709610 10.894511
C 3.463968 7.838867 11.840767
C 4.262071 6.929455 12.517599
C 5.633048 6.888458 12.261654
C 6.033320 9.584809 9.533523
C 5.061133 10.708758 9.084561
C 3.718402 10.641739 9.438745
N 3.215622 9.607023 10.216351
C 2.778047 11.618746 9.008719
C 3.164165 12.651261 8.209667
C 4.512442 12.764086 7.784371
C 5.471694 11.786119 8.216888
C 4.902151 13.824813 6.929344
C 6.195604 13.943503 6.482957
C 7.147068 12.986684 6.890262
C 6.799269 11.949770 7.726356
C 7.325696 10.172440 10.144517
C 7.234592 11.171821 11.114745
C 8.361595 11.705288 11.737255
C 9.631045 11.230987 11.395413
C 9.744580 10.225788 10.432133
C 8.607402 9.708677 9.821710
C 6.303171 8.671348 8.304398

O 10.801924 11.680086 11.938028
H 10.731753 9.862501 10.171807
H 8.235883 12.483314 12.478441
H 6.259055 11.554496 11.393644
H 8.737555 8.936564 9.073286
H 7.249951 7.736030 11.150454
H 6.269956 6.186875 12.787201
H 3.817667 6.259936 13.245381
H 2.396678 7.884594 12.035959
H 6.786326 9.211321 7.488318
H 6.921430 7.812703 8.575186
H 5.351519 8.284989 7.933953
H 2.256473 9.682798 10.515322
H 2.443357 13.393759 7.884302
H 1.744162 11.523597 9.326726
H 7.569484 11.256484 8.021554
H 8.172160 13.067133 6.544971
H 6.482714 14.757325 5.827072
H 4.147920 14.545229 6.628744
C 10.743794 12.705679 12.919665
H 11.775969 12.904263 13.204142
H 10.296805 13.620996 12.516453
H 10.180631 12.383682 13.802525

PCM calculation

SCF energy: -1095.576936 Hartree

C 6.179048 7.714017 11.268267
C 5.403389 8.673496 10.607274
C 4.038856 8.734166 10.921842
C 3.480533 7.860856 11.871210
C 4.273565 6.916185 12.505751
C 5.635217 6.837875 12.203839
C 6.037589 9.583069 9.525741
C 5.065533 10.709345 9.083528
C 3.731099 10.671027 9.476465
N 3.232098 9.662807 10.285845
C 2.795192 11.659001 9.056088
C 3.178015 12.674791 8.233770
C 4.518990 12.761669 7.775783
C 5.472806 11.772565 8.197369
C 4.906267 13.810241 6.903464
C 6.193888 13.908497 6.431661
C 7.141198 12.942284 6.830209
C 6.794290 11.915709 7.681174
C 7.324361 10.175643 10.147491
C 7.221476 11.168299 11.125154
C 8.341120 11.708059 11.757405
C 9.616644 11.246757 11.416148
C 9.741625 10.248739 10.445582
C 8.611025 9.724961 9.826219
C 6.318750 8.674550 8.295333

O 10.780859 11.704095 11.967183
H 10.730723 9.892052 10.182008
H 8.205495 12.478209 12.504808
H 6.242436 11.541379 11.405720
H 8.753389 8.959228 9.073732
H 7.238804 7.653793 11.050022
H 6.266806 6.106047 12.693581
H 3.832345 6.246725 13.235574
H 2.422173 7.933778 12.100342
H 6.806514 9.214205 7.482513
H 6.940646 7.819084 8.566357
H 5.370373 8.285234 7.919083
H 2.279579 9.753248 10.604793
H 2.461034 13.424467 7.917051
H 1.769440 11.585234 9.402175
H 7.560380 11.212686 7.964468
H 8.160427 13.006252 6.464661
H 6.479346 14.713062 5.763810
H 4.156092 14.538282 6.611659
C 10.707063 12.725807 12.963929
H 11.735668 12.931730 13.253314
H 10.251772 13.636475 12.563837
H 10.143788 12.385736 13.837802

12-(4-(trifluoromethyl)phenyl)- 12-methyl-7,12-dihydrobenzo[a]acridine (5c)

Neutral singlet, total number of atoms: 47

Vacuum calculation

SCF energy: -1318.159614 Hartree

C 7.334772 11.318535 10.934000
C 7.374566 10.264256 10.054518
C 8.591357 9.606320 9.748235
C 9.820339 10.024071 10.362952
C 9.729601 11.123212 11.264191
C 8.533400 11.747290 11.538336
C 8.608046 8.534066 8.819930
C 9.777427 7.912044 8.505085
C 11.000882 8.317804 9.107045
C 11.051659 9.343977 10.043191
C 12.418346 9.787139 10.628364
C 13.541901 8.774595 10.293597
C 13.367676 7.774530 9.328521
N 12.141734 7.643526 8.695398
C 14.420830 6.908952 8.994368
C 15.655194 7.035298 9.612259
C 15.848367 8.024714 10.576756
C 14.796245 8.873327 10.905533
C 12.359926 9.865618 12.171543
C 12.892425 10.933905 12.898725
C 12.869882 10.942590 14.292741
C 12.307844 9.874368 14.985280

C 11.775178 8.795462 14.275583
C 11.805642 8.796968 12.888391
C 12.320573 9.849758 16.486855
C 12.816554 11.133749 9.961520
H 13.281348 11.784967 14.834809
H 11.331413 7.962680 14.807413
H 11.384225 7.956891 12.349060
H 13.322814 11.784536 12.385967
H 14.953822 9.628544 11.666251
H 16.806125 8.131434 11.071686
H 16.461733 6.361577 9.346348
H 14.258016 6.139499 8.245782
H 12.101389 11.929409 10.175621
H 13.811159 11.456852 10.276158
H 12.846938 10.993790 8.879378
H 12.013406 6.849109 8.089217
H 7.677698 8.219704 8.359358
H 9.793715 7.097558 7.787383
H 10.613270 11.484322 11.764331
H 8.517002 12.579424 12.233688
H 6.398836 11.815754 11.160576
H 6.466935 9.915682 9.572209
F 11.223997 9.244047 16.999445
F 13.393578 9.171668 16.975280
F 12.376676 11.090303 17.023769

PCM calculation

SCF energy: -1318.170756 Hartree

C 7.339858 11.347720 10.908396
C 7.375732 10.278097 10.045104
C 8.590780 9.610527 9.749101
C 9.822895 10.035091 10.354934
C 9.735993 11.149122 11.240225
C 8.541206 11.781935 11.506119
C 8.602005 8.518530 8.841772
C 9.768076 7.882215 8.541883
C 10.995082 8.294499 9.136028
C 11.051393 9.345390 10.046256
C 12.417414 9.795077 10.627605
C 13.545520 8.789277 10.287171
C 13.361655 7.760813 9.352467
N 12.127290 7.597872 8.748312
C 14.419069 6.896767 9.020405
C 15.664680 7.054764 9.609595
C 15.867703 8.074672 10.542099
C 14.811880 8.921232 10.868757
C 12.359438 9.862713 12.172119
C 12.885792 10.929056 12.906218
C 12.863645 10.927356 14.300763
C 12.307005 9.850116 14.984369
C 11.780044 8.770870 14.267802

C 11.811133 8.783748 12.880805
C 12.309873 9.811268 16.482605
C 12.811613 11.146930 9.968960
H 13.273588 11.768583 14.845099
H 11.343474 7.928050 14.790115
H 11.396690 7.942757 12.337460
H 13.312595 11.786350 12.402442
H 14.978907 9.703806 11.599499
H 16.835236 8.207961 11.011302
H 16.473120 6.382594 9.345096
H 14.248648 6.105560 8.297611
H 12.097405 11.941510 10.187757
H 13.803736 11.472489 10.287523
H 12.842875 11.012409 8.885974
H 12.001041 6.802869 8.140654
H 7.670432 8.199181 8.387343
H 9.780501 7.050663 7.845106
H 10.621312 11.518602 11.731689
H 8.528479 12.626095 12.186993
H 6.405812 11.852218 11.126845
H 6.466860 9.924400 9.569071
F 11.159161 9.301115 16.990388
F 13.312237 9.028845 16.977212
F 12.475582 11.032110 17.041595

4-(12-methyl-7,12-dihydrobenzo[a]acridin-12-yl)benzonitrile (5d)

Neutral singlet, total number of atoms: 45

Vacuum calculation

SCF energy: -1073.276684 Hartree

N 7.110111 11.172628 11.058626
C 7.222220 10.308156 10.299394
C 7.362090 9.238818 9.359814
C 8.127782 9.411222 8.201450
H 8.616523 10.360296 8.017277
C 8.257494 8.367411 7.290094
C 7.633236 7.133701 7.503521
C 6.874117 6.975540 8.671236
C 6.732816 8.005192 9.588177
H 6.137960 7.864611 10.482416
H 6.381767 6.028904 8.859990
H 8.848303 8.534072 6.398543
C 7.804139 5.929719 6.548906
C 8.791080 4.964903 7.252602
C 9.995321 5.439608 7.783847
C 10.934276 4.591133 8.361345
C 10.673723 3.221938 8.423901
C 9.486057 2.723663 7.911601
C 8.547930 3.587335 7.325041
H 10.203337 6.502499 7.752920
H 11.856220 4.994917 8.762038

H 11.391190 2.545700 8.874435
H 9.271380 1.660392 7.958780
C 8.447517 6.314221 5.187089
H 7.841615 7.029844 4.629331
H 9.450075 6.726173 5.320435
H 8.544615 5.413047 4.578933
N 7.369566 3.074203 6.806985
H 7.175935 2.098483 6.966537
C 6.312544 3.872022 6.393308
C 6.438754 5.250329 6.264935
C 5.299804 5.987720 5.775252
C 4.076878 5.297875 5.472995
C 4.010275 3.892064 5.649148
C 5.095749 3.199600 6.091513
C 5.298807 7.394223 5.551491
C 2.949819 6.009730 4.993231
H 3.084515 3.374636 5.421811
H 5.049269 2.122379 6.218399
C 4.189411 8.059056 5.079397
C 2.994283 7.368349 4.796270
H 6.182259 7.974355 5.762283
H 4.239141 9.131712 4.927344
H 2.126733 7.903315 4.428008
H 2.043172 5.452484 4.780363

PCM calculation

SCF energy: -1073.291660 Hartree

N 7.090768 11.127338 11.102038
C 7.207902 10.270998 10.332991
C 7.351554 9.212665 9.384062
C 8.114644 9.403472 8.225687
H 8.598616 10.355519 8.046311
C 8.247385 8.368629 7.305211
C 7.629683 7.129799 7.510999
C 6.873476 6.954822 8.679692
C 6.727901 7.974031 9.607323
H 6.137002 7.819298 10.501620
H 6.387870 6.003908 8.863896
H 8.835924 8.548117 6.415230
C 7.803657 5.934654 6.544867
C 8.796062 4.969173 7.240358
C 10.019405 5.442011 7.729705
C 10.961465 4.595597 8.307665
C 10.683655 3.230669 8.411557
C 9.477931 2.734117 7.939116
C 8.536052 3.596096 7.351853
H 10.243560 6.500081 7.661555
H 11.898876 4.996790 8.674111
H 11.402847 2.556037 8.862025
H 9.250097 1.676028 8.017289
C 8.444436 6.331895 5.185481

H 7.839989 7.052433 4.633524
H 9.444977 6.746918 5.320650
H 8.542321 5.435057 4.570784
N 7.342593 3.084216 6.874093
H 7.147784 2.108684 7.039941
C 6.300594 3.878778 6.427027
C 6.438268 5.253760 6.265255
C 5.305291 5.989544 5.760291
C 4.078142 5.302123 5.465718
C 4.001651 3.898678 5.666876
C 5.079821 3.207763 6.130210
C 5.312154 7.394139 5.517575
C 2.955819 6.013361 4.971759
H 3.074261 3.382452 5.443824
H 5.026096 2.134506 6.279937
C 4.206918 8.059282 5.033120
C 3.008161 7.370273 4.755447
H 6.198900 7.973043 5.718830
H 4.263641 9.129219 4.864812
H 2.144724 7.904433 4.376230
H 2.046935 5.457771 4.764466

2,7-dimethoxy-9-methyl-9-phenyl-9,10-dihydroacridine (6a)

Neutral singlet, total number of atoms: 46

Vacuum calculation

SCF energy: -1056.447313 Hartree

C -3.459338 0.239752 -4.105689
C -3.855941 0.855475 -2.916887
C -3.461136 2.184730 -2.702700
C -2.699620 2.871355 -3.640483
C -2.310214 2.242777 -4.825011
C -2.693046 0.926235 -5.052556
C -4.698871 0.145404 -1.842883
C -3.922558 0.079235 -0.514246
C -4.626981 0.077612 0.704068
N -6.018274 0.229809 0.698828
C -6.659208 0.855392 -0.376688
C -6.032994 0.886945 -1.636597
C -3.934274 -0.092941 1.903389
C -2.552160 -0.271200 1.920087
C -1.848849 -0.268165 0.715327
C -2.541640 -0.090284 -0.484563
C -7.919615 1.432343 -0.216050
C -8.579371 2.035551 -1.285235
C -7.962045 2.071490 -2.535722
C -6.697255 1.500160 -2.694553
C -5.050876 -1.317164 -2.233536
H -1.715994 2.776163 -5.558494
H -6.445741 0.368076 1.602130
O -8.507648 2.635811 -3.661158

O -0.491279 -0.432216 0.602016
H -3.757068 2.684098 -1.786915
H -2.408464 3.898370 -3.448899
H -2.398836 0.423582 -5.967470
H -3.741616 -0.784426 -4.311913
H -8.396007 1.413418 0.759486
H -9.555334 2.472684 -1.123320
H -6.240881 1.544118 -3.675583
H -4.147369 -1.918393 -2.361297
H -5.646870 -1.771829 -1.439946
H -5.638623 -1.347680 -3.154267
H -2.048854 -0.400211 2.868688
H -4.480139 -0.085281 2.841979
H -1.970291 -0.090328 -1.404448
C -9.795280 3.220777 -3.560748
H -10.032397 3.596021 -4.555336
H -10.550542 2.483657 -3.263723
H -9.804557 4.054952 -2.849401
C 0.261392 -0.628277 1.787475
H 1.297145 -0.740259 1.469810
H 0.182553 0.232509 2.461850
H -0.052911 -1.534203 2.318982

PCM calculation

SCF energy: -1056.459677 Hartree

C -3.453124 0.263745 -4.100339
C -3.854703 0.870813 -2.908176
C -3.464667 2.200610 -2.684272
C -2.702631 2.897115 -3.615834
C -2.307966 2.276982 -4.804436
C -2.686304 0.959650 -5.041334
C -4.698612 0.150356 -1.839842
C -3.922747 0.081397 -0.510895
C -4.618191 0.113828 0.712241
N -6.004345 0.295707 0.720040
C -6.649418 0.891240 -0.367979
C -6.034270 0.889544 -1.633819
C -3.918142 -0.053780 1.910439
C -2.540478 -0.263254 1.920029
C -1.845831 -0.296945 0.709302
C -2.544589 -0.122283 -0.487725
C -7.910342 1.474146 -0.212637
C -8.579752 2.048133 -1.291692
C -7.974204 2.048528 -2.549802
C -6.710888 1.472258 -2.703387
C -5.043918 -1.310540 -2.241921
H -1.713769 2.817170 -5.532916
H -6.423629 0.457022 1.624549
O -8.533005 2.583321 -3.685200
O -0.491392 -0.494380 0.591375
H -3.763642 2.694759 -1.766304

H -2.415438 3.923841 -3.416734
H -2.388341 0.463732 -5.958649
H -3.730720 -0.759886 -4.315230
H -8.377110 1.482418 0.766954
H -9.553715 2.490312 -1.132273
H -6.261609 1.483122 -3.689122
H -4.138590 -1.907916 -2.373557
H -5.640499 -1.773633 -1.453294
H -5.627452 -1.338104 -3.165249
H -2.033409 -0.387901 2.867069
H -4.456300 -0.018187 2.852066
H -1.984628 -0.153766 -1.414599
C -9.828876 3.170453 -3.584546
H -10.077757 3.518755 -4.585372
H -10.573968 2.437243 -3.259702
H -9.828589 4.020301 -2.894652
C 0.265569 -0.692929 1.783737
H 1.296242 -0.834336 1.463384
H 0.207195 0.179361 2.442446
H -0.071319 -1.582376 2.325653

2,7-dimethoxy-9-(4-trifluoromethyl)-9-methyl-9,10-dihydroacridine (6b)

Neutral singlet, total number of atoms: 49

Vacuum calculation

SCF energy: -1393.597945 Hartree

C -0.483229 -1.844913 -1.730032
C -0.501712 -0.447009 -1.865274
C 0.720114 0.211743 -1.998000
C 1.927118 -0.492496 -1.996991
C 1.922825 -1.874640 -1.862057
C 0.706638 -2.553935 -1.727628
C -1.855293 0.283608 -1.860695
C -2.581122 0.035690 -0.525128
C -3.987357 0.004206 -0.491366
N -4.711977 0.106929 -1.683471
C -4.122602 -0.231294 -2.906166
C -2.721686 -0.209056 -3.034863
C -4.645832 -0.111755 0.733866
C -3.937232 -0.189135 1.930910
C -2.542477 -0.159578 1.902914
C -1.883441 -0.049969 0.676259
C -4.911271 -0.573991 -4.005615
C -4.338634 -0.888140 -5.236229
C -2.949742 -0.868780 -5.368906
C -2.160289 -0.532047 -4.266909
C -1.701836 1.821394 -2.019244
C 3.202313 -2.661605 -1.856281
F 3.238454 -3.578355 -2.857028
F 4.296544 -1.882638 -1.999554
F 3.364109 -3.358226 -0.702170

H -5.702703 -0.068658 -1.610862
O -1.735879 -0.228080 3.009042
O -2.269755 -1.157796 -6.523420
H -1.419507 -2.380232 -1.625403
H 0.695235 -3.632428 -1.621976
H 2.863364 0.040448 -2.101505
H 0.754140 1.287589 -2.104838
H -5.730847 -0.143875 0.756217
H -4.482042 -0.278706 2.860883
H -0.800674 -0.029324 0.685933
H -1.222579 2.074592 -2.968146
H -2.689639 2.285612 -2.009193
H -1.123323 2.247414 -1.195812
H -4.981971 -1.149311 -6.065445
H -5.991689 -0.598125 -3.901199
H -1.085523 -0.525326 -4.400082
C -2.350552 -0.332808 4.283343
H -1.536676 -0.368366 5.005992
H -2.984978 0.535374 4.496386
H -2.947791 -1.248073 4.367774
C -3.020461 -1.499123 -7.677790
H -2.291188 -1.681856 -8.465604
H -3.613493 -2.407030 -7.517937
H -3.683864 -0.681361 -7.982394

PCM calculation

SCF energy: -1393.611339 Hartree

C -0.495734 -1.856491 -1.728501
C -0.506499 -0.458056 -1.863985
C 0.718243 0.196152 -1.996188
C 1.921971 -0.513699 -1.994379
C 1.909554 -1.896662 -1.859115
C 0.690332 -2.572579 -1.725149
C -1.857458 0.279744 -1.860230
C -2.582543 0.032251 -0.524015
C -3.987398 -0.038067 -0.487875
N -4.719927 0.030634 -1.675570
C -4.122620 -0.273430 -2.901422
C -2.723232 -0.212609 -3.035165
C -4.641028 -0.158841 0.741997
C -3.928980 -0.202433 1.938617
C -2.534609 -0.132383 1.908158
C -1.880977 -0.017782 0.679033
C -4.906950 -0.621776 -4.005002
C -4.331224 -0.902706 -5.242107
C -2.942881 -0.843170 -5.380160
C -2.158562 -0.501024 -4.276125
C -1.694364 1.816303 -2.019193
C 3.183297 -2.687352 -1.852648
F 3.222082 -3.608981 -2.853835
F 4.286143 -1.919743 -1.993759

F 3.346389 -3.391066 -0.698644
H -5.706125 -0.172969 -1.600430
O -1.726329 -0.165871 3.017685
O -2.262014 -1.098496 -6.544940
H -1.433346 -2.389633 -1.624213
H 0.669962 -3.650937 -1.619157
H 2.858654 0.018311 -2.098574
H 0.759591 1.271444 -2.103211
H -5.724003 -0.222164 0.765747
H -4.470284 -0.297161 2.869988
H -0.798972 0.038358 0.682154
H -1.211096 2.066802 -2.966551
H -2.679325 2.287094 -2.010024
H -1.112151 2.239355 -1.197097
H -4.971195 -1.169189 -6.072085
H -5.985153 -0.676874 -3.896195
H -1.084209 -0.458243 -4.409661
C -2.345662 -0.265743 4.298862
H -1.533654 -0.267093 5.023735
H -3.002366 0.588640 4.490892
H -2.917462 -1.194299 4.393293
C -3.018218 -1.436640 -7.706311
H -2.291458 -1.586320 -8.502688
H -3.586848 -2.359800 -7.556225
H -3.701370 -0.628248 -7.985427

4-(2,7-dimethoxy-9-methyl-9,10-dihydroacridin-9-yl)benzonitrile (6c)

Neutral singlet, total number of atoms: 47

Vacuum calculation

SCF energy: -1148.715054 Hartree

C 6.850844 11.651163 5.156099
C 6.991265 10.497448 4.389920
C 6.996097 10.615830 2.988055
C 6.859713 11.875389 2.401865
C 6.726565 13.024006 3.178717
C 6.720571 12.912086 4.569668
C 7.259229 9.128309 5.042507
C 6.836342 7.993320 4.091099
C 6.847079 8.207188 2.700629
N 7.155225 9.474461 2.195669
C 6.567131 7.146331 1.837542
C 6.284105 5.872553 2.325327
C 6.271579 5.655156 3.703692
C 6.545626 6.718014 4.567422
C 6.479964 9.018791 6.364208
C 7.099617 8.832735 7.602151
C 6.355704 8.738559 8.776887
C 4.961675 8.830133 8.731988
C 4.324632 9.017241 7.494158
C 5.079198 9.108720 6.336505

C 4.191317 8.734374 9.933694
N 3.568141 8.657105 10.904187
C 8.794711 9.009142 5.245072
H 7.003915 9.601860 1.206504
O 6.596675 13.964032 5.439132
O 6.008139 4.451783 4.304028
H 4.576022 9.253050 5.387773
H 3.244675 9.088998 7.452513
H 6.854367 8.594270 9.727555
H 8.176593 8.758079 7.669577
H 6.855818 11.963101 1.319728
H 6.622577 13.982448 2.688579
H 6.843613 11.595872 6.237725
H 9.062764 8.041278 5.675947
H 9.293312 9.093680 4.277846
H 9.172140 9.809106 5.886843
H 6.071516 5.075670 1.625725
H 6.568298 7.315887 0.765171
H 6.529472 6.518663 5.631857
C 6.470900 15.270566 4.900050
H 6.392266 15.940424 5.754971
H 7.349194 15.546021 4.304900
H 5.569848 15.365949 4.283269
C 5.732304 3.333290 3.475565
H 5.560430 2.496354 4.150680
H 4.835383 3.495417 2.866749
H 6.579140 3.100362 2.819750

PCM calculation

SCF energy: -1148.732185 Hartree

C 6.883393 11.655099 5.160211
C 6.986103 10.498805 4.388913
C 6.950572 10.618158 2.987482
C 6.810188 11.882047 2.406350
C 6.713194 13.031182 3.187622
C 6.749127 12.918071 4.579112
C 7.252685 9.128917 5.040828
C 6.831065 7.992881 4.089879
C 6.801619 8.210615 2.700188
N 7.073562 9.480617 2.186436
C 6.517098 7.144882 1.841061
C 6.269824 5.865175 2.332500
C 6.299046 5.643705 3.711059
C 6.577387 6.709304 4.570027
C 6.463680 9.020526 6.358130
C 7.077378 8.834412 7.599569
C 6.326708 8.741207 8.769627
C 4.932277 8.834350 8.711818
C 4.298851 9.021590 7.471069
C 5.062520 9.111796 6.319411
C 4.155405 8.739692 9.907623

N 3.525498 8.663220 10.874876
C 8.786278 9.008459 5.255189
H 6.893838 9.609309 1.201171
O 6.662557 13.974842 5.451292
O 6.072022 4.430507 4.312364
H 4.563617 9.256132 5.368449
H 3.219560 9.094437 7.419963
H 6.821489 8.596946 9.722022
H 8.153318 8.758790 7.675578
H 6.774019 11.970196 1.325495
H 6.604791 13.990761 2.700952
H 6.912733 11.594431 6.241656
H 9.050857 8.041830 5.690376
H 9.292325 9.092071 4.291485
H 9.160064 9.806692 5.900841
H 6.052660 5.066919 1.636066
H 6.486164 7.317637 0.770302
H 6.598189 6.510738 5.635017
C 6.543116 15.288398 4.907641
H 6.499840 15.962031 5.761524
H 7.408925 15.542606 4.288097
H 5.627766 15.392927 4.316656
C 5.801703 3.305536 3.477726
H 5.664482 2.460905 4.150436
H 4.889106 3.454521 2.892041
H 6.639090 3.100344 2.803366

Additional data: HOMO and LUMO energies, ionization potentials and electron affinities, MOs primarily involved in the formation of the lowest excited states

In Table S6, HOMO and LUMO energies of the 10 photocatalysts are reported and compared to ionization potentials (IPs) and electron affinities (EAs) obtained with three well-known diagonal electron propagator methods. All the data reported in Table S6 were obtained without taking into account polarization effects (i.e., calculation of optimized geometries and properties were performed on the isolated molecule, without the employment of PCM). As expected, the presence of some functional groups can be easily related to the observed numerical values: for example, the presence of the nitrile group (which is an EWG) leads to the lowest values of the LUMO energies and to the lowest values of IPs.

Numerical values of IPs and EAs can be directly compared with transition frequencies measured with photoelectron and inverse photoelectron spectroscopies. As extensively discussed elsewhere,[26,27] a comparison of these values with oxidation and reduction potentials is not straightforward, mainly because of the relevance of polarization and solvation effects. In practice, the calculated difference between electron affinity and ionization potential in gas phase is expected to be an overestimation of the values observed in solution (usually measured through electrochemical techniques).

Polarization effects can be included in the picture (at least partially) with the employment of PCM, but in this case calculations with electron propagator methods cannot be performed. In Table S7, a comparison of HOMO and LUMO energies calculated for the isolated molecules with and without the employment of the PCM is proposed. When the PCM is employed, a decrease of the LUMO energy is observed in all the cases.

PC	B3LYP calc.		EPT results					
			IPs			EAs		
	E_{HOMO}	E_{LUMO}	OVGF	P3	P3+	OVGF	P3	P3+
**4a**	-5.40	-0.65	-6.89	-7.18	-7.06	0.64	0.61	0.61
**4b**	-5.59	-1.26	-7.08	-7.39	-7.26	0.53	0.50	0.49
**4c**	-5.66	-1.73	-7.13	-7.45	-7.32	0.49	0.45	0.44
**5a**	-5.24	-1.30	-6.62	-6.98	-6.83	0.58	0.55	0.54
**5b**	-5.20	-1.27	-6.58	-6.95	-6.80	0.59	0.55	0.54
**5c**	-5.44	-1.50	-6.82	-7.19	-7.04	0.48	0.44	0.43
**5d**	-5.51	-1.71	-6.87	-7.25	-7.10	0.43	0.39	0.38
**6a**	-4.91	-0.61	-6.37	-6.69	-6.56	0.63	0.58	0.58
**6b**	-5.08	-1.14	-6.54	-6.87	-6.74	0.53	0.48	0.47
**6c**	-5.15	-1.62	-6.59	-6.93	-6.80	0.49	0.43	0.43

*Table S6: All the calculations were performed with Gaussian 16 (revision C.01); basis set: 6-311++G**; the geometry of each molecule was optimized at B3LYP/6-311++G** level of theory, without the employment of the PCM; electron propagator theory (EPT) calculations were employed to estimate ionization potentials (IPs) and electron affinities (EAs); energies are given in eV.*

PC	Vacuum calc.		PCM calc.	
	E_{HOMO}	E_{LUMO}	E_{HOMO}	E_{LUMO}
**4a**	-5.40	-0.65	-5.47	-0.78
**4b**	-5.59	-1.26	-5.51	-1.28
**4c**	-5.66	-1.73	-5.52	-1.79
**5a**	-5.24	-1.30	-5.34	-1.45
**5b**	-5.20	-1.27	-5.32	-1.43
**5c**	-5.44	-1.50	-5.38	-1.51
**5d**	-5.51	-1.71	-5.40	-1.80
**6a**	-4.91	-0.61	-5.08	-0.80
**6b**	-5.08	-1.14	-5.11	-1.27
**6c**	-5.15	-1.62	-5.11	-1.79

Table S7A: HOMO and LUMO energies of the isolated molecules calculated with and without PCM; the corresponding optimized geometries were obtained with and without PCM, respectively; energies are given in eV.

PC	Transition	Coefficient
**4a**	Orbital 72 (HOMO) → Orbital 74 (LUMO+1)	0.69
**4b**	Orbital 88 (HOMO) → Orbital 89 (LUMO+1)	0.71
**4c**	Orbital 78 (HOMO) → Orbital 79 (LUMO+1)	0.71
**5a**	Orbital 85 (HOMO) → Orbital 86 (LUMO+1)	0.70
**5b**	Orbital 93 (HOMO) → Orbital 94 (LUMO+1)	0.70
**5c**	Orbital 101 (HOMO) → Orbital 102 (LUMO+1)	0.70
**5d**	Orbital 91 (HOMO) → Orbital 92LUMO+1)	0.71
**6a**	Orbital 88 (HOMO) → Orbital 89 (LUMO+1)	0.70
**6b**	Orbital 104 (HOMO) → Orbital 105 (LUMO+1)	0.71
**6c**	Orbital 94 (HOMO) → Orbital 95 (LUMO+1)	0.71

Table S7B: The most relevant transition involved in the formation of the lowest excited state is provided for each compound; the corresponding coefficients in the CI expansions are also reported; calculations were performed with Time-Dependent DFT, with the employment of the PCM.

Adduct between the electronic ground state of the neutral singlet 5a and TMG

Total number of atoms: 65

On the electronic ground state of the neutral singlet, two potential energy minima were found.

First optimized geometry

SCF energy: -1343.734127 Hartree

distance $N_{photocat—H}$ = 1.0296;

distance $N_{TMG—H}$ = 1.9472;

cartesian coordinates:

H 4.164504 -0.828541 3.487162
C 4.294617 -0.937477 2.415831
C 5.538563 -1.300749 1.895275
H 6.380186 -1.475134 2.556167
C 5.684046 -1.435761 0.517232
C 4.598903 -1.208058 -0.333485
C 3.348582 -0.839728 0.173853
C 3.216870 -0.713571 1.563871
H 2.257169 -0.432179 1.983400
H 4.747250 -1.312927 -1.401042
H 6.643083 -1.717182 0.095904
C 2.102153 -0.635200 -0.721720
C 2.444073 -0.564182 -2.237334
H 2.911923 -1.487511 -2.584642
H 3.103734 0.270434 -2.478002
H 1.517557 -0.433663 -2.800401
C 1.344843 0.661422 -0.331797
C 1.216571 -1.891068 -0.528338
C 1.766259 -3.170086 -0.672037
H 2.827916 -3.267712 -0.867723
C 0.995112 -4.325063 -0.566723
H 1.455499 -5.298913 -0.686112
C -0.372410 -4.211244 -0.303047
H -0.989828 -5.098376 -0.213317
C -0.944710 -2.957287 -0.149240
H -2.002906 -2.851912 0.062288
C -0.158817 -1.794097 -0.263473
C 1.996715 1.946966 -0.298918
C 3.384843 2.143406 -0.562366
C 3.969066 3.391106 -0.527739
C 3.210861 4.541130 -0.223966
C 1.867891 4.400108 0.038073
C 1.239060 3.130236 0.006400
C -0.151859 3.014379 0.269665
C -0.764431 1.799010 0.227983
H -1.825317 1.699842 0.427709
H -0.720881 3.908272 0.502902
H 1.259754 5.267664 0.274334
H 3.681291 5.517337 -0.198312
H 5.029468 3.485857 -0.736023
H 4.013814 1.297402 -0.787463
N -0.751595 -0.556964 -0.108829

H -1.755533 -0.517816 0.116318
C -4.764102 -0.046586 0.309295
N -5.814881 0.306416 1.151372
N -5.012445 0.119423 -1.042312
C -4.064396 -0.450704 -1.990892
C -7.179318 -0.137078 0.848835
C -5.686408 1.322608 -1.534131
C -0.025514 0.616973 -0.078314
H -4.951480 2.050419 -1.903270
H -6.359582 1.068618 -2.357479
H -6.261734 1.791383 -0.740534
H -4.557499 -0.539129 -2.961757
H -3.168945 0.172862 -2.112836
H -3.753303 -1.439001 -1.658238
H -7.418046 -1.055313 1.402086
H -7.898344 0.634506 1.136499
H -7.287579 -0.340670 -0.212925
C -5.544769 0.422990 2.578217
H -6.361392 0.979632 3.041759
H -5.475018 -0.553545 3.079945
H -4.618714 0.973832 2.745967
N -3.610088 -0.492018 0.709359
H -3.637729 -0.740906 1.694436

Second optimized geometry

SCF energy: -1343.715973 Hartree

distance $N_{photocat—H}$ = 1.6580;

distance $N_{TMG—H}$ = 1.0837;

cartesian coordinates:

C -0.171942 0.490835 0.495533
C 1.117848 0.646487 -0.055266
C 1.849144 -0.592389 -0.629690
C 1.088029 -1.891877 -0.274306
C -0.207180 -1.849434 0.299199
N -0.850242 -0.687689 0.639278
C -0.869462 1.655975 0.970775
C -0.335759 2.905047 0.898025
C 0.956465 3.106780 0.339087
C 1.688127 1.963551 -0.143231
C -0.867110 -3.083336 0.543732
C -0.282920 -4.301147 0.232804
C 0.996574 -4.338403 -0.331727
C 1.658042 -3.134033 -0.570722
C 1.506866 4.407663 0.256071
C 2.752800 4.630622 -0.289101
C 3.485423 3.525383 -0.769792
C 2.974901 2.246219 -0.698904
C 3.269623 -0.736252 -0.028321
C 4.400629 -1.035489 -0.795906

C 5.653343 -1.212842 -0.201549
C 5.801316 -1.095315 1.177903
C 4.680863 -0.799825 1.958078
C 3.436080 -0.626006 1.359889
C 1.859756 -0.490176 -2.182723
N -3.573222 -0.711732 0.941497
C -4.477450 -0.145497 0.146862
N -4.216919 0.004381 -1.171143
C -4.699906 1.155716 -1.937995
N -5.663780 0.284731 0.643892
C -5.826460 0.545326 2.074501
C -6.914015 0.165400 -0.114008
C -3.214061 -0.820519 -1.847405
H 4.778314 -0.704468 3.034400
H 6.772670 -1.230695 1.640410
H 2.575000 -0.396209 1.977812
H 4.321981 -1.124915 -1.872351
H 6.512383 -1.441268 -0.823497
H 2.292078 -1.382343 -2.641580
H 2.404931 0.383371 -2.543765
H 0.828209 -0.409994 -2.532283
H 2.654058 -3.166844 -0.999862
H 1.470754 -5.282266 -0.575976
H -0.818938 -5.223033 0.436193
H -1.852819 -3.054185 0.996035
H -1.850859 1.503989 1.406023
H -0.888442 3.763485 1.268113
H 0.917698 5.237697 0.635100
H 3.163407 5.632202 -0.348359
H 4.469017 3.679350 -1.201985
H 3.586149 1.439825 -1.071124
H -2.502875 -0.697715 0.772335
H -3.841711 1.658828 -2.391254
H -5.380878 0.844941 -2.734605
H -5.206526 1.861811 -1.285115
H -3.556887 -1.007393 -2.867082
H -2.241521 -0.322658 -1.882226
H -3.104052 -1.771647 -1.331390
H -7.616546 -0.445958 0.458947
H -7.365721 1.144936 -0.289023
H -6.734097 -0.324154 -1.067516
H -6.598615 1.305623 2.199339
H -6.135763 -0.352611 2.621653
H -4.898557 0.927445 2.497488
H -3.880096 -1.027769 1.849597

Adduct between the electronic ground state of the cationic doublet 5a$^{\cdot+}$ and TMG

On the electronic ground state of the cationic doublet (charge +1), two potential energy minima were found.

First optimized geometry

SCF energy: -1343.555397 Hartree

distance $N_{photocat-H}$ = 1.0805;

distance N_{TMG-H} = 1.6641;

cartesian coordinates:

H 4.220686 -0.853123 3.440173
C 4.312379 -0.952480 2.364457
C 5.535305 -1.310819 1.794989
H 6.399799 -1.490294 2.423738
C 5.633600 -1.435273 0.411906
C 4.520509 -1.201985 -0.399193
C 3.293220 -0.837357 0.160953
C 3.204269 -0.721399 1.554624
H 2.261777 -0.445157 2.015152
H 4.630942 -1.298533 -1.471819
H 6.576886 -1.712731 -0.044859
C 2.017261 -0.627147 -0.691108
C 2.297303 -0.531717 -2.226235
H 2.751360 -1.451906 -2.593835
H 2.950519 0.306339 -2.466662
H 1.354150 -0.391277 -2.757113
C 1.281588 0.663149 -0.286892
C 1.150761 -1.880842 -0.502062
C 1.678987 -3.156667 -0.698580
H 2.728429 -3.270449 -0.939120
C 0.886060 -4.295970 -0.581060
H 1.327657 -5.272344 -0.740281
C -0.472600 -4.186142 -0.254121
H -1.085151 -5.074226 -0.159422
C -1.025884 -2.938781 -0.048963
H -2.070142 -2.818905 0.211001
C -0.216262 -1.787040 -0.174449
C 1.917951 1.947741 -0.281675
C 3.299088 2.144781 -0.576270
C 3.869395 3.398505 -0.569471
C 3.099486 4.536037 -0.262914
C 1.759416 4.390769 0.033594
C 1.146719 3.121401 0.030510
C -0.242887 3.002705 0.334669
C -0.851033 1.791629 0.331378
H -1.902141 1.681195 0.564162
H -0.804590 3.899506 0.569181
H 1.154536 5.257760 0.274019
H 3.557519 5.517957 -0.257727
H 4.922960 3.506137 -0.798654
H 3.929824 1.299879 -0.797957
N -0.784720 -0.558622 0.041286
H -1.840270 -0.526877 0.270115
C -4.566282 -0.064404 0.296125

N -5.627808 0.297233 1.103227
N -4.749351 0.112095 -1.054784
C -3.832128 -0.524165 -1.991175
C -7.004547 -0.054861 0.742048
C -5.442331 1.285591 -1.589244
C -0.098306 0.615745 0.019980
H -4.722427 1.954131 -2.076088
H -6.190671 0.989320 -2.329878
H -5.933085 1.835409 -0.790669
H -4.341908 -0.641575 -2.950036
H -2.925169 0.070924 -2.157305
H -3.546820 -1.507681 -1.623412
H -7.351779 -0.892666 1.358792
H -7.673456 0.794448 0.905209
H -7.060297 -0.353420 -0.301170
C -5.409435 0.444816 2.537067
H -6.216993 1.052190 2.949546
H -5.407311 -0.518951 3.065203
H -4.466204 0.957304 2.726934
N -3.433437 -0.537289 0.750789
H -3.511033 -0.811235 1.725831

Second optimized geometry

SCF energy: -1343.567994 Hartree

distance $N_{photocat—H}$ = 1.9606;

distance $N_{TMG—H}$ = 1.0312;

cartesian coordinates:

C -0.088523 0.522810 0.276441
C 1.250888 0.649393 -0.164950
C 2.001437 -0.600311 -0.657108
C 1.204436 -1.883921 -0.385563
C -0.132182 -1.825949 0.076970
N -0.770857 -0.651950 0.361606
C -0.831148 1.683868 0.681300
C -0.278884 2.922061 0.645119
C 1.062842 3.101804 0.197082
C 1.836979 1.961103 -0.211246
C -0.858074 -3.029474 0.271648
C -0.279995 -4.256484 0.012525
C 1.041854 -4.311602 -0.448800
C 1.765451 -3.136315 -0.639491
C 1.625561 4.397119 0.152755
C 2.917599 4.599535 -0.281217
C 3.691385 3.493176 -0.683615
C 3.170091 2.218641 -0.647805
C 3.362431 -0.761576 0.063268
C 4.544236 -1.078012 -0.612948
C 5.739433 -1.269643 0.084794
C 5.771958 -1.151096 1.471564

C 4.596593 -0.840394 2.157918
C 3.407526 -0.650751 1.459766
C 2.127018 -0.491895 -2.209715
N -3.701619 -0.689027 0.926896
C -4.693162 -0.129323 0.223174
N -4.544603 0.061252 -1.102156
C -5.132081 1.211161 -1.798811
N -5.834078 0.234758 0.843105
C -5.872985 0.464917 2.289913
C -7.144905 0.131247 0.190647
C -3.580695 -0.720072 -1.881929
H 4.605830 -0.745656 3.238110
H 6.699626 -1.298200 2.012788
H 2.503387 -0.410386 2.008635
H 4.553425 -1.169237 -1.691780
H 6.643872 -1.510214 -0.462885
H 2.582064 -1.389799 -2.629206
H 2.714161 0.374708 -2.512849
H 1.129401 -0.393414 -2.641849
H 2.790150 -3.204354 -0.984552
H 1.507966 -5.268119 -0.654537
H -0.843401 -5.169083 0.167719
H -1.875422 -2.963371 0.637687
H -1.846265 1.535438 1.026701
H -0.847572 3.791607 0.955724
H 1.015122 5.236269 0.468661
H 3.337125 5.598349 -0.311343
H 4.710238 3.642809 -1.022219
H 3.806016 1.402100 -0.947992
H -2.711375 -0.653990 0.641245
H -4.334202 1.741940 -2.323636
H -5.879301 0.892072 -2.529178
H -5.588012 1.893854 -1.086646
H -3.987021 -0.857438 -2.884907
H -2.616756 -0.209481 -1.959268
H -3.437268 -1.698320 -1.428068
H -7.792309 -0.493305 0.810841
H -7.608844 1.113637 0.077947
H -7.047005 -0.338916 -0.784128
H -6.633334 1.219348 2.494620
H -6.133540 -0.446774 2.837842
H -4.913269 0.841344 2.639882
H -3.895883 -1.068746 1.840371

Adduct between the first excited electronic state of the neutral singlet 5a* and TMG

On the first excited electronic state of the neutral singlet, two potential energy minima were found.

First optimized geometry

TD-DFT energy: -1343.624773 Hartree

distance $N_{photocat—H}$ = 1.0527;

distance $N_{TMG—H}$ = 1.7924;

cartesian coordinates:

H 4.019749 -0.996473 3.528536
C 4.169150 -1.070739 2.456857
C 5.417087 -1.437429 1.947047
H 6.243206 -1.647103 2.617042
C 5.587663 -1.525618 0.568292
C 4.523310 -1.252726 -0.295371
C 3.270709 -0.882243 0.203446
C 3.112561 -0.796838 1.593526
H 2.151762 -0.503713 2.002851
H 4.691500 -1.322249 -1.362699
H 6.550954 -1.804714 0.155368
C 2.041394 -0.613942 -0.704878
C 2.403065 -0.492654 -2.222290
H 2.917514 -1.382002 -2.588732
H 3.028233 0.381693 -2.405745
H 1.481082 -0.380380 -2.795805
C 1.320536 0.674499 -0.293609
C 1.164171 -1.863914 -0.595893
C 1.675700 -3.135629 -0.855795
H 2.724847 -3.246309 -1.100383
C 0.875121 -4.273718 -0.795979
H 1.306465 -5.245749 -1.002636
C -0.487429 -4.163480 -0.462396
H -1.108540 -5.049898 -0.412779
C -1.029813 -2.926937 -0.196276
H -2.074471 -2.810731 0.064823
C -0.209993 -1.766676 -0.262186
C 1.990809 1.952569 -0.269130
C 3.373326 2.131316 -0.533622
C 3.983799 3.401347 -0.522058
C 3.227132 4.533475 -0.237811
C 1.862025 4.407818 0.042550
C 1.214505 3.142075 0.039935
C -0.167613 3.030345 0.333020
C -0.811483 1.794465 0.322915
H -1.865072 1.699752 0.551706
H -0.724342 3.930819 0.568985
H 1.269617 5.288197 0.269177
H 3.691370 5.514722 -0.228432
H 5.044261 3.486068 -0.732087
H 3.998605 1.276002 -0.731498
N -0.761361 -0.555617 0.002236
H -1.788594 -0.528461 0.230587
C -4.628783 -0.064938 0.308791
N -5.709195 0.229099 1.127564
N -4.791897 0.246735 -1.025458
C -3.828758 -0.270886 -1.988724

C -7.073618 -0.106231 0.708721
C -5.432847 1.497190 -1.436988
C -0.073067 0.646020 0.008462
H -4.675376 2.223008 -1.758837
H -6.114686 1.319546 -2.273376
H -5.991580 1.930122 -0.611763
H -4.290062 -0.261347 -2.978868
H -2.913677 0.334369 -2.029915
H -3.560127 -1.294325 -1.734321
H -7.402067 -1.036514 1.189806
H -7.765217 0.691388 0.992919
H -7.118638 -0.244683 -0.368020
C -5.516452 0.209013 2.571838
H -6.334722 0.760444 3.038583
H -5.516394 -0.809631 2.986048
H -4.579815 0.700200 2.836835
N -3.510028 -0.588894 0.726314
H -3.601662 -0.944501 1.673653

Second optimized geometry

TD-DFT energy: -1343.625448 Hartree

distance $N_{photocat—H}$ = 1.8550;

distance $N_{TMG—H}$ = 1.0435;

cartesian coordinates:

C -0.124147 0.524692 0.226665
C 1.228101 0.651266 -0.195774
C 1.992288 -0.589309 -0.674617
C 1.194564 -1.880983 -0.482691
C -0.156878 -1.833670 -0.031706
N -0.795084 -0.687952 0.283922
C -0.875920 1.651917 0.618529
C -0.299534 2.924307 0.584081
C 1.032436 3.113961 0.164519
C 1.835384 1.969070 -0.228701
C -0.880849 -3.060916 0.096714
C -0.296377 -4.270140 -0.208562
C 1.036213 -4.308576 -0.655493
C 1.756732 -3.121934 -0.782768
C 1.617384 4.420673 0.118631
C 2.936813 4.613780 -0.287003
C 3.720923 3.521443 -0.655382
C 3.172303 2.220698 -0.623140
C 3.308099 -0.781566 0.123683
C 4.534867 -1.081386 -0.477822
C 5.682903 -1.290056 0.291745
C 5.625914 -1.206008 1.680229
C 4.406026 -0.909153 2.293621
C 3.266171 -0.700578 1.522679
C 2.215779 -0.457376 -2.215647

N -3.623696 -0.757549 0.892761
C -4.617243 -0.138206 0.249839
N -4.479997 0.171701 -1.055245
C -5.068757 1.382709 -1.636742
N -5.758589 0.171284 0.903650
C -5.791242 0.273061 2.364283
C -7.071894 0.113952 0.251723
C -3.503908 -0.517945 -1.902838
H 4.343819 -0.838955 3.374347
H 6.516654 -1.365093 2.277603
H 2.327136 -0.462346 2.010648
H 4.615965 -1.145165 -1.555772
H 6.622211 -1.515581 -0.201825
H 2.748668 -1.317312 -2.624852
H 2.767353 0.450860 -2.461033
H 1.241474 -0.407380 -2.705941
H 2.786898 -3.172267 -1.115993
H 1.505975 -5.255206 -0.895663
H -0.861253 -5.189639 -0.102081
H -1.905166 -3.016613 0.447148
H -1.896212 1.509125 0.950044
H -0.879701 3.792363 0.882745
H 1.004890 5.267987 0.410691
H 3.352489 5.617152 -0.311104
H 4.751260 3.661282 -0.964054
H 3.818160 1.400084 -0.890063
H -2.621719 -0.701290 0.606752
H -4.270861 1.968720 -2.099473
H -5.808823 1.134448 -2.401221
H -5.534589 1.987802 -0.863501
H -3.904671 -0.560174 -2.916714
H -2.545392 0.008122 -1.919820
H -3.349363 -1.533695 -1.545127
H -7.710474 -0.572172 0.813947
H -7.546839 1.097644 0.232313
H -6.975251 -0.262044 -0.763344
H -6.552343 1.004447 2.638788
H -6.046806 -0.683465 2.832824
H -4.830745 0.620233 2.741354
H -3.818090 -1.206630 1.774432

Comment

A computational modelling of the adduct between the photocatalyst **5a** and the base TMG is proposed. More specifically, three different electronic states were modelled at DFT level. In the framework of the Born-Oppenheimer approximation, each electronic state is associated to a potential energy surface (PES): a stable molecular conformation corresponds to a minimum of the potential energy surface, and therefore the conformational landscape of a molecular species can be investigated through a search of the critical points of a certain PES. For each PES, the calculations performed on the adduct between **5a** and TMG lead to the identification of two minima associated with similar molecular structures: the most relevant difference lies in the position of a single proton, which can be closer to the nitrogen

atom of the photocatalyst **5a** (in this case, the photocatalyst is protonated and the minimum is labeled $E_{H\text{-photocat.}}$) or to the iminic nitrogen of the TMG (in this case, the photocatalyst is deprotonated and the minimum is labeled $E_{H\text{-TMG}}$). The relative stability of the two minima depends on the electronic state (i.e., on the specific PES where the two minima are located). On the electronic ground state of the neutral singlet $E_{H\text{-photocat.}} < E_{H\text{-TMG}}$, and therefore the protonated form of the photocatalyst is predicted to be the most stable one (with ΔE = 11.39 kcal/mol). On the other hand, $E_{H\text{-photocat.}} > E_{H\text{-TMG}}$ in the cases of the first excited state of the neutral singlet (ΔE = 0.42 kcal/mol) and of the ground state of the cationic doublet (ΔE = 7.90 kcal/mol), i.e., upon excitation or oxidation of the adduct the deprotonated form of the photocatalyst is predicted to be the most stable one. The situation is schematically depicted in Figure S92. These results support the hypothesis of a coupling between the oxidation and the deprotonation of the photocatalyst.

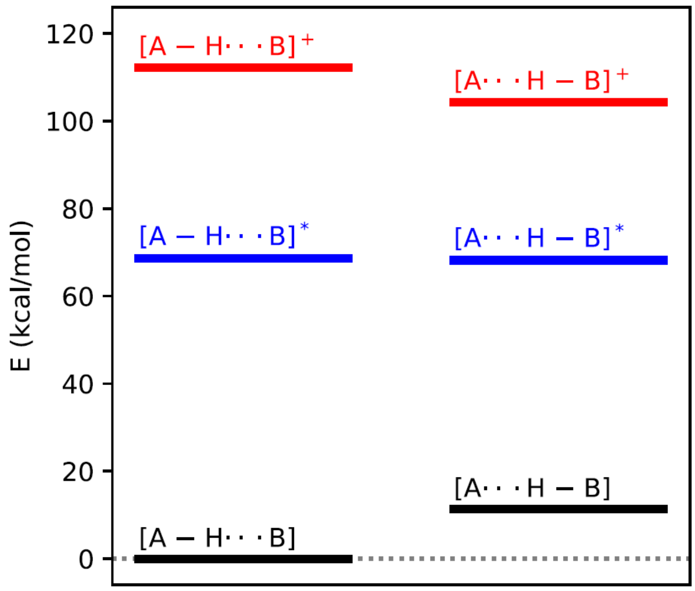

Figure S92: Energies of the six minima of the PESs discussed in the text. Energies are given in kcal/mol, relative to the smallest calculated value. Different colors are referred to different PESs: black for the ground state of the neutral singlet, blue for the first excited state of the neutral singlet and red for the ground state of the cationic doublet (i.e., the oxidized electronic state).

Adduct between the electronic ground state of the neutral singlet 5a and MeTBD

Total number of atoms: 70

On the electronic ground state of the neutral singlet, two potential energy minima were found.

First optimized geometry

SCF energy: -1459.29290999 Hartree

distance $N_{photocat}$—H = 1.0321;

distance N_{MeTBD}—H = 1.9242;

cartesian coordinates:

N -4.547302 0.593958 -1.212900
C -4.372605 0.304161 0.132055
N -5.514030 0.137822 0.889564

N -3.157267 0.241439 0.607600
C -3.375673 0.617392 -2.077664
N -0.372362 -0.202563 -0.253916
C 0.509607 0.853469 -0.139977
C 1.878537 0.719995 -0.369808
C 2.457209 -0.643904 -0.829449
C 1.399601 -1.771446 -0.734101
C 0.045575 -1.498180 -0.481581
C 2.702663 1.896144 -0.244488
C 2.110598 3.153907 0.122695
C 0.711085 3.219820 0.355341
C -0.064127 2.107514 0.228385
C -0.897098 -2.544359 -0.453630
C -0.502788 -3.854148 -0.682958
C 0.840618 -4.142583 -0.936882
C 1.767783 -3.103415 -0.955282
C 2.910167 4.317693 0.246544
C 4.266053 4.282909 0.017542
C 4.863389 3.057731 -0.345646
C 4.111148 1.909835 -0.470586
C 2.847209 -0.528463 -2.330702
C 3.637612 -1.077535 0.074167
C 4.835086 -1.593409 -0.432190
C 5.853079 -2.025235 0.422407
C 5.691939 -1.950705 1.803313
C 4.500027 -1.440849 2.322708
C 3.488915 -1.013567 1.466792
H -1.934228 -2.304437 -0.247845
H 0.267054 4.169477 0.635069
H -1.133076 2.146311 0.405352
H -1.240051 -4.649261 -0.659308
H 2.809478 -3.337417 -1.142565
H 1.963925 -0.233431 -2.901023
H 3.623706 0.217463 -2.504547
H 3.190222 -1.485348 -2.729010
H 4.995355 -1.656893 -1.501291
H 2.569134 -0.619884 1.885415
H 6.772459 -2.418165 0.001906
H 4.358543 -1.376091 3.396175
H -1.369888 -0.034249 -0.049611
H 1.162890 -5.161933 -1.114853
H 6.481722 -2.283752 2.467275
H 4.621244 0.998538 -0.737781
H 5.931799 3.013849 -0.528358
H 4.867890 5.179248 0.114107
H 2.423549 5.246444 0.527497
H -2.589272 1.223581 -1.633296
H -2.971388 -0.385402 -2.268531
H -3.664027 1.062047 -3.031161
C -3.011431 0.072565 2.049851
H -3.083404 1.045219 2.561340
H -2.004501 -0.306109 2.250302

C -5.438168 -0.318428 2.283614
H -5.688421 0.517350 2.949993
H -6.207967 -1.084309 2.427509
C -4.063044 -0.872813 2.621816
H -3.937290 -1.865717 2.177975
H -3.964642 -0.976965 3.705601
C -6.883567 0.209487 0.372889
H -7.493077 0.755794 1.100891
H -7.294880 -0.808028 0.311238
C -5.831103 0.328293 -1.858320
H -5.821295 0.821614 -2.831492
H -5.977772 -0.748359 -2.038652
C -6.950164 0.877876 -0.989171
H -7.924378 0.684726 -1.443977
H -6.831482 1.960986 -0.892875

Second optimized geometry

SCF energy: -1459.27954856 Hartree

distance $N_{photocat}$—H = 1.7841;

distance N_{MeTBD}—H = 1.0544;

cartesian coordinates:

N -4.250097 0.284539 -1.261012
C -4.265767 0.010040 0.069956
N -5.407681 0.161005 0.780165
N -3.147495 -0.405856 0.677343
C -3.150301 -0.158242 -2.123089
N -0.452056 -0.457826 -0.134715
C 0.334961 0.657460 -0.070379
C 1.723296 0.715890 -0.323211
C 2.458676 -0.561630 -0.798579
C 1.534236 -1.797576 -0.691880
C 0.152359 -1.657384 -0.406420
C 2.403979 1.977392 -0.216188
C 1.679996 3.166403 0.155057
C 0.283536 3.065056 0.404811
C -0.352728 1.867746 0.295673
C -0.644897 -2.833592 -0.394806
C -0.109080 -4.086207 -0.649526
C 1.256282 -4.219891 -0.923679
C 2.050713 -3.073457 -0.937843
C 2.339579 4.413461 0.262863
C 3.689573 4.539818 0.015532
C 4.417472 3.388677 -0.351464
C 3.800931 2.160052 -0.461959
C 2.814500 -0.399078 -2.305315
C 3.698223 -0.860992 0.081020
C 4.939177 -1.236783 -0.445318
C 6.014363 -1.554069 0.389678
C 5.870133 -1.503342 1.773615

C 4.636602 -1.132639 2.314291
C 3.569738 -0.819059 1.476968
H -1.702299 -2.730330 -0.175760
H -0.267008 3.958609 0.683897
H -1.417093 1.796325 0.489584
H -0.752072 -4.960800 -0.630520
H 3.110326 -3.180671 -1.145462
H 1.893417 -0.209043 -2.860653
H 3.495180 0.433502 -2.489958
H 3.260694 -1.308782 -2.713729
H 5.087736 -1.277452 -1.517325
H 2.618178 -0.531672 1.910611
H 6.965128 -1.839015 -0.048302
H 4.506909 -1.087487 3.390499
H -2.201287 -0.403746 0.212026
H 1.693029 -5.192411 -1.120845
H 6.703613 -1.747472 2.422842
H 4.411648 1.314220 -0.734046
H 5.481809 3.466175 -0.549463
H 4.183705 5.501015 0.100483
H 1.750724 5.280818 0.547040
H -2.185577 -0.091435 -1.623935
H -3.308603 -1.190087 -2.457488
H -3.127426 0.491407 -2.998197
C -5.497233 0.597188 -1.971536
H -5.224527 1.152544 -2.869511
H -5.991402 -0.329723 -2.291476
C -6.414872 1.419602 -1.086712
H -5.952021 2.386819 -0.872345
H -7.361967 1.605285 -1.596060
C -6.665112 0.657852 0.200293
H -7.132793 1.303639 0.947032
H -7.337724 -0.190340 0.028294
C -5.492439 -0.170431 2.214149
H -5.429475 0.755186 2.797523
H -6.482246 -0.599976 2.383671
C -3.072398 -0.629890 2.121149
H -2.268294 -1.346071 2.293782
H -2.803493 0.300149 2.636407
C -4.406042 -1.146969 2.631006
H -4.606942 -2.136865 2.211470
H -4.393277 -1.237772 3.718502

Adduct between the electronic ground state of the cationic doublet 5a$^{\cdot+}$ and MeTBD

On the electronic ground state of the cationic doublet (charge +1), two potential energy minima were found.

First optimized geometry

SCF energy: -1459.11467872 Hartree

distance $N_{photocat}$—H = 1.1035 Å;

distance N_{MeTBD}—H = 1.5918 Å;

cartesian coordinates:

N -4.286964 0.546776 -1.231228
C -4.176442 0.300787 0.117621
N -5.337604 0.123479 0.826351
N -2.979240 0.269536 0.667620
C -3.085915 0.685540 -2.042366
N -0.439040 -0.165878 -0.095157
C 0.434799 0.875546 -0.033677
C 1.810280 0.714505 -0.322682
C 2.334017 -0.651804 -0.799544
C 1.268573 -1.753188 -0.702944
C -0.072167 -1.453565 -0.387999
C 2.649479 1.873340 -0.228391
C 2.075889 3.137292 0.149195
C 0.680090 3.231421 0.431078
C -0.118580 2.139820 0.345350
C -1.061628 -2.462953 -0.352651
C -0.716060 -3.769446 -0.631567
C 0.613984 -4.082223 -0.943839
C 1.585212 -3.084150 -0.974304
C 2.888672 4.285620 0.239121
C 4.240229 4.224862 -0.032606
C 4.818842 2.995900 -0.401182
C 4.050413 1.856561 -0.493767
C 2.662558 -0.511133 -2.321602
C 3.537295 -1.121250 0.054944
C 4.698169 -1.657478 -0.508882
C 5.737351 -2.119665 0.302195
C 5.630082 -2.056002 1.688751
C 4.472367 -1.527125 2.261636
C 3.437941 -1.067799 1.451672
H -2.078239 -2.189455 -0.100185
H 0.268624 4.192757 0.716091
H -1.178290 2.192148 0.559418
H -1.468654 -4.547852 -0.605125
H 2.607780 -3.354523 -1.205374
H 1.768159 -0.184064 -2.854615
H 3.450534 0.219746 -2.499693
H 2.966853 -1.469658 -2.742003
H 4.815494 -1.713300 -1.583680
H 2.545580 -0.660304 1.914364
H 6.630594 -2.527562 -0.157323
H 4.374225 -1.472079 3.339965
H -1.502815 0.030143 0.123077
H 0.893754 -5.106343 -1.159817
H 6.437526 -2.413337 2.317431
H 4.538767 0.933847 -0.760374
H 5.880575 2.941206 -0.610650

H 4.853509 5.115206 0.039553
H 2.429162 5.224407 0.527306
H -2.380853 1.364703 -1.565588
H -2.586665 -0.273268 -2.226953
H -3.370086 1.111783 -3.004397
C -2.911877 0.162712 2.125297
H -3.052961 1.148756 2.591772
H -1.904731 -0.167809 2.396575
C -5.328312 -0.309696 2.231107
H -5.647695 0.529273 2.861802
H -6.078783 -1.099367 2.341024
C -3.958336 -0.806410 2.662564
H -3.771138 -1.805804 2.257866
H -3.919554 -0.874593 3.752458
C -6.687854 0.222223 0.259780
H -7.307991 0.787054 0.963482
H -7.114720 -0.787153 0.192699
C -5.550019 0.304657 -1.929147
H -5.488883 0.792765 -2.902133
H -5.706568 -0.768891 -2.108415
C -6.691423 0.881487 -1.107885
H -7.650562 0.700878 -1.597826
H -6.557792 1.962983 -1.015480

Second optimized geometry

SCF energy: -1459.13259514 Hartree

distance $N_{photocat}$—H = 2.0392 Å;

distance N_{MeTBD}—H = 1.0235 Å;

cartesian coordinates:

N -4.393197 0.429905 -1.274321
C -4.427291 0.195205 0.055999
N -5.602988 0.095471 0.707860
N -3.274747 0.071346 0.735380
C -3.121620 0.482781 -1.996878
N -0.366119 -0.269566 0.030632
C 0.498108 0.781540 0.065727
C 1.872115 0.712067 -0.269733
C 2.454237 -0.616302 -0.783356
C 1.443544 -1.764634 -0.656529
C 0.099512 -1.513707 -0.289201
C 2.659723 1.912017 -0.190991
C 2.044203 3.141655 0.228750
C 0.658452 3.160323 0.563720
C -0.082575 2.027119 0.484462
C -0.822284 -2.591172 -0.232947
C -0.426913 -3.880227 -0.531343
C 0.903701 -4.126067 -0.894811
C 1.817962 -3.076226 -0.951695
C 2.805801 4.329224 0.306693

C 4.145053 4.340760 -0.017014
C 4.765356 3.144670 -0.428498
C 4.048476 1.971238 -0.510334
C 2.725793 -0.450376 -2.312070
C 3.706562 -1.034139 0.025492
C 4.876996 -1.502428 -0.578584
C 5.963453 -1.921407 0.193564
C 5.896303 -1.882044 1.583581
C 4.730480 -1.420986 2.197895
C 3.649768 -1.005017 1.425685
H -1.843794 -2.378630 0.058149
H 0.209787 4.094978 0.881813
H -1.134261 2.030097 0.741674
H -1.139563 -4.695151 -0.481886
H 2.844296 -3.291768 -1.223608
H 1.796648 -0.166447 -2.809897
H 3.467763 0.321657 -2.514588
H 3.064197 -1.388353 -2.753619
H 4.963685 -1.537576 -1.657306
H 2.751261 -0.650382 1.919095
H 6.862003 -2.276572 -0.298685
H 4.662726 -1.385274 3.279572
H -2.374508 0.004903 0.253011
H 1.227796 -5.133335 -1.129124
H 6.739899 -2.205520 2.182617
H 4.570599 1.078214 -0.812255
H 5.819689 3.143553 -0.680508
H 4.717949 5.258574 0.045899
H 2.310818 5.239280 0.628171
H -2.419225 1.155215 -1.503893
H -2.665189 -0.506330 -2.096593
H -3.316303 0.874494 -2.992726
C -5.635398 0.409961 -2.060973
H -5.447385 0.964815 -2.979166
H -5.893069 -0.619546 -2.336639
C -6.753515 1.056334 -1.260295
H -6.517589 2.110125 -1.089943
H -7.692223 1.007063 -1.814448
C -6.905683 0.325928 0.061566
H -7.517439 0.906078 0.756603
H -7.396404 -0.642620 -0.083412
C -5.688967 -0.266790 2.135538
H -5.901400 0.639671 2.712787
H -6.545221 -0.936139 2.244633
C -3.224860 -0.105848 2.188311
H -2.279406 -0.595888 2.419426
H -3.228693 0.869741 2.686375
C -4.418275 -0.936572 2.632617
H -4.336769 -1.947837 2.224868
H -4.450625 -1.012455 3.720669

Comment

In the case of the adduct between **5a** and MeTBD, two PESs were taken into account: the first one is referred to the electronic ground state of the neutral singlet, the second one to the electronic ground state of the cationic doublet. The picture is similar to the one proposed for the adduct between **5a** and TMG (compare figures 1 and 2): in the case of the neutral singlet $E_{H\text{-photocat}} < E_{H\text{-MeTBD}}$ (lowest energy associated to the protonated form of the photocatalyst, with ΔE=8.38 kcal/mol), while for the cationic doublet $E_{H\text{-photocat}} > E_{H\text{-MeTBD}}$ (lowest energy associated to the deprotonated form of the photocatalyst, with ΔE=11.24 kcal/mol). The situation is schematically depicted in Figure S93. These results support the hypothesis of a coupling between the oxidation and the deprotonation of the photocatalyst.

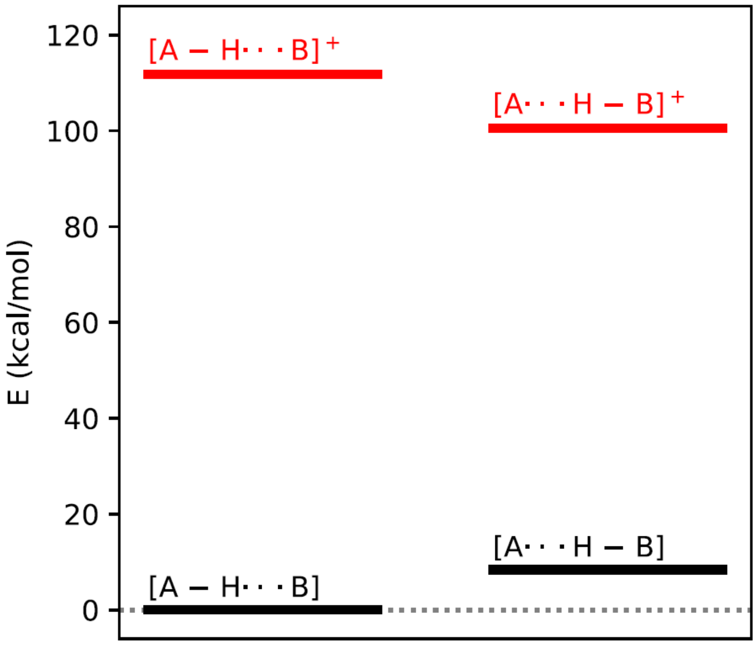

Figure S93: Energies of the four minima of the PESs discussed in the text. Energies are given in kcal/mol, relative to the smallest calculated value. Different colors are referred to different PESs: black for the ground state of the neutral singlet and red for the ground state of the cationic doublet (i.e., the oxidized electronic state).

Estimation of the pK_a values of the N-H group in PC 5a

Estimation of the pK_a values of the N-H group in photocatalyst **5a** were accomplished using a relative determination method by evaluating the difference in energy of the relative minima in the adducts with MeTBD, where the proton was deliberately positioned on the photocatalyst and on the MeTBD base, and considering the experimental pK_a value for the MeTBDH$^+$/MeTBD couple, pK_a(MeTBDH+/MeTBD) = 25.4 in acetonitrile.

For the ground state 5a:

Adduct of 5a with MeTBD, with the proton located on the PC (releative minimum)	Adduct of 5a with MeTBD, with the proton located on the base (releative minimum)
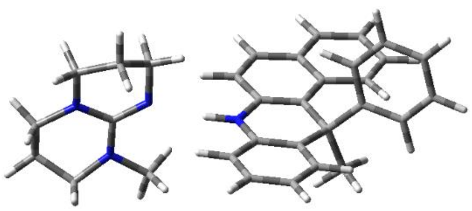	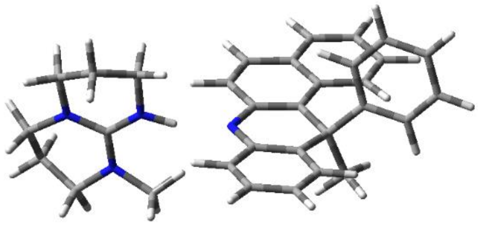
SCF Energy = -1459.29290999 Hartree	SCF Energy = -1459.27954856 Hartree

distance H-N(**5a**) = 1.0321 Å<br>distance H-N(MeTBD) = 1.9242 Å	distance H-N(**5a**) = 1.7841 Å<br>distance H-N(MeTBD) = 1.0544 Å<br>**relative energy = +8.38 Kcal/mol**

*Table S8: Energies and N-H distances relative to the two relative minima in the adduct between **5a** and MeTBD.*

Thus:

ΔG^0 = -2.303 RT [pK_a(MeTBDH$^+$/MeTBD) – pK_a(**5a**)] ~ 8.38 Kcal/mol

with RT = 0.593 Kcal/mol and pK_a(MeTBDH$^+$/MeTBD) = 25.4 in acetonitrile it results:

pK_a(**5a**) ~ 31.5

For the oxidized state 5a$^{\cdot+}$:

Adduct of 5a$^{\cdot+}$ with MeTBD, with the proton located on the PC (releative minimum)	Adduct of 5a$^{\cdot+}$ with MeTBD, with the proton located on the base (releative minimum)
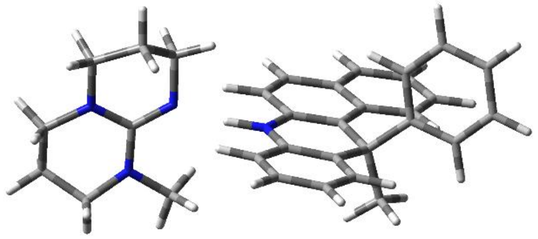	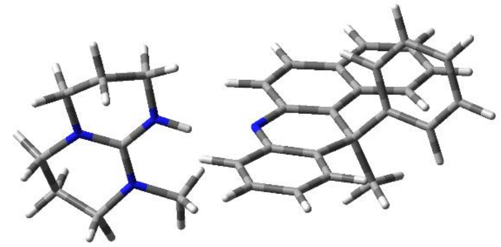
SCF Energy = -1459.11467872 Hartree<br>distance H-N(**5a**$^{\cdot+}$) = 1.1035 Å<br>distance H***N(MeTBD) = 1.5918 Å	SCF Energy = -1459.13259514 Hartree<br>distance H***N(**5a**$^{\cdot+}$) = 2.0392 Å<br>distance H-N(MeTBD) = 1.0235 Å<br>**relative energy = -11.24 Kcalmol^{-1}**

*Table S9: Energies and N-H distances relative to the two relative minima in the adduct between **5a**$^{\cdot+}$ and MeTBD.*

ΔG^0 = -2.303 RT [pK_a(MeTBDH$^+$/MeTBD) - pK_a(**5a**$^{\cdot+}$)] ~ -11.24 Kcal/mol

with RT = 0.593 Kcal/mol and pK_a(MeTBDH$^+$/MeTBD) = 25.4 in acetonitrile it results:

pK_a(**5a**$^{\cdot+}$) ~ 17.2

UV-Vis absorption of 5a in the presence of MeTBD

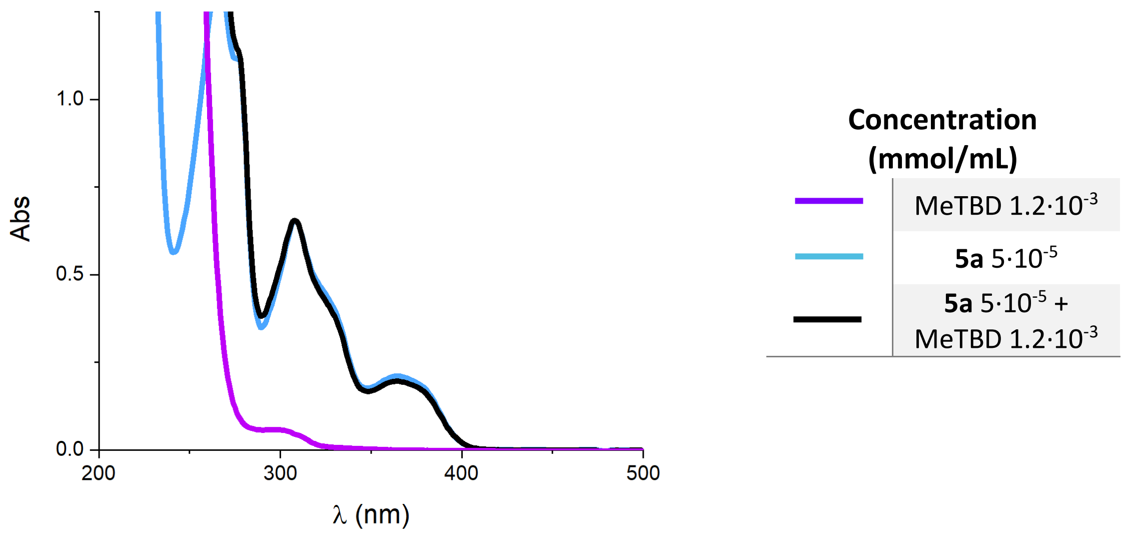

*Figure S94: Superimposed UV-Vis absorption of **5a** 5·10^{-5} M (blue trace), MeTBD 1.2·10^{-3} M (purple trace) and **5a** 5·10^{-5} M in the presence of MeTBD 1.2·10^{-3} M (black trace). Recorded in MeCN in quartz cuvettes (1 cm optical path).*

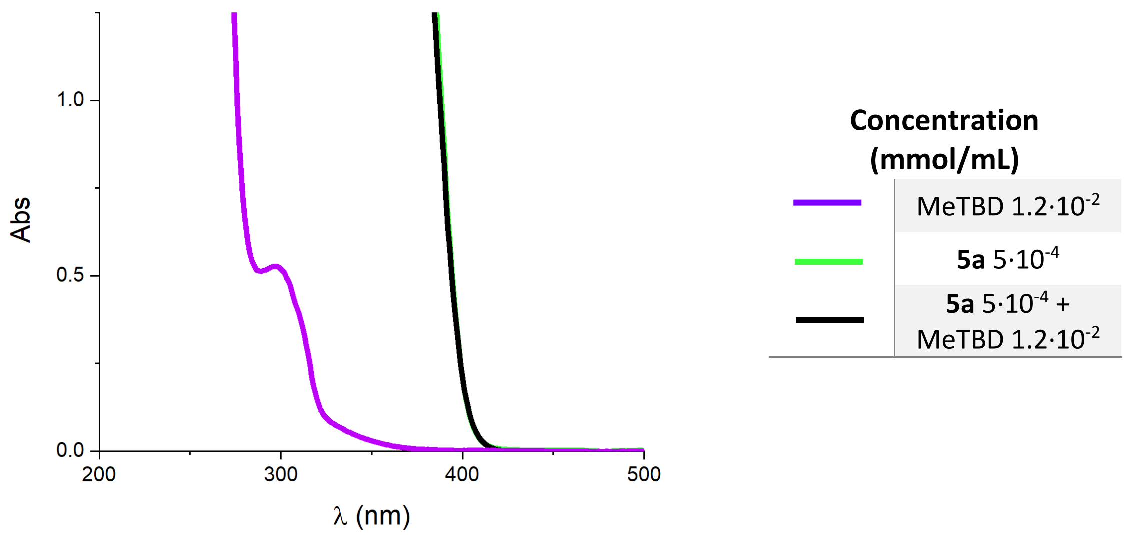

*Figure S95: Superimposed UV-Vis absorption of **5a** 5·10^{-4} M (green trace), MeTBD 1.2·10^{-2} M (purple trace) and **5a** 5·10^{-4} M in the presence of MeTBD 1.2·10^{-2} M (black trace). The black and green traces are superimposed. Recorded in MeCN in quartz cuvettes (1 cm optical path).*

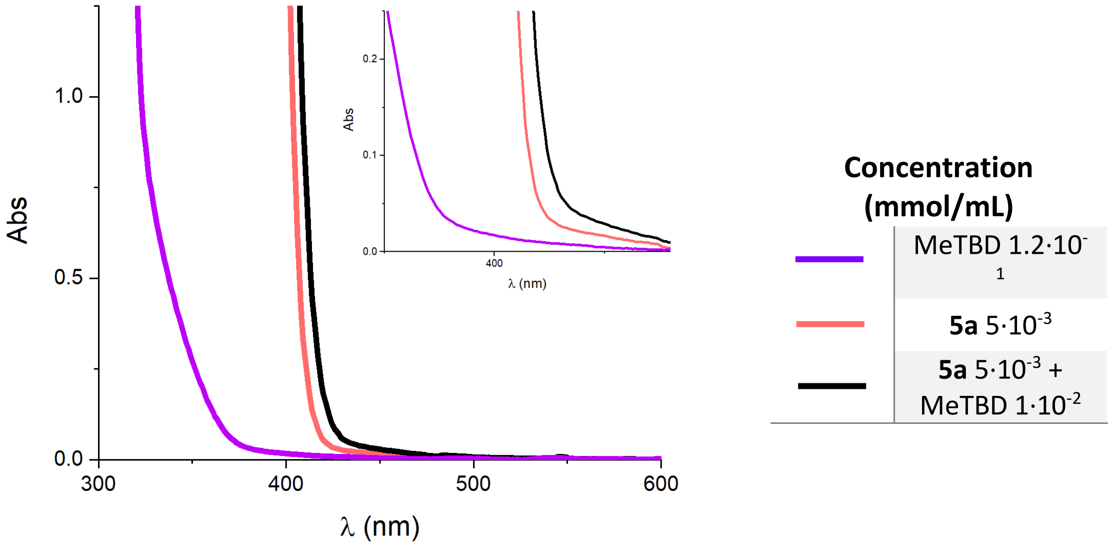

*Figure S96: Superimposed UV-Vis absorption of **5a** $5{\cdot}10^{-3}$ M (red trace), MeTBD $1.2{\cdot}10^{-1}$ M (purple trace) and **5a** $5{\cdot}10^{-3}$ M in the presence of MeTBD $1{\cdot}10^{-1}$ M (black trace). Inset: enlargement showing a redshift of the absorption band of about 10 nm. Recorded in MeCN in quartz cuvettes (1 cm optical path).*

Fluorescence emission of 5a in the presence of MeTBD

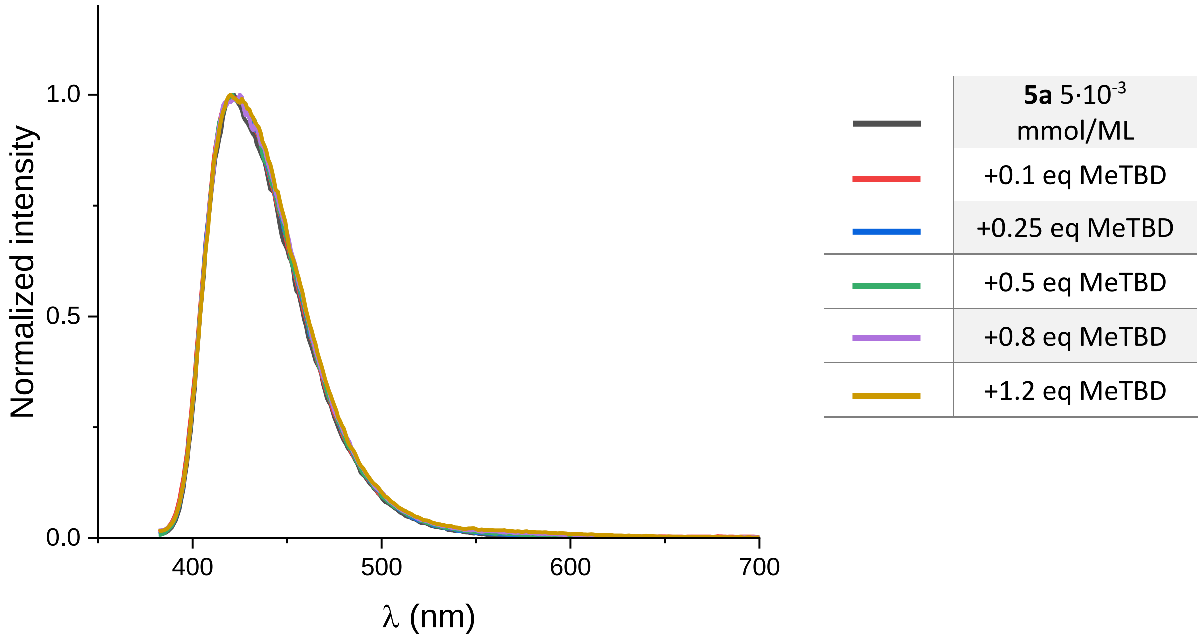

Figure S97: Superimposed fluorescence emission of 5a 5·10-3 M in the presence of different amount of MeTBD. Recorded in MeCN in quartz cuvettes (1mm optical path placed inside the cuvette cell with an angle of 45 degrees).

NMR Titrations

Titration with MeTBD

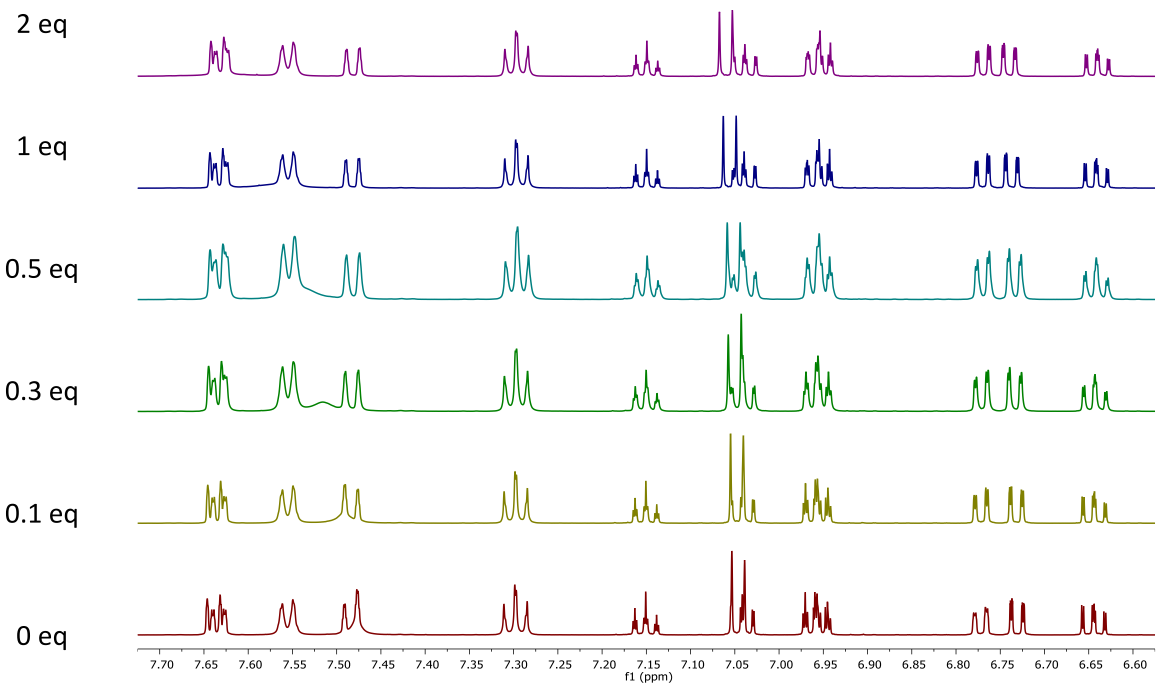

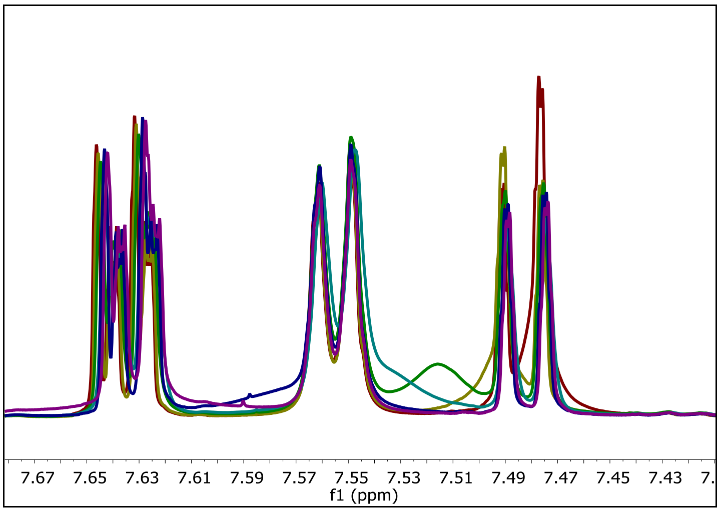

*Figure S98: Top: NMR spectra of **5a** in the presence of increasing amounts of MeTBD (only aromatic region shown). Conditions: **5a** 0.01 M in CD_3CN (red trace), **5a** 0.01 M and MeTBD 0.001 M in CD_3CN (yellow trace), **5a** 0.01 M and MeTBD 0.003 M in CD_3CN (green trace), **5a** 0.01 M and MeTBD 0.005 M in CD_3CN (blue trace), **5a** 0.01 M and MeTBD 0.01 M in CD_3CN (indigo trace), **5a** 0.01 M and MeTBD 0.02 M in CD_3CN (violet trace). Bottom:* Enlargement from 7.43ppm to 7.67ppm.

Titration with TMG

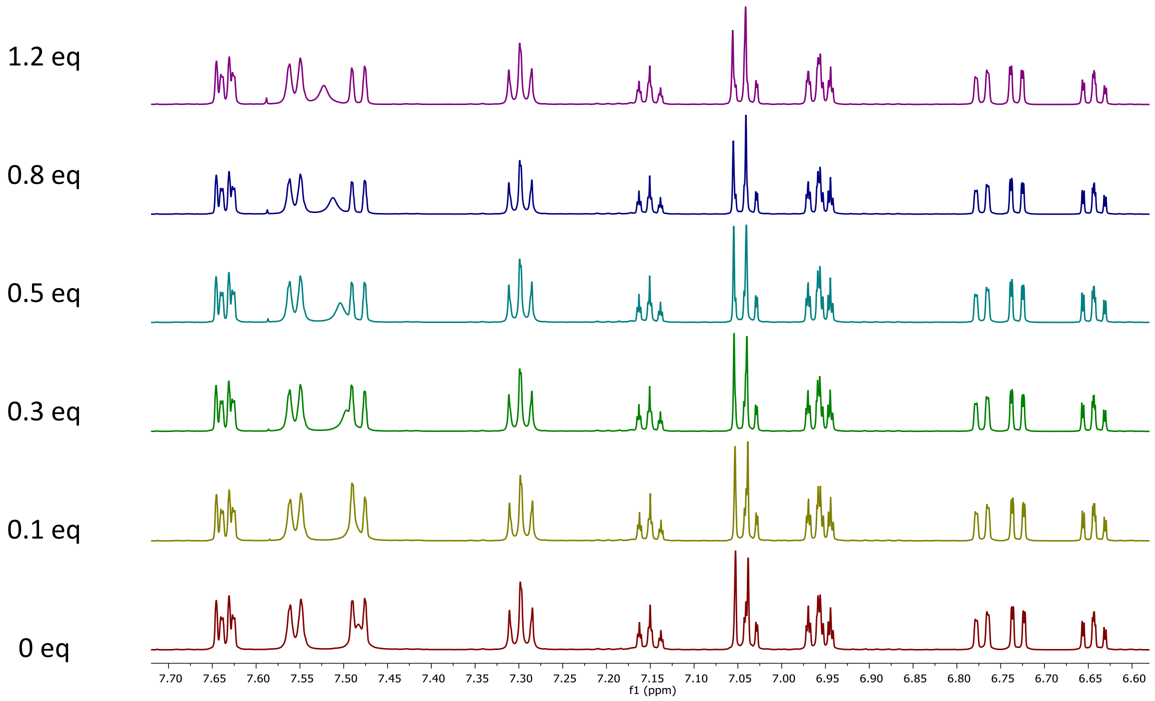

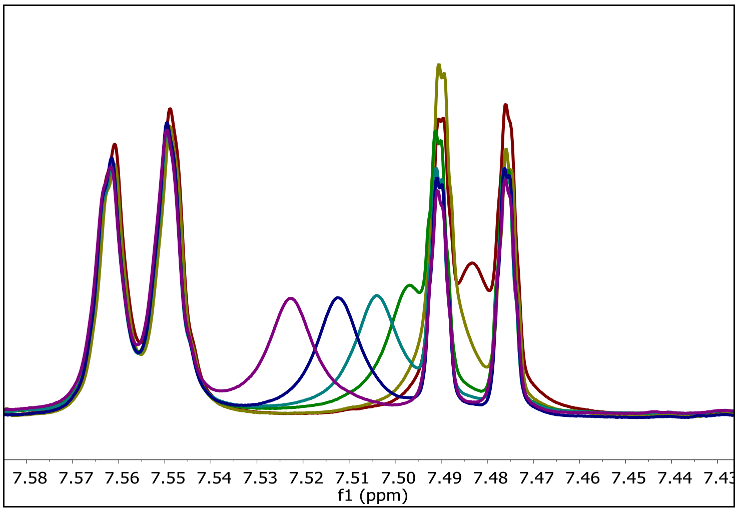

*Figure S99: Top: NMR spectra of **5a** in the presence of increasing amounts of TMG (only aromatic region shown). Conditions: **5a** 0.01 M in CD_3CN (red trace), **5a** 0.01 M and TMG 0.001 M in CD_3CN (yellow trace), **5a** 0.01 M and TMG 0.003 M in CD_3CN (green trace), **5a** 0.01 M and TMG 0.005 M in CD_3CN (blue trace), **5a** 0.01 M and TMG 0.008 M in CD_3CN (indigo trace), **5a** 0.01 M and TMG 0.012 M in CD_3CN (violet trace). Bottom:* Enlargement from 7.43ppm to 7.58ppm.

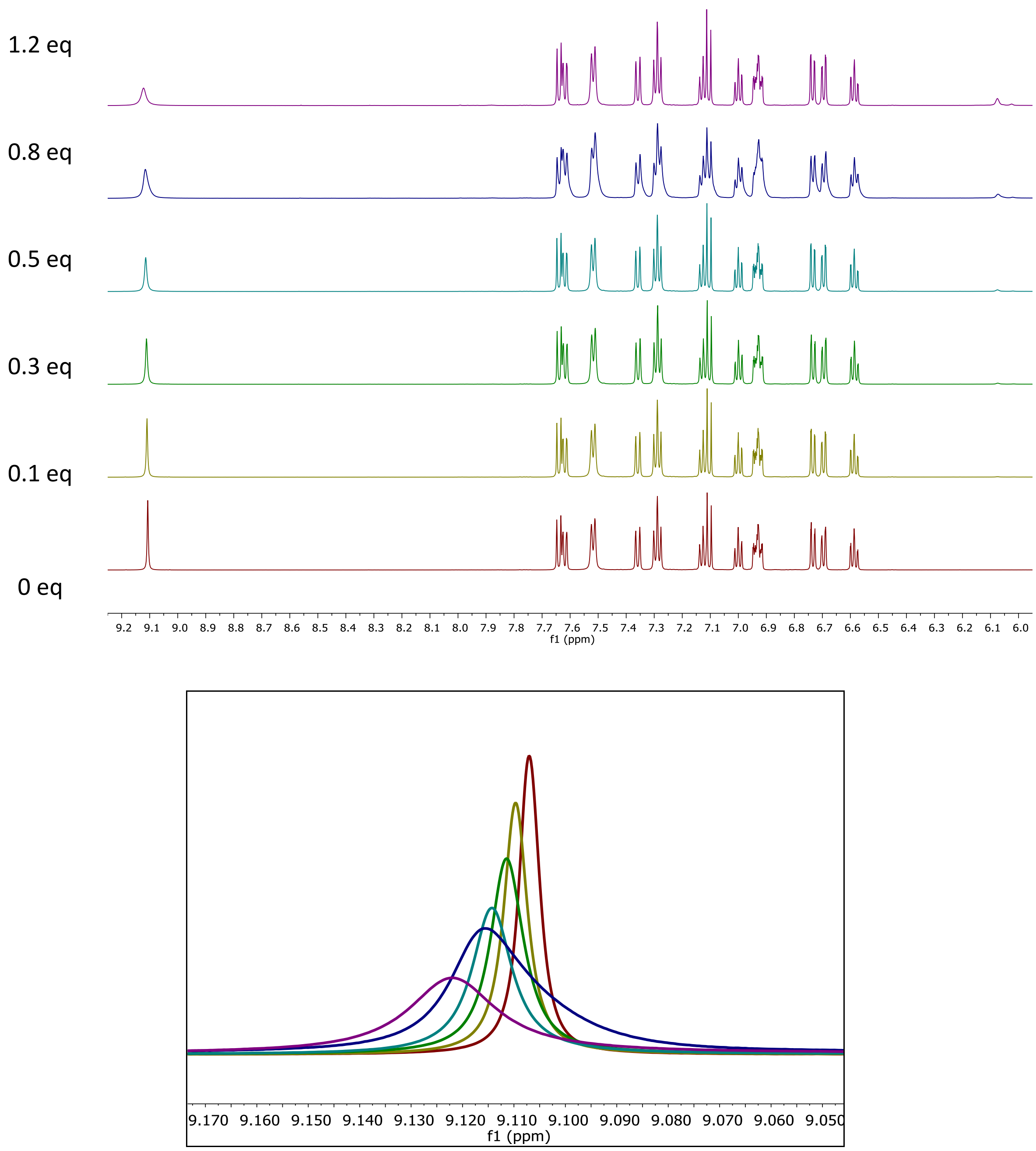

*Figure S100: Top: NMR spectra of **5a** in the presence of increasing amounts of TMG (only aromatic region shown). Conditions: **5a** 0.01 M in DMSO-d$_6$ (red trace), **5a** 0.01 M and TMG 0.001 M in DMSO-d$_6$ (yellow trace), **5a** 0.01 M and TMG 0.003 M in DMSO-d$_6$ (green trace), **5a** 0.01 M and TMG 0.005 M in DMSO-d$_6$ (blue trace), **5a** 0.01 M and TMG 0.008 M in DMSO-d$_6$ (indigo trace), **5a** 0.01 M and TMG 0.012 M in DMSO-d$_6$ (violet trace). Bottom:* Enlargement from 9.05ppm to 9.17ppm.

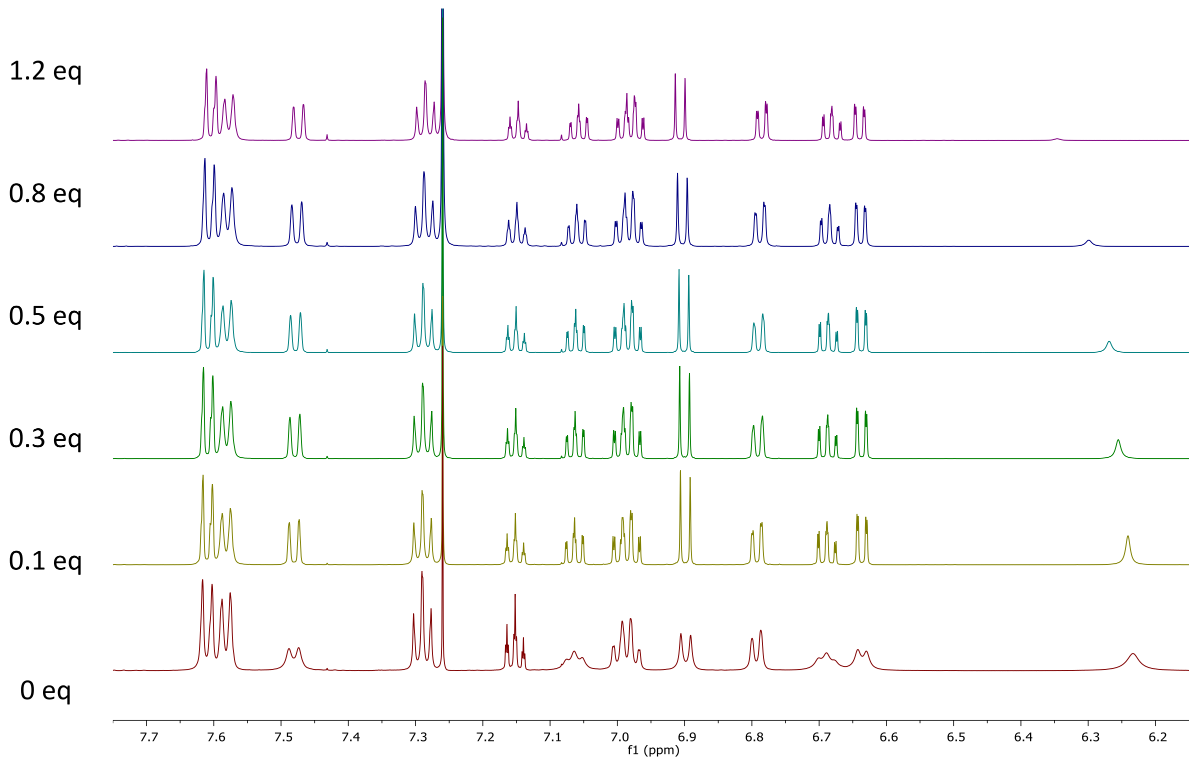

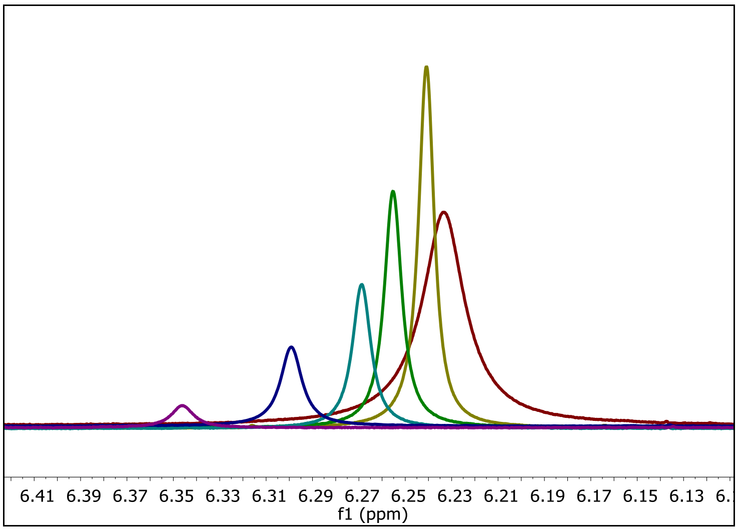

*Figure S101A: Top NMR spectra of **5a** in the presence of increasing amounts of TMG (only aromatic region shown). Conditions: **5a** 0.01 M in $CDCl_3$ (red trace), **5a** 0.01 M and TMG 0.001 M in $CDCl_3$ (yellow trace), **5a** 0.01 M and TMG 0.003 M in $CDCl_3$ (green trace), **5a** 0.01 M and TMG 0.005 M in $CDCl_3$ (blue trace), **5a** 0.01 M and TMG 0.008 M in $CDCl_3$ (indigo trace), **5a** 0.01 M and TMG 0.012 M in $CDCl_3$ (violet trace). Bottom: Enlargement from 6.12 to 6.41 ppm.*

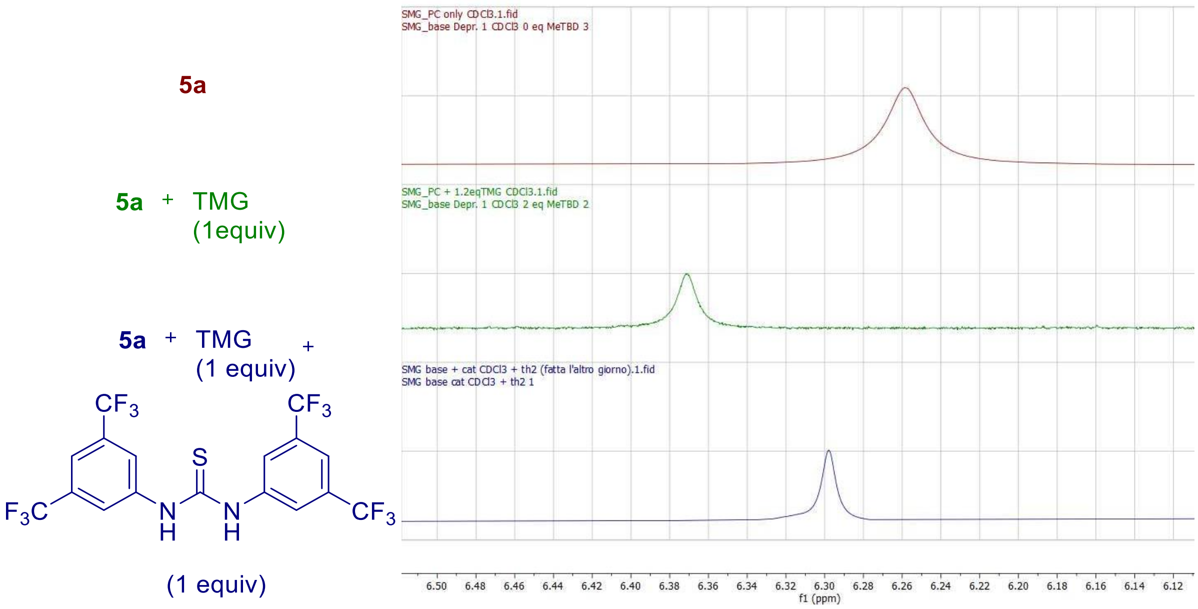

*Figure S101B: NMR spectra of **5a** in the presence of TMG and Schreiner's Thiourea (enlargement). Conditions: **5a** 0.01 M in $CDCl_3$ (red trace), **5a** 0.01 M and TMG 0.01 M in $CDCl_3$ (green trace), **5a** 0.01 M, TMG 0,01 M and Schreiner's Thiourea 0.01 M in $CDCl_3$ (blue line).*

Cyclic Voltammetry with the presence of a base

CV with MeTBD

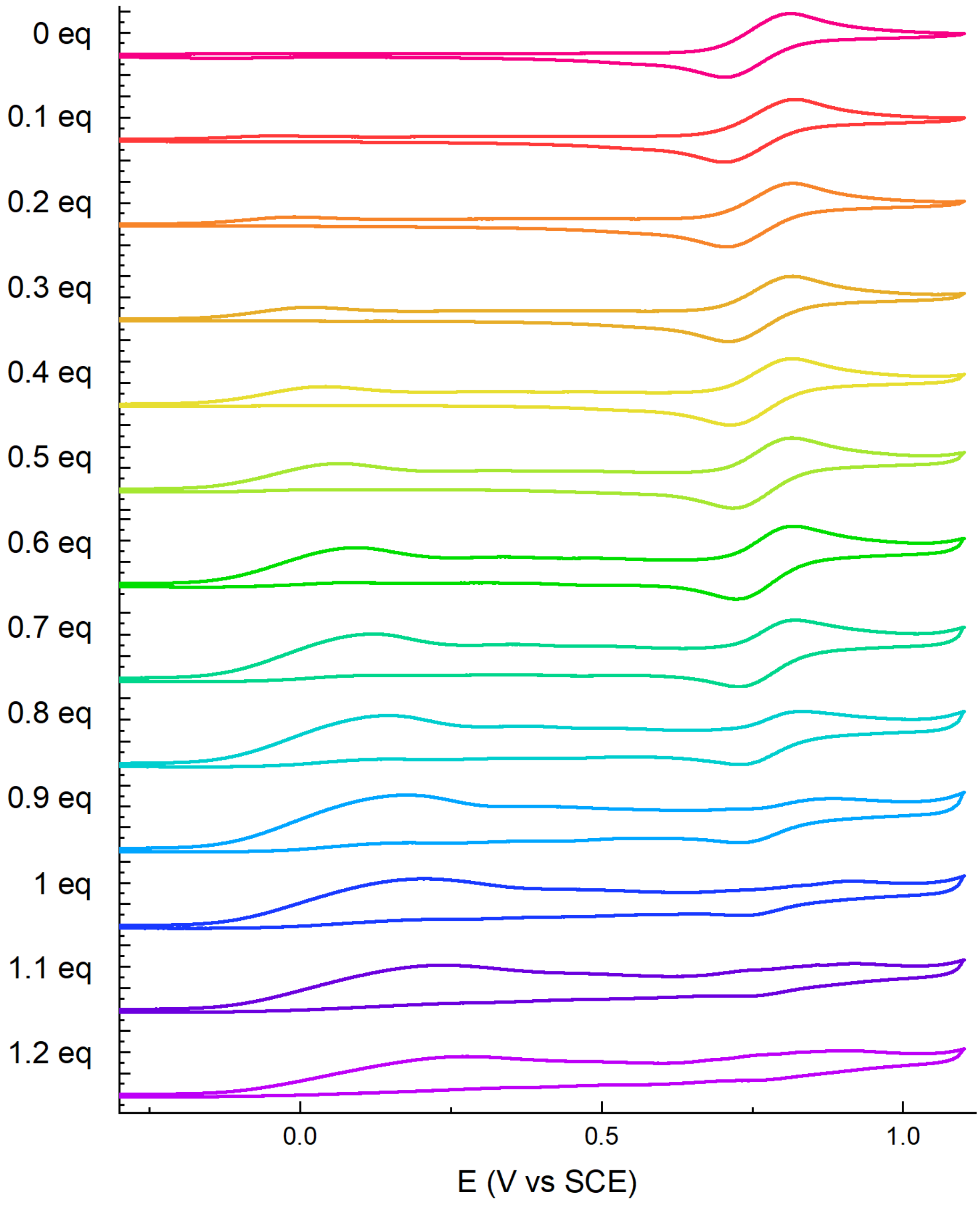

*Figure S102: Cyclic voltammograms of **5a** 5·10^{-3} M with increasing amounts of MeTBD. Recorded with GC as the working electrode in MeCN with 0.1 M [Bu_4N][PF_6] as the supporting electrolyte at 0.1 V/s scan rate. Equivalents of MeTBD are referred to the molar ratio between the amount of MeTBD and the initial concentration of **5a** (5·10^{-3} M).*

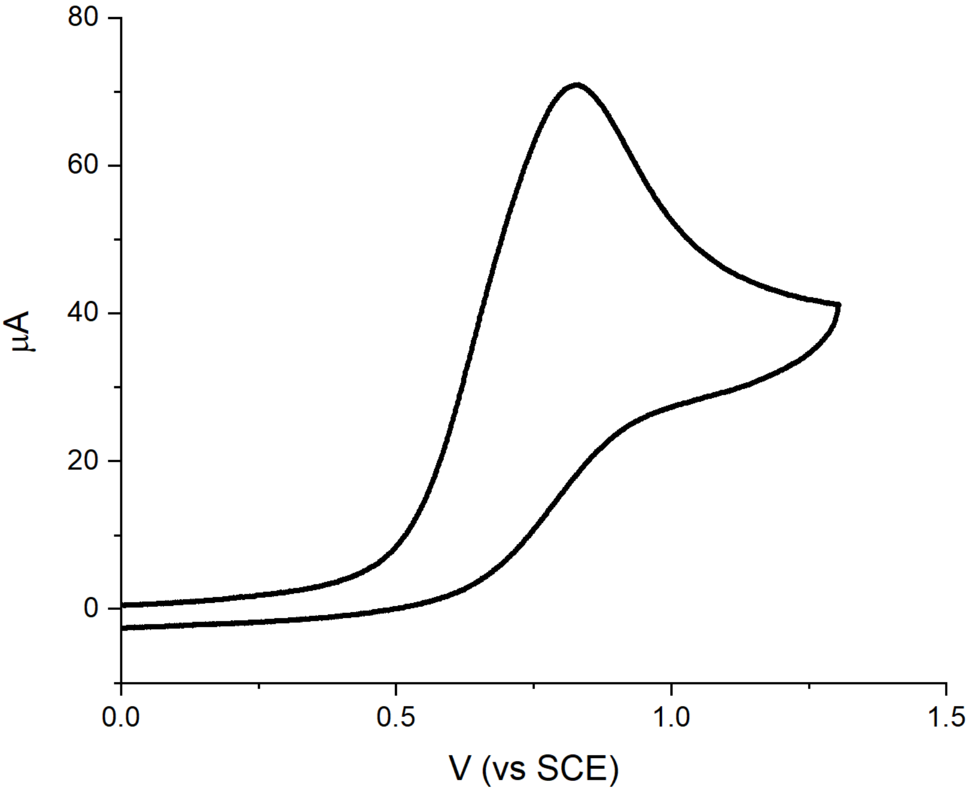

Figure S103: Cyclic voltammogram of MeTBD 5·10^{-3} M. Recorded with GC as the working electrode in MeCN with 0.1 M [Bu_4N][PF_6] as the supporting electrolyte at 0.1 V/s scan rate.

CV with TBD

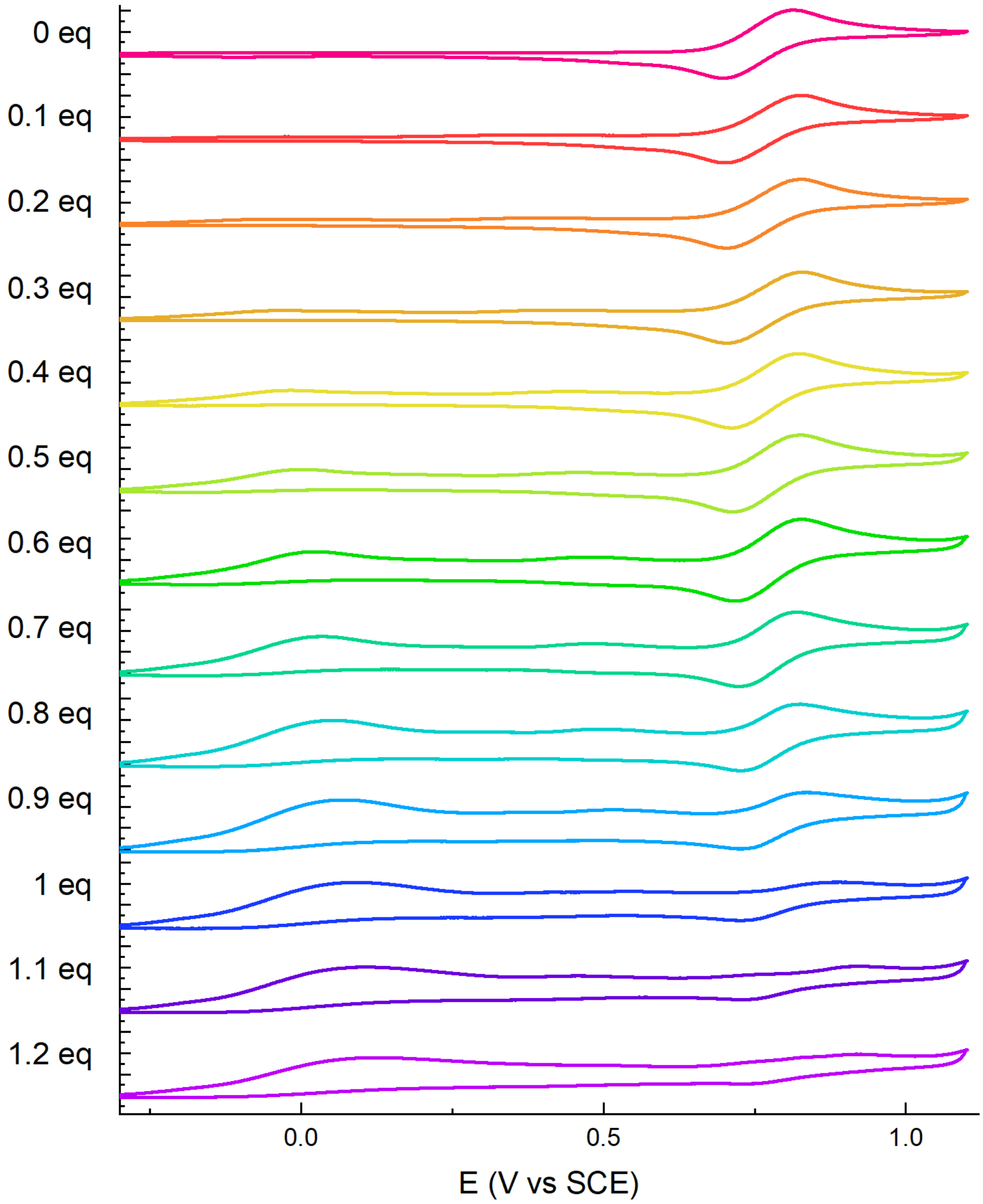

*Figure S104: Cyclic voltammograms of **5a** 5·10^{-3} M with increasing amounts of TBD. Recorded with GC as the working electrode in MeCN with 0.1 M [Bu_4N][PF_6] as the supporting electrolyte at 0.1 V/s scan rate. Equivalents of TBD are referred to the molar ratio between the amount of TBD and the initial concentration of **5a** (5·10^{-3} M).*

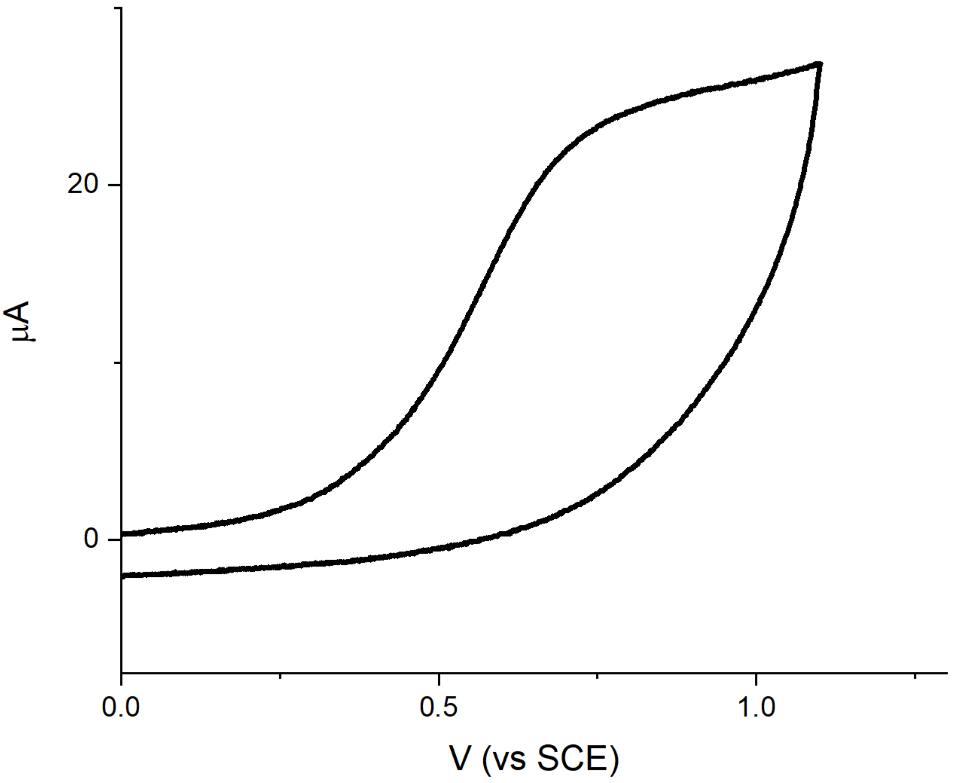

Figure S105: Cyclic voltammogram of TBD 5·10^{-3} M. Recorded with GC as the working electrode in MeCN with 0.1 M [Bu_4N][PF_6] as the supporting electrolyte at 0.1 V/s scan rate.

CV with TMG

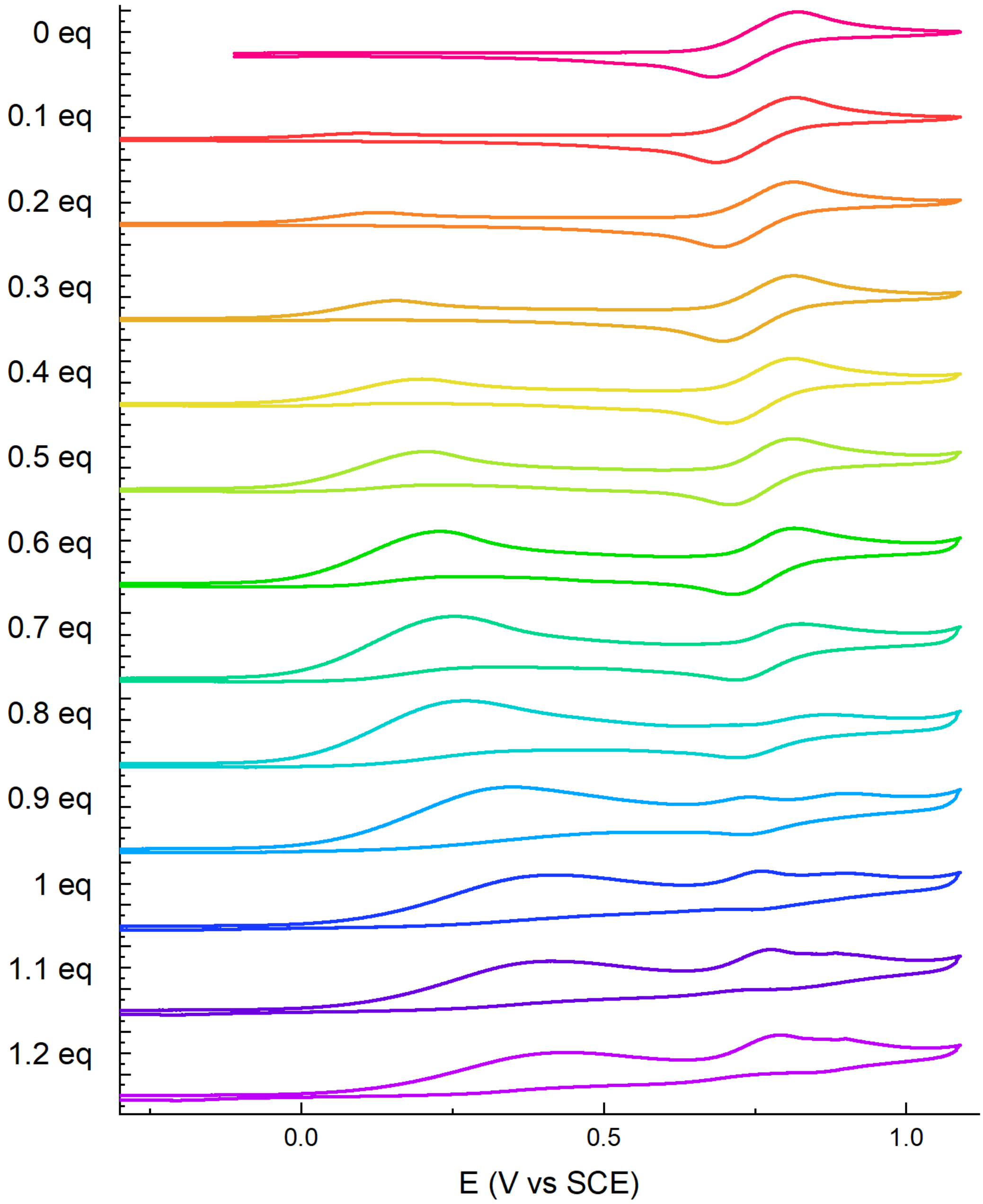

*Figure S106: Cyclic voltammograms of **5a** $5 \cdot 10^{-3}$ M with increasing amounts of TMG. Recorded with GC as the working electrode in MeCN with 0.1 M $[Bu_4N][PF_6]$ as the supporting electrolyte at 0.1 V/s scan rate. Equivalents of TMG are referred to the molar ratio between the amount of TMG and the initial concentration of **5a** ($5 \cdot 10^{-3}$ M).*

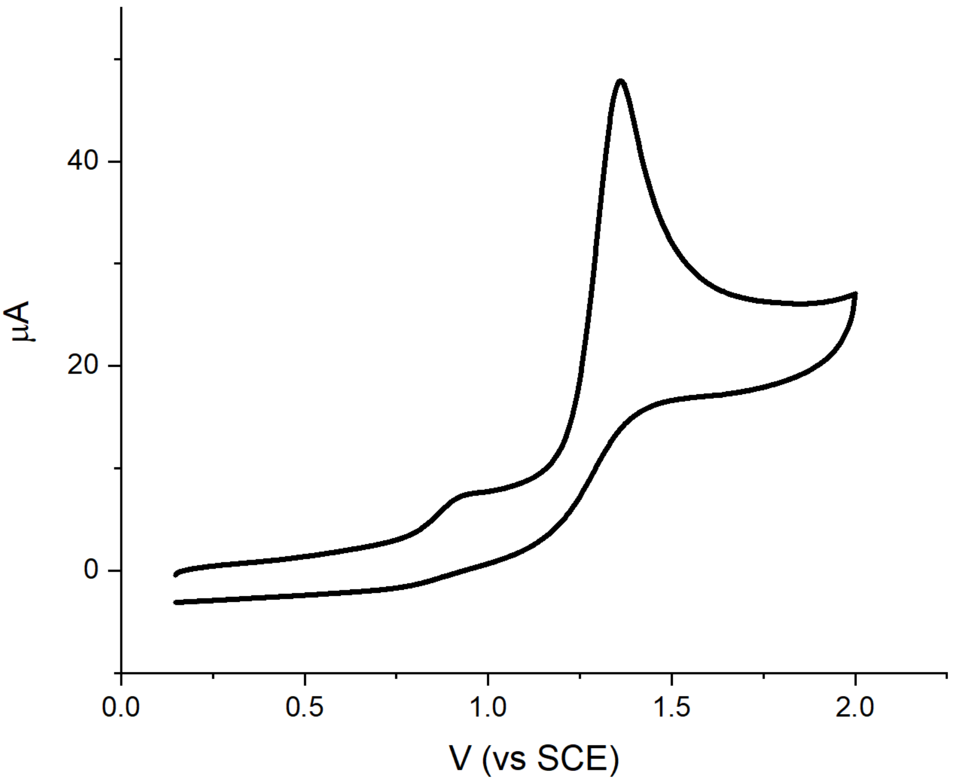

Figure S107: Cyclic voltammogram of TMG 5·10^{-3} M. Recorded with GC as the working electrode in MeCN with 0.1 M [Bu_4N][PF_6] as the supporting electrolyte at 0.1 V/s scan rate.

CV with DBU

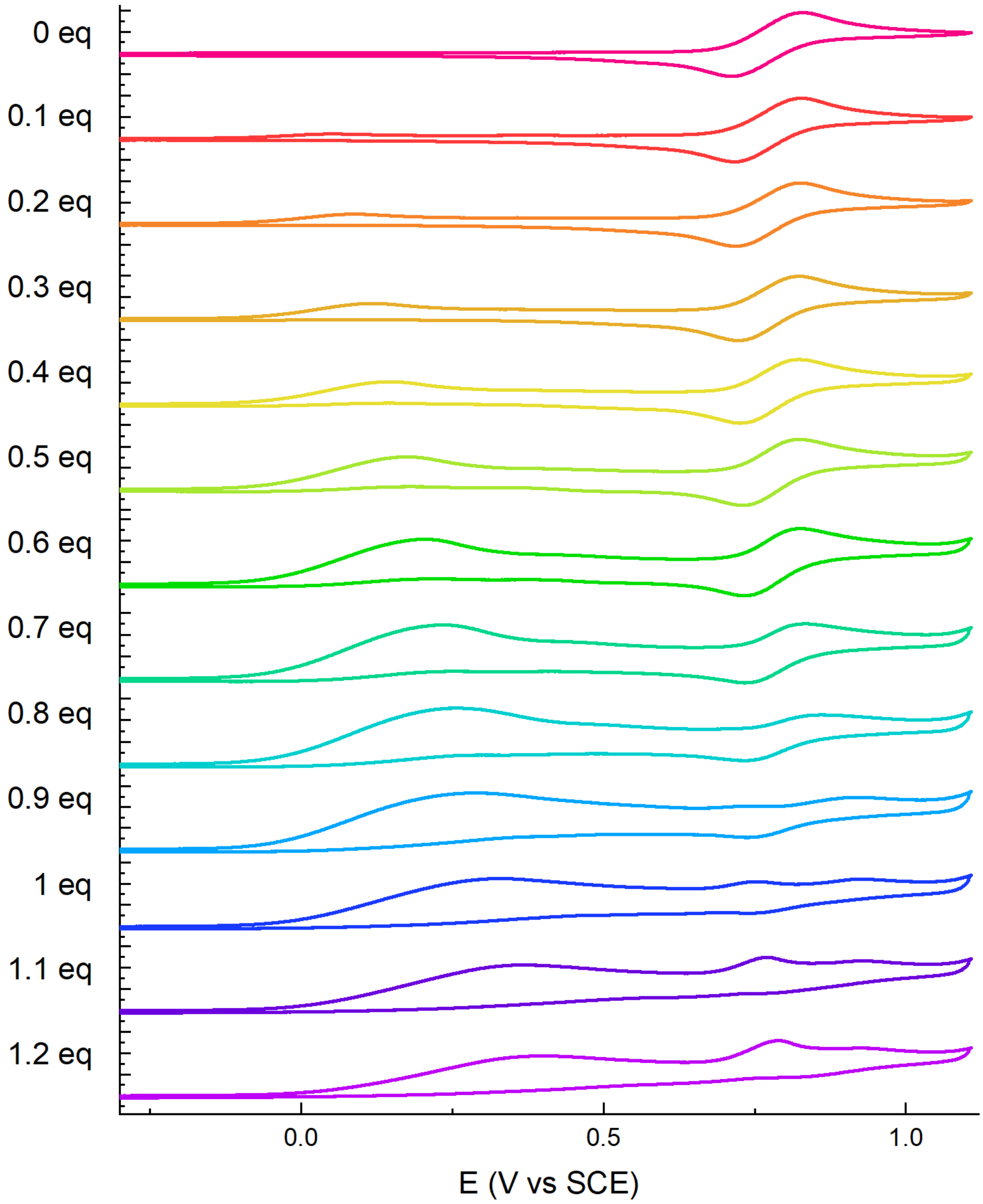

*Figure S108: Cyclic voltammogram of **5a** $5{\cdot}10^{-3}$ M with increasing amounts of DBU. Recorded with GC as the working electrode in MeCN with 0.1 M $[Bu_4N][PF_6]$ as the supporting electrolyte at 0.1 V/s scan rate. Equivalents of DBU are referred to the molar ratio between the amount of DBU and the initial concentration of **5a** ($5{\cdot}10^{-3}$ M).*

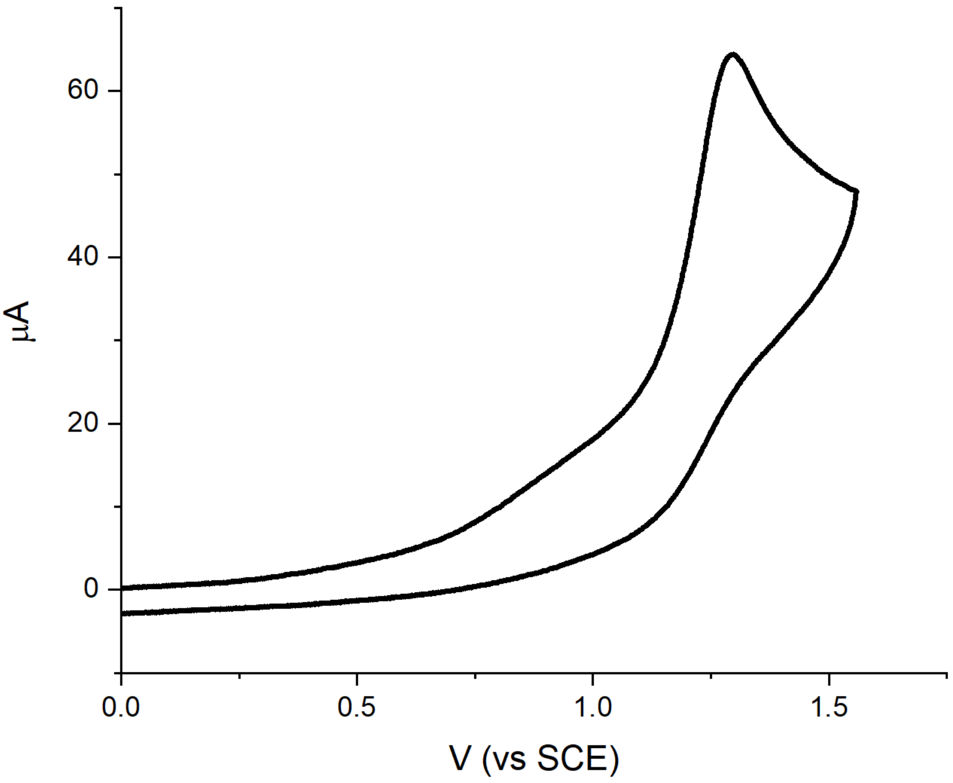

Figure S109: Cyclic voltammogram of DBU 5·10^{-3} M. Recorded with GC as the working electrode in MeCN with 0.1 M $[Bu_4N][PF_6]$ as the supporting electrolyte at 0.1 V/s scan rate.

CV with DBN

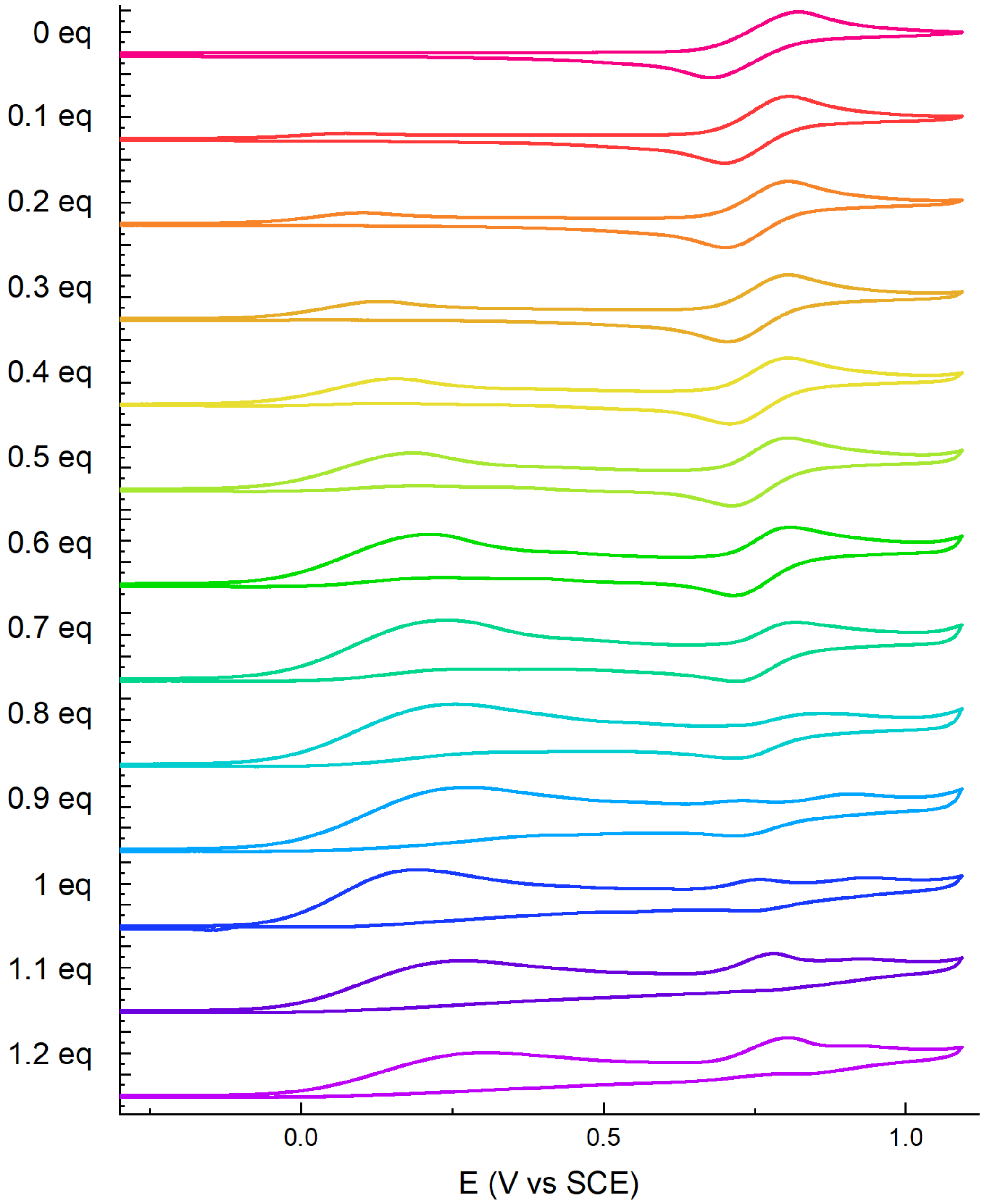

*Figure S110: Cyclic voltammogram of **5a** $5{\cdot}10^{-3}$ M with increasing amounts of DBN. Recorded with GC as the working electrode in MeCN with 0.1 M $[Bu_4N][PF_6]$ as the supporting electrolyte at 0.1 V/s scan rate. Equivalents of DBN are referred to the molar ratio between the amount of DBN and the initial concentration of **5a** ($5{\cdot}10^{-3}$ M).*

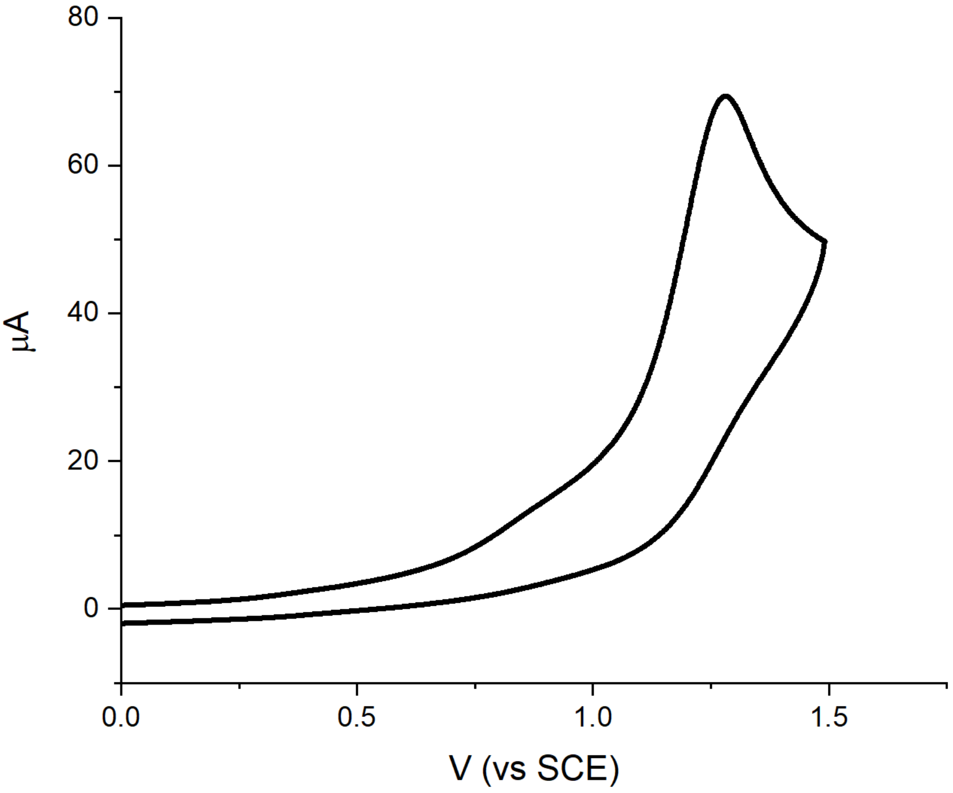

Figure S111: Cyclic voltammogram of DBN 5·10⁻³ M. Recorded with GC as the working electrode in MeCN with 0.1 M $[Bu_4N][PF_6]$ as the supporting electrolyte at 0.1 V/s scan rate.

Stern-Volmer quenching of 5a

The Stern-Volmer quenching study was performed by measuring the excited-state lifetime of PC **5a** $2 \cdot 10^{-5}$ M with increasing amounts of 4-chlorobenzonitrile **12** as the quencher. The ratio between τ_0 (excited-state lifetime of **5a** alone) and τ (excited-state lifetime of **5a** at a given quencher concentration) was plotted as a function of the concentration of quencher. $\lambda_{excitation}$ = 341.6 nm, $\lambda_{analysis}$ = 425 nm.

The data points were linearly fitted according to the Stern-Volmer equation:

$$\frac{\tau_0}{\tau} = 1 + K_{SV}[Q] = 1 + k_q \tau_0 [Q]$$

A value of K_{SV} = 65.6 M^{-1} was obtained and the value $k_q = 7.11 \cdot 10^9$ M^{-1} s^{-1} was hence calculated.

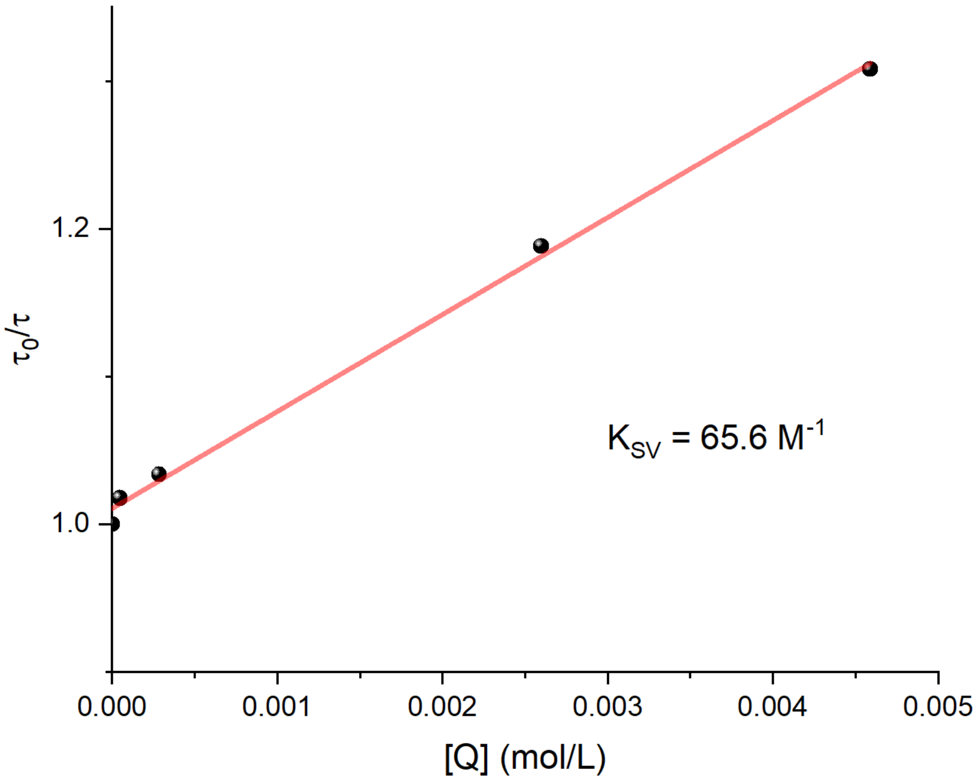

*Figure S112: Stern-Volmer plot of **5a** 2·10^-5 M with 4-chlorobenzonitrile **12** as the quencher Q (black dots) and linear fitting of the experimental data points (red line).*

Reaction quantum yield measurements

Actinometry

A ferrioxalate actinometry solution was prepared by following the Hammond variation of the Hatchard and Parker procedure outlined in Handbook of Photochemistry.[28] Ferrioxalate actinometer solution measures the decomposition of ferric ions to ferrous ions, which are complexed by 1,10-phenanthroline and monitored by UV/Vis absorbance at 510 nm. The moles of iron-phenanthroline complex formed are related to moles of photons absorbed. The following solutions were prepared:

A) 20 mL of 1,10-phenantroline 0.2% by weight in water
B) Buffer solution:
   a. 8.2 g $NaOAc \cdot H_2O$
   b. 1 mL concentrated H_2SO_4 (98%)
   c. Diluted to 100 mL with water
C) $Fe_2(SO_4)_3$ solution:
   a. 10 g $Fe_2(SO_4)_3 \cdot nH_2O$ (approximately 20% Fe by weight)
   b. 5.5 mL concentrated H_2SO_4 (98%)
   c. Diluted to 100 mL with water
D) 100 mL of $K_2C_2O_4$ 1.2 M in water

The actinometric solution ($K_3Fe(C_2O_4)_3$ solution) was prepared in the dark by mixing 0.5 mL of $Fe_2(SO_4)_3$ solution with 0.5 mL of $K_2C_2O_4$ solution in a volumetric flask and diluting to 10 mL. The $K_3Fe(C_2O_4)_3$ solution was stored in the dark. The actinometric measurements were done as follows:

1) 1 mL of the $K_3Fe(C_2O_4)_3$ solution was added to a quartz cuvette with 1 cm optical path
2) The cuvette was irradiated under stirring with a PR160L 427 nm Kessil lamp set at 25% of its maximum output power placed at a distance of 20 cm for increasing amount of time (5, 10, 20, 30 and 45 seconds). *Note: since the absorbance of the $K_3Fe(C_2O_4)_3$ solution at 427 nm is greater than 2, all the incident photons are absorbed, and no correction factors shall be applied in the following calculations*.
3) All the irradiated solution was transferred to a 10 mL volumetric flask, to which 2 mL of the 0.2% 1,10-phenantroline solution and 0.5 mL of the buffer solution were added. The flask was filled to the mark with water and the content thoroughly mixed.
4) A blank sample was prepared repeating step 3) with 1 mL $K_3Fe(C_2O_4)_3$ solution kept in the dark without irradiation.
5) The absorbances at 510 nm of the solutions prepared according to step 3) at increasing time intervals were measured and their difference with the blank ΔA was calculated (Figure S113). The absorbance at 510 nm of the blank was lower than A = 0.06 as recommended.

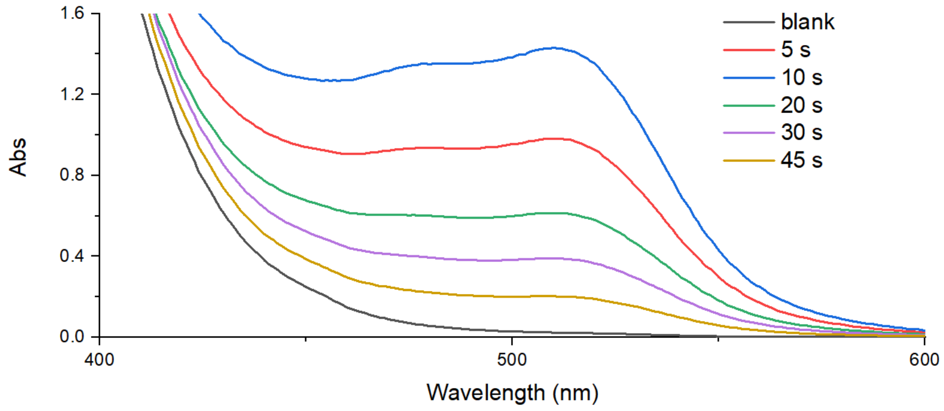

Figure S113: Absorbance spectra of the solutions prepared according to Step 3) at increasing time intervals.

The values of ΔA obtained were plotted as a function of the irradiation time (5, 10, 20, 30 and 45 seconds) and linearly interpolated with the following function (Figure S114):

$$\Delta A = \frac{\varepsilon b \varphi V_1 I}{V_2 V_3} t$$

Where:

ΔA are the values obtained at each time interval in Step 5)

b is the path length of the cuvette

ε is the extinction coefficient of Fe-1,10-phenantroline complex at 510 nm ($1.11 \cdot 10^4$ M^{-1} cm^{-1})

ϕ is the quantum yield of ferrous production at 427 nm (1.04)

V_1 is the volume in mL of irradiated $K_3Fe(C_2O_4)_3$ solution transferred to the 10 mL flask (1 mL)

V_2 is the volume in L of irradiated $K_3Fe(C_2O_4)_3$ solution (0.001 L)

V_3 is the volume in mL of the volumetric flask used for workup of irradiated aliquots (10 mL)

I is the photon flux in einsteins s^{-1} (moles of photons s^{-1})

t is the irradiation time in seconds

From the slope of the interpolation, the light intensity was thus calculated to be I = $2.67 \cdot 10^{-9}$ mol s^{-1}

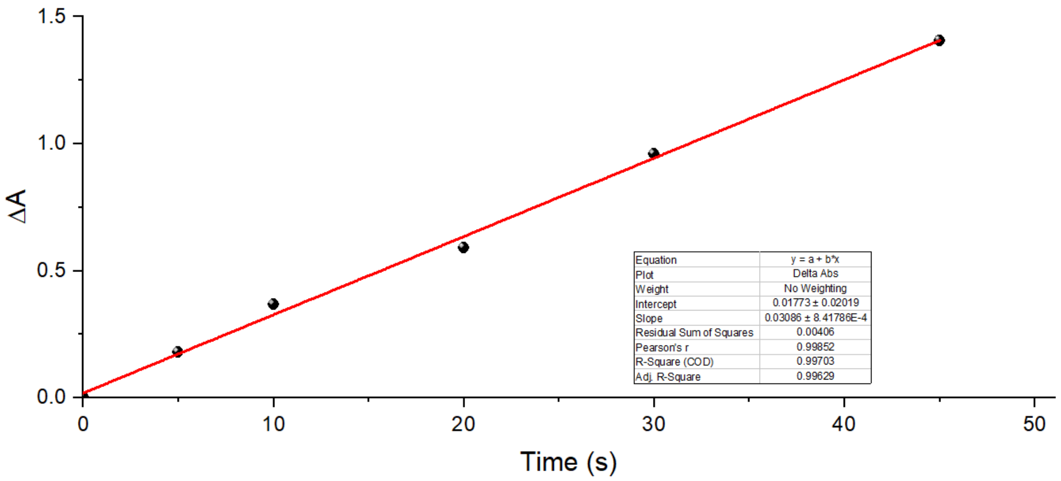

Figure S114: Plot and linear interpolation of the values of ΔA obtained according to Step 5) and their linear interpolation.

Quantum yield of the reduction of 27

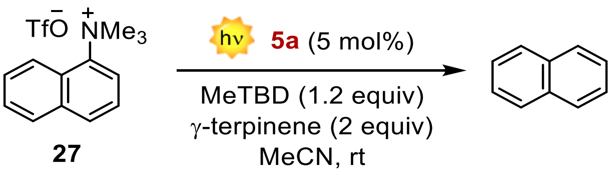

The same cuvette used for the actinometric measurements was charged with N,N,N-trimethylnaphthalen-1-aminium trifluoromethanesulfonate **27** (33.5 mg, 0.1 mmol) and PC **5a** (5 mol%) and degassed with Ar. 1 mL of already degassed MeCN was added, followed by γ-terpinene (32 µL, 0.2 mmol, 2 equiv) and MeTBD (1.2 mmol, 1.2 equiv). The reaction mixture was purged with Ar for 1 minute. The absorbance spectrum of the reaction mixture was recorded (Figure S115).

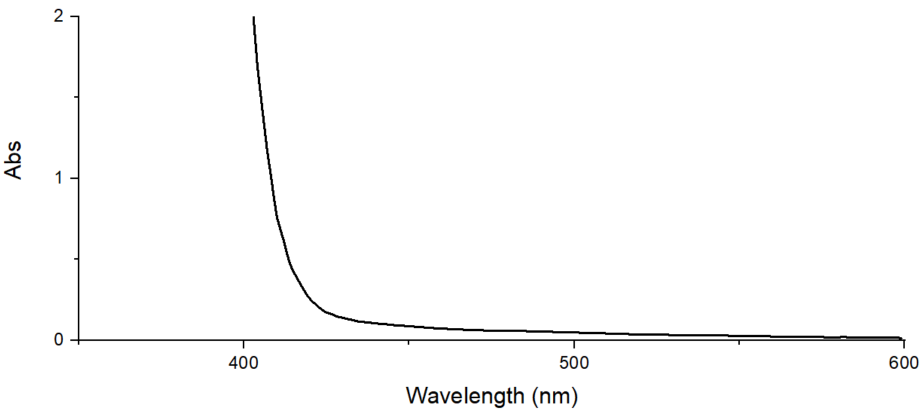

Figure S115: Absorbance spectrum of the reaction mixture.

The absorbance of the reaction mixture at 427 nm is A = 0.16. The fraction f of light absorbed can be calculated according to $f = 1\text{-}10^{-A} = 0.31$. Hence, the reaction mixture only absorbs 31% of the incident photons, and the effective photon flux is calculated correcting the photon flux obtained from actinometry according to $I_{eff} = f \cdot I = 0.31 \cdot 2.67 \cdot 10^{-9}$ mol s^{-1} = $8.28 \cdot 10^{-10}$ mol s^{-1}.

The cuvette containing the reaction mixture was then irradiated with the same setup as in Step 2) of the actinometric measurements. 50 µL aliquots of the reaction were sampled after 22, 47 and 52 hours of irradiation, diluted with 500 µL of $CDCl_3$ and analyzed by ^{1}H NMR to determine the consumption of the starting material **27** with CH_2Br_2 as the internal standard. The conversion (millimols of starting material **27** consumed) at given time intervals was plotted against the millimols of photons absorbed by the reaction mixture (obtained by multiplication of the effective photon flux I_{eff} with the irradiation time in seconds). The plot was linearly interpolated and the slope of the interpolation is, by definition, the quantum yield of the reaction (Figure S116).[19] The value of quantum yield is then ϕ = 0.15.

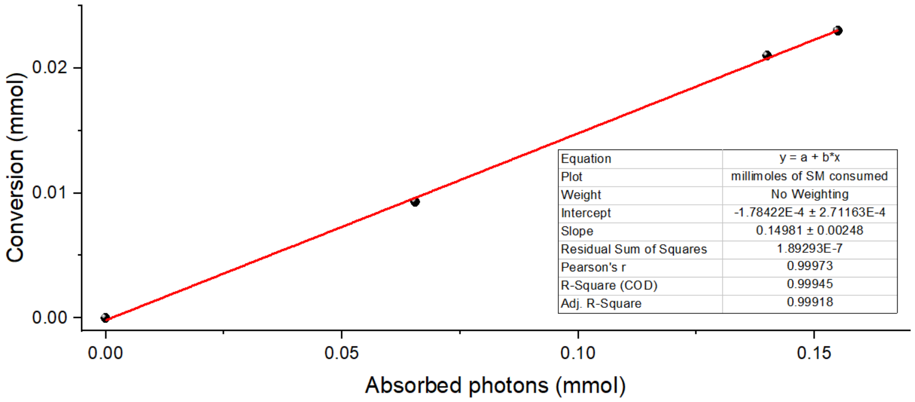

Figure S116: Plot of starting material conversion vs absorbed photons and linear interpolation.

Quantum yield of the reduction of 7

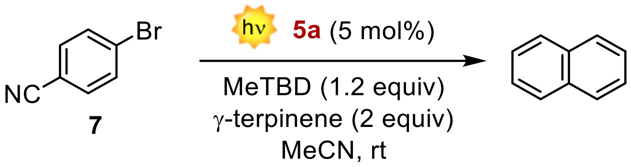

The same cuvette used for the actinometric measurements was charged with 4-Bromobenzonitrile **7** (18,2 mg, 0.1 mmol) and PC **5a** (5 mol%) and degassed with Ar. 1 mL of already degassed MeCN was added, followed by γ-terpinene (32 µL, 0.2 mmol, 2 equiv) and MeTBD (1.2 mmol, 1.2 equiv). The reaction mixture was purged with Ar for 1 minute.

Following the same procedure described before, we obtained a QY value $\phi < 0.10$.

I. References

(1) Ragazzon, G.; Schäfer, C.; Franchi, P.; Silvi, S.; Colasson, B.; Lucarini, M.; Credi, A. Remote Electrochemical Modulation of PKa in a Rotaxane by Co-Conformational Allostery. *Proc Natl Acad Sci U S A* **2018**, *115* (38), 9385–9390.

(2) Wang, S.; Force, G.; Carpentier, J. F.; Sarazin, Y.; Bour, C.; Gandon, V.; Lebœuf, D.; Lebœuf, D. Modular Synthesis of 9,10-Dihydroacridines through an Ortho-C Alkenylation/Hydroarylation Sequence between Anilines and Aryl Alkynes in Hexafluoroisopropanol. *Org Lett* **2021**, *23* (7), 2565–2570.

(3) Chernowsky, C. P.; Chmiel, A. F.; Wickens, Z. K. Electrochemical Activation of Diverse Conventional Photoredox Catalysts Induces Potent Photoreductant Activity**. *Angewandte Chemie* **2021**, *133* (39), 21588–21595.

(4) Wang, J.; Xu, B.; Sun, H.; Song, G. Palladium Nanoparticles Supported on Functional Ionic Liquid Modified Magnetic Nanoparticles as Recyclable Catalyst for Room Temperature Suzuki Reaction. *Tetrahedron Lett* **2013**, *54* (3), 238–241.

(5) Babin, V.; Sallustrau, A.; Loreau, O.; Caillé, F.; Goudet, A.; Cahuzac, H.; del Vecchio, A.; Taran, F.; Audisio, D. A General Procedure for Carbon Isotope Labeling of Linear Urea Derivatives with Carbon Dioxide. *Chemical Communications* **2021**, *57* (54), 6680–6683.

(6) Chao, S.; Romuald, C.; Fournel-Marotte, K.; Clavel, C.; Coutrot, F. A Strategy Utilizing a Recyclable Macrocycle Transporter for the Efficient Synthesis of a Triazolium-Based [2]Rotaxane. *Angewandte Chemie International Edition* **2014**, *53* (27), 6914–6919.

(7) Zhong, Z.; Xu, P.; Zhou, A. Electrochemical Phosphorylation of Arenols and Anilines Leading to Organophosphates and Phosphoramidates. *Org Biomol Chem* **2021**, *19* (24), 5342–5347.

(8) Zhuang, R.; Xu, J.; Cai, Z.; Tang, G.; Fang, M.; Zhao, Y. Copper-Catalyzed C-P Bond Construction via Direct Coupling of Phenylboronic Acids with H-Phosphonate Diesters. *Org Lett* **2011**, *13* (8), 2110–2113.

(9) Yi, Y. Q. Q.; Yang, W. C.; Zhai, D. D.; Zhang, X. Y.; Li, S. Q.; Guan, B. T. Nickel-Catalyzed C–N Bond Reduction of Aromatic and Benzylic Quaternary Ammonium Triflates. *Chemical Communications* **2016**, *52* (72), 10894–10897.

(10) Hu, J.; Sun, H.; Cai, W.; Pu, X.; Zhang, Y.; Shi, Z. Nickel-Catalyzed Borylation of Aryl- and Benzyltrimethylammonium Salts via C-N Bond Cleavage. *Journal of Organic Chemistry* **2016**, *81* (1), 14–24.

(11) Tayama, E.; Sotome, S. Dearomative [2,3] Sigmatropic Rearrangement of Ammonium Ylides Followed by 1,4-Elimination to Form α-(Ortho-Vinylphenyl)Amino Acid Esters. *Org Biomol Chem* **2018**, *16* (26), 4833–4839.

(12) Miyano, S.; Lu, L. D. L.; Viti, S. M.; Barry Sharpless, K. Kinetic Resolution of Racemic β-Hydroxy Amines by Enantioselective N-Oxide Formation. *Journal of Organic Chemistry* **1985**, *50* (22), 4350–4360.

(13) Würth, C.; Grabolle, M.; Pauli, J.; Spieles, M.; Resch-Genger, U. Relative and Absolute Determination of Fluorescence Quantum Yields of Transparent Samples. *Nature Protocols 2013 8:8* **2013**, *8* (8), 1535–1550.

(14) Rehm, D.; Weller, A. Kinetics of Fluorescence Quenching by Electron and H-Atom Transfer. *Isr J Chem* **1970**, *8* (2), 259–271.

(15) Romero, N. A.; Nicewicz, D. A. Organic Photoredox Catalysis. *Chem Rev* **2016**, *116* (17), 10075–10166.

(16) Discekici, E. H.; Treat, N. J.; Poelma, S. O.; Mattson, K. M.; Hudson, Z. M.; Luo, Y.; Hawker, C. J.; de Alaniz, J. R. A Highly Reducing Metal-Free Photoredox Catalyst: Design and Application in Radical Dehalogenations. *Chemical Communications* **2015**, *51* (58), 11705–11708.

(17) Vogt, D. B.; Seath, C. P.; Wang, H.; Jui, N. T. Selective C-F Functionalization of Unactivated Trifluoromethylarenes. *J Am Chem Soc* **2019**, *141* (33), 13203–13211.

(18) Yan, Z.; Yuan, X.-A.; Zhu, C.; Xie, J.; Yan,] Z; Hao, Y. Z.; Zhu, C.; Xie, J.; Yuan, X.-A.; Hu, C. Z. Selective Hydroarylation of 1,3-Diynes Using a Dimeric Manganese Catalyst: Modular Synthesis of Z-Enynes. *Angewandte Chemie International Edition* **2018**, *57* (39), 12906–12910.

(19) Huang, J. L.; Dai, X. J.; Li, C. J. Iridium-Catalyzed Direct Dehydroxylation of Alcohols. *European J Org Chem* **2013**, *2013* (29), 6496–6500.

(20) Kariofillis, S. K.; Jiang, S.; Żurański, A. M.; Gandhi, S. S.; Martinez Alvarado, J. I.; Doyle, A. G. Using Data Science To Guide Aryl Bromide Substrate Scope Analysis in a Ni/Photoredox-Catalyzed Cross-Coupling with Acetals as Alcohol-Derived Radical Sources. *J Am Chem Soc* **2022**, *144* (2), 1045–1055.

(21) Ke, J.; Wang, H.; Zhou, L.; Mou, C.; Zhang, J.; Pan, L.; Chi, Y. R. Hydrodehalogenation of Aryl Halides through Direct Electrolysis. *Chemistry – A European Journal* **2019**, *25* (28), 6911–6914.

(22) Ushijima, S.; Moriyama, K.; Togo, H. Facile Preparation of Aromatic Esters from Aromatic Bromides with Ethyl Formate or DMF and Molecular Iodine via Aryllithium. *Tetrahedron* **2012**, *68* (24), 4701–4709.

(23) Malapit, C. A.; Visco, M. D.; Reeves, J. T.; Busacca, C. A.; Howell, A. R.; Senanayake, C. H. Nickel- or Cobalt-Catalyzed Cross-Coupling of Arylsulfonic Acid Salts with Grignard Reagents. *Adv Synth Catal* **2015**, *357* (10), 2199–2204.

(24) Wang, H.; Li, L.; Bai, X. F.; Shang, J. Y.; Yang, K. F.; Xu, L. W. Efficient Palladium-Catalyzed C-O Hydrogenolysis of Benzylic Alcohols and Aromatic Ketones with Polymethylhydrosiloxane. *Adv Synth Catal* **2013**, *355* (2–3), 341–347.

(25) Plank, T. N.; Drake, J. L.; Kim, D. K.; Funk, T. W. Air-Stable, Nitrile-Ligated (Cyclopentadienone)Iron Dicarbonyl Compounds as Transfer Reduction and Oxidation Catalysts. *Adv Synth Catal* **2012**, *354* (4), 597–601.

(26) Sworakowski, J.; Lipiński, J.; Janus, K. On the Reliability of Determination of Energies of HOMO and LUMO Levels in Organic Semiconductors from Electrochemical Measurements. A Simple Picture Based on the Electrostatic Model. *Org Electron* **2016**, *33*, 300–310.

(27) Sworakowski, J. How Accurate Are Energies of HOMO and LUMO Levels in Small-Molecule Organic Semiconductors Determined from Cyclic Voltammetry or Optical Spectroscopy? *Synth Met* **2018**, *235*, 125–130.

(28) Murov, S. L. *Handbook of Photochemistry*; Marcel Dekker, New York, 1973.

J. NMR spectra

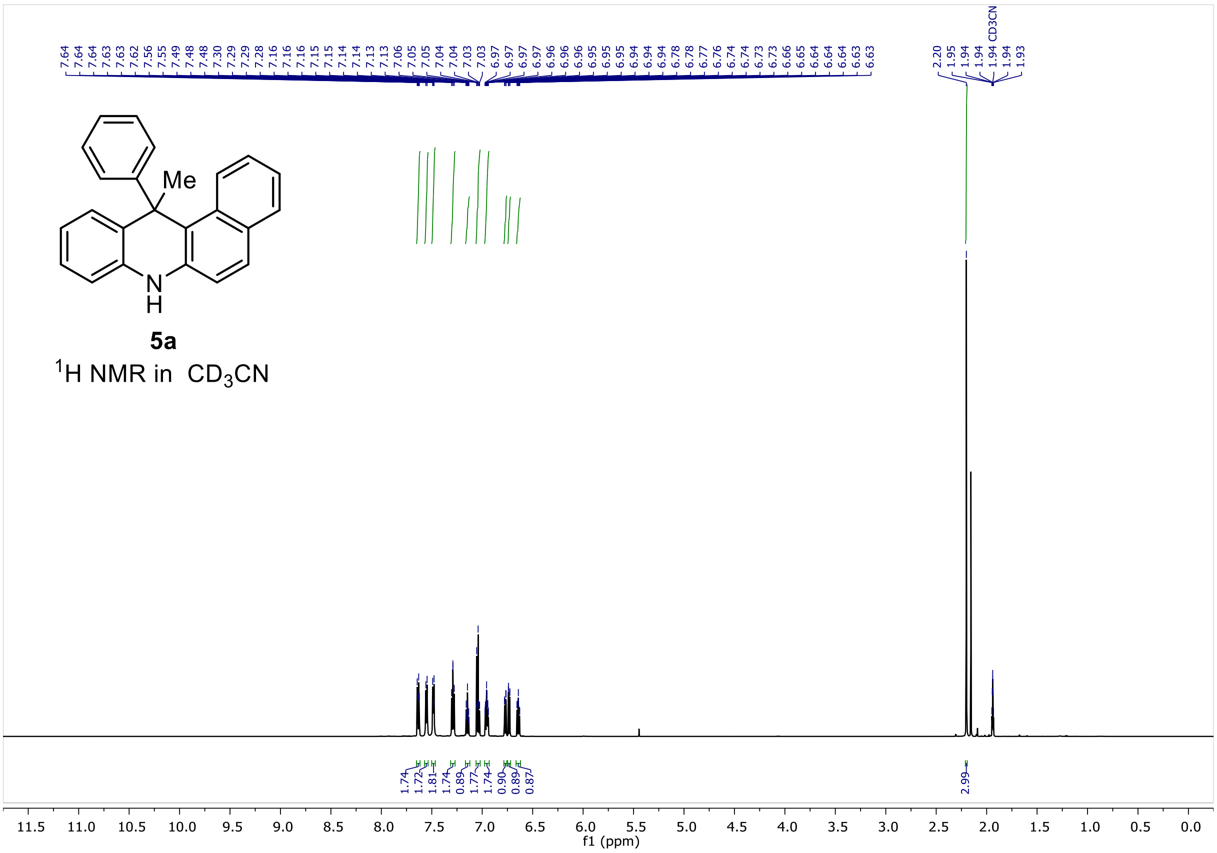

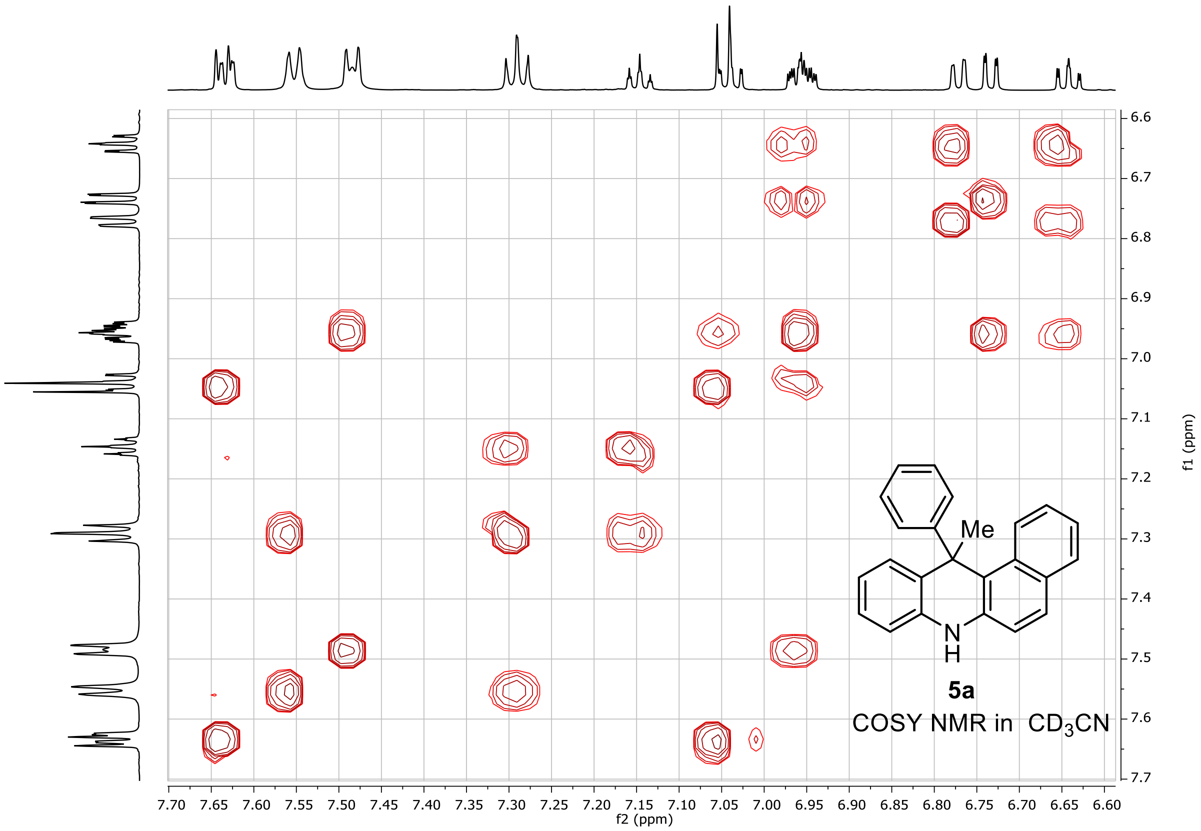

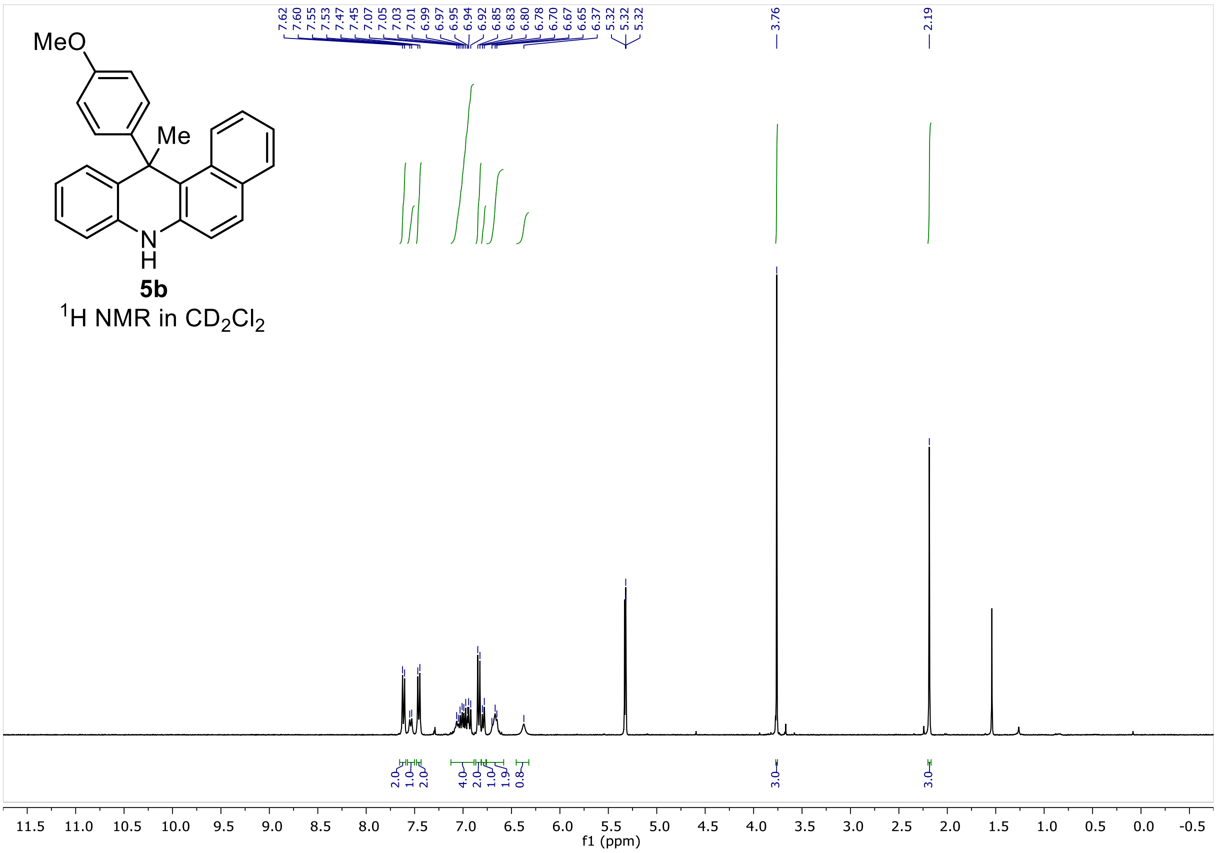
MeO
Me
N
H
5b
^{1}H NMR in CD_2Cl_2
7.62 7.60 7.55 7.53 7.47 7.45 7.07 7.05 7.03 7.01 6.99 6.97 6.95 6.94 6.92 6.85 6.83 6.80 6.78 6.70 6.67 6.65 6.37 5.32 5.32 5.32
3.76
2.19
2.0 1.0 2.0 4.0 2.0 1.0 1.9 0.8
3.0
3.0
11.5 11.0 10.5 10.0 9.5 9.0 8.5 8.0 7.5 7.0 6.5 6.0 5.5 5.0 4.5 4.0 3.5 3.0 2.5 2.0 1.5 1.0 0.5 0.0 -0.5
f1 (ppm)

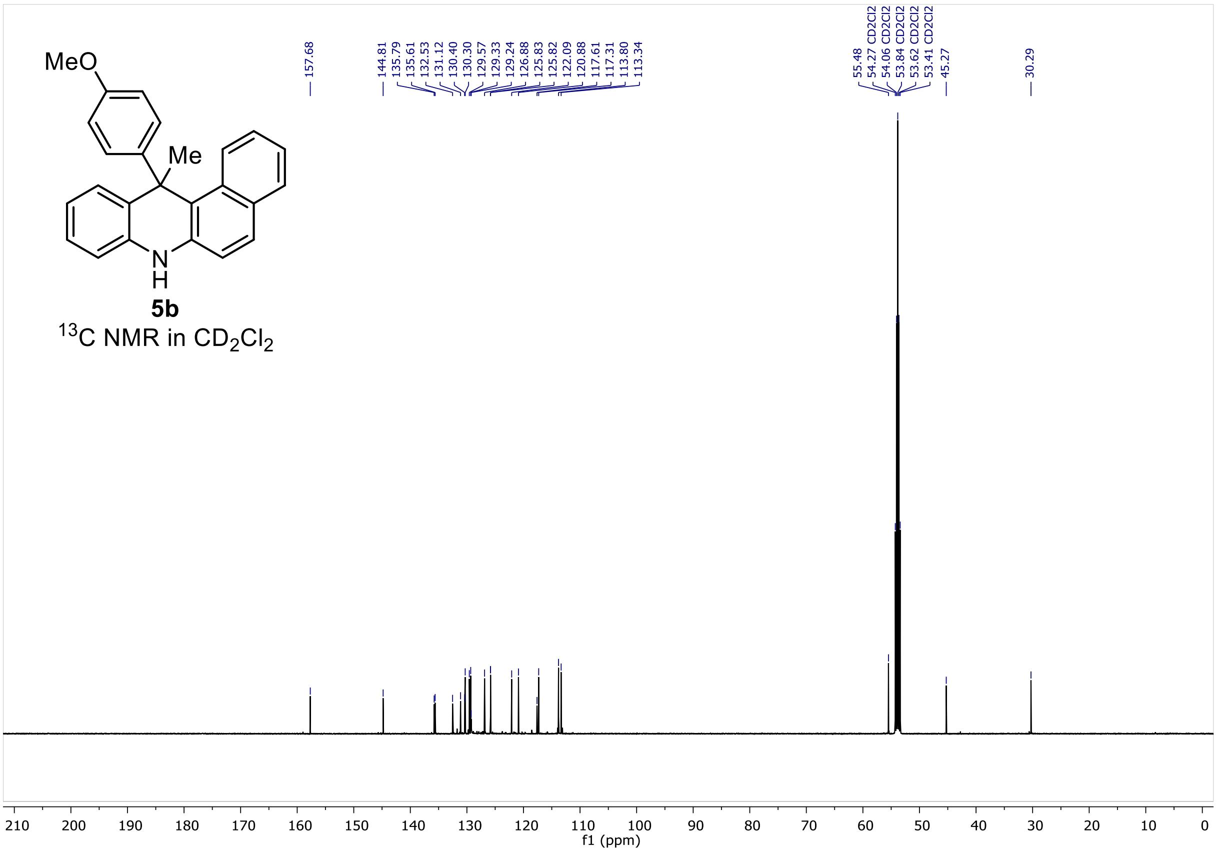
MeO
Me
N
H
5b
^{13}C NMR in CD_2Cl_2
157.68
144.81 135.79 135.61 132.53 131.12 130.40 130.30 129.57 129.33 129.24 126.88 125.83 125.82 122.09 120.88 117.61 117.31 113.80 113.34
55.48 54.27 CD2Cl2 54.06 CD2Cl2 53.84 CD2Cl2 53.62 CD2Cl2 53.41 CD2Cl2
45.27
30.29
210 200 190 180 170 160 150 140 130 120 110 100 90 80 70 60 50 40 30 20 10 0
f1 (ppm)

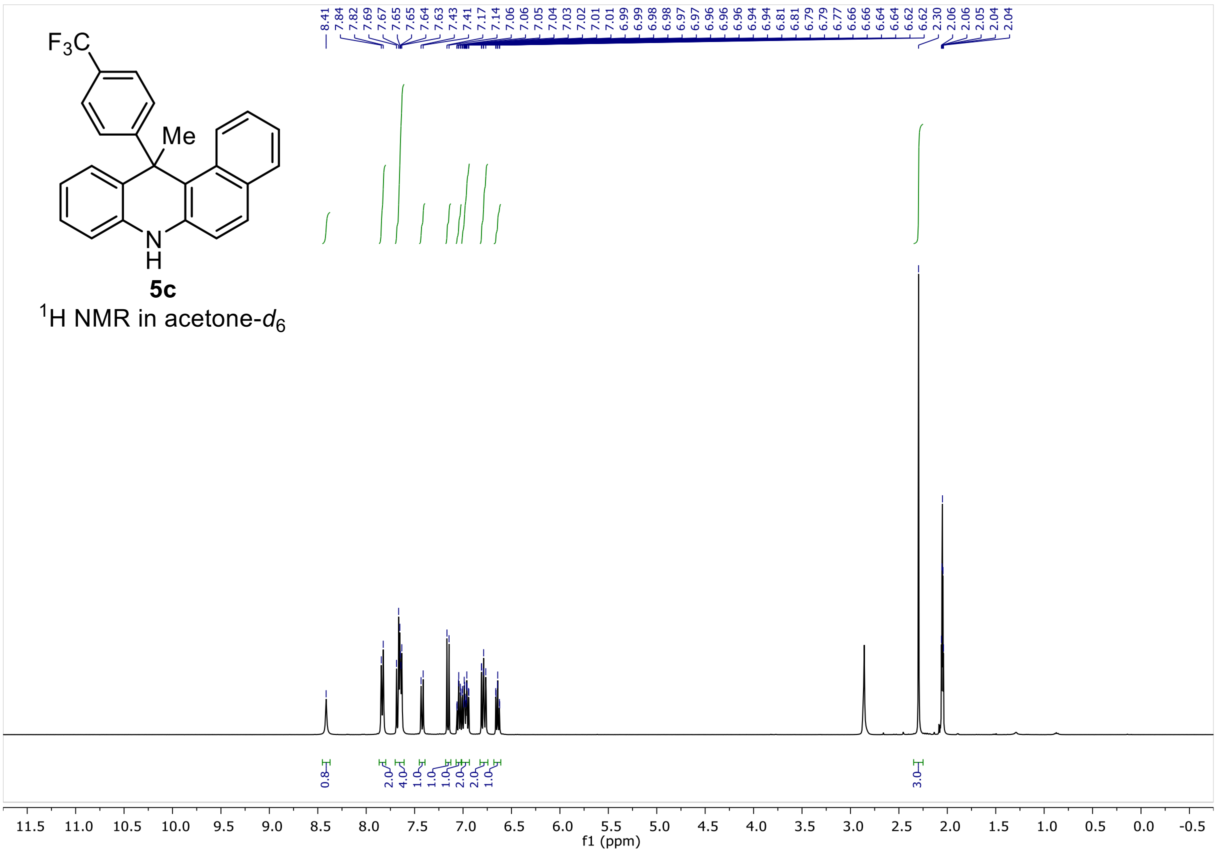

F_3C
Me
N
H
5c
^{1}H NMR in acetone-d_6
8.41
7.84
7.82
7.69
7.67
7.65
7.65
7.64
7.63
7.43
7.41
7.17
7.14
7.06
7.06
7.05
7.04
7.03
7.02
7.01
7.01
6.99
6.99
6.98
6.97
6.97
6.96
6.96
6.96
6.94
6.94
6.81
6.81
6.79
6.79
6.77
6.66
6.66
6.64
6.64
6.62
6.62
2.30
2.06
2.06
2.05
2.04
2.04
0.8
2.0
4.0
1.0
1.0
1.0
2.0
2.0
1.0
3.0
f1 (ppm)

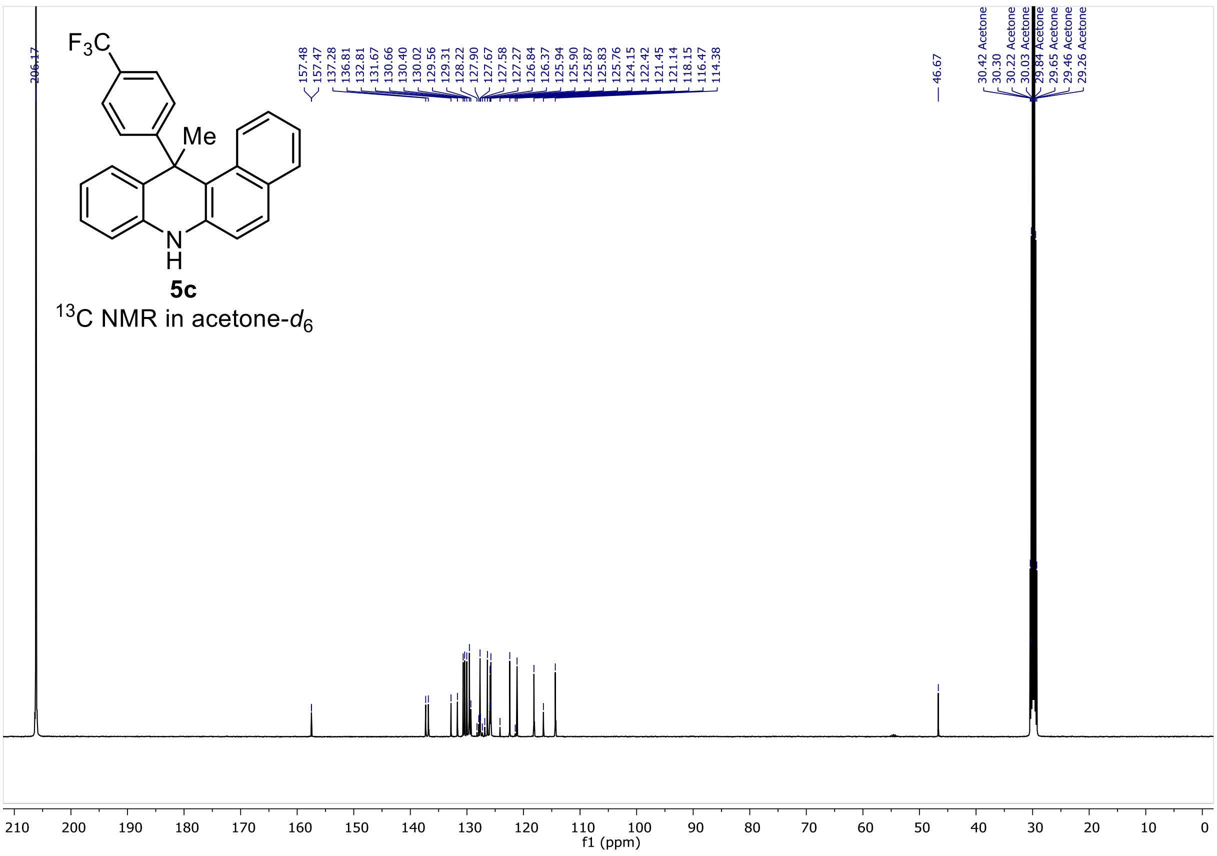

206.17
F_3C
Me
N
H
5c
^{13}C NMR in acetone-d_6
157.48
157.47
137.28
136.81
132.81
131.67
130.66
130.40
130.02
129.56
129.31
128.22
127.90
127.67
127.58
127.27
126.84
126.37
125.94
125.90
125.87
125.83
125.76
124.15
122.42
121.45
121.14
118.15
116.47
114.38
46.67
30.42 Acetone
30.30
30.22 Acetone
30.03 Acetone
29.84 Acetone
29.65 Acetone
29.46 Acetone
29.26 Acetone
f1 (ppm)

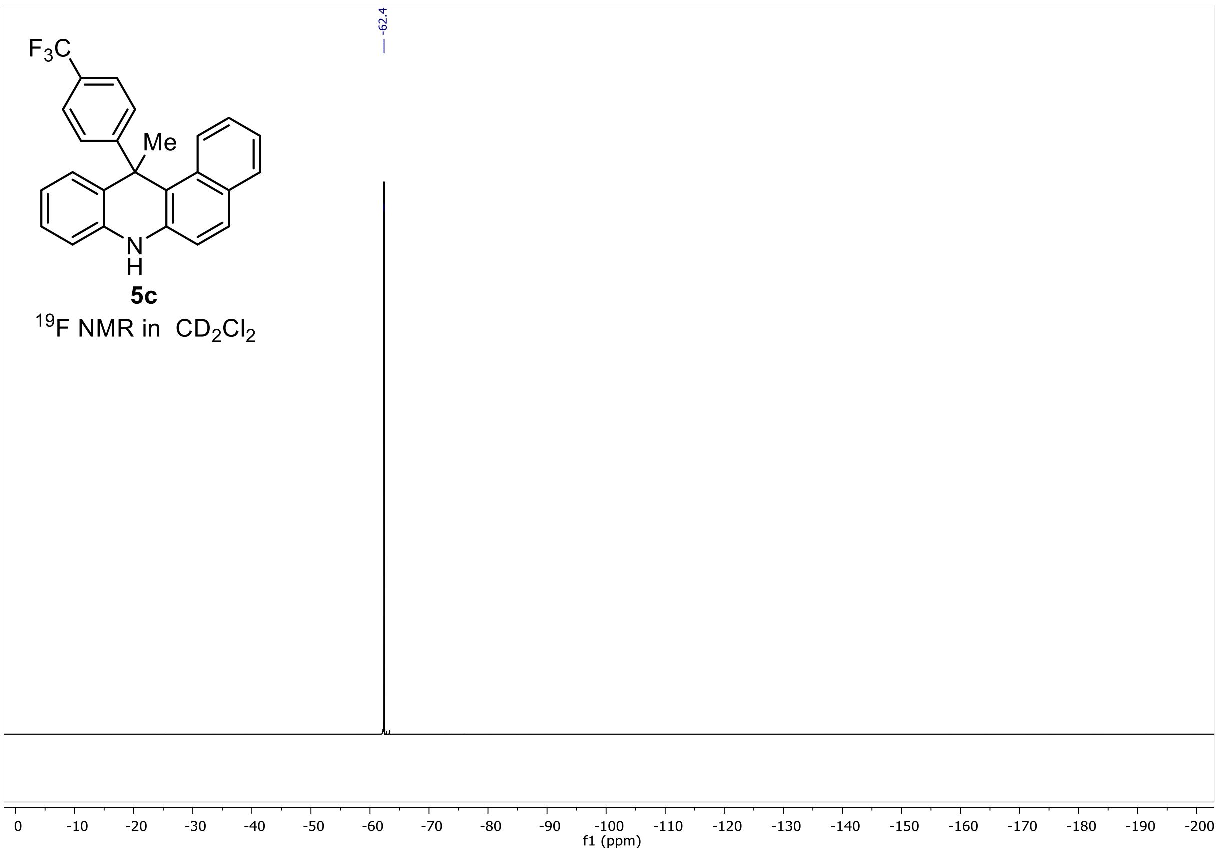

F3C
Me
N
H
5c
19F NMR in CD2Cl2
-62.4
f1 (ppm)

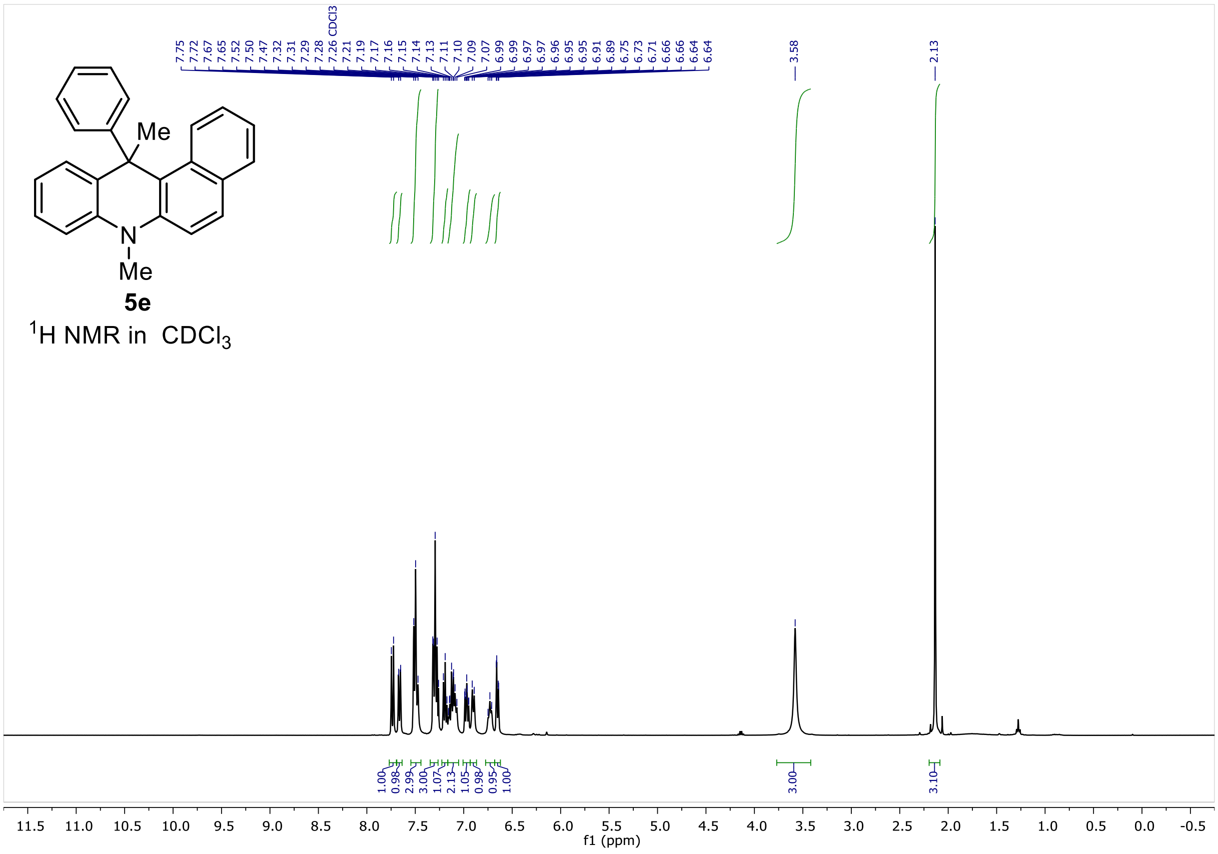

Me
N
Me
5e
1H NMR in CDCl3
3.58
2.13
f1 (ppm)

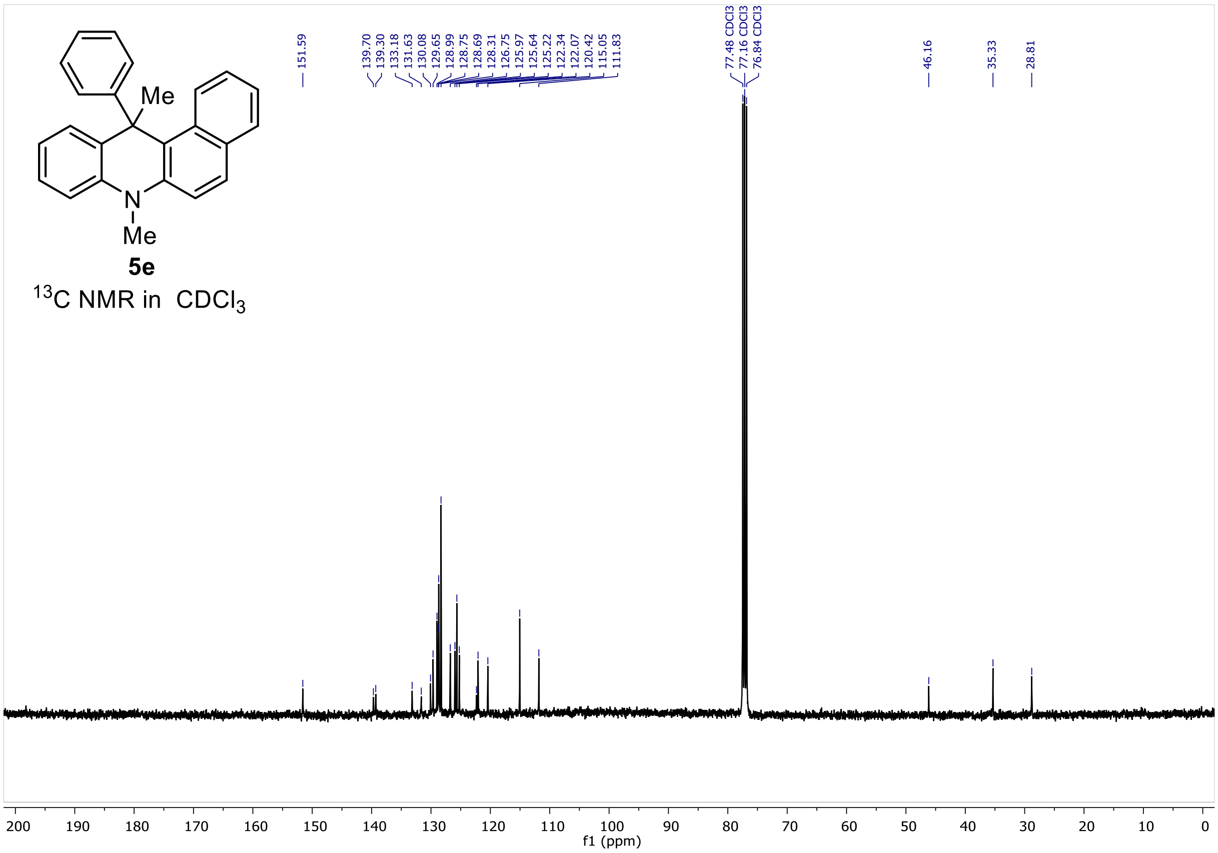
Me
N
Me
5e
13C NMR in CDCl3
151.59
139.70
139.30
133.18
131.63
130.08
129.65
128.99
128.75
128.69
128.31
126.75
125.97
125.64
125.22
122.34
122.07
120.42
115.05
111.83
77.48 CDCl3
77.16 CDCl3
76.84 CDCl3
46.16
35.33
28.81
f1 (ppm)

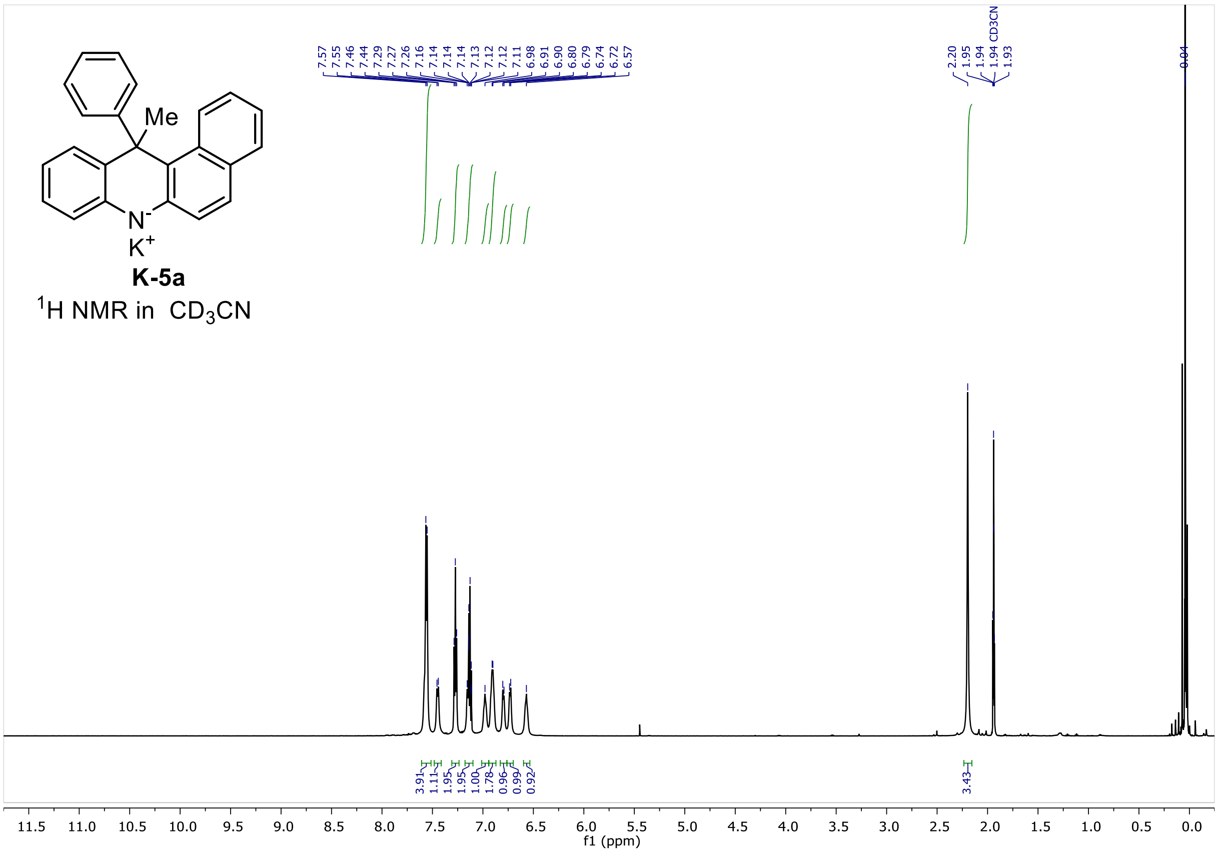
Me
N-
K+
K-5a
1H NMR in CD3CN
7.57
7.55
7.46
7.44
7.29
7.27
7.26
7.16
7.14
7.14
7.13
7.12
7.11
6.98
6.91
6.90
6.80
6.79
6.74
6.72
6.57
2.20
1.95
1.94 CD3CN
1.94
1.93
0.04
3.91
1.11
1.95
1.95
1.00
1.78
0.96
0.99
0.92
3.43
f1 (ppm)

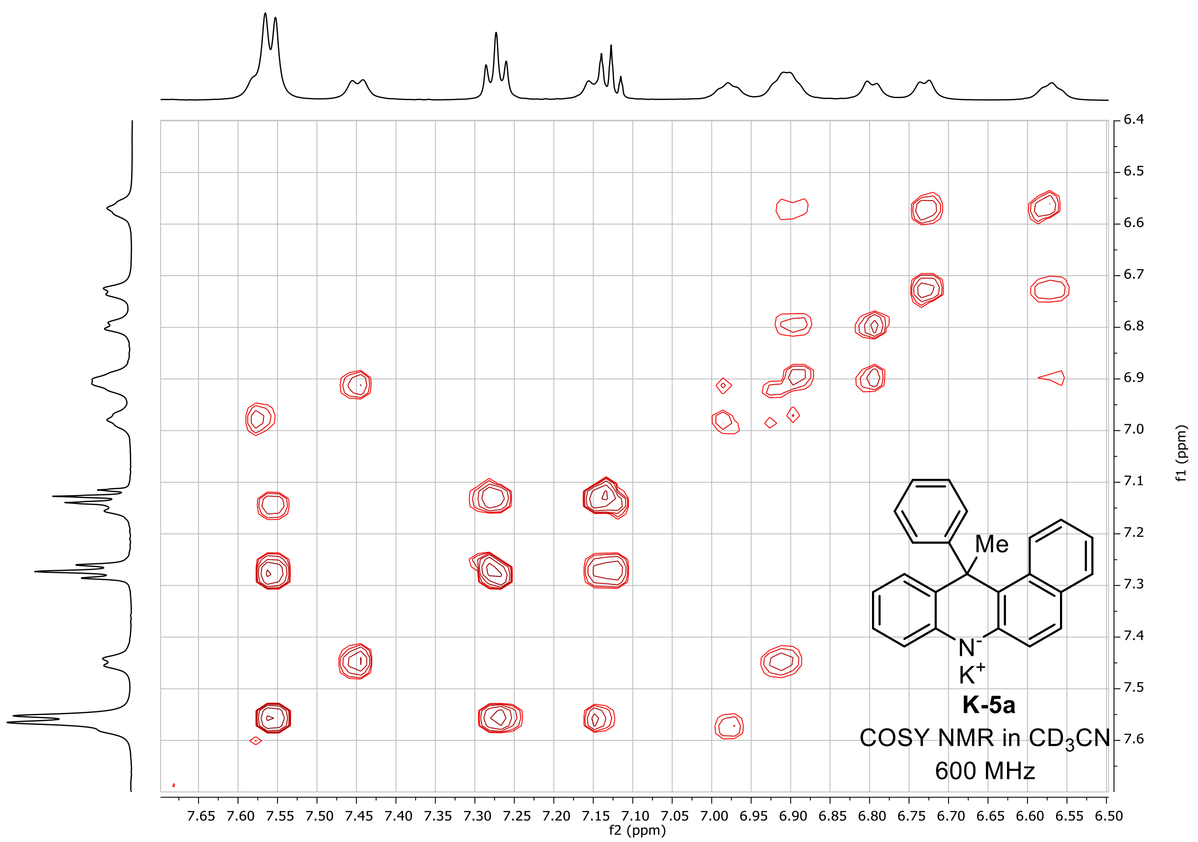
K-5a
COSY NMR in CD$_3$CN
600 MHz
f2 (ppm)
f1 (ppm)

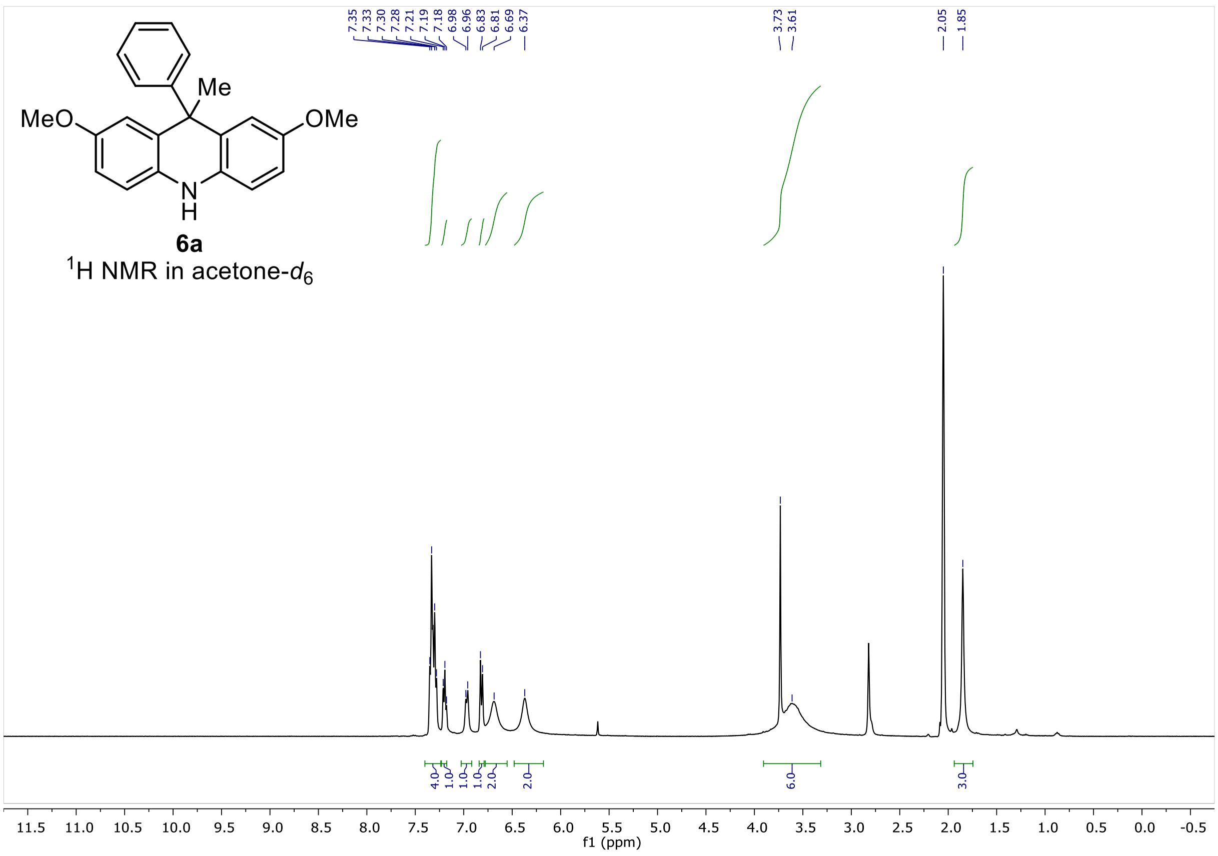
6a
^{1}H NMR in acetone-d_6
f1 (ppm)

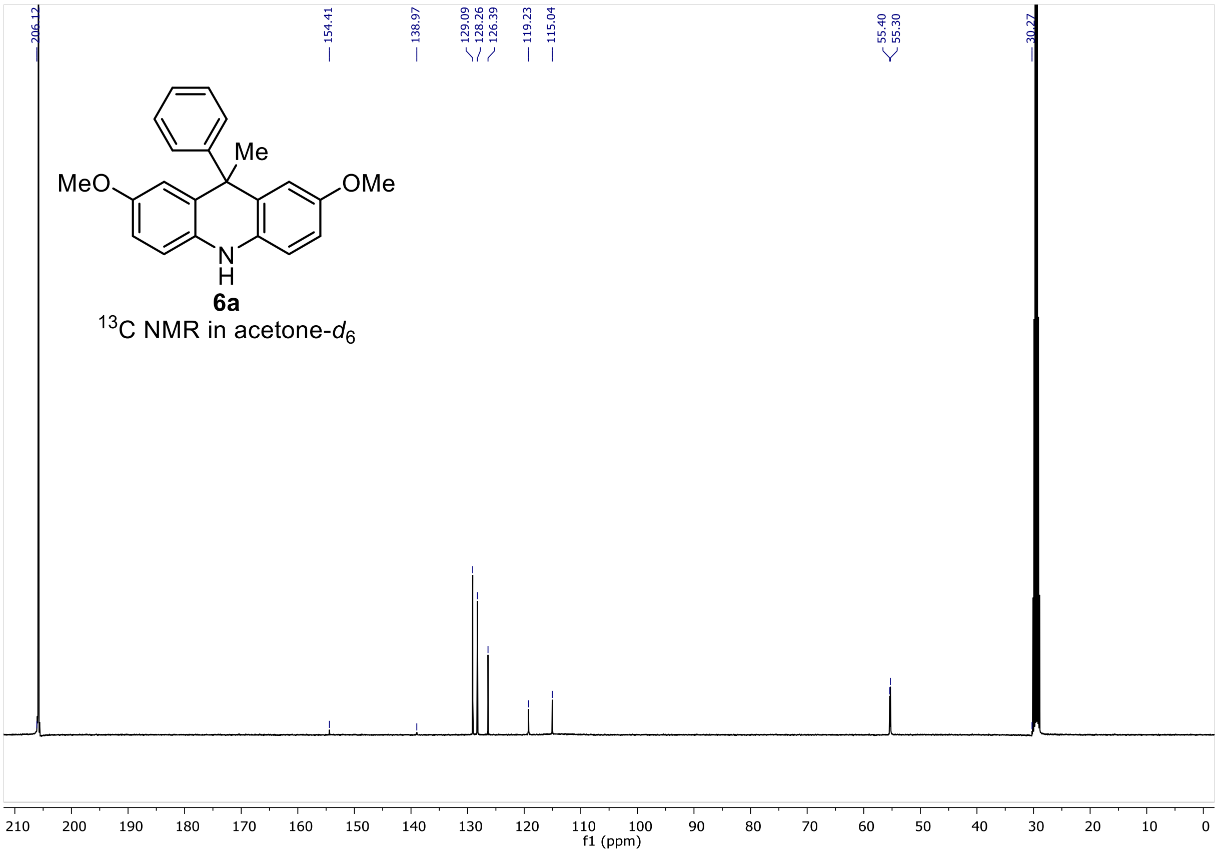
206.12
154.41
138.97
129.09
128.26
126.39
119.23
115.04
55.40
55.30
30.27
MeO
OMe
Me
N
H
6a
^{13}C NMR in acetone-d_6
f1 (ppm)

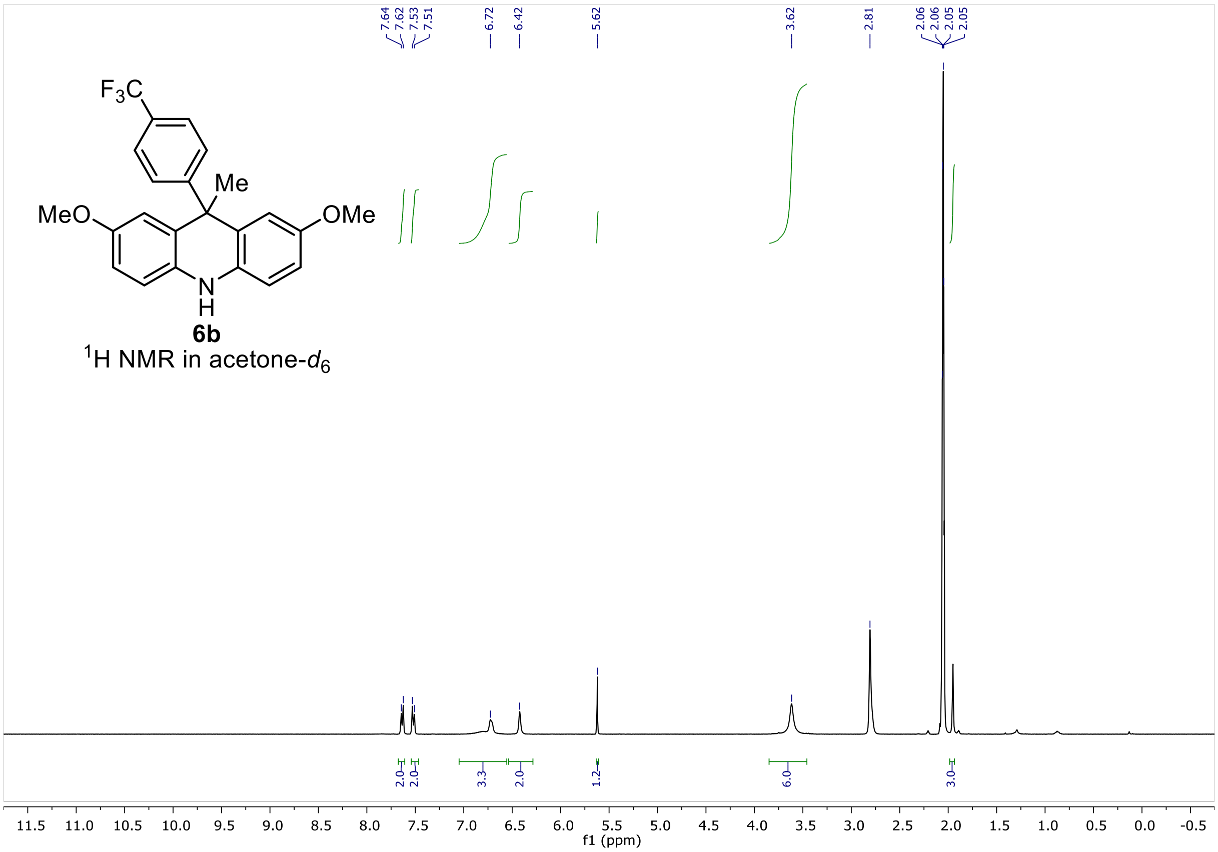
7.64
7.62
7.53
7.51
6.72
6.42
5.62
3.62
2.81
2.06
2.06
2.05
2.05
F_3C
MeO
OMe
Me
N
H
6b
^{1}H NMR in acetone-d_6
2.0
2.0
3.3
2.0
1.2
6.0
3.0
f1 (ppm)

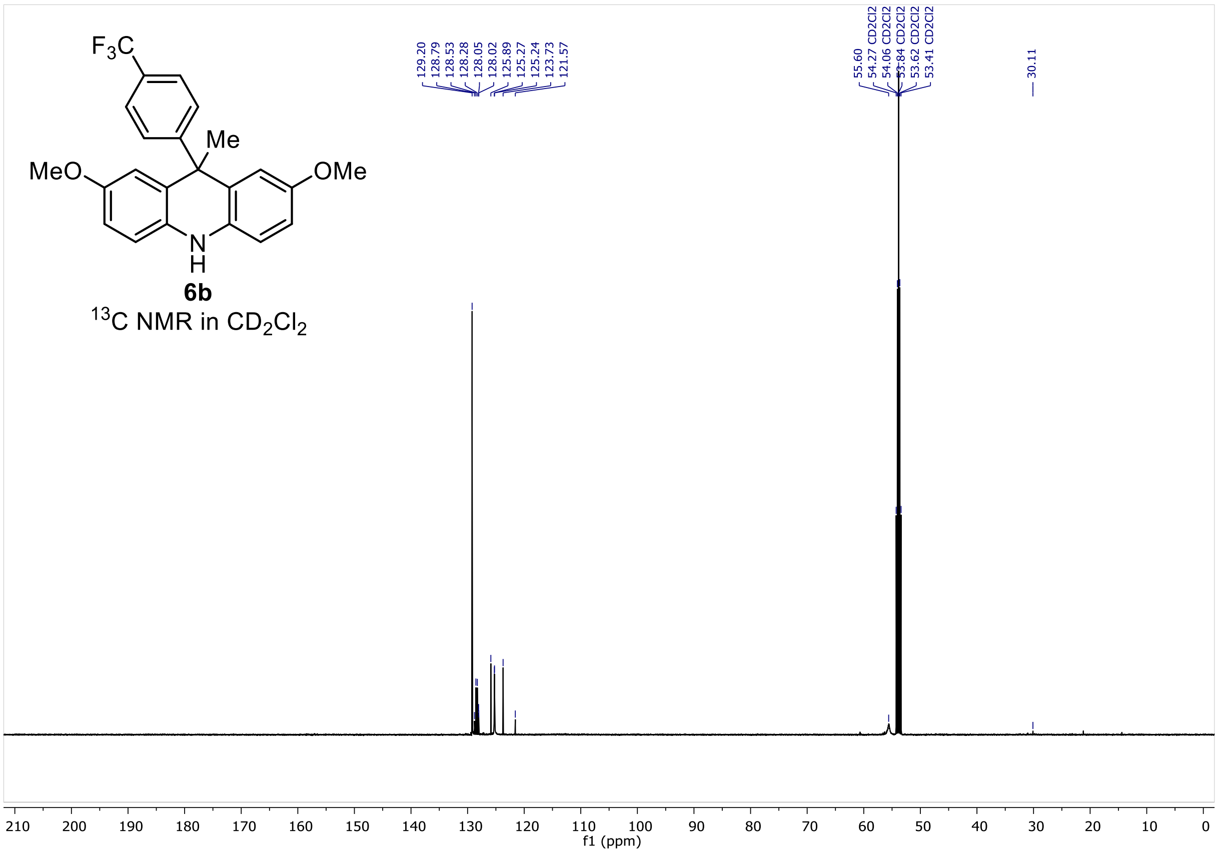
F3C
Me
MeO
OMe
N
H
6b
13C NMR in CD2Cl2
129.20
128.79
128.53
128.28
128.05
128.02
125.89
125.27
125.24
123.73
121.57
55.60
54.27 CD2Cl2
54.06 CD2Cl2
53.84 CD2Cl2
53.62 CD2Cl2
53.41 CD2Cl2
30.11
f1 (ppm)

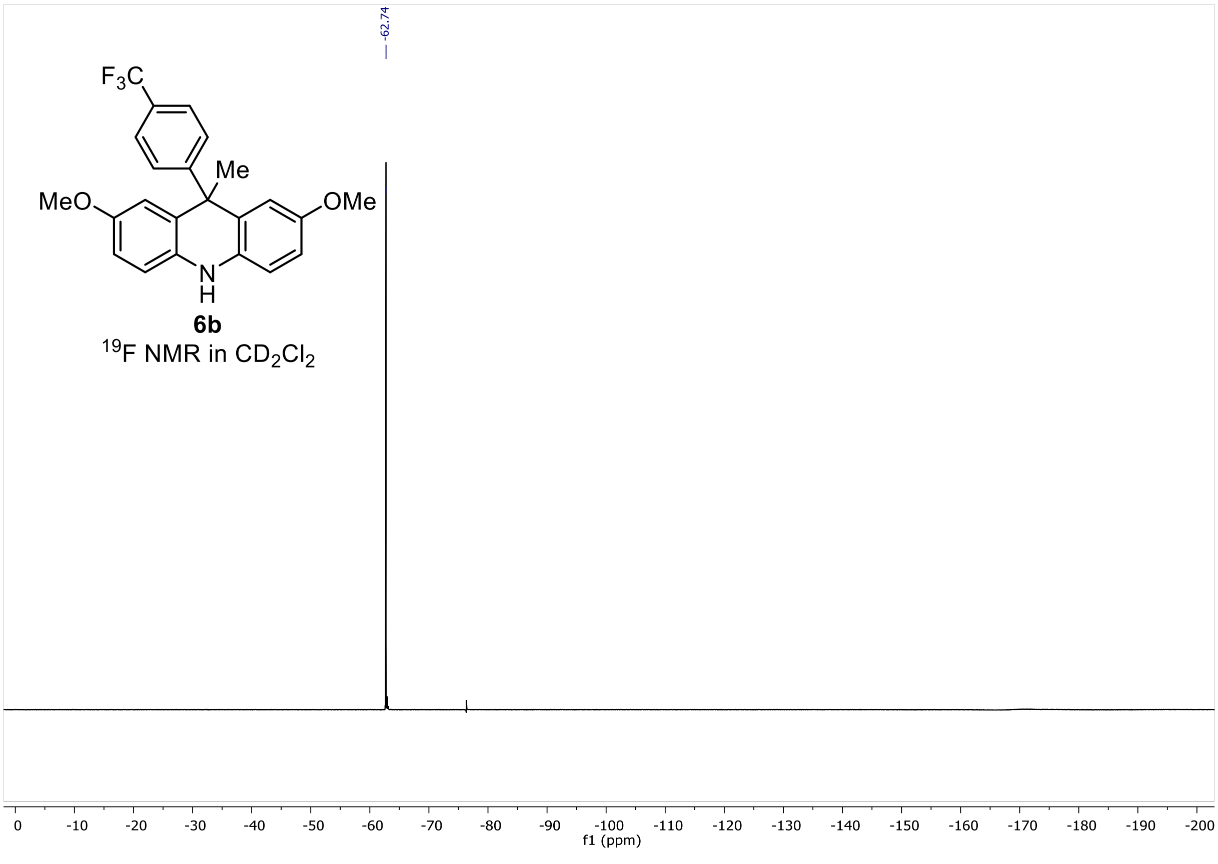
-62.74
F3C
Me
MeO
OMe
N
H
6b
19F NMR in CD2Cl2
f1 (ppm)

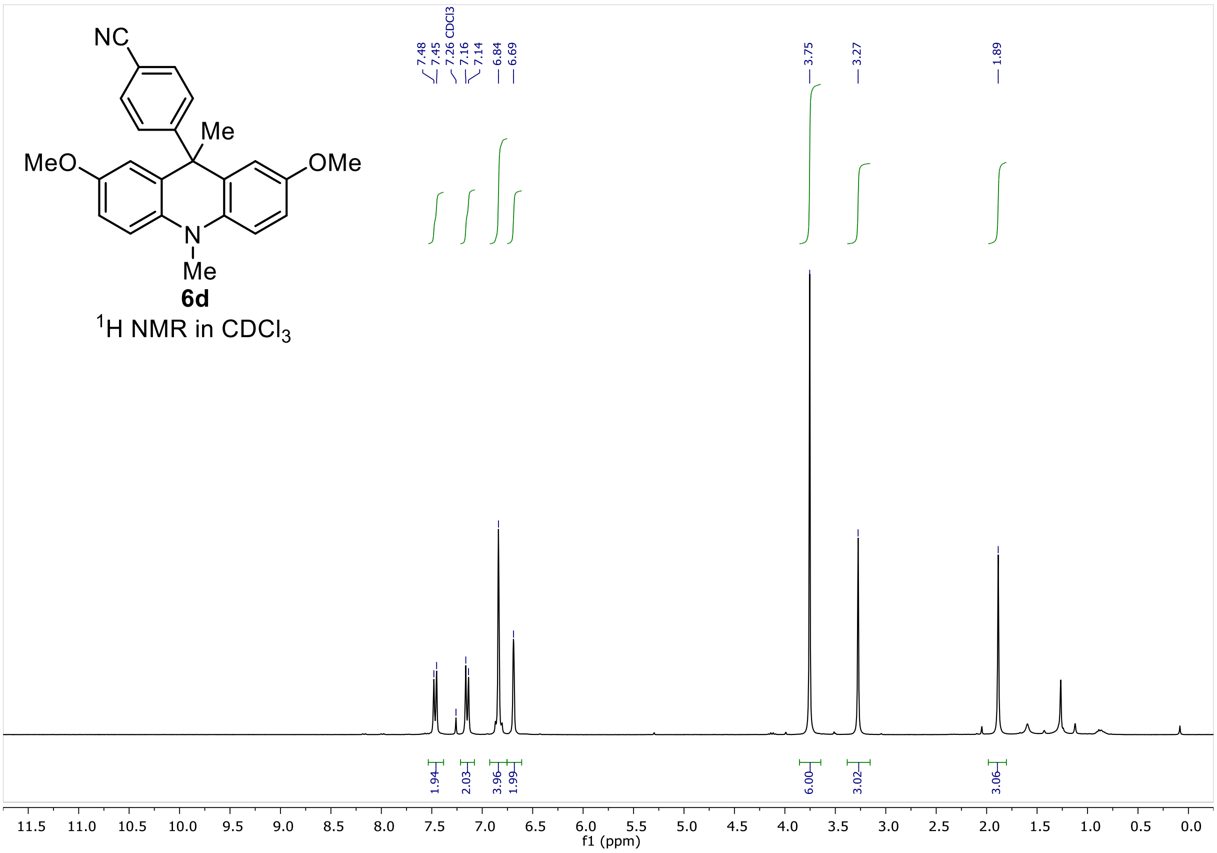
NC
Me
MeO
OMe
N
Me
6d
1H NMR in CDCl3
7.48
7.45
7.26 CDCl3
7.16
7.14
6.84
6.69
3.75
3.27
1.89
1.94
2.03
3.96
1.99
6.00
3.02
3.06
11.5 11.0 10.5 10.0 9.5 9.0 8.5 8.0 7.5 7.0 6.5 6.0 5.5 5.0 4.5 4.0 3.5 3.0 2.5 2.0 1.5 1.0 0.5 0.0
f1 (ppm)

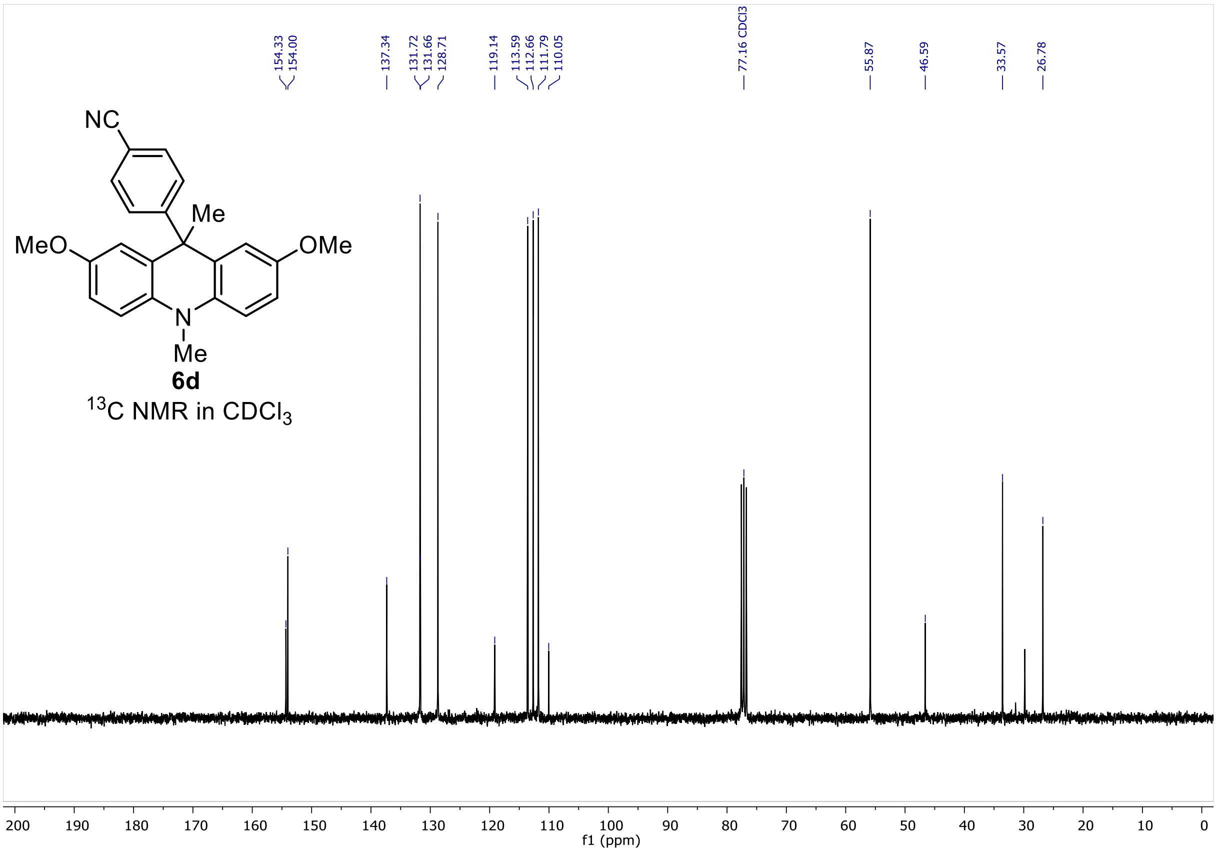
154.33
154.00
137.34
131.72
131.66
128.71
119.14
113.59
112.66
111.79
110.05
77.16 CDCl3
55.87
46.59
33.57
26.78
NC
Me
MeO
OMe
N
Me
6d
13C NMR in CDCl3
200 190 180 170 160 150 140 130 120 110 100 90 80 70 60 50 40 30 20 10 0
f1 (ppm)
